# METTL3–METTL14 Heterodimer Assembly Enables Multivalent RNA Engagement and Is Tuned by Interface-Targeting Peptides

**DOI:** 10.1021/jacs.6c09634

**Published:** 2026-10-07

**Authors:** Francesco Fiorentino, Boyi Xiao, Bianca Cesaro, Sara Venezia, Jujun Zhou, Emanuele Fabbrizi, Fabrizio Casano, Gebremedhin Solomon Hailu, Xiaodong Cheng, Robin A. Corey, Alessandro Fatica, Antonello Mai, Stefano Tomassi, Dante Rotili

**Affiliations:** † Department of Biochemical Sciences ″A. Rossi Fanelli″, 9311 Sapienza University of Rome, Piazzale Aldo 5, 00185 Rome, Italy; ‡ School of Physiology, Pharmacology & Neuroscience, 1980 University of Bristol, Biomedical Sciences Building, University Walk, Bristol BS8 1TD, U.K.; § Department of Biology and Biotechnologies ″Charles Darwin″, Sapienza University of Rome, Piazzale Aldo 5, 00185 Rome, Italy; ∥ Department of Epigenetics and Molecular Carcinogenesis, 4002 University of Texas MD Anderson Cancer Center, Houston, Texas 77030, United States; ⊥ Department of Drug Chemistry and Technologies, Sapienza University of Rome, Piazzale Aldo 5, 00185 Rome, Italy; # Department of Science, Roma Tre University, Viale Marconi 446, 00146 Rome, Italy; ∇ Department of Life Sciences, Health and Health Professions, 207131 Link Campus University, Via del Casale di S. Pio V, 44, 00165 Roma, Italy; ○ Biostructures and Biosystems National Institute (INBB), Via dei Carpegna 19, 00165 Rome, Italy

## Abstract

RNA N^6^-methyladenosine (m^6^A) deposition by the METTL3–METTL14 complex regulates RNA metabolism and thereby influences cell differentiation and stress responses. Dysregulated m^6^A deposition has been implicated in multiple cancers, where METTL3–METTL14 supports oncogenic programs. Yet the molecular basis of RNA recognition by this complex remains unclear, in part due to the lack of an RNA-bound structure of the full assembly. Here, we combined native mass spectrometry (MS), molecular modeling, biochemical assays, and cell-based studies to define how METTL3–METTL14 engages RNA and how this process can be perturbed through interface targeting. Native MS of the intact heterodimer revealed simultaneous binding of multiple RNA molecules, with RNA-dependent stoichiometries and higher-occupancy states consistent with cooperative interactions. Molecular dynamics simulations identified a structured RNA-bound assembly in which the METTL14 C-terminal RGG-rich region stabilizes two RNA molecules, supporting a pre-engaged substrate configuration. We further developed a focused library of METTL14-derived peptides that bind METTL3 and interfere with protein–protein interactions, identifying the minimal METTL14 epitope for interface engagement. The most potent peptide, **R8–14**, binds with submicromolar affinity, disrupts both METTL3–METTL14 association and higher-order RNA-bound states, and reduces m^6^A levels, c-Myc expression, and cancer cell viability. Together, these findings demonstrate that RNA recognition by METTL3–METTL14 is governed by multivalent, higher-order interactions that enhance binding avidity. Notably, this assembly can be perturbed allosterically through protein–protein interface targeting rather than active-site inhibition, highlighting multivalent RNA recognition as a mechanistically and pharmacologically actionable feature of the RNA m^6^A writer complex.

## Graphical abstract

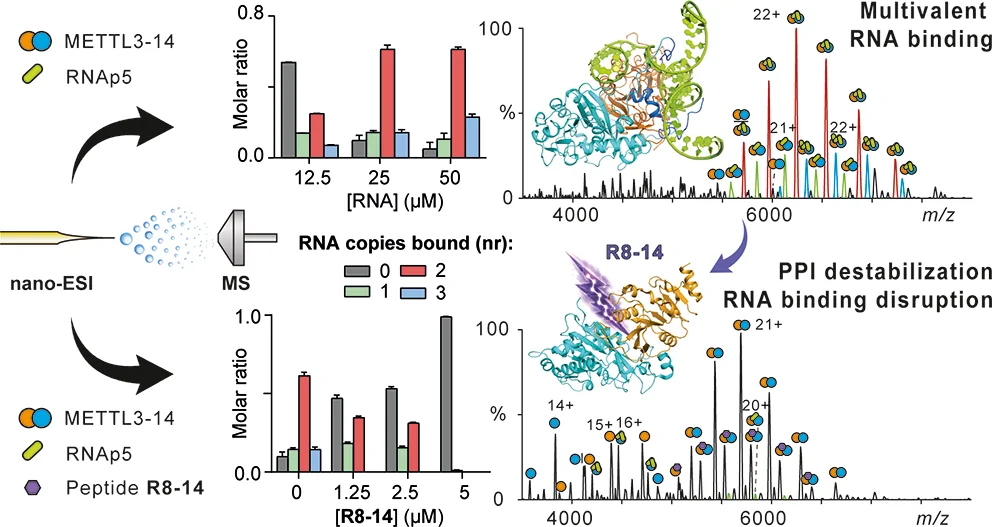

## Introduction

The most abundant internal RNA modification in eukaryotic mRNAs and long noncoding RNAs is N^6^-methyladenosine (m^6^A), which modulates splicing, nuclear export, translation, and stability through context-dependent effects on RNA structure and RNA-protein interactions.^1–3^ In mammalian cells, m^6^A deposition is primarily catalyzed by the METTL3–METTL14 methyltransferase complex.^4^ METTL3 is an *S*-adenosyl-l-methionine (SAM)-dependent methyltransferase that targets adenosine within the 5′-DRACH-3′ (D = A/G/U, R = A/G, H = A/C/U) consensus motif.^5–10^ METTL14 is catalytically inactive, yet it is essential for heterodimer stability, substrate recognition and catalytic activity.^8,9^ METTL3, as the catalytic active subunit, is essential for METTL14 stability in cells,^9^ and knockout of either subunit in mouse embryonic stem cells results in complete loss of m^6^A.^11^

METTL3–METTL14 exerts its cellular functions within a larger m^6^A writer machinery,^12,13^ including: WTAP, which promotes nuclear speckle localization and stabilizes the writer complex;^14^ VIRMA, which coordinates region-selective m^6^A deposition;^15^ RBM15 and RBM15B, which help recruit the machinery to selected RNA targets;^16^ and ZC3H13, which together with HAKAI, stabilizes the assembly and reinforces nuclear retention.^17,18^ Substrate specificity *in vivo* therefore emerges from the interplay between the catalytic core and these auxiliary modules.

The biological implications of the m^6^A modification influence several cellular processes: elevated expression or altered activity of METTL3-METTL14 has been reported in multiple hematological and solid tumors, where it supports survival and proliferation and blocks differentiation through m^6^A deposition on oncogenic transcripts.^12,13^ In acute myeloid leukemia (AML), METTL3 and METTL14 are crucial for leukemic cell growth both *in vitro* and *in vivo*: their depletion or inhibition induces cell cycle arrest, differentiation, and apoptosis, while impairing hematopoietic stem/progenitor cell proliferation.^19–22^ Similarly, in breast cancer, increased METTL3 activity correlates with tumor growth, metastasis, and chemotherapy resistance.^23–28^

Collectively, these findings identify METTL3–METTL14 as an attractive therapeutic target, with promising results in AML models.^12^ Nevertheless, evidence for therapeutic efficacy in solid tumors remains limited to selected preclinical settings, and most efforts have focused on active-site inhibition.^12,13,21,28^ This motivates the development of orthogonal strategies targeting the METTL3–METTL14 protein–protein interaction (PPI). Destabilizing this interface may suppress catalytic activity while disrupting the heterodimeric assembly required for RNA recruitment. Peptide-based METTL3–METTL14 PPI modulators have recently been reported,^29,30^ yet their biochemical validation has only focused on binding and cellular phenotypes, while a quantitative assessment of direct catalytic inhibition remains limited. Furthermore, the relationship between interface engagement, complex destabilization, and altered RNA binding, including its impact on RNA-binding stoichiometry, remains elusive.

This question reflects a broader challenge in RNA biology: RNA–protein interactions are central to RNA processing, nuclear export, translation, turnover, and ribonucleoprotein assembly, yet they are often mediated by dynamic, multivalent, and composite interaction surfaces that are difficult to resolve mechanistically.^31,32^ They are commonly studied through complementary approaches, including cross-linking/sequencing methods that map transcriptome-wide contacts, structural biology that defines individual complexes, and biophysical assays that quantify binding in solution.^33,34^ However, heterogeneous RNA–protein assemblies can populate multiple stoichiometries and ligand-sensitive states that ensemble measurements may not fully resolve. Methods capable of preserving intact complexes while separating coexisting binding states are therefore particularly valuable for defining how RNA recognition is organized and perturbed.

This is especially relevant for METTL3–METTL14, whose molecular basis of RNA engagement remains only partially understood. No structure of the complex bound to RNA is currently available, and accurate modeling of RNA–protein interactions is difficult because of the intrinsic flexibility, charge distribution, and conformational plasticity of RNA.^35^ Structurally, METTL3-METTL14 methyltransferase domains form a butterfly shaped heterodimer with a positively charged groove at the interface, well suited for RNA binding.^7–9^ Crucially, METTL14 contains a C-terminal region enriched in arginine–glycine–glycine (RGG) motifs. Although absent from current structures, this region is essential in solution: its deletion markedly reduces RNA binding and RNA methylation activity, defining it as an indispensable auxiliary RNA-contact surface that complements the catalytic groove.^10,36–38^ Accordingly, arginine methylation on METTL14 RGG motif modulates substrate engagement and catalytic output,^38^ and the same region is required for high-affinity recognition of structured RNAs.^10,39,40^ Consistent with a broader role in nucleic-acid engagement, METTL3–METTL14 has been reported to methylate DNA substrates *in vitro*,^40–42^ although this activity is restrained by the regulatory proteins WTAP and VIRMA.^37^

Together, these observations indicate that substrate recognition by METTL3–METTL14 is more versatile than previously appreciated and remains incompletely defined at the molecular level. Current evidence supports a model in which METTL3–METTL14 acts as a multivalent RNA-binding platform: the catalytic pocket engages the DRACH motif, while peripheral regions, such as the METTL14 RGG tail, stabilize additional RNA contacts. Whether the heterodimer binds one or multiple RNAs simultaneously remains unclear, limiting both our understanding of substrate recognition and the design of strategies for a comprehensive therapeutic exploitation.

To dissect this machinery, we focused on the METTL3–METTL14 heterodimer, the minimal catalytic and RNA-engaging unit. This system allows the intrinsic RNA-binding properties of METTL3–METTL14, and the effects of targeting its interface, to be examined without the confounding contribution of accessory subunits. Building on this, we established a workflow that directly links interface targeting to RNA engagement. We optimized native mass spectrometry (MS) for the analysis of the intact METTL3-METTL14 complex and for quantifying its interactions with distinct RNA substrates. Native MS is uniquely suited to preserve noncovalent assemblies and resolve coexisting oligomeric states within heterogeneous populations, enabling clear readouts of interaction stoichiometries and estimation of binding affinities.^43–46^ Beyond protein-only assemblies, native MS has become an established approach for studying nucleic acids and their complexes, enabling characterization of duplexes and G-quadruplexes, resolution of stoichiometry- and site-specific ligand binding to structured RNAs such as riboswitch aptamers, and dissection of RNA–protein interactions.^47–51^

Using this platform, we measured METTL3–METTL14 interactions across diverse RNA architectures and integrated these observations with AI-based molecular modeling and molecular dynamics (MD) simulations to interrogate protein–RNA interactions at the molecular level. We then leveraged structural information to design peptides that mimic METTL14 epitopes contacting METTL3, with the goal of destabilizing the heterodimer and indirectly remodeling RNA recruitment. Integrating native MS with biochemical assays, we quantified peptide binding and complex destabilization and directly measured the resulting changes in RNA engagement. Finally, we bridged biophysical and cellular analyses by showing that METTL3–METTL14 interface targeting translates into impaired cancer cell viability and remodeling of global m^6^A levels and m^6^A-associated regulatory factors in AML and breast cancer models.

Our findings uncover fundamental features of RNA engagement by METTL3–METTL14, revealing how heterodimer architecture enables multivalent substrate recognition, and pave the way for the development of interface-targeting chemical tools with the ability to disrupt RNA binding and inhibit the methyltransferase activity.

## Results

### Native MS Reveals Multivalent RNA Binding by METTL3–METTL14

We first expressed and purified the full METTL3-METTL14 complex and optimized native MS conditions to monitor the intact assembly in both apo and ligand-bound states (Figure 1A). Even after extensive buffer exchange into 200 mM ammonium acetate (pH 7.5), persistent salt adducts were observed. This is consistent with a strong propensity of the complex to retain solution ions under native conditions, in line with the nucleic-acid-binding nature of METTL3–METTL14, which contains an electropositive RNA-binding groove and a basic METTL14 RGG-rich region. Persistent adduct heterogeneity is indeed commonly observed in native MS analyses of highly charged nucleic-acid-associated assemblies.^50,52,53^ Following optimization of instrumental parameters, native MS revealed the presence of charge states consistent with a ∼119 kDa species corresponding to the intact METTL3–METTL14 heterodimer as the dominant population, together with a minor fraction of free METTL14 and only trace signals attributable to METTL3 (Figure 1B). Having obtained well-resolved spectra for the apo state of METTL3-METTL14, we next explored the substrate-binding properties of the complex with different RNA substrates.

**Figure 1:**
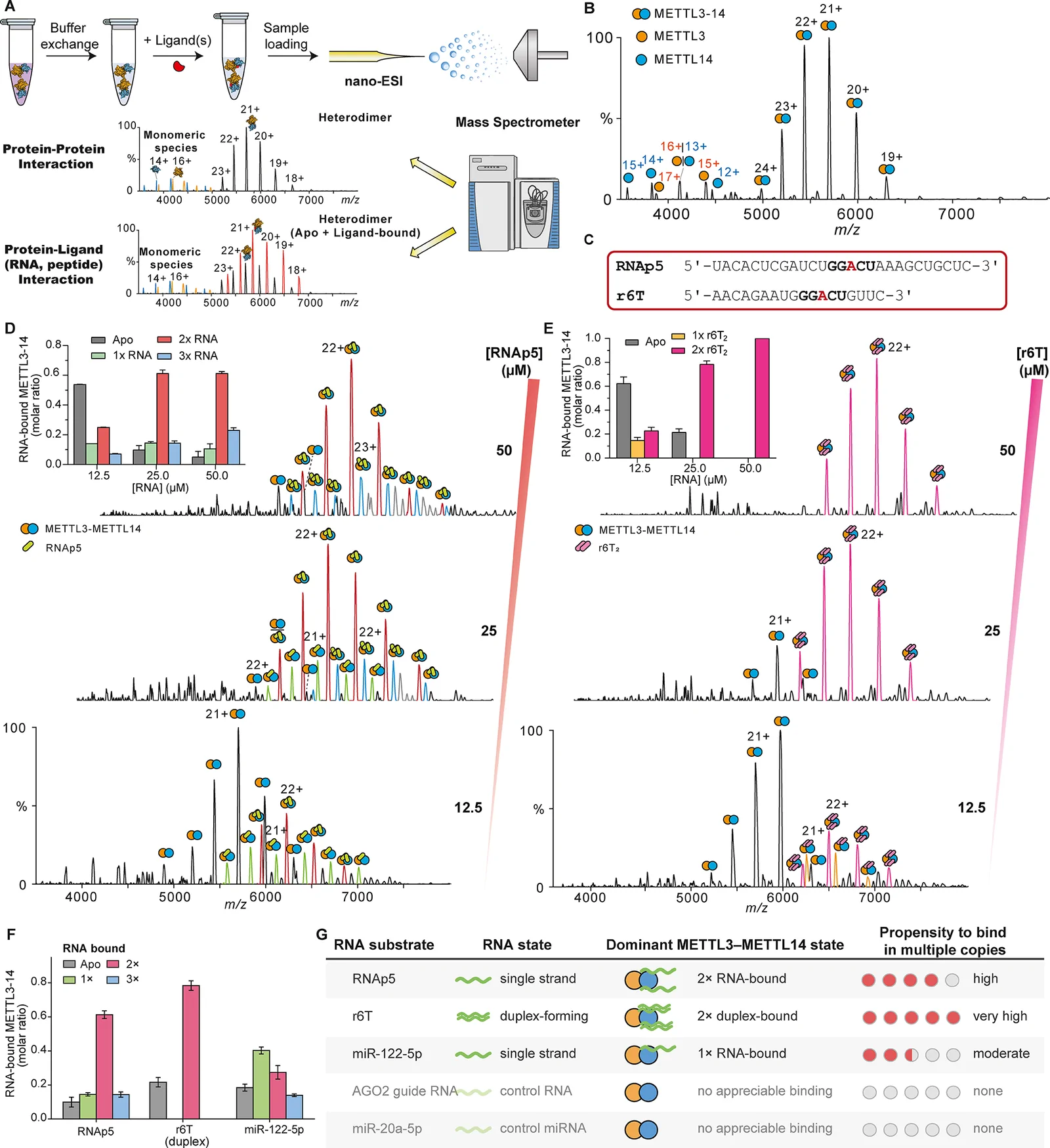
Identification of multivalent and cooperative RNA binding by METTL3-METTL14 by native MS. (A) Schematic overview of the workflow employed for the native MS analysis of METTL3-METTL14 and its interactions. (B) Native mass spectrum of apo METTL3–METTL14 (2.5 μM) in 200 mM ammonium acetate (pH 7.5). (C) Sequences of the oligonucleotides used as substrates. (D, E) Native mass spectra of METTL3-METTL14 (2.5 μM) in the presence of increasing concentrations of RNAp5 (D) or r6T (E). Charge state series corresponding to RNA-bound complexes appear in a concentration-dependent manner. Bar charts report the relative abundances of METTL3–METTL14 bound to one, two, or three RNA molecules (RNAp5) or to one or two r6T duplex units (r6T_2_), as determined from the assigned charge-state series. Peaks corresponding to complexes bound to more than three RNA molecules, detected mainly at the highest RNA concentration, are annotated in gray. The quantification shows a consistent shift toward the two-RNA–bound species at the tested concentrations. (F) Distribution of RNA-bound states of METTL3–METTL14 (2.5 μM) for RNAp5, r6T, and miR-122–5p at 25 μM RNA. Bars show the molar ratio of apo, one-, two-, and three-RNA-bound complex; for r6T (a duplex under these conditions), bound species correspond to duplex units. (G) Schematic summary of the dominant native MS states across all RNAs tested, including nonsubstrate controls (AGO2 guide RNA, miR-20a-5p), which showed no appreciable binding. Filled circles indicate the tendency of each RNA to bind METTL3–METTL14 in more than one copy, as shown in panels (D–F) and in the Supporting Information. The summary reflects relative stoichiometry rather than absolute affinity. Data are expressed as mean ± standard deviation (SD) of three biological replicates (*n* = 3).

To probe substrate binding, we selected RNA probe 5 (RNAp5) as a model substrate, a 27nt oligonucleotide (Figure 1C) designed according to known METTL3–METTL14 substrates^4^ and widely used in biochemical methylation assays.^7,9,21,54–58^ We titrated RNAp5 into METTL3–METTL14 (2.5 μM) and monitored complex formation by native MS. Upon addition of increasing RNA concentrations (12.5, 25, and 50 μM), we observed new adduct peak series corresponding to RNA-bound heterodimer that increased in a concentration-dependent manner, together with higher-occupancy RNA-bound species (Figure 1D). Even at the lowest RNA concentration, the spectra revealed multiple RNA-bound states of the complex, and binding of two RNA molecules was more prevalent than the one-RNA-bound form. This effect became especially pronounced at 25 and 50 μM RNA, where the two-RNA-bound METTL3-METTL14 dominated (Figure 1D). The concentration-dependent enrichment and saturation of the two-RNA-bound state are distinctive of specific binding. States up to three bound RNAs were quantified, being well resolved and supported by enough charge states for reliable integration, whereas higher-order species remained minor, which appeared only at high RNA excess, were annotated but not quantified. To rule out the possibility that RNAp5 self-associates into a duplex under our experimental conditions, we analyzed the RNA alone at the same concentrations through native MS (Figure S1A). Under these conditions RNAp5 remains predominantly monomeric, indicating that the higher-order species do not arise from binding of a preformed RNA duplex and are more consistent with binding to individual RNA molecules.

To further investigate the binding features of METTL3-METTL14 with different RNAs, we analyzed r6T, a 18nt oligonucleotide (Figure 1C) previously used as a model substrate in METTL3–METTL14 studies.^10,40,41^ This RNA has been proposed to self-associate *via* partial complementarity, yielding a duplex-like assembly,^10^ but its solution-state oligomeric form has not been directly established. We first analyzed r6T alone by native MS at 12.5, 25, and 50 μM (Figure S1B). Under these conditions, r6T was detected predominantly as a duplex, indicating that self-association is strongly favored at the concentrations used for binding experiments. We then titrated r6T into METTL3–METTL14 and monitored complex formation by native MS (Figure 1E). Strikingly, the RNA-bound population was dominated by a species corresponding to binding of two r6T duplexes, which became essentially exclusive at 25 and 50 μM RNA, with only a minor contribution from a single-duplex-bound complex at 12.5 μM (Figure 1E). Consistent with r6T existing as a duplex in solution, the observed mass shifts correspond to association of the heterodimer with two duplex units (*i.e.*, four r6T strands), rather than binding of four independent monomers. Taken together, these data indicate that METTL3–METTL14 interacts with r6T in its duplex form, with a clear preference for the two-duplex-bound state. Thus, far, we observed that METTL3–METTL14 interacts with more than one RNA molecule simultaneously, consistent with multivalent RNA binding. Notably, for RNAp5 and for r6T (in its duplex form), we consistently observed a concentration-dependent enrichment of the two-RNA–bound state, suggesting that binding of the first RNA molecule increases the affinity of the protein for the second one, a characteristic feature of cooperative binding.^43,59–62^

We next examined miR-122–5p (Figure S2A), a 22nt miRNA acting as AGO2 guide RNA^63^ and reported to undergo METTL3–METTL14-dependent methylation in a cell-specific manner, a modification associated with reduced AGO2 loading and impaired intracellular activity.^64^ We first established by native MS that miR-122–5p remains predominantly single-stranded at the concentrations tested and does not self-associate into duplexes (Figure S1C). We then titrated miR-122–5p into METTL3–METTL14 and monitored complex formation by native MS at the same concentrations (Figure S2B). As before, the spectra revealed multiple RNA-bound species, indicating that the heterodimer can engage more than one miR-122–5p molecule. However, in contrast to RNAp5 and r6T, the one-RNA-bound complex predominated throughout the titration, whereas the two-RNA–bound state increased only at the highest RNA concentration (Figure S2B). Overall, these data show that METTL3–METTL14 can bind multiple miR-122–5p copies, but with a clear bias toward lower-occupancy binding relative to the other RNAs examined. As a specificity control, we tested two small RNAs that are established human AGO2 guide strands, including a synthetic AGO2 guide RNA^65,66^ and miR-20a-5p (Figure S3A).^63^ Unlike miR-122–5p, these RNAs have not been reported as METTL3–METTL14 substrates. At 25 μM, native MS did not detect appreciable binding to METTL3–METTL14 for either RNA (Figure S3B,C), underscoring the specificity of the interactions observed for miR-122–5p and the other RNAs tested.

Taken together, these data suggest that the intact METTL3–METTL14 heterodimer engages more than one RNA molecule simultaneously, but that the extent and stoichiometry of this multivalent binding depend on the RNA. RNAp5 and r6T (in its duplex form) showed a concentration-dependent enrichment of higher-occupancy complexes consistent with coupled binding events and positive cooperativity (Figure 1C,D), whereas miR-122–5p remained dominated by the one-RNA–bound state across the titration (Figure S2), indicating weaker cooperative engagement (Figure 1F). The reduced higher-order binding of miR-122–5p may reflect a less favorable architecture and sequence context for the auxiliary contacts needed to stabilize cooperative higher-order binding. Finally, the lack of binding to nonsubstrate AGO2 guide RNAs underscores the specificity of these interactions. The properties of each RNA, including sequence, structure, and self-association state, govern not only whether the heterodimer binds, but also the stoichiometry and cooperativity of the resulting complexes (Figure 1G).

### Molecular Modeling of METTL3-METTL14 Interactions with Its RNA Substrates

Native MS established that METTL3-METTL14 engages multiple RNA molecules with substrate-dependent cooperativity but could not reveal the structural basis of this behavior. To address this, we employed molecular modeling to predict how METTL3-METTL14 engages its RNA substrates. Recent advances in generative AI-based modeling have made these systems more tractable, enabling more reliable prediction of RNA-protein assemblies.^67^ We used the Chai-1 package,^68^ providing the sequences of METTL3, METTL14, and two copies of the RNAp5 substrate together with SAM as input. The METTL3-METTL14 core was predicted with high confidence in both the presence and absence of the METTL14 RGG region, with root-mean-square deviations (RMSDs) of ∼0.3 Å relative to the experimentally solved structure (PDB 5IL1).^8^ Notably, inclusion of the METTL14 RGG region yielded a binding mode in which two RNA molecules were accommodated as separate strands on METTL3-METTL14 (Figure 2A), supporting multivalent substrate engagement. By contrast, omission of this motif led RNAp5 to bind as a duplex (Figure 2B), a configuration not supported by our experimental data (Figure S1A).

**Figure 2:**
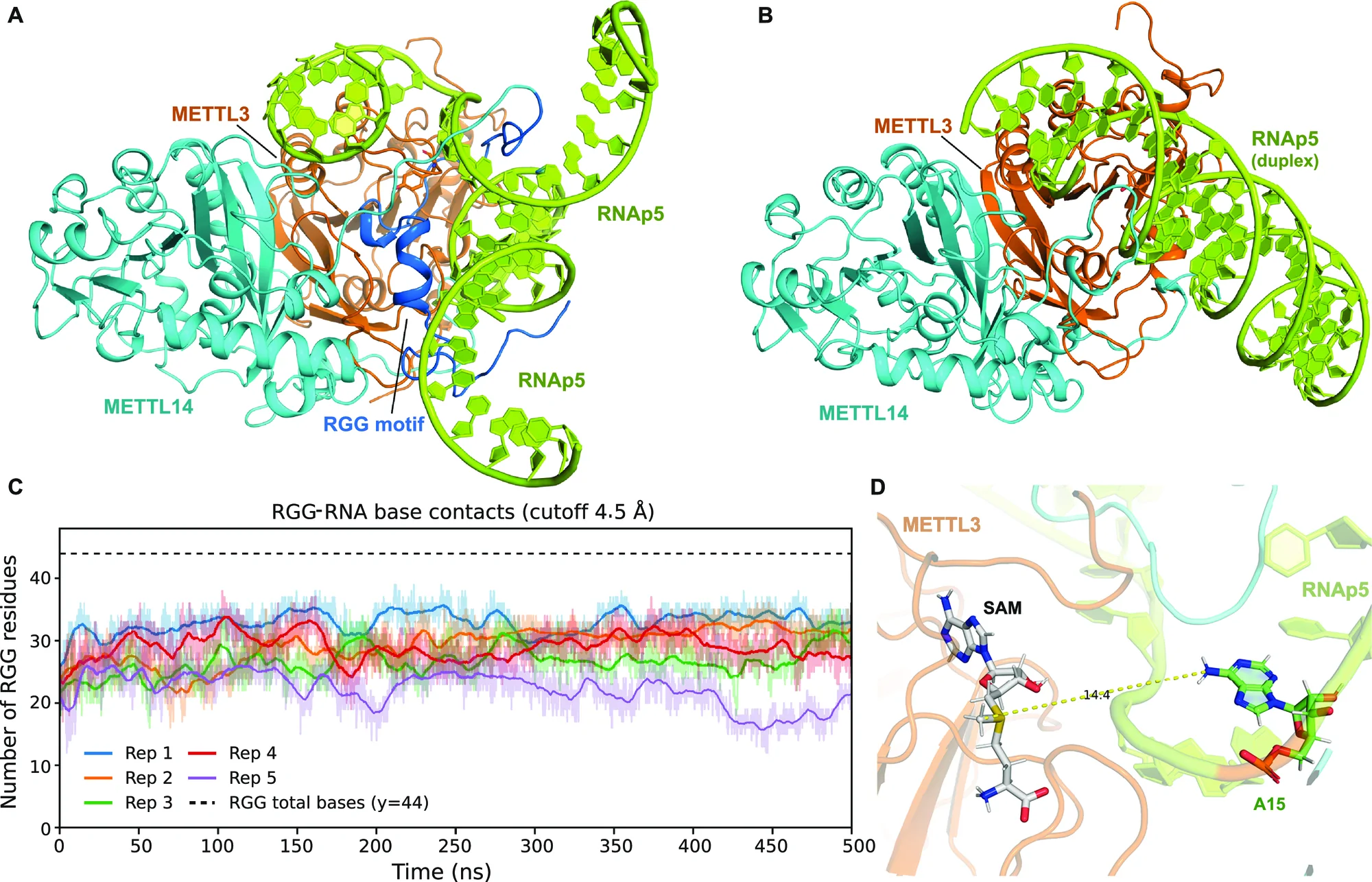
Molecular modeling and MD simulations of multivalent RNA binding by METTL3–METTL14. (A) Structural model of the METTL3-METTL14 complex predicted by Chai-1, shown with METTL3 in orange, METTL14 in cyan, the RGG motif in blue, and bound RNAp5 in lime. (B) Equivalent model generated in the absence of the METTL14 RGG motif. (C) Number of contacts formed between the RGG motif and RNAp5 molecules throughout the MD simulations. (D) Representative MD snapshot showing a 14.4 Å distance between the N6 atom of the target adenosine and the SAM methyl group.

These distinct binding modes were maintained during 500 ns MD simulations, with the RNA remaining relatively stable in both cases but showing greater overall stability when the RGG motif was present (Figure S4A,B). In the RGG-containing complex, one RNA occupied the groove at the METTL3–METTL14 interface, whereas the second was positioned closer to METTL3, near the SAM pocket. Both RNAs contacted the RGG region through their central portions, while the free 3′ and 5′ ends remained the most flexible segments (Supporting Video 1). This organization suggests that the RGG motif helps stabilize a two-RNA assembly while limiting inter-RNA duplex formation, with approximately 65% of the residues within the RGG region forming RNA contacts during the simulations (Figure 2C). Together, these observations support a model in which the RGG region stabilizes two separate RNA strands and suppresses extensive interstrand pairing, reconciling the predicted assembly with the predominantly monomeric behavior of RNAp5 observed by native MS (Figure S1A).

Clustering analysis of the RGG-containing trajectories identified two closely related conformational states that preserved the overall RNA-binding mode and differed mainly in the conformation of the flexible RNA tail (Figure S5). Consistently, RGG-containing complexes formed fewer interstrand hydrogen bonds than the RGG-lacking complex (5–15 *vs* 20–25), in accordance with the more extensively duplexed state predicted only in the truncated model (Figure S6). This behavior is coherent with the stabilization of RNA duplexes by Watson–Crick pairing and base stacking,^69^ whereas the RGG region provides competing multivalent RNA–protein contacts that stabilize the strands separately.^70,71^

Across all MD simulations in the presence of the RGG motif, the distance between the N6 atom of the target adenosine (A15) and the SAM methyl group was approximately 15–20 Å (Figures 2D and S7), with two replicates sampling shorter distances of ∼8–10 Å (Figures S7 and S8). Omission of the RGG motif increased this distance by ∼5–10 Å, placing the target N6 farther from the SAM methyl group and making this arrangement less compatible with direct methyl transfer (Figure S9). These data support interpretation of the RGG-containing assembly as a pre-engaged state in which the target adenosine is positioned more favorably relative to SAM.

We next asked whether this RGG-dependent stabilization also extended to miR-122–5p, an RNA substrate showing distinct cooperative binding behavior by native MS. We modeled METTL3-METTL14 bound to two miR-122–5p molecules in the presence of the full-length RGG region. The overall binding mode remained stable during MD simulations (Figure S10A) and was broadly similar to that observed for RNAp5 (Figure S10B,C). Despite this overall similarity, the two miR-122–5p strands behaved more asymmetrically than the RNAp5 strands. The strand closer to the SAM pocket retained the target adenosine in a solvent-accessible conformation, with the RGG region limiting intrastrand base pairing (Figure S10D). Conversely, the distal strand showed greater intrastrand pairing and partial occlusion of the same motif (Figure S10E). Direct comparison of the 2× RNA complexes revealed a conserved interaction surface at the METTL3–METTL14 core but substrate-specific engagement of the RGG region (Figure S11). RNAp5 contacted predominantly the C-terminal portion of the RGG region, whereas miR-122–5p engaged its central portion, indicating that the RGG region behaves as a flexible, arginine-rich clamp rather than as a fixed sequence-recognition element.

Base-level contact analysis confirmed preferential RGG engagement of the miR-122–5p strand closer to the catalytic site, whereas the RNAp5 complex showed a more balanced distribution of RGG contacts between the two strands (Figure S12). This difference parallels the preferential enrichment of the 2× RNA-bound state for RNAp5 and the predominance of the 1× RNA-bound state for miR-122–5p observed by native MS.

Congruent with the RNAp5 simulations, omission of the RGG region from the miR-122–5p system increased RNA conformational variability and produced a larger and more variable distance between the target adenosine and the SAM methyl group (Figures S13 and S14), supporting a role for the RGG region in stabilizing the target adenosine near the catalytic site.

Comparison of the 1× RNA and 2× RNA simulations for RNAp5 and miR-122–5p clarified how a second RNA is accommodated. In all 1× RNA models the RNA occupies the groove between METTL3 and METTL14 (Figure S15). RMSD and root-mean-square fluctuation (RMSF) analyses showed that the RGG region provides an additional anchoring surface that restricts RNA mobility for the extended RNAp5, whereas the flexibility of the more compact miR-122–5p was largely unchanged (Figures S16–S17). The contribution of the RGG region depended on occupancy. In the 2× RNA assemblies, it stabilized two separate strands while limiting interstrand duplex formation (Figures 2, S6, S10 and S11). In the 1× RNA complexes, by contrast, it shifted the balance from RNA self-folding toward RNA–protein contacts (Figures S15–S17). Binding of the first RNA thus partially organizes the flexible RGG region into a composite surface spanning the groove and the RGG motif, able to accommodate a second RNA without displacing the first, providing a plausible structural rationale for the multivalent RNA binding observed by native MS.

Together, these findings indicate that the METTL14 RGG region acts as a flexible, substrate-adaptive clamp that extends the METTL3–METTL14 groove into a composite surface for multivalent RNA engagement. Its effects depend on both RNA identity and occupancy, with more balanced stabilization of the two RNAp5 strands but more asymmetric engagement of miR-122–5p, consistent with the differences in cooperative binding observed by native MS.

### Identification METTL3–METTL14 Peptide Disruptors Guided by Native MS

Having established that METTL3-METTL14 can engage multiple RNA molecules simultaneously, we next asked whether this multivalent RNA recognition could be perturbed by disrupting the PPIs that stabilize the complex. We therefore designed a set of METTL14-derived peptides to mimic regions contacting METTL3 and thereby destabilize the heterodimer and/or impair RNA recruitment. The METTL3-facing surface of METTL14 spans Arg254-Glu317 and contributes approximately half of the ∼2500 Å^2^ heterodimer interface, accounting for most of the contacts between the protomers (Figure S18).^7–9^ Using a “frameshift” strategy, we generated peptides **1–3** and evaluated binding to METTL3-METTL14 by native MS (Figure S19). Among the extended segments, only peptide **1** formed a stable complex with the heterodimer with an apparent dissociation constant (*K*_D_) of 5.2 μM. Peptide **1** was designed to cover the N-terminal β-strand (Arg254-Lys264) followed by a loop (Asn265-Thr284), identifying this region as the only METTL14 segment able to bind METTL3-METTL14 with low-micromolar affinity. Dissection of peptide **1** into its constituent segments (Figure S19B,C) showed that the β-strand fragment **4**, a buried element in the native protein, bound weakly (*K*_D_ = 28.7 μM). In contrast, peptide **5**, covering a loop occupying a groove in METTL3, retained low-micromolar affinity (*K*_D_ = 7.5 μM). All fragments derived from peptides **2** or **3** (**6** – **10**) showed minimal or no binding (Table 1).

**Table 1:** Apparent *K*_D_ Values for the Interaction between Each Peptide and the METTL3-METTL14 Complex Determined by Native MS^a^

Peptide	Sequence	*K*_D_ (μM)
**1**	Ac-R ^254^ RCEDICWIKTNKNNPGK**TKTLDPKAVFQRT** ^**284**^-CONH_2_	5.2 ± 1.1
**2**	Ac-K ^285^ EHCLMGIKGTVKRSTDGDFIHANVDIDLIITE ^317^-CONH_2_	N.D.^b^
**3**	Ac-**T** ^**272**^ **KTLDPKAVFQRT** KEHCLMGIKGTVKRSTDGDFIH^306^-CONH_2_	N.D.
**4**	Ac-R ^254^ RCEDICWIKT ^264^-CONH_2_	28.7 ± 6.4
**5**	Ac–N^265^KNNPGK**TKTLDPKAVFQRT** ^**284**^-CONH_2_	7.5 ± 1.1
**6**	Ac-K ^285^ EHCLMGIKGTVKRSTDGDFIH^306^-CONH_2_	N.D.
**7**	Ac-G^294^TVKRSTDGDFIHANVDIDLIITE ^317^-CONH_2_	N.D.
**8**	Ac–K^285^EHCLMGIK^293^–CONH_2_	20.1 ± 4.3
**9**	Ac–H^306^ANVDIDLIITE ^317^-CONH_2_	N.D.
**10**	Ac-**T** ^**272**^ **KTLDPKAVFQRT** KEHCLMGIK ^293^-CONH_2_	18.7 ± 2.9
**11**	Ac-**T** ^**272**^ **KTLDPKAVFQRT** ^**284**^-CONH_2_	6.3 ± 1.1

a Values are reported as mean ± SD of three biological replicates (n = 3). Underscored sequences indicate β-strands; sequences in bold indicate the **T** ^**2** **72**^ **-T** ^**284**^ **Loop**.

b Less than 25% binding at 40 μM.

Having identified peptide **1** and its loop segment as the minimal region binding METTL3 with low-micromolar affinity, we next sought to define the smallest fragment retaining this activity, as the basis for a compact PPI disruptor. We therefore focused on the β4-β5 loop (Thr272-Thr284), which establishes both polar and hydrophobic contacts with METTL3 and contains a defined turn plus a C-terminal Val280-Phe281 pair deeply embedded in the METTL3 surface (Figure S20A). The peptide corresponding to this loop (**11**) preserved low-micromolar affinity (*K*_D_ = 6.3 μM; Figures S20–S22). Shortened analogues further mapped the minimal recognition motif. Peptide **12**, bearing a Lys227 → Arg substitution, strengthened binding (*K*_D_ = 3.0 μM), whereas peptide **13**, carrying a d-Pro at the loop turn, showed lower affinity (*K*_D_ = 8.1 μM). Further truncation identified **14** as the smallest construct retaining low-micromolar affinity (*K*_D_ = 2.7 μM; Figure 3A,B), defining the minimal determinants for METTL3 engagement (Table 2). We then measured IC_50_ values using RNAp5 as substrate (Table 2). Peptide **11** inhibited weakly (IC_50_ = 83 μM), whereas **12** was more active (IC_50_ = 41 μM), and **14** was markedly more potent (IC_50_ = 9.0 μM), demonstrating that Lys → Arg substitution and loop compaction enhance inhibition of METTL3-METTL14. By contrast, **13** was less potent (IC_50_ = 144 μM), consistent with disruption of the turn geometry required for productive interface distortion.

**Figure 3:**
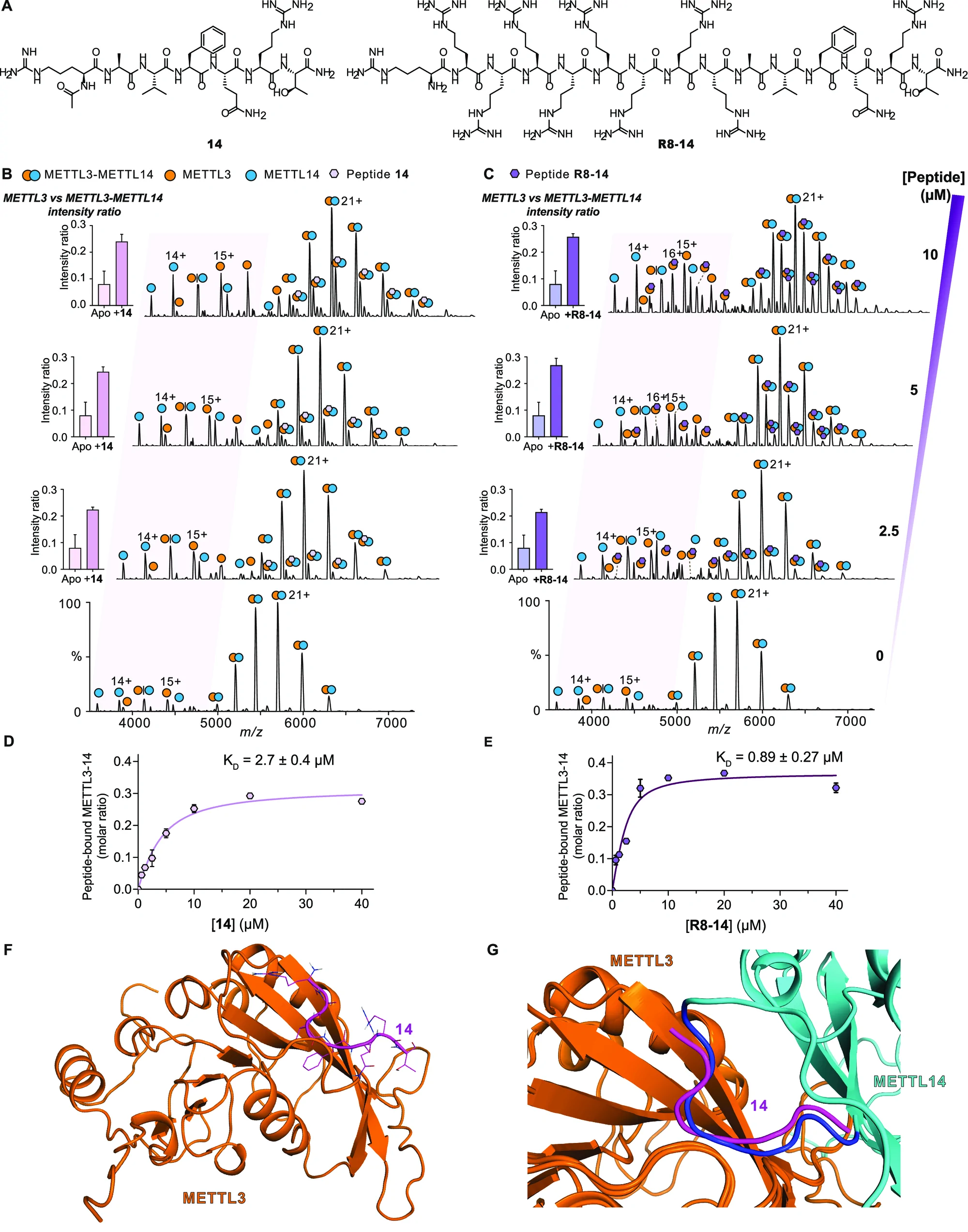
Identification of METTL14-derived peptides that bind METTL3 and destabilize the METTL3–METTL14 heterodimer. (A) Structures of peptides **14** and **R8–14**. (B, C) Native mass spectra of METTL3–METTL14 (2.5 μM) recorded in the presence of increasing concentrations of **14** (B) or **R8–14** (C). Charge states corresponding to peptide-bound complex become apparent and their intensity increases in a concentration-dependent manner, accompanied by an increase in the intensity of peaks assigned to free METTL3 and METTL14. Bar charts report the ratio between the total peak intensities of free METTL3 and METTL3–METTL14 complex, calculated from the assigned charge-state series. This ratio increases upon peptide addition relative to the apo complex, indicating peptide-induced release of free METTL3 and destabilization of the heterodimer. (D, E) Dose–response curves showing concentration-dependent binding of METTL3–METTL14 to peptide **14** or **R8–14**. The molar ratio of peptide-bound complex, including species bound to more than one peptide, was quantified across peptide concentrations and fitted to derive apparent *K*_D_ values for the first binding event. Data are reported as mean ± SD from three independent biological replicates (*n* = 3). (F) Modeling peptide binding to METTL3–METTL14 showing the best pose following a global docking approach, with METTL3 in orange and the peptide in pink. (G) Overlay of the best docking pose (pink) in relation to the METTL3-METTL14 complex, with the originator METTL14 loop (dark blue).

**Table 2:** Apparent *K*_D_ Values for the Interaction of Each Peptide with the METTL3–METTL14 Complex Determined by Native MS and Relative Inhibition of METTL3–METTL14 Catalytic Activity Measured Using RNA Probe 5 as the Substrate^a^

Peptide	Sequence	*K*_D_ (μM)	IC_50_ (μM)
**11**	Ac-T^272^KTLDPKAVFQRT^284^-CONH_2_	6.3 ± 1.1	83 ± 14
**12**	Ac-T^272^KTLDPRAVFQRT^284^-CONH_2_	3.0 ± 0.6	41 ± 6
**R8–12**	NH_2_-RRRRRRRRT^272^KTLDPRAVFQRT^284^-CONH_2_	3.8 ± 0.6	38 ± 3
**13**	Ac-T^272^KTLDpKAVFQRT^284^-CONH_2_	8.1 ± 1.5	144 ± 6
**14**	Ac-RA^279^VFQRT^284^-CONH_2_	2.7 ± 0.4	9.0 ± 0.1
**R8–14**	NH_2_-RRRRRRRRRA^279^VFQRT^284^-CONH_2_	0.89 ± 0.27	0.23 ± 0.01
**15**	Ac-[CKTLDPKAVFQRC]-CONH_2_	13.3 ± 1.4	155 ± 13
**16**	Ac-[CKTLDPKAVFQRC]-methylene-CONH_2_	8.9 ± 1.7	7.0 ± 0.5
**17**	Ac-[CKTLDPKAVFQRC]-ethane-CONH_2_	5.9 ± 0.6	2.8 ± 0.22
**18**	Ac-[CKTLDPKAVFQRC]-acetone-CONH_2_	8.0 ± 1.2	5.3 ± 0.62
**19**	Ac-[CKTLDPKAVFQRC]-orthoxylylene-CONH_2_	10.9 ± 1.3	22 ± 1
**20**	Ac-[CKTLDPKAVFQRC]-metaxylylene-CONH_2_	6.4 ± 0.8	2.7 ± 0.2
**21**	Ac-[CKTLDPKAVFQRC]-paraxylylene-CONH_2_	3.2 ± 0.6	0.77 ± 0.01
**22**	[GTKTLDPKAVFE]RT-CONH_2_	12.9 ± 1.4	111 ± 21
**23**	[GabaTKTLDPKAVFE]RT-CONH_2_	9.4 ± 1.0	17 ± 3

a Values are reported as mean ± SD of three biological replicates (n = 3). Modifications from the original sequence are underscored.

To gain structural insight into peptide **14** binding, we performed global docking using GNINA 1.3 ^72^ which consistently predicted binding to METTL3 (Figures 3F, and S23A) in a pose closely overlapping with the corresponding METTL14 loop in the native heterodimer (Figure 3G), indicating that **14** recapitulates a key interfacial binding element. This pose remained stable during MD simulations (Figure S23B) supporting a high confidence predicted pose. Together, these data support a mechanism in which **14** competes with METTL14 for METTL3 binding and destabilizes the METTL3-METTL14 complex.

We next assessed whether appending an octa-arginine cell-penetrating sequence (R8)^73,74^ could modulate the binding and inhibitory properties of peptides **12** and **14**, in light of potential application in cellular contexts. **R8–12** displayed affinity comparable to **12** (*K*_D_ = 3.8 μM) and showed no significant improvement in inhibition (IC_50_ = 38 μM). Differently, peptide **R8–14** was the tightest binder (*K*_D_ = 0.87 μM, Figure 3A,C) and the most potent inhibitor (IC_50_ = 0.23 μM, Table 2), indicating that the R8 tag does not compromise and can further enhance peptide activity.

We also asked whether macrocyclization of the METTL14 loop pharmacophore could improve potency through conformational preorganization, a strategy used to restrain short peptide flexibility and reduce the entropic penalty upon binding.^75,76^ The resulting macrocycles (syntheses Schemes S1–S2) bound METTL3-METTL14 with *K*_D_ values of 3–13 μM (Figures S20–22), yet showed wide differences in potency. Among the peptides obtained through the cross-linking approach known as Chemical Linkage of Peptides onto Scaffolds (CLIPS),^77,78^ the most active were the *meta*- and *para*-xylylene peptides **20** and **21**, with low/submicromolar *K*_D_ and IC_50_ values, whereas the *ortho*-xylylene **19** was less potent (Table 2). Shorter-linker macrocycles **16–18** retained low-micromolar activity, while the disulfide-linked **15** was substantially weaker (Table 2). Finally, lactam cyclization between Thr272 and Gln282 (Scheme S2) yielded **22** (*K*_D_ = 12.9 μM; IC_50_ = 111 μM) and **23** (*K*_D_ = 9.4 μM; IC_50_ = 17 μM), confirming that potency is highly sensitive to the imposed loop constraint despite broadly similar binding affinities (Table 2).

Native MS further indicated partial destabilization of the METTL3–METTL14 heterodimer: for most peptides, we observed a slight increase in the relative abundance of free METTL3 and METTL14 protomers (Figure S21), an effect that became more pronounced for low- to submicromolar inhibitors. This is exemplified by the higher ratio between free METTL3 and full METTL3-METTL14 complex upon addition of peptides **14** and **R8–14** relative to the apo state (Figure 3B,C), indicating peptide-induced destabilization of the heterodimer. Notably, **R8–14** also formed an adduct with free METTL3, but not with METTL14, supporting selective engagement of the METTL3 surface, consistent with the intended METTL14-mimetic mechanism.

Overall, the native MS and inhibition data underscore that binding is required, but not sufficient for functional disruption: several midmicromolar binders display weak inhibition, consistent with nonproductive interaction. In contrast, peptides forming low-micromolar complexes were enriched among the low- to submicromolar inhibitors. Thus, our workflow identified potent destabilizers of the METTL3-METTL14 heterodimer, among which peptide **14** and its R8-conjugated analogue **R8–14**, were selected for further functional studies.

### Peptide-Induced Heterodimer Destabilization Disrupts Multivalent RNA Binding

Having identified peptides that bind and destabilize the heterodimer, we next asked whether this perturbation remodels the multivalent RNA-bound states seen earlier by native MS. Since these peptides act on the interface rather than the catalytic site, linking interface destabilization to impaired RNA engagement would show that complex disruption suppresses substrate recruitment, and hence m^6^A deposition, through a mechanism distinct from active-site inhibition.

We incubated METTL3–METTL14 with RNAp5 and increasing concentrations of peptide **14** or **R8–14**, and monitored the distribution of RNA-bound species by native MS. Both peptides produced a dose-dependent reduction in RNA-bound METTL3–METTL14, with **R8–14** exerting the strongest effect and fully abolishing RNA binding at 5 μM (Figure 4A,B). In parallel, charge states assigned to free METTL3 and METTL14 increased, mirroring the destabilization pattern observed with peptides alone. Disruption of the RNA-bound heterodimer was accompanied by the appearance of charge-state series assigned to METTL14 bound to two copies of RNAp5, consistent with METTL14 (possibly *via* its RGG motif) being the principal RNA-binding subunit. Inspection of individual RNA-bound states revealed that peptide-induced RNA displacement proceeds through preferential loss of the 2× RNA-bound state, whereas the singly bound species remained relatively constant and did not accumulate (Figure 4A,B—insets). This pattern is consistent with disruption of a cooperatively stabilized RNA-bound assembly rather than a simple reduction of RNA affinity. Quantification of total RNA-bound METTL3–METTL14 across peptide concentrations yielded half-maximal RNA displacement values (EC_50_) of 4.4 μM for peptide **14** and 1.8 μM for **R8–14** (Figure 4C,D), consistent with their inhibitory potencies. Together, these data indicate that **14** and **R8–14** not only weaken METTL3–METTL14 interaction but also disrupt the RNA binding.

**Figure 4:**
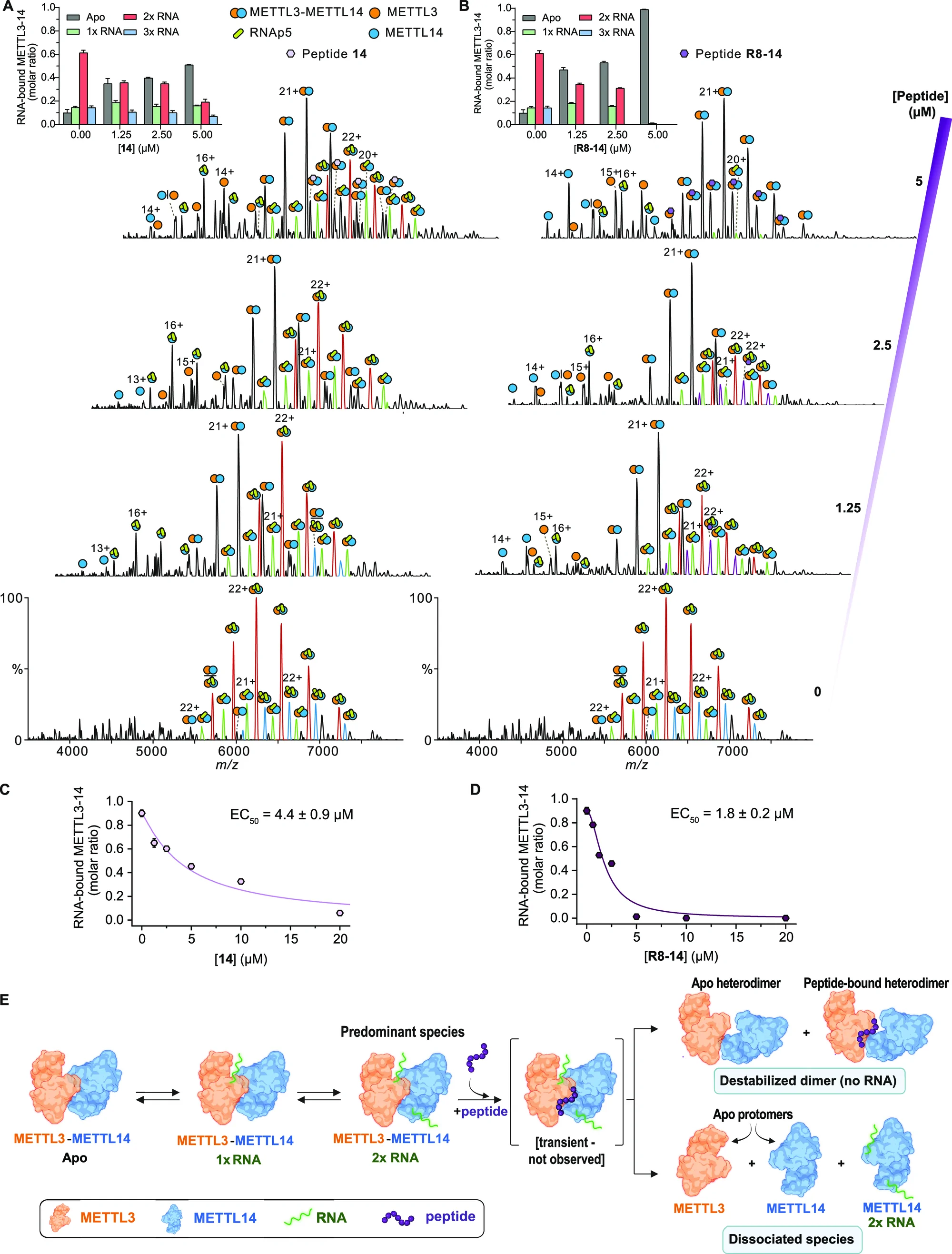
Native MS analysis showing that peptide-induced destabilization of METTL3–METTL14 disrupts multivalent RNA binding. (A, B) Native mass spectra of METTL3–METTL14 (2.5 μM) recorded in the presence of RNAp5 (25 μM) and increasing concentrations of **14** (A) or **R8–14** (B). Charge state series corresponding to RNA-bound complexes decrease in a concentration-dependent manner, accompanied by an increased contribution of charge states assigned to free METTL3 and METTL14. Bar charts report the relative abundances of METTL3–METTL14 bound to one, two, or three RNA molecules as determined from the assigned charge-state series. (C, D) Quantification of METTL3–METTL14 bound to RNAp5 across the tested peptide concentrations and fitting to derive EC_50_ values. The quantification shows a dose-dependent decrease of total RNA bound species at the tested concentrations. Data are expressed as mean ± SD of three biological replicates (*n* = 3). (E) Proposed model of peptide-induced disruption of multivalent RNAp5 binding. The heterodimer populates apo, 1× and 2× RNAp5-bound states, with the 2× RNAp5-bound species predominating; peptide binding at the METTL3-METTL14 interface yields a destabilized dimer devoid of RNA and dissociated species, including METTL14 retaining two RNAp5 molecules. (Created in https://BioRender.com).

We expanded our analysis to the METTL3-mimicking peptide RSM3 and its analogue SM3 lacking the octa-arginine motif,^29^ and benchmarked our METTL14 loop-derived inhibitor by determining their inhibitory potencies, including that of the linear analogue RM3. All three were less potent than **R8–14**: RM3 inhibited with an IC_50_ of 2.7 μM, SM3 with 9.4 μM, and RSM3 with 1.0 μM. Native MS showed that both SM3 and RSM3 reduced the abundance of RNAp5-bound METTL3–METTL14 in a concentration-dependent manner, again with preferential depletion of the 2× RNA-bound population and a concomitant increase in free METTL3 and METTL14 (Figure S28A,B). EC_50_ values were 4.5 μM for SM3 and 3.5 μM for RSM3 (Figure S28C,D), indicating that the METTL3-mimicking scaffold also impairs RNA engagement, albeit less effectively than the METTL14-derived peptides.

We then evaluated the effects of **14**, **R8–14**, SM3, and RSM3 on METTL3–METTL14 binding to r6T and miR-122–5p to determine how peptide-driven destabilization affects engagement of distinct RNA scaffolds. All four peptides reduced binding to both RNAs in a concentration-dependent manner, with **R8–14** and RSM3 producing stronger effects than their non-R8 counterparts and **R8–14** remaining the most potent one (Figures S29 and S30). As seen with RNAp5, the cooperatively bound r6T displayed preferential loss of the 2× RNA-bound population without accumulation of the 1× state, together with a direct increase in apo METTL3–METTL14 (Figure S25).

Collectively, these experiments show that both METTL14- and METTL3-mimicking peptides remodel METTL3–METTL14 binding to multiple RNA substrates. For RNAs showing preferential binding of two RNA molecules, peptide treatment depleted the higher-occupancy states without accumulation of singly bound intermediates, consistent with disruption of a multivalent, cooperatively stabilized RNA-bound assembly (mechanistic model in Figure 4E). These native MS data mechanistically link interface-targeting peptide binding to impaired substrate recruitment by METTL3–METTL14. Among the compounds tested, **R8–14** consistently exerted the strongest effects on both heterodimer integrity and RNA engagement.

### Interface-Targeting Peptides Disrupt METTL3–METTL14 Function and Impair Cancer Cell Viability

Having established a direct biophysical link between peptide binding, heterodimer destabilization, and loss of multivalent RNA engagement, we next asked whether this translates into cellular activity in cancer models dependent on METTL3–METTL14. Selected peptides were evaluated in a panel of cancer cell lines after 24 h incubation, using RSM3 as a reference control. We focused on AML (MOLM-13) and breast cancer (triple-negative MDA-MB-231 and ER^+^ MCF-7), reported to depend on METTL3-driven m^6^A programs,^12,13,19–28^ as well as osteosarcoma (MG-63) and ovarian cancer (SK-OV3), in which aberrant METTL3–METTL14 activity has been linked to malignant phenotypes.^79–87^ Among the tested compounds, **R8–14** displayed the most pronounced activity, with preferential reduction of viability in MOLM-13 and MDA-MB-231 cells (Figures 5A,B, and Table S1). Both **R8–14** and RSM3 showed moderate effects in MCF-7 and minimal or no activity in MG-63 and SK-OV3 cells (Figure S28 and Table S1), indicating selective cytotoxicity.

**Figure 5:**
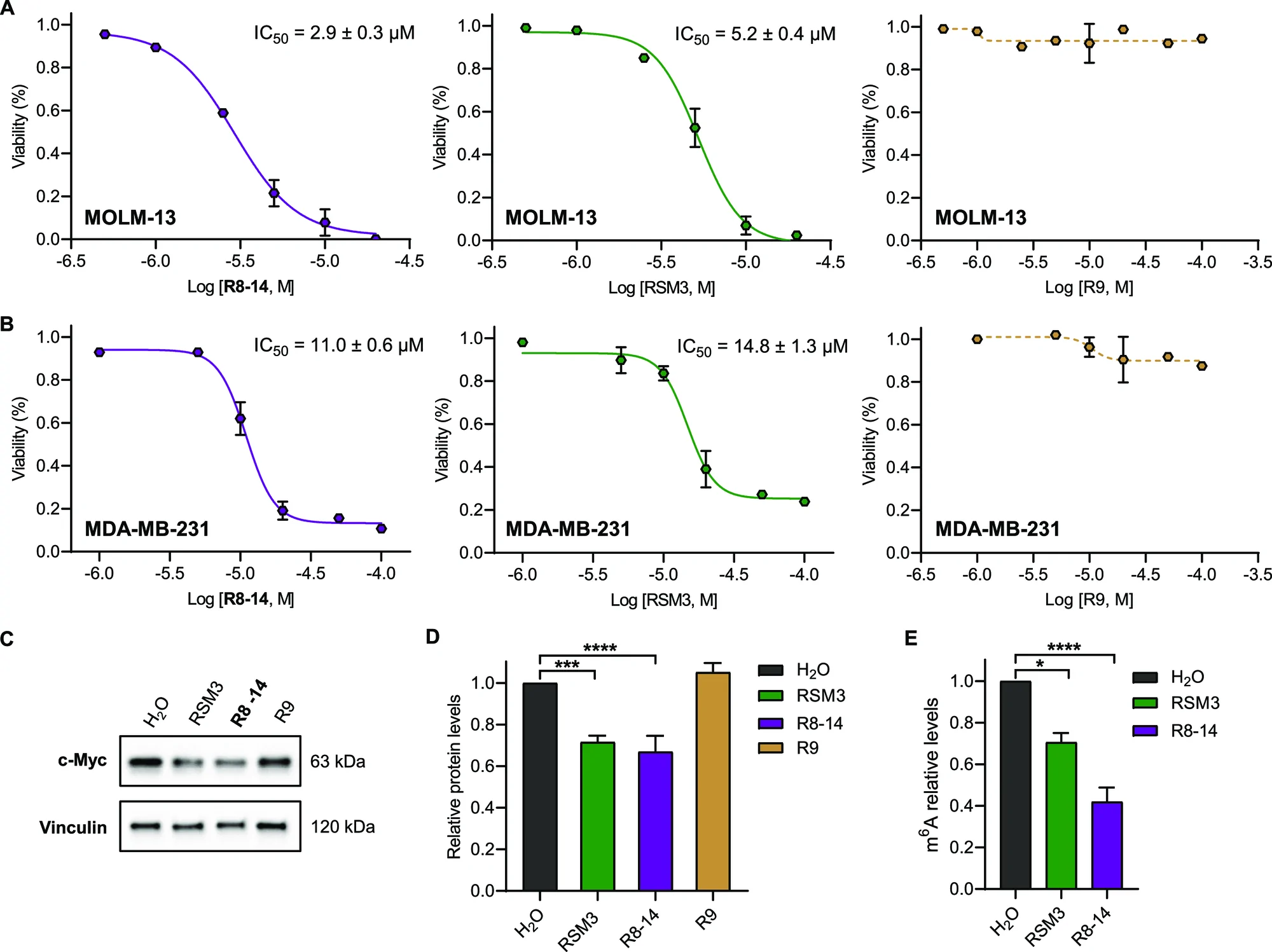
Cellular activity of interface-targeting peptides in AML and breast cancer models. (A, B) Dose–response curves showing the antiproliferative effects of **R8–14**, RSM3, and R9 (from left to right) after 24 h treatment at increasing concentrations in MOLM-13 (A) and MDA-MB-231 (B) cells. Vehicle-treated cells (H_2_O) served as controls. (C) WB analysis of c-Myc levels in MOLM-13 cells following 24 h exposure to RSM3, **R8–14**, or R9 (2.5 μM). Vinculin was used as a loading control. (D) Densitometric quantification of c-Myc levels, expressed as fold change relative to vehicle (H_2_O) after vinculin normalization. (E) Global m^6^A quantification in MOLM-13 cells after 24 h treatment with RSM3 or **R8–14**, expressed as fold change relative to vehicle. All experiments were performed in triplicate (*n* = 3); data are presented as mean ± SD. Statistical significance was assessed by one-way ANOVA *versus* vehicle (*p < 0.05; ***p < 0.001).

To exclude nonspecific cytotoxicity from the polycationic motif, we tested a nona-arginine peptide (R9), which carries the same number of sequential arginine residues as **R8–14**, yet binds METTL3-METTL14 only marginally, with ∼10% of the complex bound even at a large excess (40 μM; Figure S27). R9 did not reduce viability in any cell line at 100 μM (Figures 5A,B, S28 and Table S1), indicating that the arginine tag alone is not cytotoxic. Consistently, **R8–12**, which contains the same R8 tag but inhibits METTL3–METTL14 only weakly *in vitro* (IC_50_ ≈ 38 μM, Table 2), was also inactive across all cell lines (Figure S29 and Table S1).

Peptides lacking the polyarginine tag (**14** and **SM3**) reduced viability in MOLM-13 and MDA-MB-231 at substantially higher concentrations (Figure S29 and Table S1), suggesting that the R8 motif is essential for efficient intracellular delivery and biological activity, and the effects of **R8–14** are linked to a specific activity rather than generalized toxicity. Together, these results confirm that cellular activity requires both intracellular delivery and an effective METTL3–METTL14–disrupting pharmacophore. Finally, neither **R8–14** nor RSM3 affected viability of noncancerous AHH1 B lymphocytes or HME mammary epithelial cells (Figure S30), supporting a degree of cancer selectivity consistent with the dependence of malignant cells on METTL3–METTL14.

Because METTL3-dependent mRNA methylation promotes translation of oncogenic transcripts in AML cells, including c-Myc,^20^ we examined c-Myc protein levels after peptide treatment. Both **R8–14** and RSM3 (at 2.5 μM) significantly reduced c-Myc levels in MOLM-13 cells after 24 h, whereas R9 had no effect (Figure 5C,D), consistent with specific inhibition of the METTL3–METTL14 complex. We also tested whether peptide treatment altered METTL3 or METTL14 protein abundance since METTL3 was shown to protect METTL14 from STUB1-mediated ubiquitination and proteasomal degradation.^88^ In contrast to previous reports for RSM3 in other cellular contexts,^29^ neither **R8–14** nor RSM3 measurably reduced METTL3 or METTL14 levels in MOLM-13 or MDA-MB-231 cells (Figures S31 and S32), indicating that functional inhibition can occur without degradation of the writer components and that the STUB1-dependent degradation pathway may be context-specific.

Consistent with intracellular target engagement, both **R8–14** and RSM3 increased WTAP protein levels in MOLM-13 cells (Figure S33A), in line with prior observations linking METTL3 perturbation to WTAP upregulation.^89^ Moreover, **R8–14** significantly reduced global m^6^A levels after 24 h in MOLM-13 (Figure 5E) and MDA-MB-231 cells (Figure S33B), with RSM3 showing a weaker but similar trend. Together, these data show that **R8–14** efficiently engages the target in cells, preferentially impairs viability of METTL3-dependent cancer models, suppresses downstream oncogenic signaling, and reduces m^6^A output without nonspecific toxicity or widespread degradation of METTL3-METTL14. The convergence of biochemical, structural, and cellular readouts establishes a direct link between heterodimer destabilization, reduced RNA binding, and functional cellular outcomes, providing clear evidence of target engagement and therapeutic potential for the developed peptide.

## Discussion

In this study, we combined native MS, molecular modeling, synthetic chemistry, biochemical and cellular assays to address two connected questions about METTL3–METTL14: how the machinery engages RNA and how perturbation of its heterodimer interface reshapes RNA binding and catalytic output. Our native MS data reveal that METTL3–METTL14 is not limited to binding a single RNA oligonucleotide. Instead, the intact heterodimer accommodates multiple RNAs simultaneously, and the extent of higher-order binding depends on RNA identity. For RNAp5 and r6T, higher-occupancy states were enriched in a concentration-dependent manner, consistent with coupled binding events and positive cooperativity (Figure 1), whereas miR-122–5p remained dominated by singly bound states (Figure S2). MD simulations indicate that sequence and architecture of miR-122–5p limit the auxiliary contacts, particularly those involving the METTL14 RGG-rich region, that stabilize higher-order binding, so that a second molecule can bind but is not cooperatively enriched. These observations provide direct experimental evidence that RNA sequence and structure dictate not only affinity but also stoichiometry and cooperativity, reinforcing the view that writer–substrate recognition is more versatile than previously appreciated.^10,36–42^

The preference for the two-RNA-bound state for RNAp5 and for r6T is consistent with coupled binding events, and modeling results provide a structural framework for this behavior. In the presence of the METTL14 RGG region, MD simulations converged on an assembly in which two RNA molecules remain bound as separate strands, which could be also viewed as two different parts for larger RNA molecules. In the RNAp5 system, one RNA mainly occupied the groove at the METTL3–METTL14 interface, whereas the second was positioned closer to the METTL3 side near the SAM pocket, with relatively balanced RGG contacts across the two strands (Figures 2 and S12). In the miR-122–5p system, the same architecture was retained, but the two strands were stabilized more asymmetrically (Figures S10–S12). Comparison of the 1× and 2× RNA simulations further suggests that the first RNA partially organizes the flexible RGG region into a composite binding surface capable of accommodating a second RNA without displacing the first (Figures S15–S17). Together with the lower number of inter-RNA hydrogen bonds, these observations suggest that the RGG motif stabilizes multivalent RNA engagement in a substrate-dependent manner, while limiting formation of a stable duplex.

This multivalent behavior may be relevant in cells since cooperative RNA binding could enhance the local avidity and retention of METTL3–METTL14 in RNA-rich nuclear environments, including speckle-associated assemblies where the writer complex concentrates and acts on nascent transcripts.^90^ Moreover, by engaging more than one RNA molecule, the complex may sample nearby substrates or RNA elements more efficiently, a property that could be particularly advantageous for structurally complex RNAs. The RNA binding mode characterized here therefore reflects intrinsic properties of the METTL3–METTL14 catalytic core. Accessory components of the larger m^6^A writer machinery may reshape this binding repertoire by influencing nuclear localization, core positioning, and recruitment to selected transcripts. For instance, WTAP–VIRMA can scaffold and position the catalytic core while also restricting nonproductive nucleic-acid engagement.^91,92^ Thus, *in vivo* specificity likely emerges from the interplay between RNA sequence and structure, intrinsic METTL3–METTL14 RNA-binding surfaces, and recruitment guided by accessory proteins. At the same time, this layered mode of RNA engagement creates a potential vulnerability, as perturbation of the heterodimer interface could propagate across the complex and disrupt RNA engagement beyond the catalytic site.

Guided by this rationale, we engineered a focused library of METTL14-derived peptides to perturb the heterodimer interface, identifying peptide **14** as the minimal loop-mimicking segment retaining low-micromolar binding and inhibition (Figure 3). Importantly, binding alone was not sufficient for inhibition, indicating that productive interface engagement must distort the complex in a way that affects catalysis and/or substrate recruitment. Docking supported this interpretation, placing **14** on METTL3 in a pose closely overlapping the corresponding METTL14, consistent with a competitive METTL14-mimetic binding mode.

Among the developed peptides, the cell-penetrating peptide **R8–14** was the most effective disruptor, combining <1 μM inhibition with the strongest effects on heterodimer integrity and RNA engagement. For the cooperatively binding substrates RNAp5 and r6T, peptide treatment selectively depleted higher-occupancy RNA-bound states without accumulation of singly bound intermediates, consistent with collapse of a cooperatively stabilized multivalent assembly rather than uniform weakening of RNA affinity (Figure 4). This loss of RNA is accompanied by the release of free METTL3 and METTL14, indicating that the peptides destabilize the heterodimer, leading to substrate release, and do not compete directly with RNA at its binding surface. The native MS and modeling results support and explain each other on this point. Modeling places the peptides at the METTL3 interface (Figure 3), away from the RNA-contacting groove and the METTL14 RGG surface, confirming that peptide and RNA occupy distinct sites. Native MS shows that, once the heterodimer is disrupted, RNA is retained on METTL14, including as a two-RNA-bound species, identifying it as the principal RNA-binding subunit. This is in line with simulations showing that the METTL14 RGG region contacts both RNA molecules and stabilizes their simultaneous binding (Figure 2). This also explains why displacement is cooperative: since the two-RNA-bound assembly depends on contacts organized around the intact interface, disrupting the interface collapses the higher-occupancy state selectively. The same logic underlies recent interface-directed inhibitors of nucleic-acid-modifying enzymes, such as the allosteric, PPI-disrupting inhibitors of DNMT3A.^93^ Together, these findings establish a mechanistic link between heterodimer integrity and RNA binding stoichiometry, addressing a key unresolved aspect of the writer–substrate engagement.^35^

The cellular relevance of these biophysical findings was supported in cancer models known to depend on METTL3–METTL14 activity. Consistent with this framework, **R8–14** preferentially reduced viability in MOLM-13 and MDA-MB-231 cells (Figure 5), consistent with selective cytotoxicity in contexts where the METTL3–METTL14 axis is more strongly linked to proliferation and survival.^12,13,19–28^ Importantly, the lack of activity of R9 and weakly active **R8–12** argues against nonspecific polyarginine toxicity and supports the requirement for an effective intracellular pharmacophore. A full quantitative correlation between biochemical and cellular potencies will nonetheless require matched measurements of membrane integrity and intracellular peptide concentration, a natural direction for further optimization of these compounds.

Mechanistically, the reduction of c-Myc in MOLM-13 is consistent with suppression of METTL3-driven oncogenic programs in AML,^12,13,19–22^ while the decrease in global m^6^A supports a direct impact on the writer output. Notably, these changes occurred without detectable METTL3 or METTL14 degradation, indicating that interface targeting can inhibit function independently of protein turnover. Together, these results link interface engagement, heterodimer destabilization, and reduced RNA binding to altered m^6^A output and cancer cell phenotypes in models where METTL3–METTL14 is a recognized dependency. This approach may extend to the larger m^6^A writer machinery, where interactions involving accessory components may represent pharmacologically actionable nodes, for instance by disrupting contacts between the catalytic core and accessory subunits or interfering with adaptor-mediated recruitment. Thus, interface-directed modulation of the METTL3–METTL14 core provides a proof-of-concept for pharmacologically exploiting the broader architecture of the m^6^A writer complex.

## Conclusion

This work establishes native MS, complemented by AI-driven molecular modeling, as a quantitative platform to interrogate RNA-binding stoichiometry and interface perturbation in the METTL3–METTL14 writer complex. Together, these approaches show that METTL3–METTL14 behaves as a multivalent RNA-binding assembly whose engagement is shaped by RNA identity and supported by the METTL14 RGG-rich region, and that heterodimer integrity is required to sustain higher-order RNA binding. Interface-targeting METTL14-derived peptides exploit this coupling: the cell-penetrating peptide **R8–14**, which emerged as the most effective disruptor, links heterodimer destabilization to loss of multivalent RNA engagement, reduced cellular m^6^A output, and selective antiproliferative effects in METTL3-dependent cancer models.

Collectively, our study advances the molecular understanding of how METTL3–METTL14 recognizes RNA substrates and identifies its multivalent RNA-binding behavior as both a mechanistically important feature and a pharmacologically actionable vulnerability. By integrating native MS, molecular modeling, and rational peptide design, we establish a framework to interrogate RNA-binding stoichiometry and develop interface-targeting disruptors of writer function. Beyond METTL3–METTL14, this framework may be extended to the whole m^6^A writer machinery, including its accessory regulatory components, as well as to other RNA-modifying enzymes and multicomponent ribonucleoprotein assemblies. Interrogating protein–protein and RNA–protein interfaces in these systems may enable mechanistic dissection and therapeutic modulation of RNA-regulatory machines, offering complementary strategies beyond conventional active site inhibition.

## Supplementary Material





## Data Availability

All supporting files related to the Chai-1 analyses, including predicted poses and associated confidence scores, Gnina docking poses, TPRS files, and raw molecular dynamics analysis files, have been deposited in OSF and are publicly available at: https://osf.io/n6ysv/.

## Notes

Materials and Methods, Synthetic schemes for peptides **15–21** (Scheme S1) and **22–23** (Scheme S2), supporting native MS characterization of RNA self-association (Figure S1), native MS analysis of METTL3–METTL14 binding to miR-122–5p (Figure S2), native MS evaluation of binding to AGO2 guide RNA and miR-20a-5p (Figure S3), AI and MD analysis of the METTL3–METTL14/RNAp5 complex (Figure S4), conformational clustering analysis of MD trajectories (Figure S5), interstrand RNAp5 hydrogen-bond analysis (Figure S6), MD distance analyses for the catalytic SAM–RNAp5 target adenosine in RGG-containing and RGG-lacking complexes (Figures S7–S9), AI and MD analysis of the METTL3–METTL14/miR-122–5p complex (Figure S10), comparative interaction patterns of RNAp5 and miR-122–5p in the 2× RNA-bound METTL3–METTL14 (+ RGG motif). (Figure S11), base-level analysis of RGG motif-RNA contacts in METTL3-METTL14 complexes containing two miR-122–5p or two RNAp5 molecules (Figure S12), MD distance analyses for the catalytic SAM–miR-122–5p target adenosine in RGG-containing and RGG-lacking complexes (Figures S13–S14), representative structures of the 1× RNA-bound METTL3–METTL14 (+RGG) complexes (Figure S15), RNA structural dynamics in the 1× RNA-bound METTL3–METTL14 systems. (Figure S16), per-residue RNA flexibility in the 1× RNA-bound METTL3–METTL14 systems (Figure S17), structural illustration of the METTL3–METTL14 interface and truncation strategy (Figures S18 and S19), chemical structures of peptides **11–23** (Figure S20), native MS screening of peptides **11–23** (Figure S21), K_D_ fitting (Figure S22), docking and MD analysis of peptide binding to the METTL complex (Figure S23), native MS competition experiments with RNAp5, r6T, and miR-122–5p in the presence of peptides (Figures S24–S26), native MS analysis of R9 binding to METTL3-METTL14 (Figure S27), supporting antiproliferative activity and IC_50_ determinations in cancer cell lines (Figures S28–S29), effects on cell viability of noncancer cells (Figure S30), WB analysis of METTL3 and METTL14 levels in MOLM-13 cells in the presence or absence of MG132 following peptide treatment (Figure S31), WB analysis of METTL3 and METTL14 levels in MDA-MB-231 and MOLM-13 cells following peptide treatment (Figure S32), WB analysis of WTAP levels in MOLM-13 cells and global m^6^A quantification in MDA-MB-231 cells after peptide treatment (Figure S33), IC_50_ values for peptide effects on cell viability (Table S1), HPLC and HRMS characterization data for peptides **4**, **8**, **9**, **11–23**, RM3, SM3, and RSM3 (PDF)
MD trajectory of the METTL3–METTL14 (+RGG) complex bound to two RNAp5 substrates (MP4)

## Abbreviations

AML: acute myeloid leukemia
CLIPS: chemical linkage of peptides onto scaffolds
m^6^A: N^6^-methyladenosine
MD: molecular dynamics
MS: mass spectrometry
PPI: protein–protein interaction
RMSD: root-mean-square deviation
RMSF: root-mean-square fluctuation
SAM: S-adenosyl-l-methionine
