# Supplementary material for: METTL3–METTL14 Heterodimer Assembly Enables Multivalent RNA Engagement and Is Tuned by Interface-Targeting Peptides
Source: J Am Chem Soc. 2026 Oct 7;148(41):44138–55. doi: 10.1021/jacs.6c09634 (PMC13693457; doi:10.1021/jacs.6c09634)
Supplement: Supplementary file 1 [file ja6c09634_si_001.pdf]

Supporting Information

METTL3–METTL14 heterodimer assembly enables multivalent RNA engagement and is tuned by interface-targeting peptides

Francesco Fiorentino[1], Boyi Xiao[2], Bianca Cesaro[3], Sara Venezia[3], Jujun Zhou[4], Emanuele Fabbrizi[5], Fabrizio Casano[5], Gebremedhin Solomon Hailu[6], Xiaodong Cheng[4], Robin A. Corey[2], Alessandro Fatica[3], Antonello Mai[5], Stefano Tomassi[7*], Dante Rotili[6,8*]*

[1] Department of Biochemical Sciences "A. Rossi Fanelli", Sapienza University of Rome, Piazzale Aldo 5, 00185, Rome, Italy.

[2] School of Physiology, Pharmacology & Neuroscience, University of Bristol, Biomedical Sciences Building, University Walk, Bristol BS8 1TD, UK.

[3] Department of Biology and Biotechnologies "Charles Darwin", Sapienza University of Rome, Rome, Italy

[4] Department of Epigenetics and Molecular Carcinogenesis, University of Texas MD Anderson Cancer Center, Houston, Texas 77030, USA.

[5] Department of Drug Chemistry and Technologies, Sapienza University of Rome, Piazzale Aldo 5, 00185, Rome, Italy.

[6] Department of Science, Roma Tre University, Viale Marconi 446, 00146, Rome, Italy.

[7] Department of Life Sciences, Health and Health Professions, Link Campus, Via del Casale di S. Pio V, 44, 00165, Roma, Italy.

[8] Biostructures and Biosystems National Institute (INBB), Via dei Carpegna 19, 00165, Rome, Italy.

[*]Correspondence: Dante Rotili (dante.rotili@uniroma3.it), Stefano Tomassi (s.tomassi@unilink.it), Antonello Mai (antonello.mai@uniroma1.it),

MATERIALS AND METHODS

Protein expression and purification

Human METTL3-METTL14 was expressed and purified as previously reported.[1] Briefly, DNA fragments encoding the METTLL3 (residues 1-580) and METTL14 (residues 1-456) amplified separately by PCR and sub-cloned into pBacMam2-DiEx-LIC. The resulting plasmids were transformed into DH10Bac™ Competent *E. coli* (Invitrogen) and recombinant viral DNA bacmids were purified and followed by a recombinant baculovirus generation in Sf9 insect cells. The Sf9 cells grown in HyQ® SFX insect serum-free medium (Thermo Scientific) and were co-infected with 10 ml of P3 viral stocks per 0.8L of suspension cell culture and incubated at 27 °C using a platform shaker set at 100 RPM. The cells were collected after 72 h of post infection time, when viability dropped to 70-80%. Harvested cells were re-suspended in PBS, 1× protease inhibitor cocktail (100× protease inhibitor stock in 70% ethanol (0.25 mg/ml Aprotinin, 0.25 mg/ml Leupeptin, 0.25 mg/ml Pepstatin A and 0.25 mg/ml E-64) and 2X Roche complete EDTA-free protease inhibitor cocktail tablet. The cells were lysed chemically by rotating 30 min with NP40 (final concentration of 0.6%), 50 U/mL benzonase nuclease (Sigma), 2 mM 2-mercaptoethanol and 10% glycerol, followed by sonication at frequency of 7 (10 s on/10 s off) for 2 min (Sonicator 3000, Misoni). The crude extract was clarified by high-speed centrifugation (60 min at 36,000×g at 4 ˚C) by Beckman Coulter centrifuge. The recombinant protein was purified by incubating the cleared lysate with equilibrate Anti-FLAG M2 Affinity agarose gel (Sigma Aldrich, Cat # A2220) for 3 h rotating, followed by wash with 10 CV TBS (50 mM Tris-HCl, with 150 mM NaCl, pH 7.4) with 2 mM 2-mercaptoethanol, 1× protease inhibitor cocktail (100× protease inhibitor stock in 70% ethanol, same as above) and 1× Roche 7 complete EDTA-free protease inhibitor cocktail tablet. The recombinant protein was eluted by competitive elution with a solution containing 100 µg/mL FLAG peptide (Sigma Aldrich, Cat # F4799) in TBS in addition of 1 mM dithiothreitol and 10% glycerol. The purity of the complex was evaluated by sodium dodecyl sulfate polyacrylamide gel electrophoresis (SDS-PAGE). The protein complexes were concentrated and flash frozen.

Native mass spectrometry

Purified METTL3-METTL14 was buffer exchanged into MS Buffer (200 mM ammonium acetate pH 7.5) through dialysis at 4 °C for 16 h. The concentration of the freshly buffer-exchanged protein was determined using an N50 NanoPhotometer (IMPLEN). The protein samples were further diluted as desired in 200 mM ammonium acetate buffer as necessary and loaded into a gold-coated capillary Clark borosilicate capillary (Harvard Apparatus) prepared in-house.[2] The experiments were performed on a Q Exactive UHMR Hybrid Quadrupole-Orbitrap mass spectrometer (Thermo Fisher Scientific) in positive polarity. The instrument parameters used for data collection were: capillary voltage 1.2 kV, S-lens RF 100%, scan range from 2,000 to 10,000 m/z, HCD collision energy 150 V, source fragmentation 0 V, in-source trapping 0 V. The ion transfer optics was set as follows: injection flatapole 5V, inter-flatapole lens 4V, bent flatapole 2 V, transfer multipole 0 V. The resolution of the instrument was 4375 at m/z = 200 (transient time of 64 ms), argon pressure in the HCD cell was maintained at approximately at 5×10^{-10} mbar and source temperature was kept at 100 °C. The

noise level was set at 3 rather than the default value of 4.64. Calibration of the instruments was performed using 10 mg/ml solution of cesium iodide in water. Data were analyzed using the Xcalibur 3.0 (Thermo Scientific), NaViA,[3] and UniDec[4] software packages.

Ligand binding experiments and relative quantification

RNAp5 was purchased from Sino Biological. R6T and miR-122-5p were purchased from Biomers. Peptides **1-3**, **5-7**, and **10** were purchased from Biomatik, while peptides **4**, **8**, **11-23**, RM3, SM3, and RSM3 were synthesized in-house as detailed below. Peptide R9 was purchased from MedChemExpress.

RNA and peptide binding assays were prepared by dilution in 200 mM ammonium acetate (pH 7.5). Native MS measurements were acquired at a total METTL3–METTL14 concentration of 2.5 μM. Peak intensities were extracted using UniDec, and binding was quantified as the ratio of the summed intensities of RNA- and/or peptide-bound species to the total intensity of all detected species. For relative quantification of free METTL3, the intensity of peaks assigned to METTL3 was expressed relative to those assigned to the METTL3–METTL14 complex. All experiments were performed in triplicate (n=3).

The apparent dissociation constant (K_D) values for individual peptide-binding events to METTL3–METT14 were obtained by adding increasing amounts of peptide while keeping the protein concentration constant. The reported values were not corrected for peptide binding to free METTL3. Peak intensities were extracted and the ratios of the intensity of the 1× peptide-bound species to the total intensity of all assigned heterodimeric species was calculated.[5] Average and S.D. values of these ratios from three independent experiments were plotted against peptide concentration. The data were fitted using GraphPad Prism 8.0 with the equation:

$$y = \frac{B_{max} \cdot \left[([P_{tot}] + x + K_D) - \sqrt{([P_{tot}] + x + K_D)^2 - 4[P_{tot}]x}\right]}{2[P_{tot}]}$$

where x refers to peptide concentration, y is the molar ratio of protein-peptide species, $[P_{tot}]$ is the total protein concentration (2.5 μM) and B_{max} is the maximum specific binding, in the same units as y. All observed peptide-bound species were included in the calculated molar ratio; however, only the first peptide-binding event was fitted, as higher-order events were weakly populated and prone to nonspecific adduct contributions at high peptide concentrations.

Peptide synthesis and analysis

Peptides **4**, **8**, **11-23** were synthesized were synthesized using an ultrasound-assisted solid-phase peptide synthesis (US-SPPS) protocol.[6] Cyclic peptides based on cysteine thiol reactivity (**15-21**) were assembled on a solid support, cleaved from the resin, and then cyclized in solution as unprotected oligomers by mild oxidation (**15**) or by reaction with aliphatic/aromatic dibromides (**16-21**). Lactam-bridged peptides **22-23** were cyclized on-resin, with the isopeptide bond introduced between the Nα of the terminal Gly or GABA and a Glu residue at position 282, following removal of the orthogonal O-allyl ester protecting group (Schemes S1-S2). Peptides RM3, SM3, and RSM3 were synthesized as previously reported.[7]

Standard Nα Fmoc-amino acids, trifluoroacetic acid or TFA (purity 99%), (1-cyano-2-ethoxy-2-oxoethylidenaminooxy)dimethylamino-morpholinocarbenium hexafluorophosphate or COMU (CAS 1075198-30-9), Ethyl 2-cyano-2-(hydroxyimino)acetate or OXYMA pure (CAS 3849-21-6), *N,N*-diisopropylethylamine or DIPEA (CAS 7087-68-5) piperidine (CAS 110-89-4), Rink amide resin (medium loading) were purchased from IRIS Biotech (Marktredwitz, Germany). Triisopropylsilane or TIS (CAS 6485-79-6), acetic anhydride or Ac_2O (purity > 98 %), ammonium carbonate 99.999% trace metals basis (CAS 506-87-6), D,L-dithiothreitol solution 1 M in water (CAS 3483-12-3), α,α'-dibromo-para-xylene (CAS 623-24-5), α,α'-dibromo-meta-xylene (CAS 626-15-3) and α,α'-dibromo-ortho-xylene (CAS 91-13-4), dichloroacetone (CAS 534-07-6), diiodomethane (CAS 75-11-6), diiodoethane (CAS 624-73-7) were purchased from Sigma-Aldrich. Peptide synthesis solvents, water and acetonitrile for HPLC, were reagent grade and were acquired from commercial sources (Sigma-Aldrich, Milano, Italy) and used without any further purification unless otherwise stated. Peptides were assembled on Macherey-Nagel Chromab. vacuum manifold and using an ultrasonic bath SONOREX RK 52 H (interior dimensions 150 × 140 × 100 mm and operating volume 1.2 L) by BANDELIN electronic (Germany). Peptides were purified on a Dionex UltiMate 3000 UHPLC (Thermo Fisher) equipped with with a Diode Array Detector DAD-3000 (Thermo Fisher) and a C18-bounded semi-preparative RP-HPLC column (Hypersil GOLD™ C18 Selectivity Reversed Phase HPLC Column, 5 µm, 10 × 250 mm, Thermo Fisher). Peptides were analyzed by analytical HPLC on the same HPLC system as above equipped with a C18-bounded analytical RP-HPLC column (Hypersil GOLD™ C18 Selectivity 5 µm 4.6 × 250 mm, Thermo Fisher) using a gradient elution. Eluents: A) H_2O + 0.1% TFA; B) CH_3CN + 0.1% TFA. The chromatographic run comprised 5 min at 10% solvent A, a subsequent 20 min linear gradient from 10% to 90% solvent B, and a final 5 min isocratic step at 90% solvent B. The flow rate was 1.0 mL/min, and the column was kept at a constant temperature of 30 °C. High-resolution mass spectrometry (HRMS) was performed using a Q Exactive UHMR Hybrid Quadrupole-Orbitrap mass spectrometer (Thermo Fisher) in positive polarity for all peptides except peptide **9**. The instrument parameters used for MS spectra collection were the following: capillary voltage of 1 kV, scan range of 350 to 5000 m/z, HCD collision voltage of 0 V, source fragmentation of 0 V, and in-source trapping of 0 V. The ion transfer optics were set as follows: injection flatapole of 5 V, inner-flatapole lens of 4 V, bent flatapole of 2 V, and transfer multipole of 0 V. The resolution of the instrument was 200,000 at m/z = 400, the nitrogen pressure in the HCD cell was maintained at approximately 1.0×10^{-10} mbar, and the source temperature was kept at 100 °C. Calibration of the instrument was performed using a 10 mg/mL solution of cesium iodide in water. Data were analyzed using the Xcalibur 3.0 (Thermo Scientific). Solutions for flow injection analysis were constituted by dissolving each compound in LC-MS-grade MeOH (c = 50 µM).

AI-based modeling with Chai-1

The AI-based protein-folding software Chai-1[8] was used (via the webserver https://lab.chaidiscovery.com/) to predict the structure of the METTL3-METTL14 complex alone or with two bound RNA molecules. For METTL3, residues 356-580 of UniProt ID Q86U44 were used For METTL14, residues 263-407 or 263-456

(with RGG motif) of UniProt ID Q9HCE5 were used. For the RNA, the RNAp5 motif was chosen. For each submission, Chai-1 generated 5 output structures poses for further analysis.

Peptide docking with GNINA

Poses for peptide **14** against METTL3 were generated using the GNINA docking software,[9] which utilizes a convolutional neural network (CNN) to score poses. Docking was performed against the METTL3 subunit of the crystal structure of the METTL3–METTL14 complex (PDB ID: 5IL0). We performed a global docking, i.e. with no defined docking box. The top-ranked pose selected for structural analysis showed a GNINA affinity score of −6.715 and a CNN score of 0.81. Note, adding the R8-tag to the peptide dramatically decreased the confidence of the pose, so these were not used for further analyses.

Molecular dynamics simulations

MD simulations were conducted to assess the stability and understand the dynamics of the complex over time. Selected model from the Chai-1 and GNINA analyses were each built into solvent boxes containing TIP3 waters and neutralized with sodium chloride ions at concentration 0.15M via CHARMM-GUI Solution Builder.[10] All molecules were described using the CHARMM36m forcefield.[11]

Using GROMACS 2024.4,[12] the systems were minimized and equilibrated according to standard CHARMM-GUI protocol. MD simulations were then conducted across each system for 500 ns with a total of 5 repeats. Simulations used a 2 fs time step in the NPT ensemble with the velocity-rescale thermostat and C-rescale pressure coupling. The results of the MD simulations were analyzed using built-in GROMACS tools.

Cell cultures and reagents

Human breast adenocarcinoma cell lines MDA-MB-231 and MCF-7, human acute myeloid leukemia cell line MOLM13, human ovarian adenocarcinoma SK-OV-3, human osteosarcoma MG-63, B lymphocyte AHH1, and mammary epithelial cells (HME) cell lines were purchased from the American Type Culture Collection (ATCC, Manassas, VA, USA). MDA-MB-231, MCF-7, and SK-OV3 were cultured in Dulbecco's Modified Eagle Medium (DMEM) medium (Gibco, Thermo Fisher Scientific) with 10% fetal bovine serum (FBS, Gibco), 1× L-Glutamine (Gibco), 2× Penicillin-Streptomycin (Gibco). MOLM13, MG-63, and AHH1 were cultured in Roswell Park Memorial Institute (RPMI) medium 1640 (Gibco) with 10% FBS, 1× L-Glutamine, 1× Penicillin-Streptomycin. HME CELLS were cultured in Human Mammary Epithelial Cell Basal Medium (Lonza) supplemented with Bovine Pituitary Extract (0.004 ml/ml), recombinant human Epidermal Growth Factor (10 ng/ml), Insulin (5 µg/ml), Hydrocortisone (0.5 µg/ml), and antibiotics. All cell lines were growth at 37 °C under an atmosphere containing 5% CO_2. Cell lines were routinely tested for mycoplasma contamination with LookOut Mycoplasma PCR Detection Kit (Merck KGaA, Darmstadt, Germany). MG132 was used 10 µM for 6 h.

Cell growth and viability analysis

Data for growth curves were obtained with the Countess 3 Automated Cell Counter (Thermo Fisher Scientific) and trypan blue staining. Cell viability was measured by MTT [3-(4,5-dimethylthiazol-2-yl)-2,5 diphenyl tetrazolium bromide] assay using the CellTiter 96 Non-Radioactive Cell Proliferation Assay (Promega Italia, Milan, Italy) after 24 h of treatment. Drug dose–response curves were visualized using linear regression analysis (setting: log(inhibitor) versus normalized response). Half-maximal inhibitory concentration (IC_{50}) values were determined from fitting curves.

Quantification of global N^6-methyladenosine (m^6A) levels

Global quantification of m^6A levels was performed using the EpiQuik m^6A RNA methylation quantification Kit (EpigenTek, Farmingdale, NY, USA) from purified mRNA, according to manufacturer instructions. In brief, mRNA was isolated from total RNA after 24 h of **R8-14** or RSM3 (2.5 µM for MOLM13 and 8 µM for MDA-MB-231), using two rounds of purification with the Dynabeads mRNA purification kit (Thermo Fisher), then 50 ng of purified mRNA were utilized form m^6A quantification.

Immunoblot analysis

Protein extracts were prepared by lysing cells/tissues in RIPA buffer supplemented with protease and phosphatase inhibitors. Lysates were clarified by centrifugation at 13,000 × g for 15 min at 4 °C, and protein concentration was determined using Bradford assay. Equal amounts of protein (20–40 µg) were resolved by SDS–PAGE on polyacrylamide gels and transferred onto nitrocellulose membranes. Membranes were blocked for 1 h at room temperature in 5% non-fat dry milk in TBS-T and then incubated overnight at 4 °C with primary antibodies diluted in 5% BSA.

The antibodies used were: anti-METTL3 (1:1,000, EPR18810, Abcam, Cambridge, MA, USA), anti-METTL14 (1:500, AMAB91276, Atlas Antibodies, Stockholm, Sweden), anti-WTAP (1:1000, 60188-1-Ig, ProteinTech, Rosemont, IL, USA), anti-vinculin (1:10,000, sc-73264, Santa Cruz Biotechnology, TX, USA), anti-p21 (1:500, SC-397, Santa Cruz Biotechnology, TX, USA). After washing with TBS-T, membranes were incubated with secondary antibodies for 1 h at room temperature. Detection was carried out with Clarity Western ECL Blotting Substrate (BioRad, Hercules, CA, USA) using ChemiDocTM MP System and images were analyzed using Image LabTM Software (BioRad).

General synthetic protocol for linear peptides (4, 8, 9, 11, 12, R8-12, 13, 14, R8-14).

A Rink amide resin (85 mg, 0.075 mmol, 0.88 mmol/g) was swollen in a mixture of dichloromethane (DCM) and *N,N*-dimethylformamide (DMF) for 60 minutes, then drained on a solid-phase peptide synthesis (SPPS) manifold (Macherey-Nagel Chromab.) without further treatment. Linear oligomer elongation was performed using an ultrasound-assisted SPPS (US-SPPS) protocol, as previously reported by us.[6, 13] Fmoc deprotection was achieved by treating the resin with 10% piperidine in DMF under ultrasound irradiation in a SONOREX RK 52 H ultrasonic bath (2 × 1 min, ~1.5 mL per treatment). Amino acid couplings were carried out with 3 eq of the corresponding Fmoc-protected amino acids and coupling reagents (COMU and OXYMA Pure), along

with 6 eq of *N,N*-diisopropylethylamine (DIPEA), relative to resin loading. The reactants were dissolved in DMF (2 mL), combined with DIPEA, and transferred to the SPPS reactor, followed by ultrasound irradiation for 5 minutes. After each coupling and deprotection step, the resin was washed sequentially with DMF (2 × 0.5 min) and DCM (2 × 0.5 min). The extent of coupling was monitored by the Kaiser ninhydrin test.

Final acetylation of the resin-bound peptides was performed by treating with acetic anhydride (2 eq) and DIPEA (4 eq) in DMF (1.5 mL), followed by agitation for 20 minutes. The resin was then washed with DMF (3 × 0.5 min), methanol (4 × 0.5 min), and diethyl ether (3 × 0.5 min), and dried thoroughly under vacuum.

Cleavage of crude peptides from the resin was accomplished by treatment with a mixture of trifluoroacetic acid (TFA) and triisopropylsilane (TIS) (95:5 v/v, total volume 3 mL) for 3 hours at room temperature. Following filtration, peptides were precipitated by dilution of the cleavage mixture with 30 mL of cold diethyl ether and centrifuged at 6000 rpm for 15 minutes. The supernatant was discarded, and the peptide pellet was washed once more by resuspension in 30 mL of cold diethyl ether and centrifugation under the same conditions. The resulting solid was dried under vacuum.

Purification of crude peptides was performed by semi-preparative reverse-phase high-performance liquid chromatography (RP-HPLC) using solvent A (water + 0.1% TFA) and solvent B (acetonitrile + 0.1% TFA), with a linear gradient from 10% to 60% solvent B over 20 minutes at a flow rate of 4 mL min^{-1}. Fractions of interest were pooled, concentrated to remove organic solvent, frozen, and lyophilized.

Peptide Ac-Arg-Arg-Cys-Glu-Asp-Ile-Cys-Trp-Ile-Lys-Thr-$CONH_2$ (4).

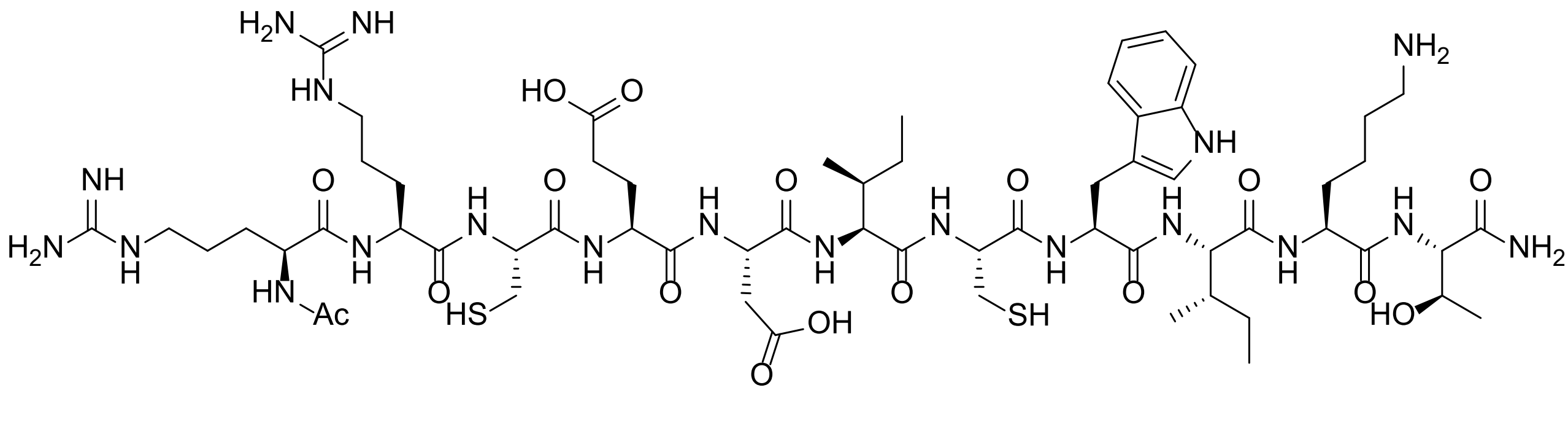

Crude yield: 43 %, purity: 98.52%, t^R 13.057 min, (analytical HPLC, 10 to 90% acetonitrile (0.1% TFA) in water (0.1% TFA) over 20 min, flow rate of 1.0 mL/min); HR-MS (ESI-MS): calculated: 1463.725 for $C_{62}H_{103}N_{20}O_{17}S_2^+$ $[M+H]^+$, found: 1463.727.

Peptide Ac-Lys-Glu-His-Cys-Leu-Met-Gly-Ile-Lys-$CONH_2$ (8).

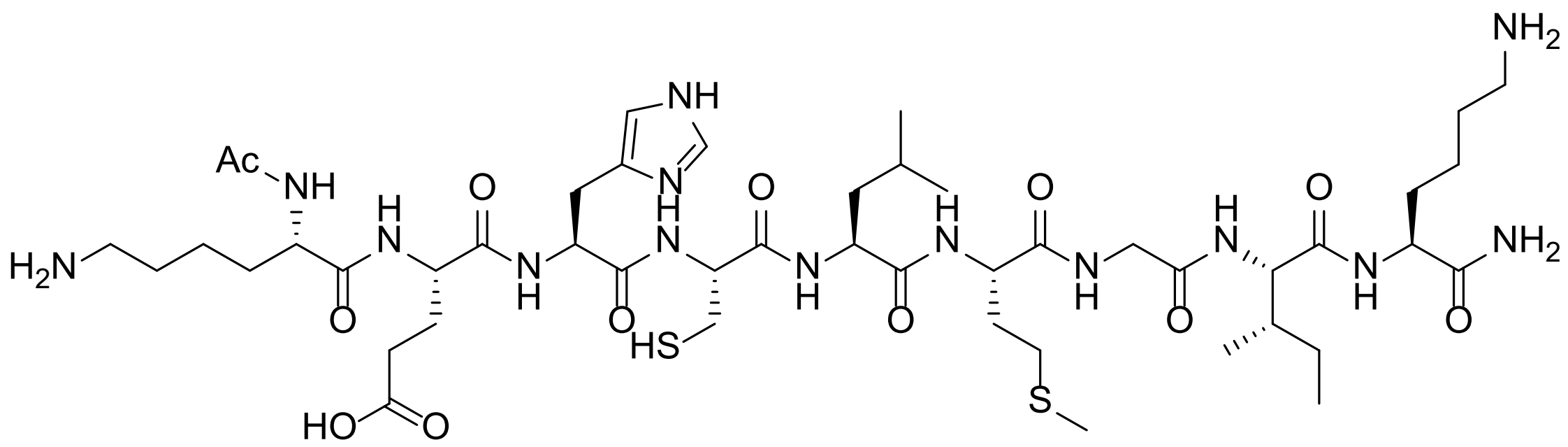

Crude yield: 38 %, purity: 99.54%, t^R 12.150 min, (analytical HPLC, 10 to 90% acetonitrile (0.1% TFA) in water (0.1% TFA) over 20 min, flow rate of 1.0 mL/min); HR-MS (ESI-MS): calculated: 1099.575 for $C_{47}H_{83}N_{14}O_{12}S_2^+$ $[M+H]^+$, found: 1099.575.

Peptide Ac-His-Ala-Asn-Val-Asp-Ile-Asp-Leu-Ile-Ile-Thr-Glu-CONH₂ (9).

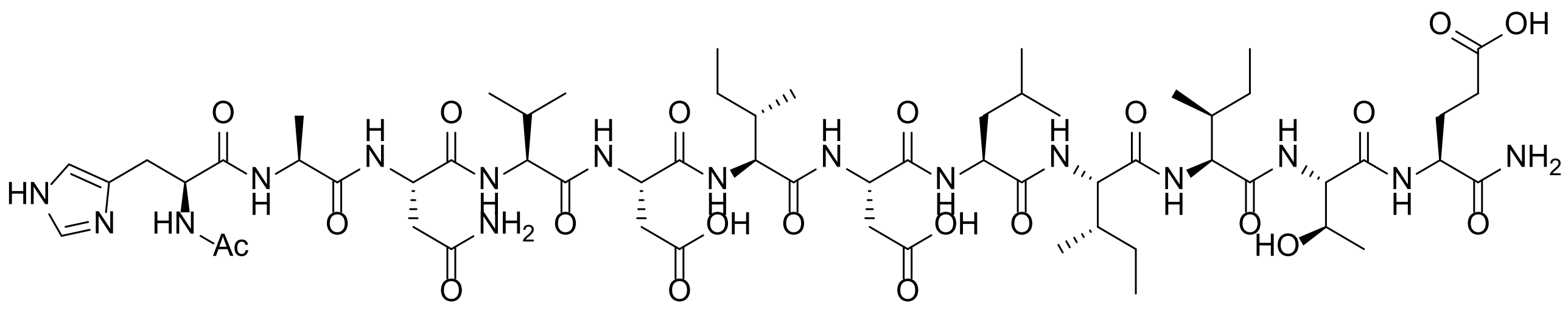

Crude yield: 30 %, purity: 98.32%, t^R 11.240 min, (analytical HPLC, 10 to 90% acetonitrile (0.1% TFA) in water (0.1% TFA) over 20 min, flow rate of 1.0 mL/min); HR-MS (ESI-MS): calculated: 1391.718 for $C_{61}H_{99}N_{16}O_{21}^-$ $[M\text{-}H]^-$, found: 1391.719.

Peptide Ac-Thr-Lys-Thr-Leu-Asp-Pro-Lys-Ala-Val-Phe-Gln-Arg-Thr-CONH₂ (11).

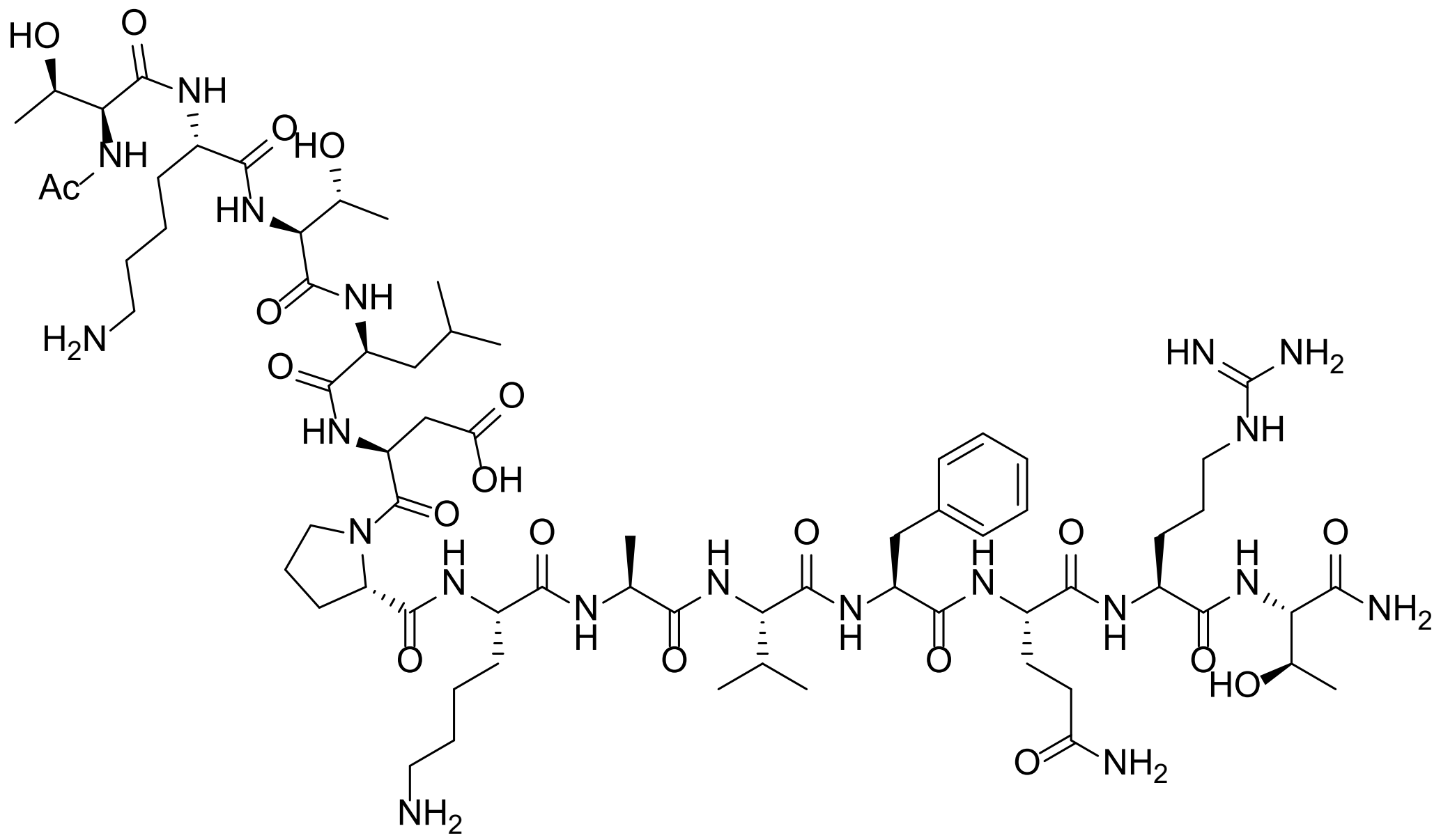

Crude yield: 40 %, purity: 98.72%, t^R 12.500 min, (analytical HPLC, 10 to 90% acetonitrile (0.1% TFA) in water (0.1% TFA) over 20 min, flow rate of 1.0 mL/min); HR-MS (ESI-MS): calculated: 1545.875 for $C_{69}H_{117}N_{20}O_{20}^+$ $[M+H]^+$, found: 1545.863; calculated: 777.441 for $C_{69}H_{118}N_{20}O_{20}^{2+}$ $[M+2H]^{2+}/2$, found: 777.461.

Peptide Ac-Thr-Lys-Thr-Leu-Asp-Pro-Arg-Ala-Val-Phe-Gln-Arg-Thr-CONH$_2$ (12).

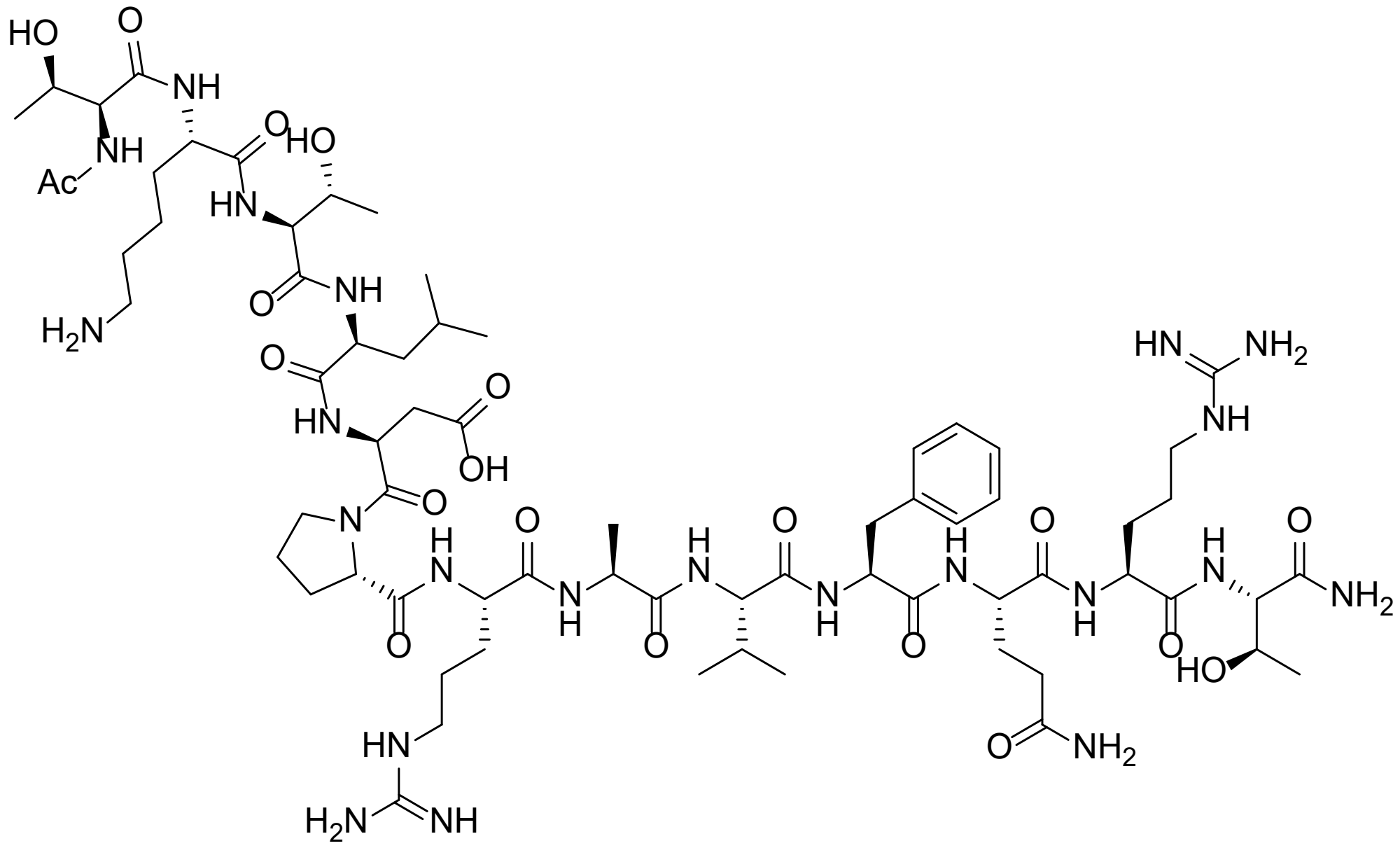

Crude yield: 34 %, purity: 97.52%, t^R 12.533 min, (analytical HPLC, 10 to 90% acetonitrile (0.1% TFA) in water (0.1% TFA) over 20 min, flow rate of 1.0 mL/min); HR-MS (ESI-MS): calculated: 1573.881 for $C_{69}H_{117}N_{22}O_{20}^{+}$ $[M+H]^{+}$, found: 1574.871; calculated: 787.444 for $C_{69}H_{118}N_{22}O_{20}^{2+}$ $[M+2H]^{2+}/2$, found: 787.444.

Peptide NH$_2$-Arg-Arg Arg-Arg-Arg-Arg-Arg-Arg- Thr-Lys-Thr-Leu-Asp-Pro-Arg-Ala-Val-Phe-Gln-Arg-Thr-CONH$_2$ (R8-12).

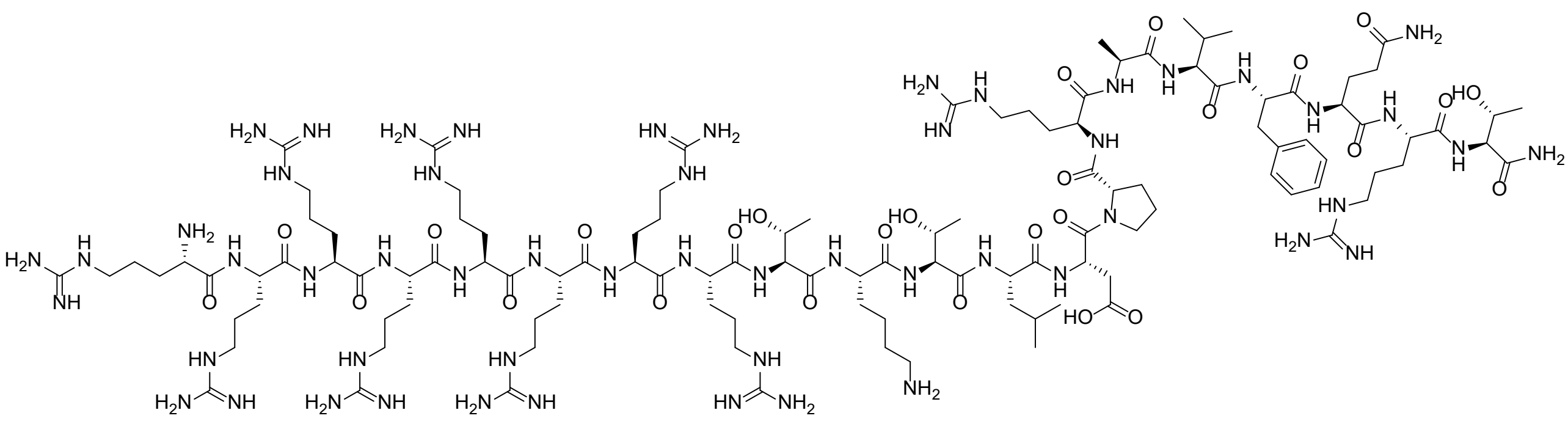

Crude yield: 26 %, purity: 95.25%, t^R 12.070 min, (analytical HPLC, 10 to 90% acetonitrile (0.1% TFA) in water (0.1% TFA) over 20 min, flow rate of 1.0 mL/min); HR-MS (ESI-MS): calculated: 1390.843 for $C_{115}H_{212}N_{54}O_{27}^{2+}$ $[M+2H]^{2+}/2$, found: 1390.843.

Peptide Ac-Thr-Lys-Thr-Leu-Asp-(D-Pro)-Lys-Ala-Val-Phe-Gln-Arg-Thr-CONH₂ (13).

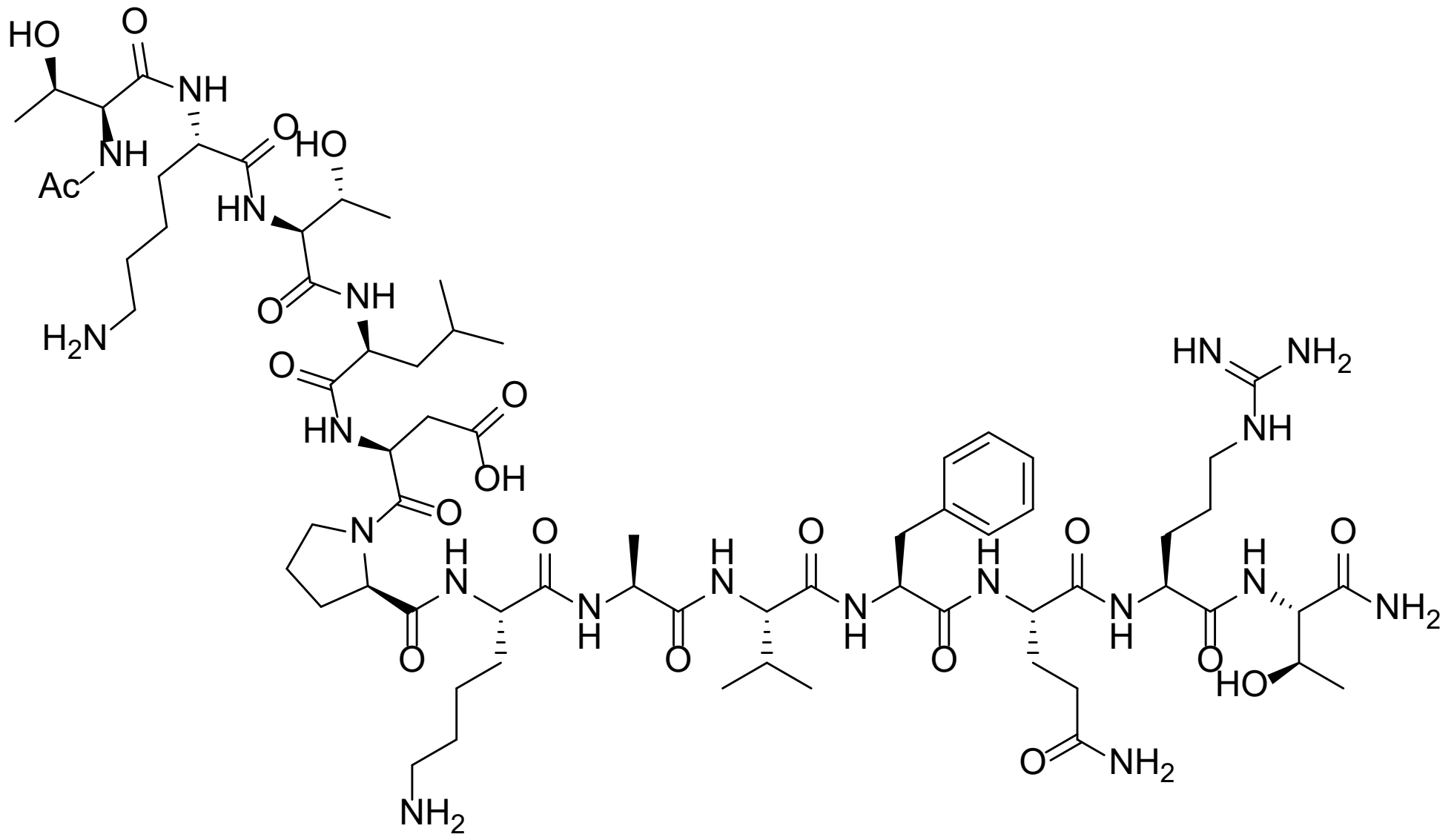

Crude yield: 44 %, purity: 98.23%, t^R 12.030 min, (analytical HPLC, 10 to 90% acetonitrile (0.1% TFA) in water (0.1% TFA) over 20 min, flow rate of 1.0 mL/min); HR-MS (ESI-MS): calculated: 1545.875 for $C_{69}H_{117}N_{20}O_{20}^{+}$ $[M+H]^{+}$, found: 1545.859; calculated: 777.441 for $C_{69}H_{118}N_{20}O_{20}^{2+}$ $[M+2H]^{2+}/2$, found: 777.441.

Peptide Ac-<u>Arg</u>-Ala-Val-Phe-Gln-Arg-Thr-CONH₂ (14).

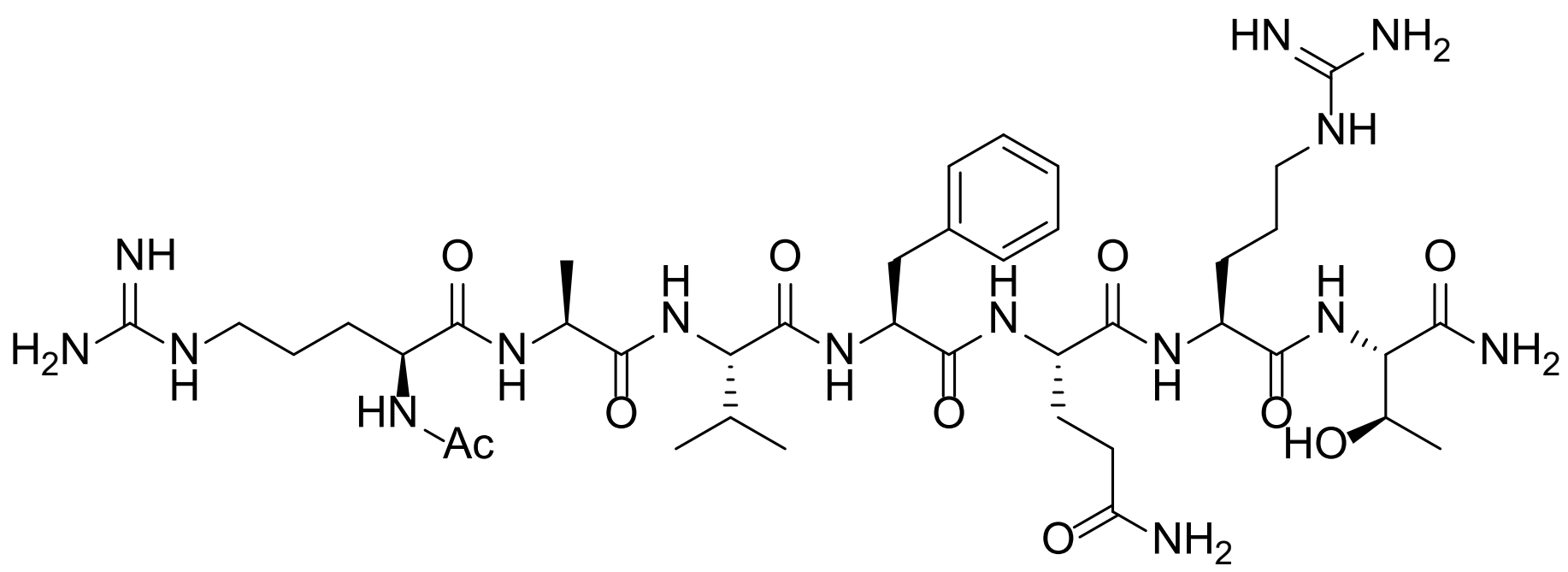

Crude yield: 52 %, purity: 98.58%, t^R 11.850 min, (analytical HPLC, 10 to 90% acetonitrile (0.1% TFA) in water (0.1% TFA) over 20 min, flow rate of 1.0 mL/min); HR-MS (ESI-MS): calculated: 918.527 for $C_{40}H_{68}N_{15}O_{10}$ $[M+H]^{+}$, found: 918.528.

Peptide NH_2-Arg-Arg Arg-Arg-Arg-Arg-Arg-Arg-Arg-Ala-Val-Phe-Gln-Arg-Thr-$CONH_2$ (R8-14).

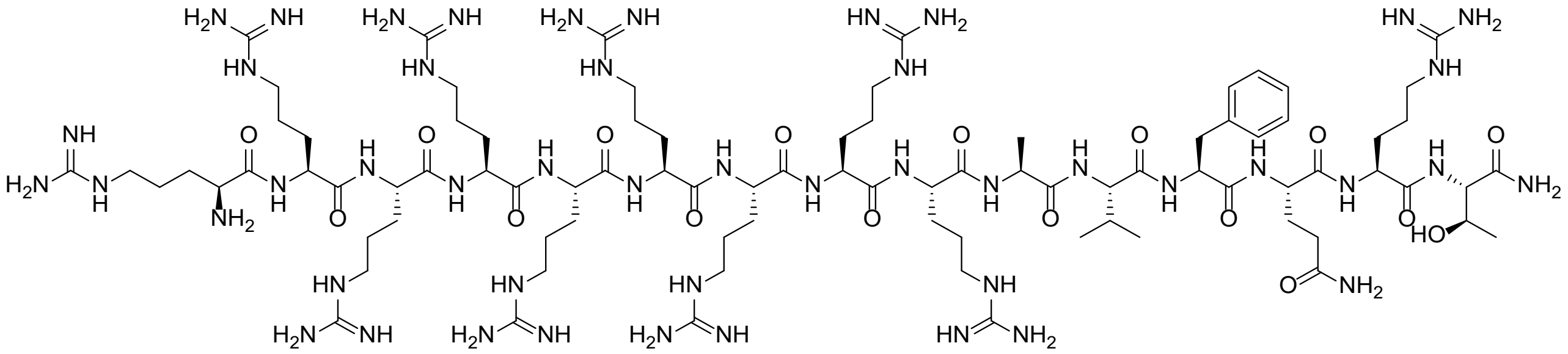

Crude yield: 37 %, purity: 97.27%, t^R 11.267 min, (analytical HPLC, 10 to 90% acetonitrile (0.1% TFA) in water (0.1% TFA) over 20 min, flow rate of 1.0 mL/min); HR-MS (ESI-MS): calculated: 1063.166 for $C_{86}H_{163}N_{47}O_{17}$ $[M+2H]^{2+}/2$, found: 1063.169.

General synthetic procedure for the linear intermediate of thiol-based cyclic peptides (15-21).

The linear version of peptide **15** were assembled as above stated according to the US-SPPS protocol. Once the resin-bound oligomer was obtained, this was cleaved from the solid support using a solution of TFA/TIS/*D*,*L*-Dithiothreitol (95:2.5:2.5, total volume 3 mL) and stirring the resulting mixture for 3h at room temperature. The resin was filtered out and the crude peptides were obtained as TFA salt by precipitation of the acidic medium in 30 mL of cold Et_2O and then centrifuged (6000 rpm × 15 min). The liquid phase was carefully removed and the remaining solid suspended again in 30 mL Et_2O as above stated. The wet solid was dried under vacuum until weight resulted as constant.

Synthesis of peptide Ac-[Cys-Lys-Thr-Leu-Asp-Pro-Lys-Ala-Val-Phe-Gln-Arg-Cys]-$CONH_2$ (15).

Disulfide bond formation was obtained by dissolving the linear oligomer (0.011 mmol, 1 eq) in 11.0 mL of a solution of ammonium carbonate (0.1M, pH = 9.15). The solution was allowed to stir at room temperature at a final concentration of peptide of 1 mM and the cyclization evolution monitored by HPLC. When cyclization was complete, the crude aqueous solution was frozen and then lyophilized. The resulting solid was re-dissolved in a mixture of water/acetonitrile 95:5 and purified by semi-preparative HPLC.

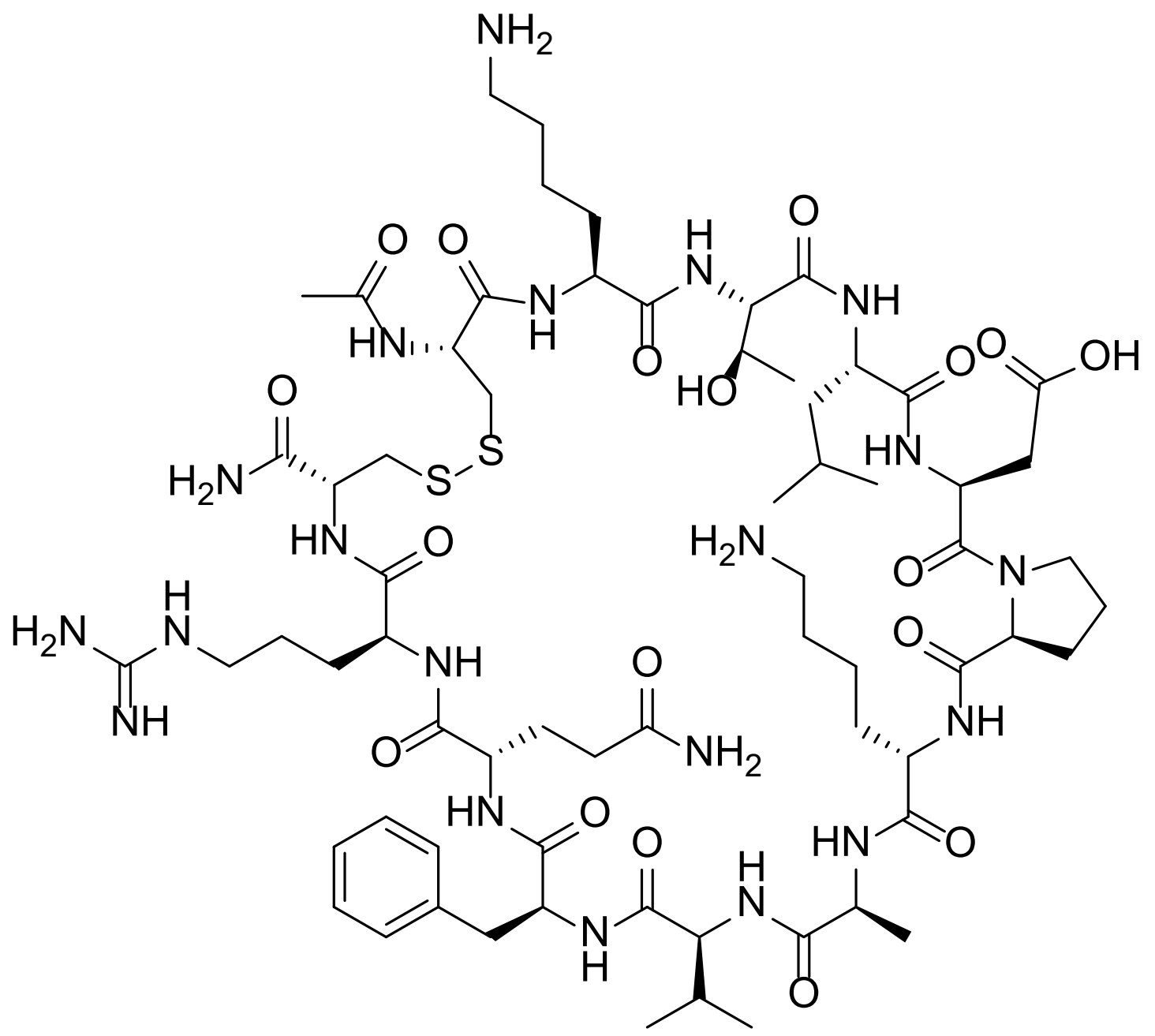

Crude yield: 39 %, purity: 99.61%, t^R 12.767 min, (analytical HPLC, 10 to 90% acetonitrile (0.1% TFA) in water (0.1% TFA) over 20 min, flow rate of 1.0 mL/min); HR-MS (ESI-MS): calculated: 1547.782 for $C_{67}H_{111}N_{20}O_{18}S_2$ $[M+H]^+$, found: 1547.787; calculated: 774.395 for $C_{67}H_{112}N_{20}O_{18}S_2$ $[M+2H]^{2+}/2$, found: 774.400; calculated: 516.599 for $C_{67}H_{113}N_{20}O_{18}S_2$ $[M+3H]^{3+}/3$, found: 516.603.

Synthesis of Chemical LInkage of Peptide onto Scaffold (CLIPS)-based cyclic peptides 16-18.

CLIPS-based cyclization with aliphatic modules was carried out dissolving the linear crude peptide (0.011 mmol, 1 eq) in a solution (22 mL) of THF / water (1:1) at a final concentration of 1 mM. Then TCEP-HCl (tris(2-carboxyethyl)phosphine hydrochloride, 1.5 eq), TEA (triethylamine, 15 eq) and aliphatic dibromide (10 eq) were added. The reaction was stirred for 16 h at room temperature and then cyclization evolution monitored by analytical HPLC. After complete conversion the solution was evaporated from the organic solvent, frozen, and then lyophilized. The resulting solid was re-dissolved in a mixture of water/acetonitrile 95:5 and purified by semi-preparative HPLC.

Peptide Ac-[Cys-Lys-Thr-Leu-Asp-Pro-Lys-Ala-Val-Phe-Gln-Arg-Cys]-Methylene-CONH$_2$ (16).

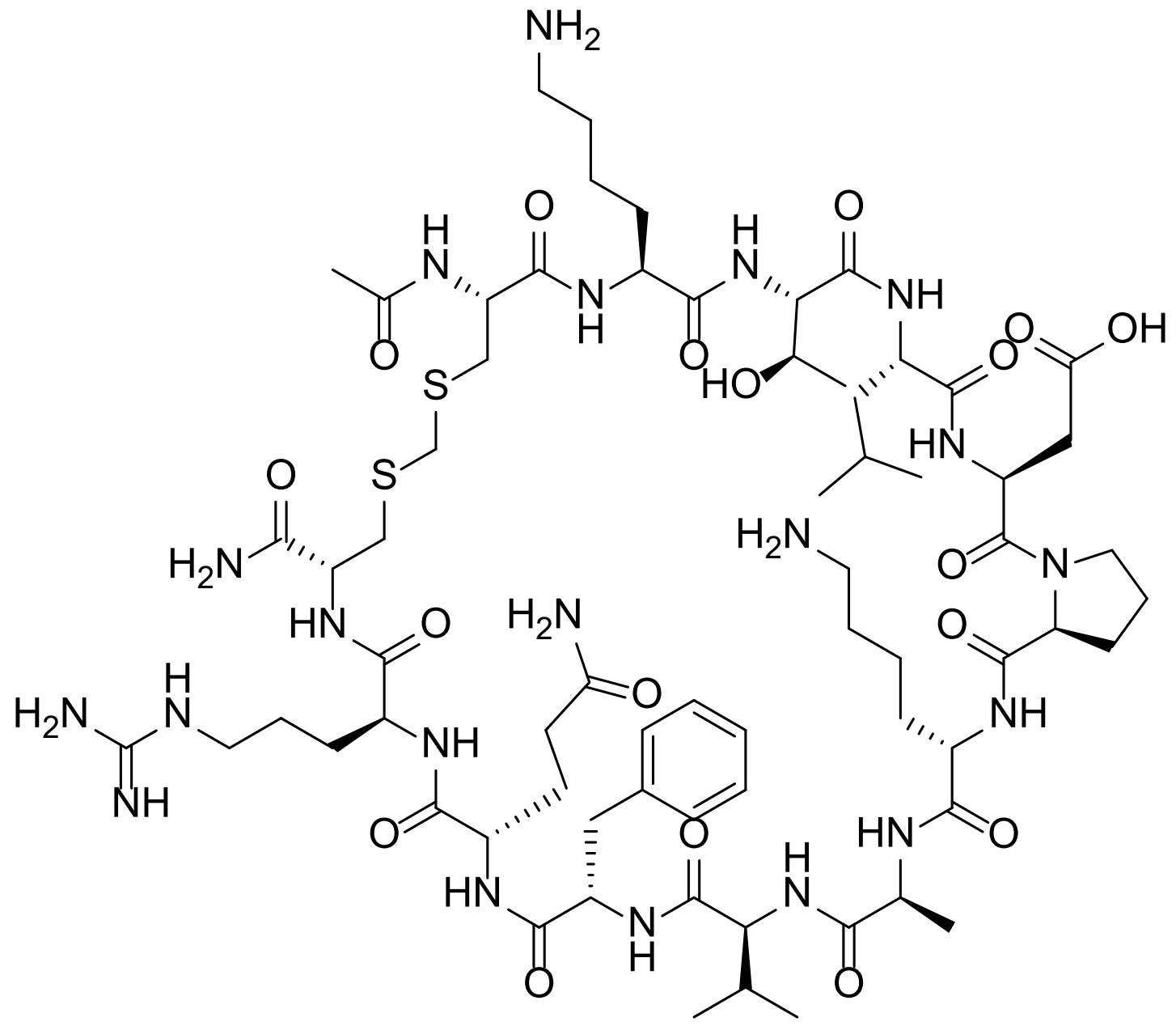

Crude yield: 24 %, purity: 98.52%, t^R 12.697 min, (analytical HPLC, 10 to 90% acetonitrile (0.1% TFA) in water (0.1% TFA) over 20 min, flow rate of 1.0 mL/min); HR-MS (ESI-MS): calculated: 781.403 for $C_{68}H_{114}N_{20}O_{18}S_2$ $[M+2H]^{2+}/2$, found: 781.403; calculated: 521.271 for $C_{68}H_{115}N_{20}O_{18}S_2$ $[M+3H]^{3+}/3$, found: 521.271.

Peptide Ac-[Cys-Lys-Thr-Leu-Asp-Pro-Lys-Ala-Val-Phe-Gln-Arg-Cys]-Ethane-CONH$_2$ (17)

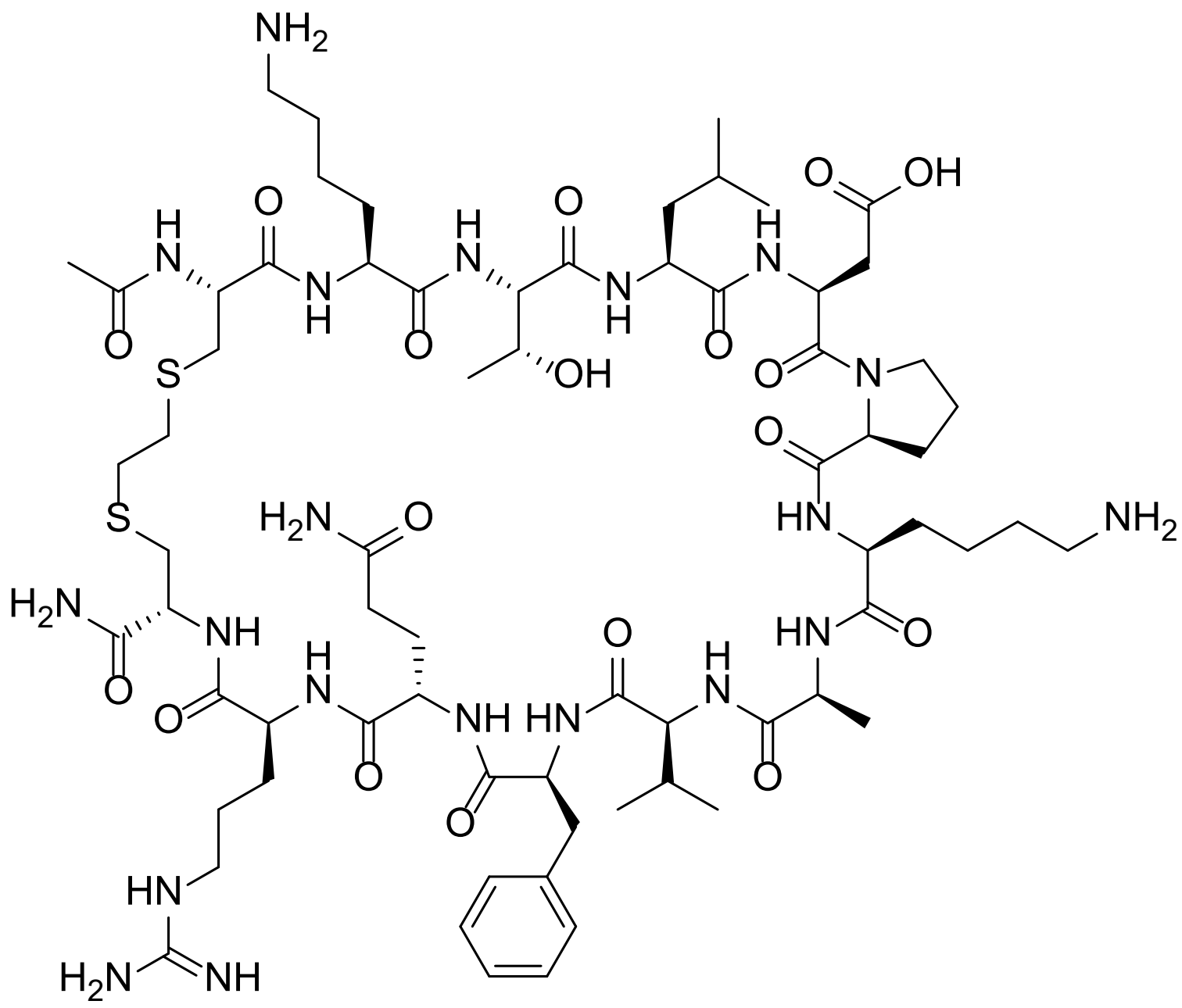

Crude yield: 10 %, purity: 96.36%, t^R 12.803 min, (analytical HPLC, 10 to 90% acetonitrile (0.1% TFA) in water (0.1% TFA) over 20 min, flow rate of 1.0 mL/min); HR-MS (ESI-MS): calculated: 788.410 for $C_{69}H_{116}N_{20}O_{18}S_2$ $[M+H]^{2+}/2$, found: 788.411; calculated: 525.943 for $C_{69}H_{117}N_{20}O_{18}S_2$ $[M+3H]^{3+}/3$, found: 525.943.

Peptide Ac-[Cys-Lys-Thr-Leu-Asp-Pro-Lys-Ala-Val-Phe-Gln-Arg-Cys]-Acetone-CONH₂ (18)

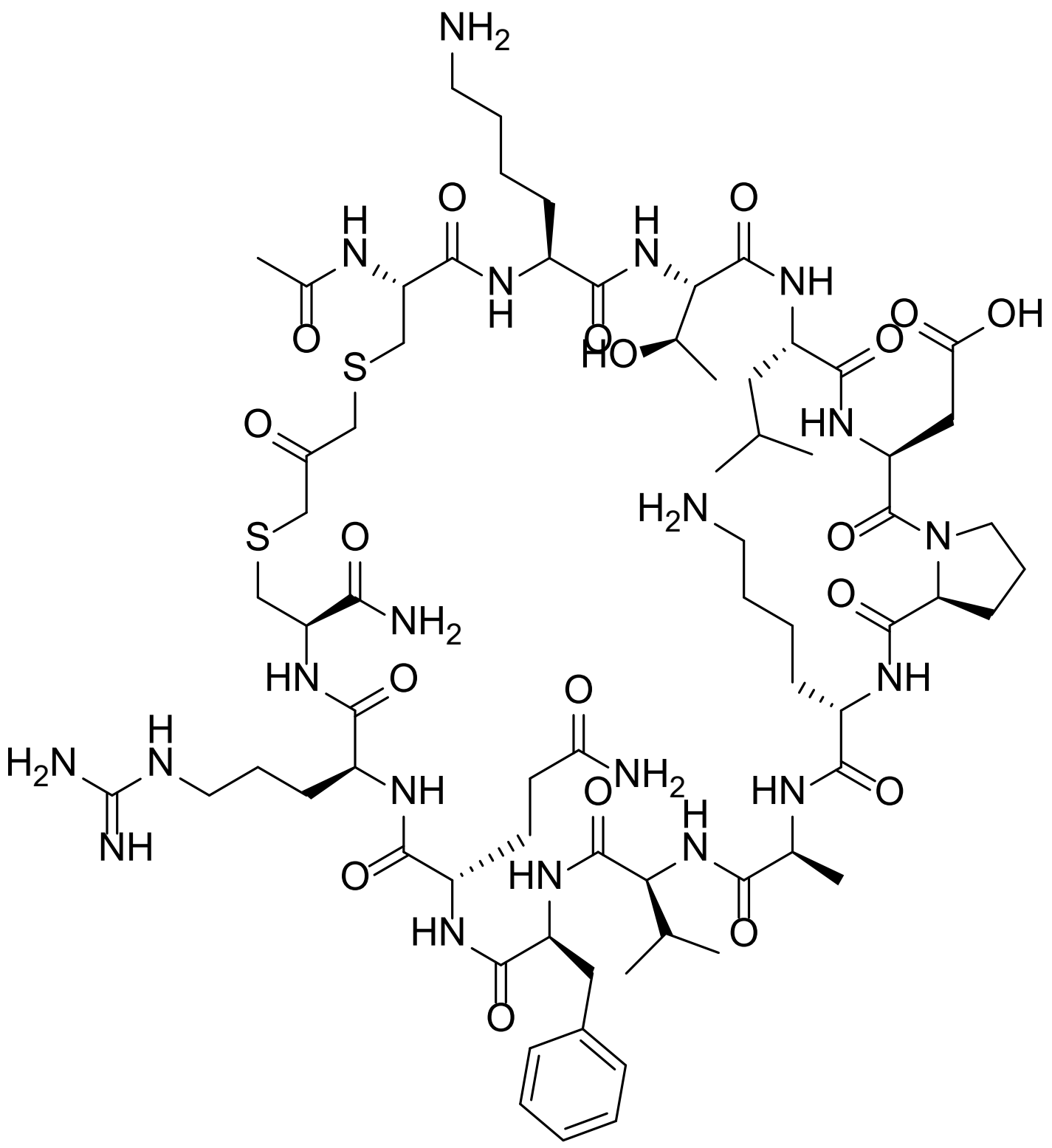

Crude yield: 28 %, purity: ≥ 95%, t^R 12.753 min, (analytical HPLC, 10 to 90% acetonitrile (0.1% TFA) in water (0.1% TFA) over 20 min, flow rate of 1.0 mL/min); HR-MS (ESI-MS): calculated: 802.408 for $C_{70}H_{116}N_{20}O_{19}S_2$ $[M+2H]^{2+}/2$, found: 802.408; calculated: 535.274 for $C_{70}H_{117}N_{20}O_{19}S_2$ $[M+3H]^{3+}/3$, found: 535.274.

Synthesis of Chemical LInkage of Peptide onto Scaffold (CLIPS)-based cyclic peptides 19-21.

CLIPS cyclization with aromatic scaffold was accomplished charging a round-bottom flask with the linear oligomer (0.011 mmol, 1 eq) together with tris(2-carboxyethyl)phosphine (TCEP) (0.044 mmol, 4 eq) and the mixture dissolved in 11 mL of a solution of K_2HPO_4 (0.5M). After complete dissolution, a solution of alkylating agent (0.044 mmol, 4 eq) in acetonitrile (11 mL) was added dropwise at 0 °C. The solution was allowed to stir at room temperature at a final concentration of peptide of 1 mM and the cyclization evolution monitored by HPLC. When cyclization was complete, the organic solvent was evaporated under reduced pressure without heating, and the crude aqueous solution was frozen to be lyophilized. The resulting solid was re-dissolved in a mixture of water/acetonitrile 95:5 and purified by semi-preparative HPLC.

Peptide Ac-[Cys-Lys-Thr-Leu-Asp-Pro-Lys-Ala-Val-Phe-Gln-Arg-Cys]-orthoXylylen-CONH$_2$ (19).

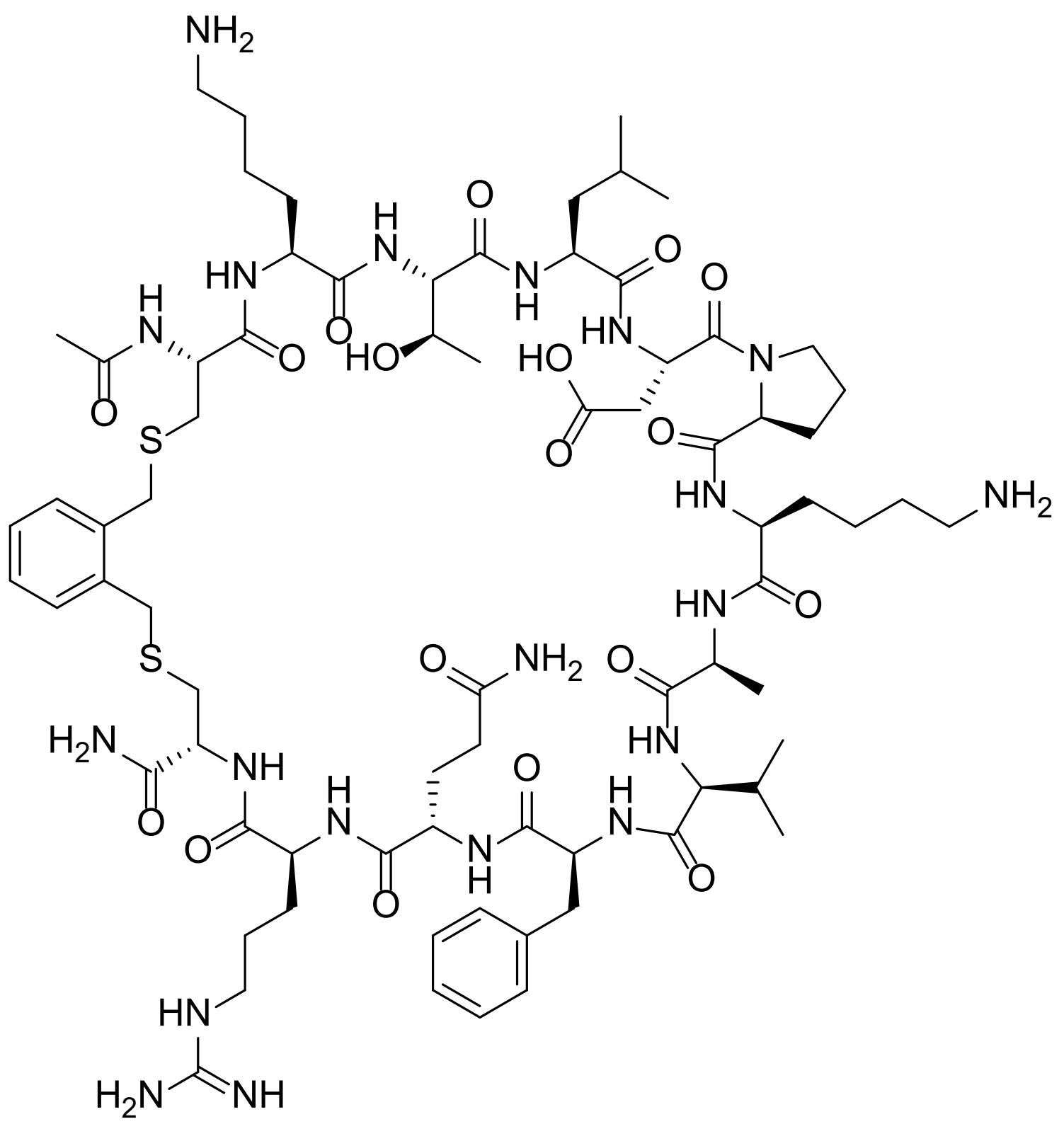

Crude yield: 23 %, purity: 99.71 %, t^R 13.473 min, (analytical HPLC, 10 to 90% acetonitrile (0.1% TFA) in water (0.1% TFA) over 20 min, flow rate of 1.0 mL/min); HR-MS (ESI-MS): calculated: 826.426 for $C_{75}H_{120}N_{20}O_{18}S_2$ $[M+2H]^{2+}/2$, found: 826.426; calculated: 551.286 for $C_{75}H_{121}N_{20}O_{18}S_2$ $[M+2H]^{3+}/3$, found: 551.287.

Peptide Ac-[Cys-Lys-Thr-Leu-Asp-Pro-Lys-Ala-Val-Phe-Gln-Arg-Cys]-metaXylylen-CONH$_2$ (20).

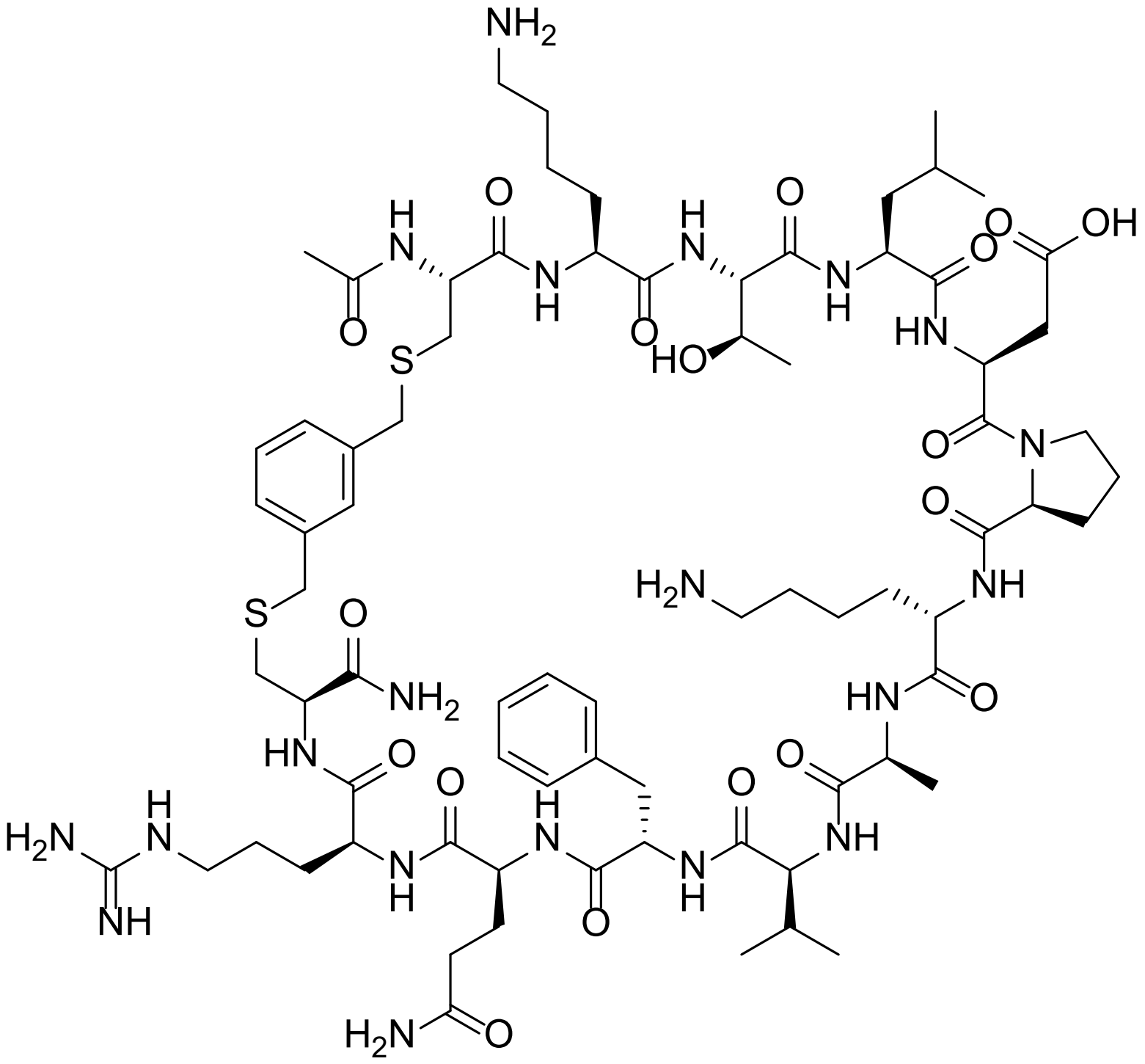

Crude yield: 28 %, purity: 96.06%, t^R 13.387 min, (analytical HPLC, 10 to 90% acetonitrile (0.1% TFA) in water (0.1% TFA) over 20 min, flow rate of 1.0 mL/min); HR-MS (ESI-MS): calculated: 826.426 for $C_{75}H_{120}N_{20}O_{18}S_2$ $[M+2H]^{2+}/2$, found: 826.426; calculated: 551.286 for $C_{75}H_{121}N_{20}O_{18}S_2$ $[M+2H]^{3+}/3$, found: 551.286.

Peptide Ac-[Cys-Lys-Thr-Leu-Asp-Pro-Lys-Ala-Val-Phe-Gln-Arg-Cys]-paraXylylen-CONH$_2$ (21).

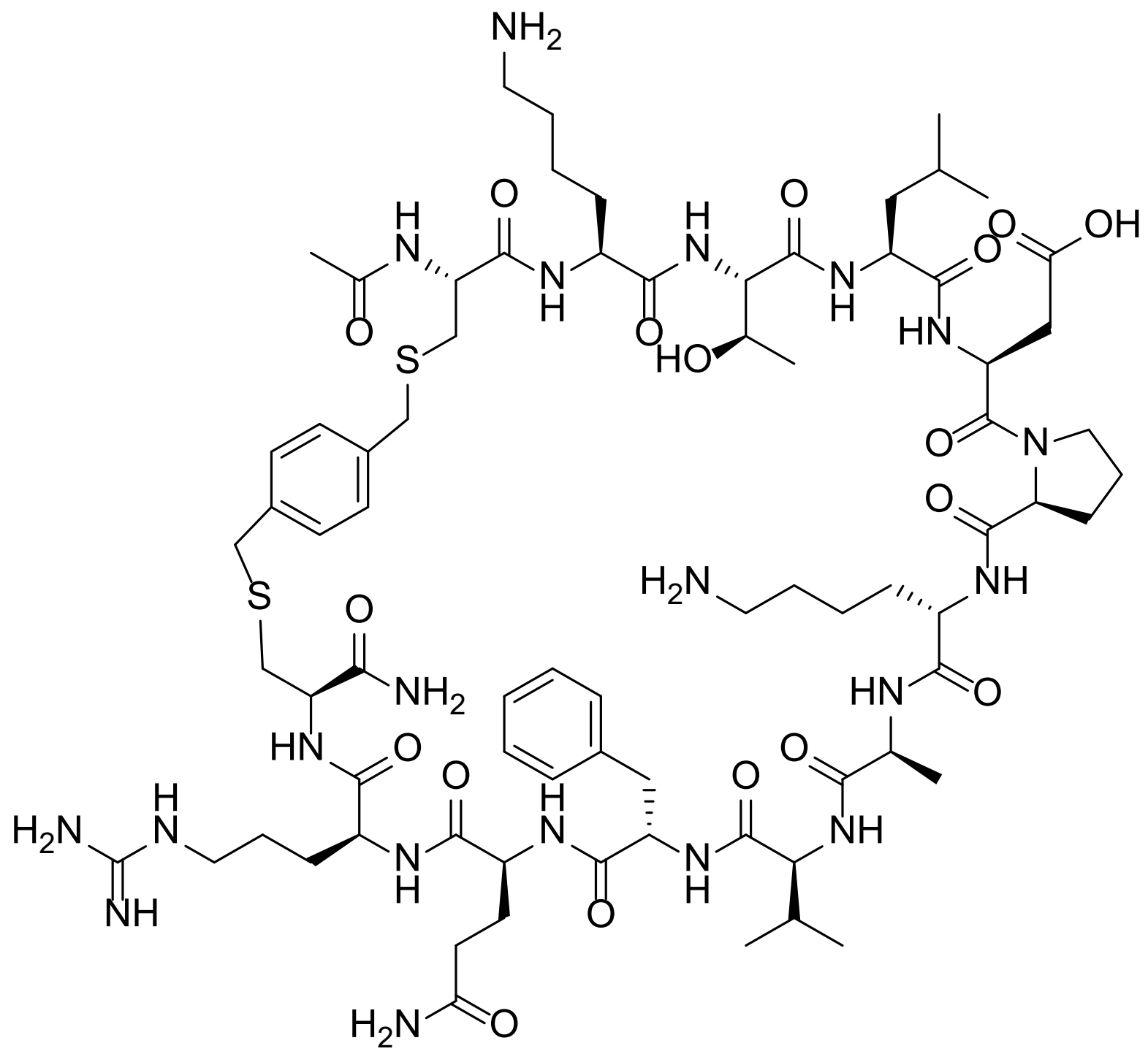

Crude yield: 31 %, purity: 99.55%, t^R 13.353 min, (analytical HPLC, 10 to 90% acetonitrile (0.1% TFA) in water (0.1% TFA) over 20 min, flow rate of 1.0 mL/min); HR-MS (ESI-MS): calculated: 826.426 for $C_{75}H_{120}N_{20}O_{18}S_2$ $[M+2H]^{2+}/2$, found: 826.427; calculated: 551.287 for $C_{75}H_{121}N_{20}O_{18}S_2$ $[M+2H]^{3+}/3$, found: 551.286.

General synthetic protocol for lactam-based cyclic peptides 22-23.

Linear intermediates were assembled on solid support according to the US-SPPS protocol as already stated for peptides **4, 8, 9, 11, 12, 13, 14, R8-14**. On resin cyclization was carried out removing allyl ester protective group from the Glu residue at position 282 of the sequence. The removal was obtained treating the solid support with a mixture of tetrakis(triphenylphosphine)Palladium(0) (12 mg, 0.01 mmol, 0.1 eq relative to the initial loading of the resin) and DMBA (78 mg, 1 mmol, 5 eq relative to the initial loading of the resin) in dry DCM/DMF (1:1, 3 mL) for 90 minutes under argon atmosphere and gentle stirring with a magnetic bar. Afterwards, the resin was rinsed with DMF (3×), DCM (3×), MeOH (3×) and then treated with a solution of piperidine 10% in DMF to remove the N-terminal Fmoc-protective group from glycine (**22**) or γ-aminobutyric acid (**23**). The head-to-side-chain lactam bond was built on resin using a solution of PyAOP (130 mg, 0.25 mmol, 2.5 eq), HOAt (0.1 mmol, 1 eq) and DIPEA (87 µL, 0.5 mmol, 5 eq) in DMF (3 mL) and let it react overnight. Evolution of the reaction was qualitatively determined by Kaiser test and/or HPLC analysis on a small aliquot of resin. After completion of cyclization the resin was rinse with DMF and DCM and then dried until constant weight was obtained. The cyclic peptides were removed from the solid support by treating with TFA/TIS (95:5, 3ml) for 3 hours. The exhausted resin was filtered off, the liquid collected, and crude peptides precipitated by dilution of the cleavage mixture with 30 mL of cold diethyl ether and centrifuged at 6000 rpm for 15 minutes. The supernatant was discarded, and the peptide pellet was washed once more by resuspension in 30 mL of cold diethyl ether and centrifugation under the same conditions. The resulting solid was dried under vacuum.

Purification of crude peptides was performed by semi-preparative reverse-phase high-performance liquid chromatography (RP-HPLC) using solvent A (water + 0.1% TFA) and solvent B (acetonitrile + 0.1% TFA), with a linear gradient from 10% to 60% solvent B over 20 minutes at a flow rate of 4 mL min^{-1}. Fractions of interest were pooled, concentrated to remove organic solvent, frozen, and lyophilized.

Peptide [Gly-Thr-Lys-Thr-Leu-Asp-Pro-Lys-Ala-Val-Phe-Glu]-Arg-Thr-CONH$_2$ (22)

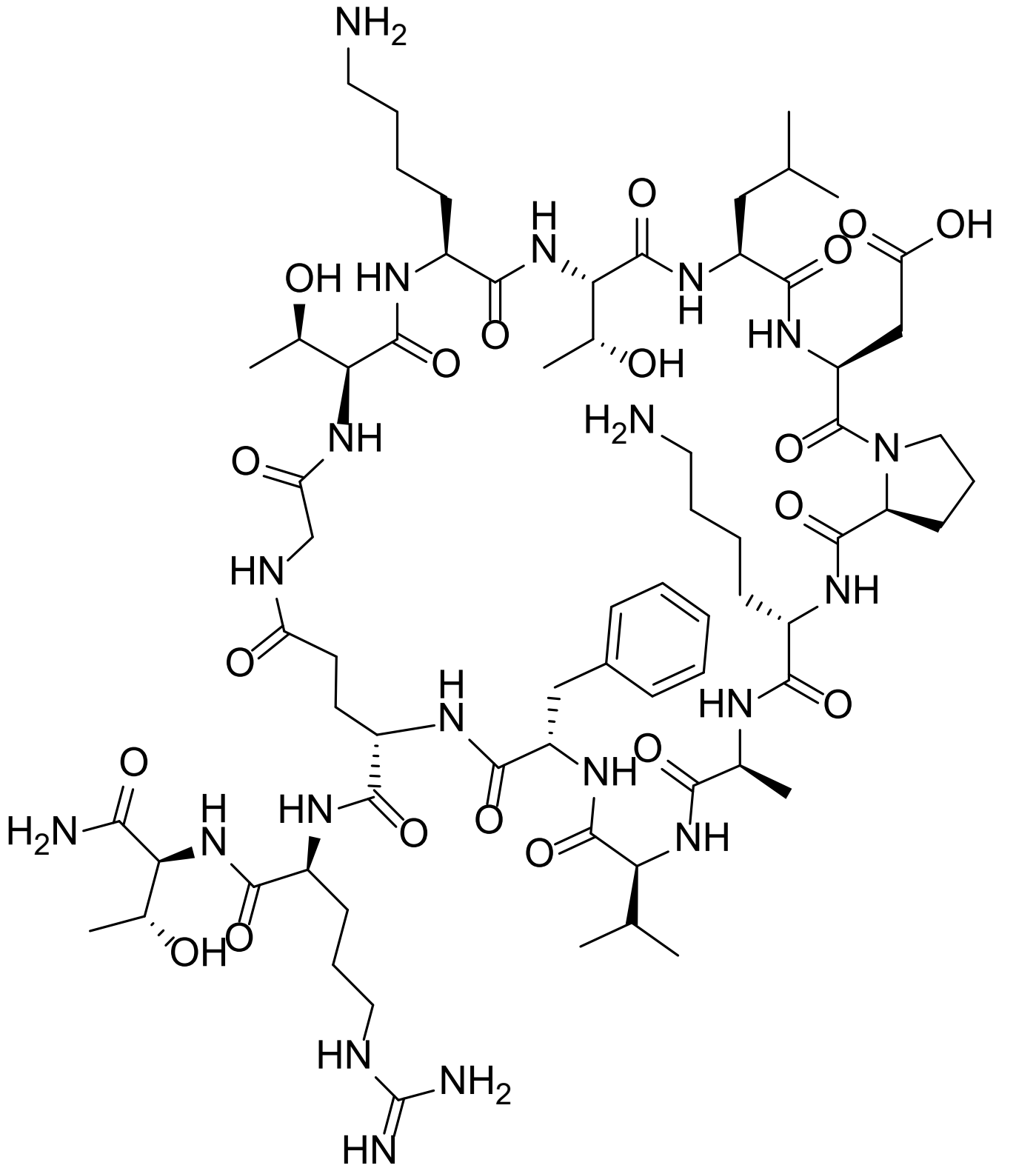

Crude yield: 23 %, purity: 96.56%, t^R 12.597 min, (analytical HPLC, 10 to 90% acetonitrile (0.1% TFA) in water (0.1% TFA) over 20 min, flow rate of 1.0 mL/min); HR-MS (ESI-MS): calculated: 772.434 for $C_{69}H_{116}N_{20}O_{20}$ $[M+2H]^{2+}/2$, found: 772.433; calculated: 515.291 for $C_{69}H_{117}N_{20}O_{20}$ $[M+3H]^{3+}/3$, found: 515.292.

Peptide [GABA-Thr-Lys-Thr-Leu-Asp-Pro-Lys-Ala-Val-Phe-Glu]-Arg-Thr-CONH₂ (23)

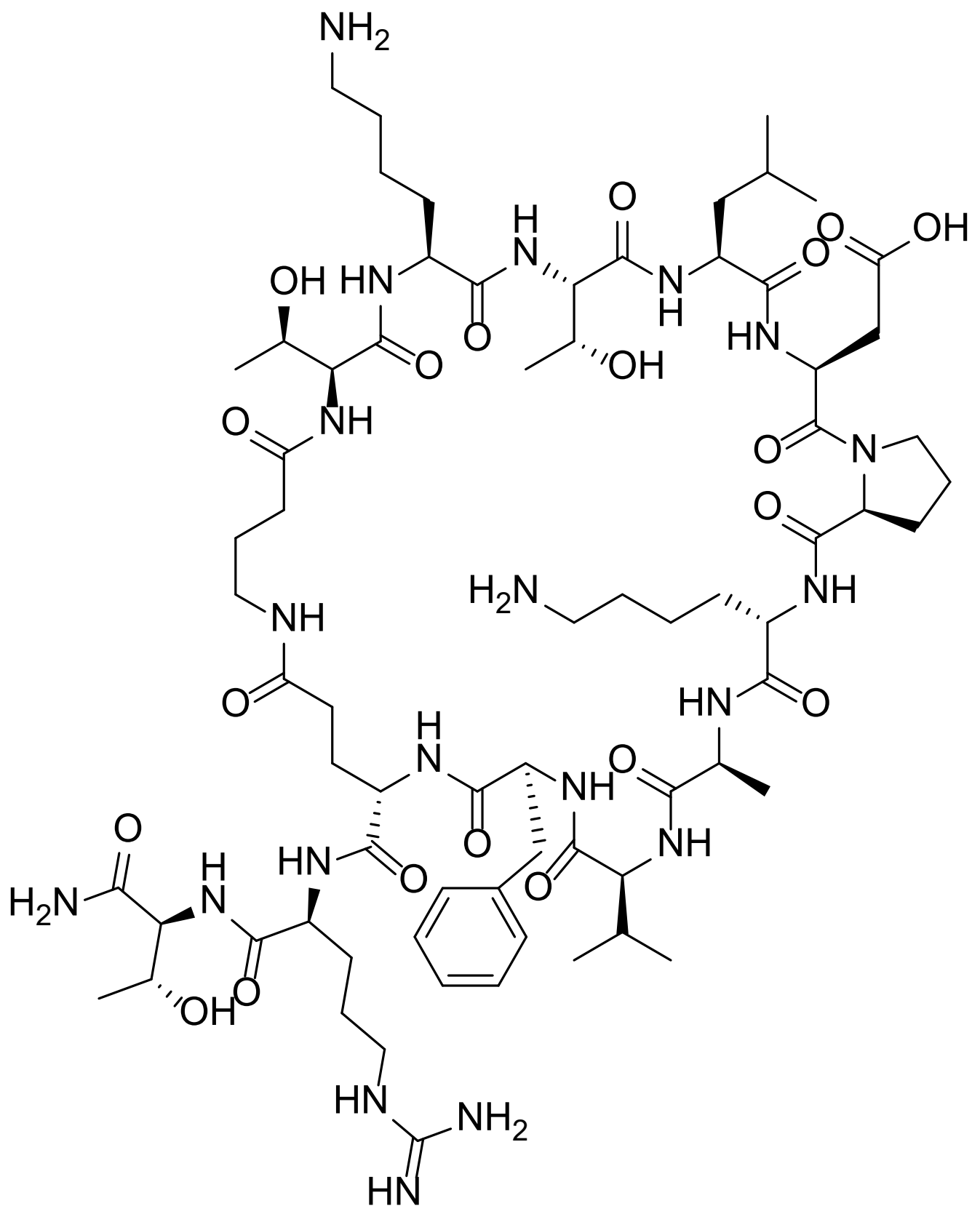

Crude yield: 28 %, purity: 98.81%, t^R 12.727 min, (analytical HPLC, 10 to 90% acetonitrile (0.1% TFA) in water (0.1% TFA) over 20 min, flow rate of 1.0 mL/min); HR-MS (ESI-MS): calculated: 786.449 for $C_{71}H_{120}N_{20}O_{20}$ $[M+H]^{2+}/2$ found: 786.452; calculated: 524.635 for $C_{71}H_{121}N_{20}O_{20}$ $[M+3H]^{3+}/3$ found 524.638.

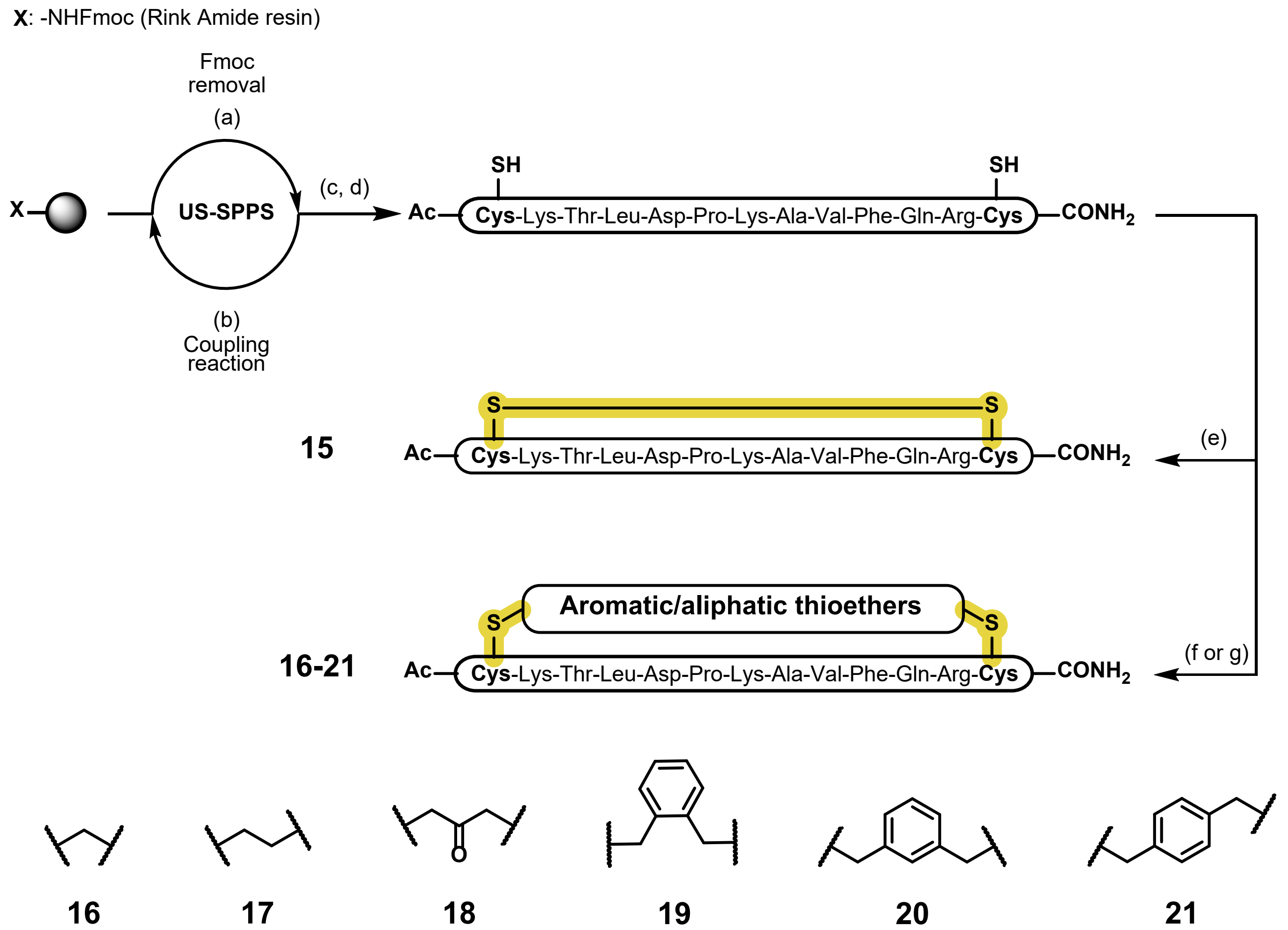

Scheme S1. Synthetic scheme of peptides **15-21**. Reagent and conditions: (a) Piperidine solution 10% in DMF, 2 × 1 min US irradiation; (b) Fmoc-AA-OH, HBTU, HOBt, DIPEA, DMF, 5 min, US irradiation; (c) Ac_2O, DIPEA, DMF, 10 min; (d) TFA, TIS, *D*,*L*-dithiothreitol (95:2.5:2.5), 3 h; (e) Ammonium carbonate 0.1M; (f) Aliphatic dibromide, TCEP, TEA, THF/water (1:1); (g) *para*-, *meta*-, or *ortho*-xylylene dibromide, TCEP, K_2HPO_4 0.5 M in water/CH_3CN (1:1).

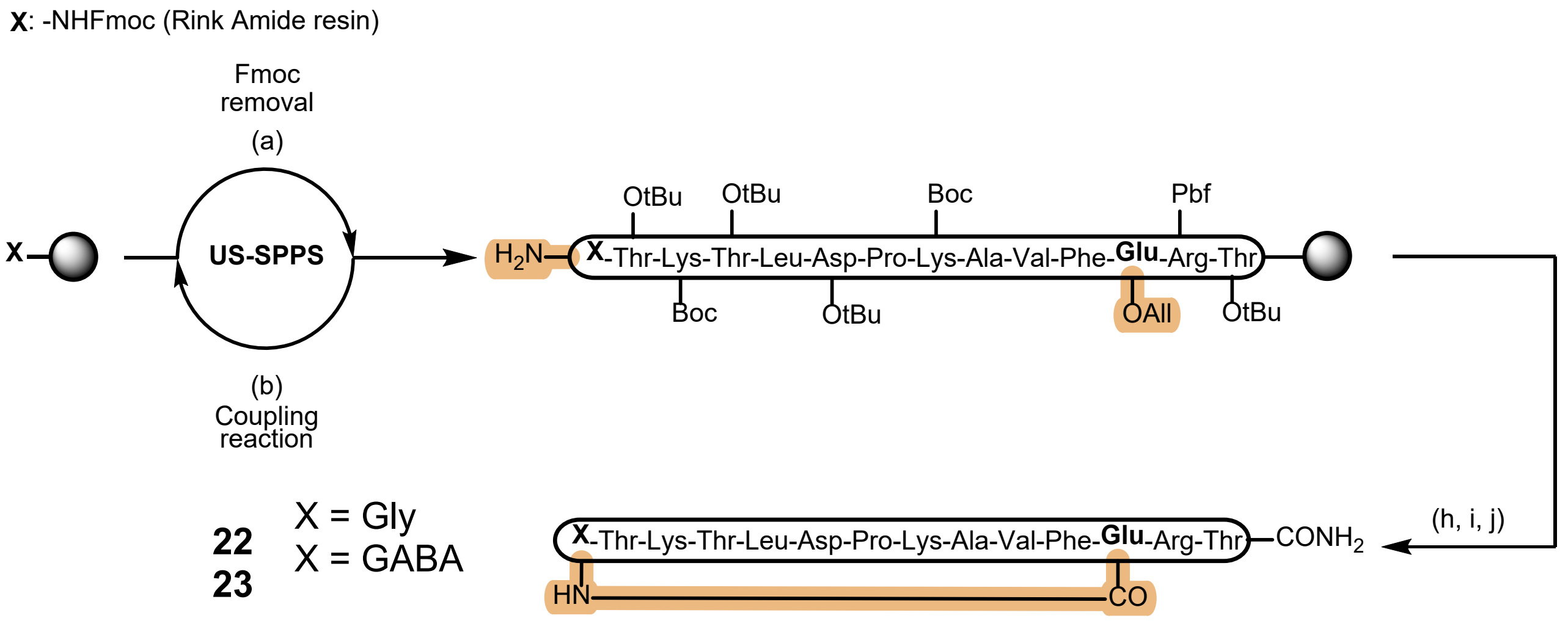

Scheme S2. Synthetic scheme of peptides **22-23**. Reagent and conditions: (a) Piperidine solution 10% in DMF, 2 × 1 min US irradiation; (b) Fmoc-AA-OH, HBTU, HOBt, DIPEA, DMF, 5 min, US irradiation (h) Tetrakis-triphenylphosphine Palladium (0), DMBA, DCM/DMF dry (1:1), 2 × 90 min; (i) PyAOP, HOAt, DIPEA, DMF dry, 16h; (j) TFA, TIS (95:5), 3h.

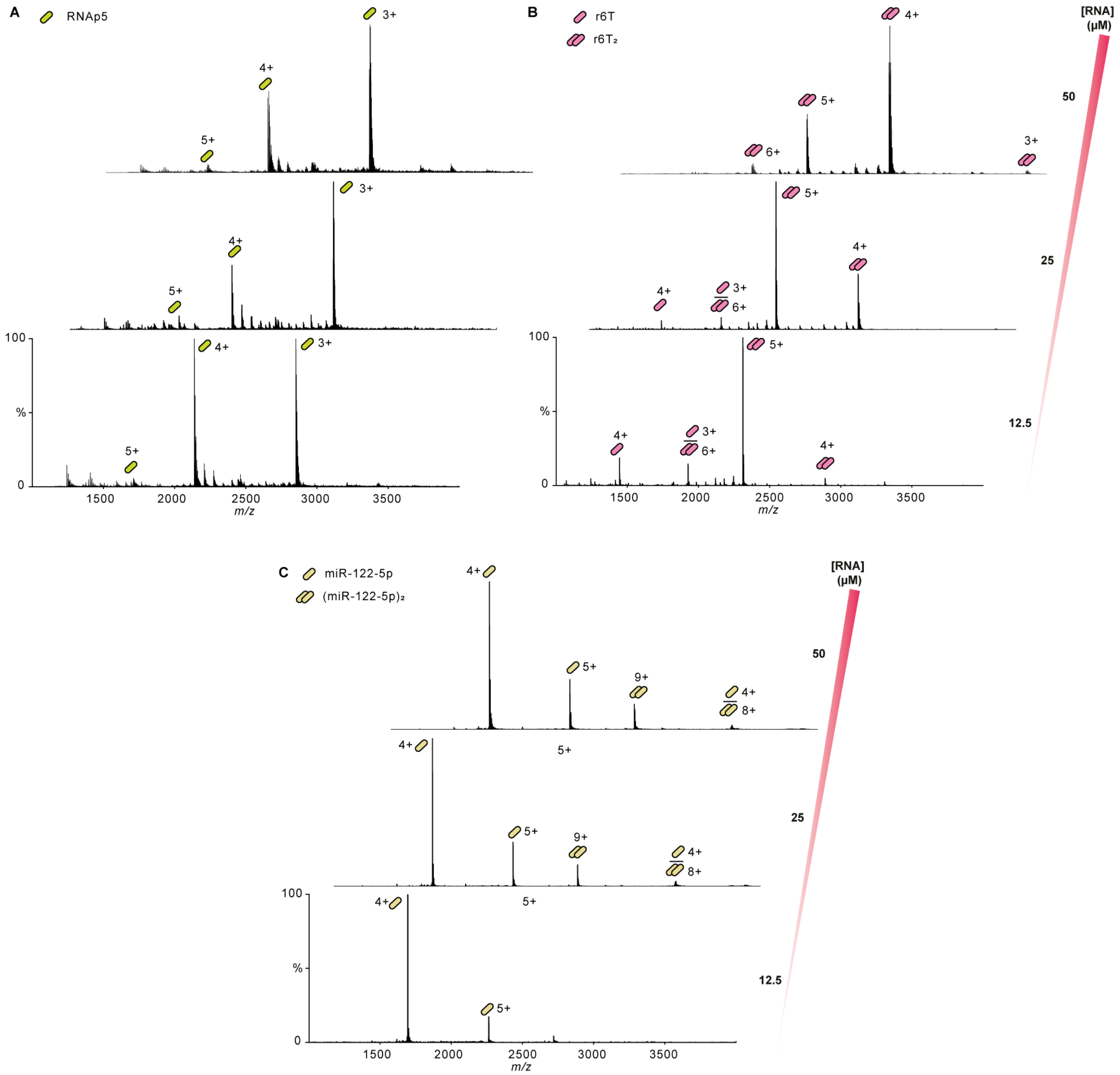

Figure S1. Native MS characterization of RNA self-association across a concentration series. A) RNAp5: charge-state envelopes for the monomer are observed at all concentrations tested. B) r6T: duplex charge-state envelopes dominate across the concentration series, with <5% monomer present at 12.5 µM. C) miR-122-5p: monomer charge-state envelopes dominate at all concentrations tested, with <5% duplex detected at 12.5 µM. Low-abundance signals accompanying the main charge states correspond to minor non-covalent adducts of the RNA, commonly observed for oligonucleotides in native ESI-MS owing to their polyanionic character.

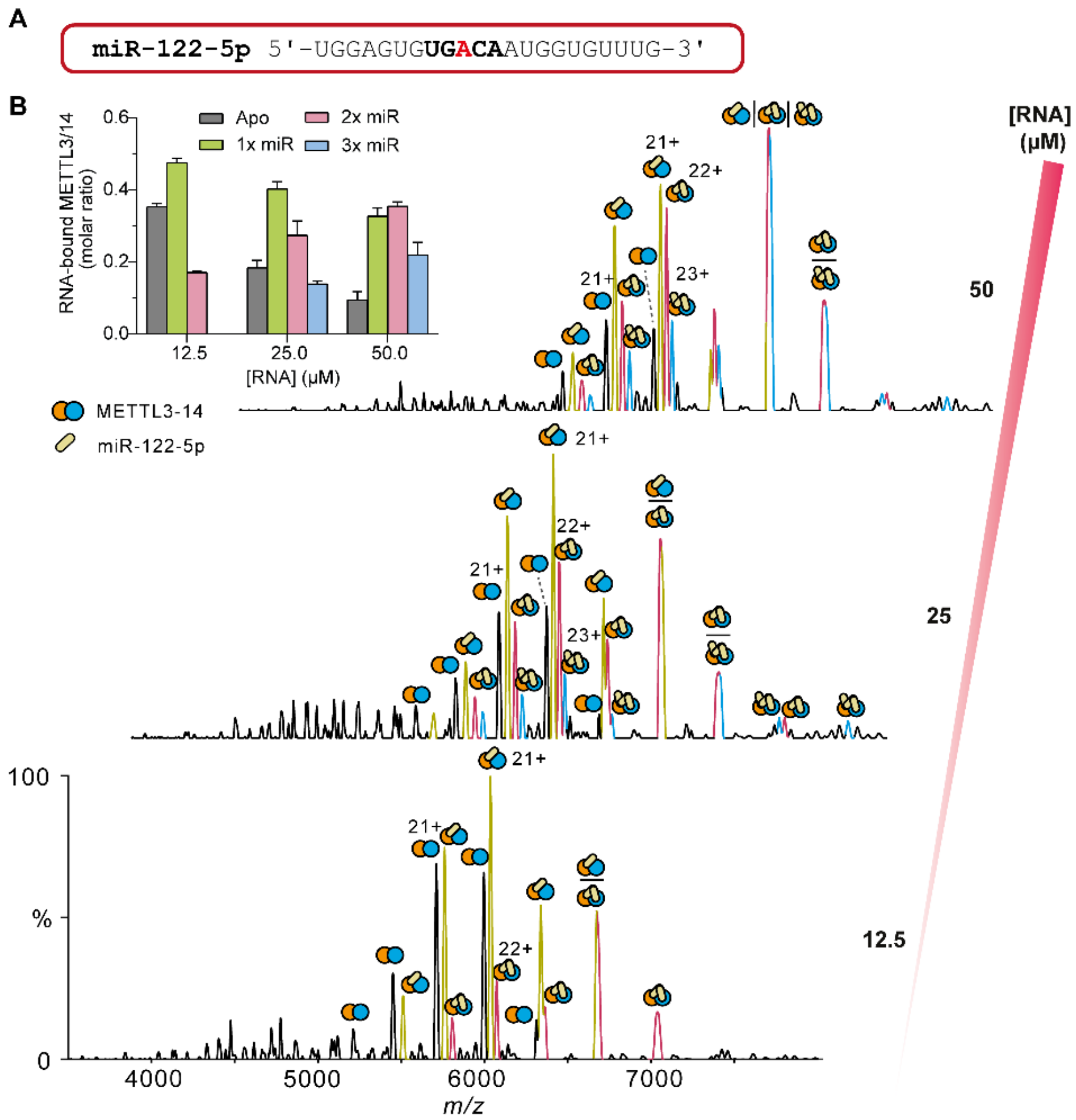

Figure S2. A) Sequence of miR-122-5p. B) Native mass spectra of METTL3-METTL14 (2.5 μM) in the presence of increasing concentrations of miR-122-5p. Charge state series corresponding to RNA-bound complexes appear in a concentration-dependent manner. Bar charts report the relative abundances of METTL3–METTL14 bound to one, two, or three RNA molecules, as determined from the assigned charge-state series. The quantification shows a consistent shift toward the two-RNA–bound species at the tested concentrations. Data are expressed as mean ± standard deviation (SD) of three biological replicates (n = 3).

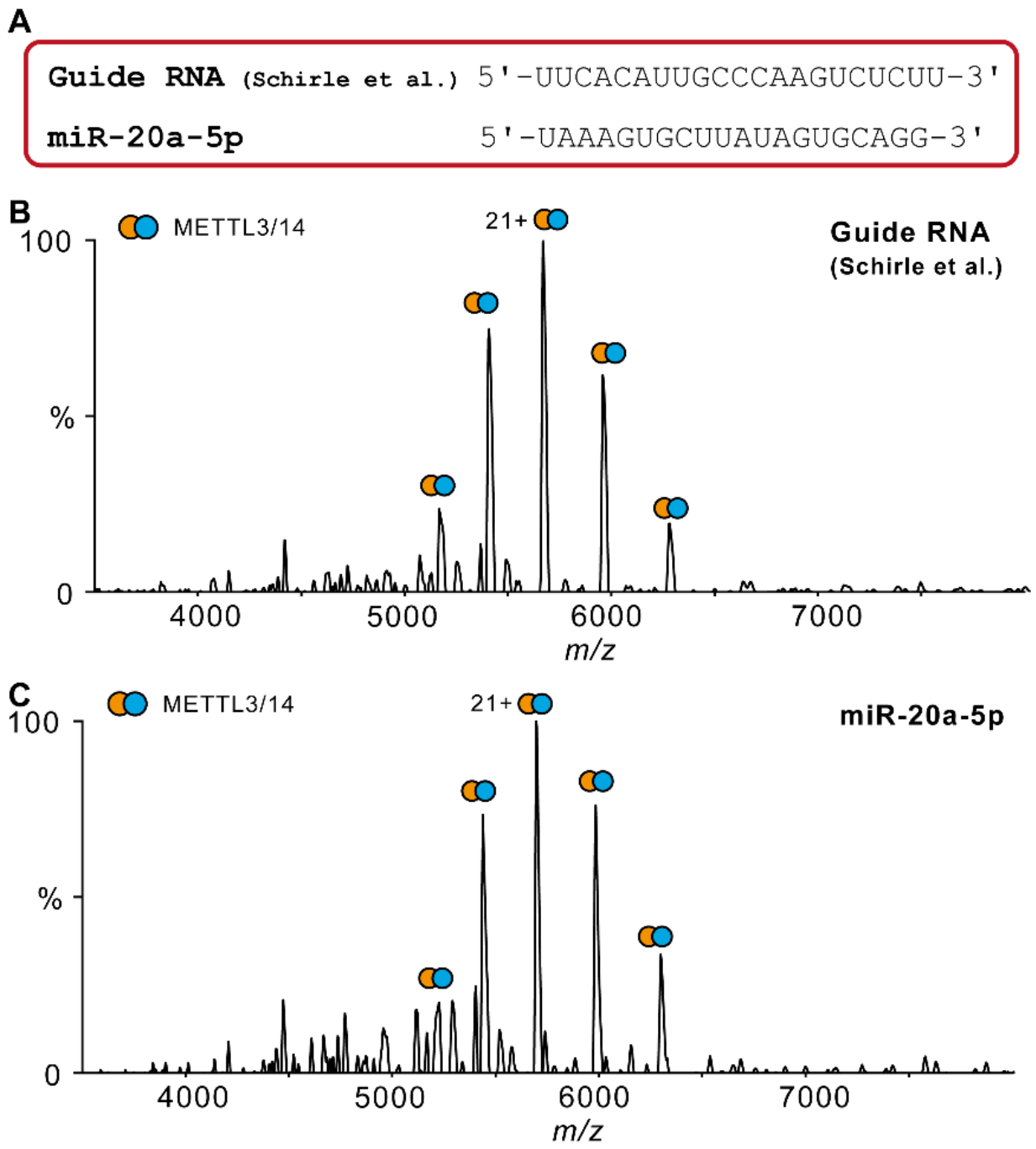

Figure S3. A) Sequences of AGO2 guide RNA reported by Schirle et al.[14-15] and miR-20a-5p. B, C) Native mass spectra of METTL3-METTL14 (2.5 µM) in the presence of the synthetic guide RNA (B) and miR-20a-5p (C) at a concentration of 25 µM. No binding was detected.

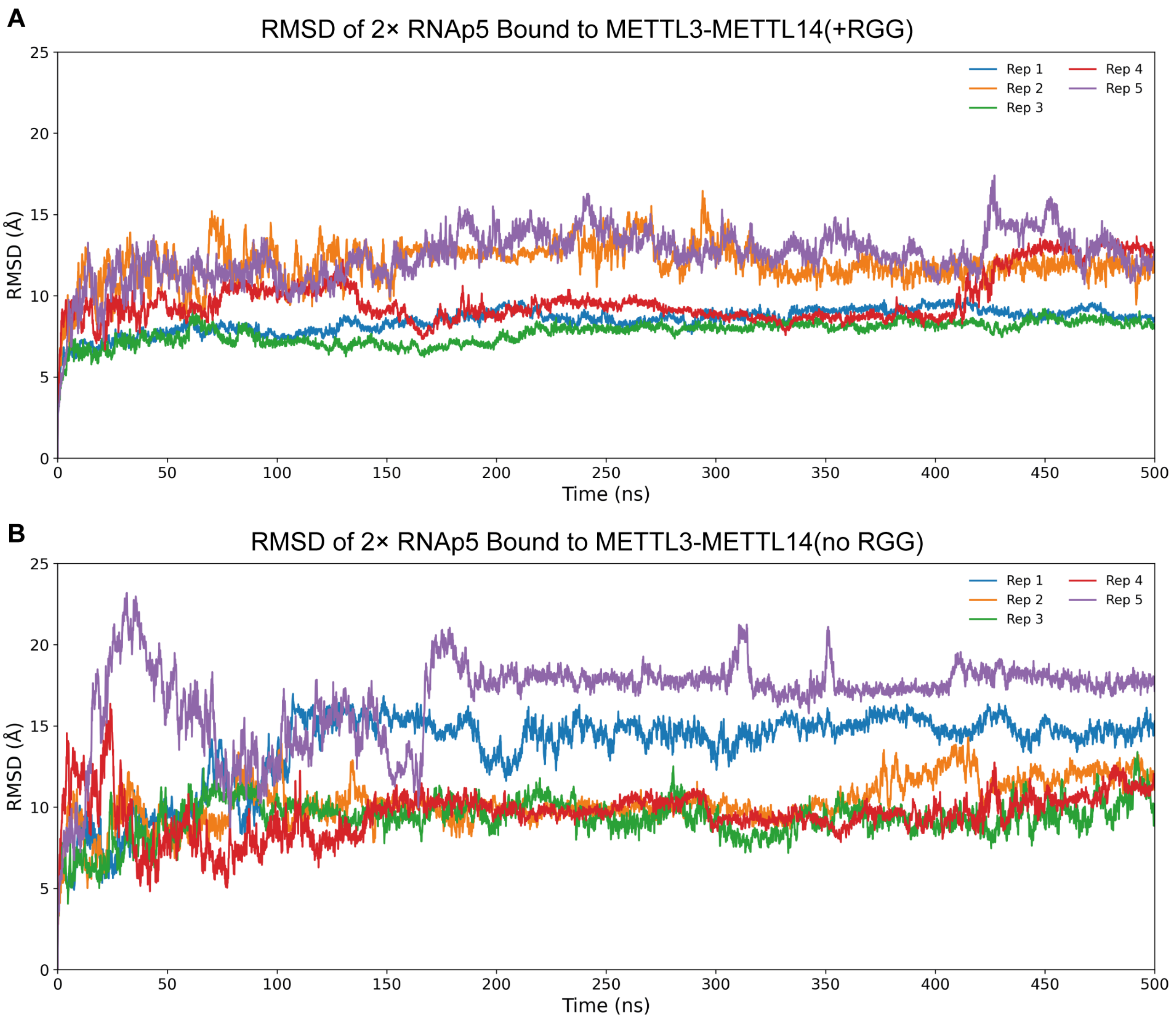

Figure S4. MD analysis of the METTL3-METTL14/RNAp5 complex. A) RMSD of two RNAp5 molecules bound to METTL3-METTL14 in the presence of the RGG motif, calculated after fitting the trajectory to the protein backbone. Each trace represents an independent MD repeat. B) Equivalent analysis for the complex lacking the RGG motif.

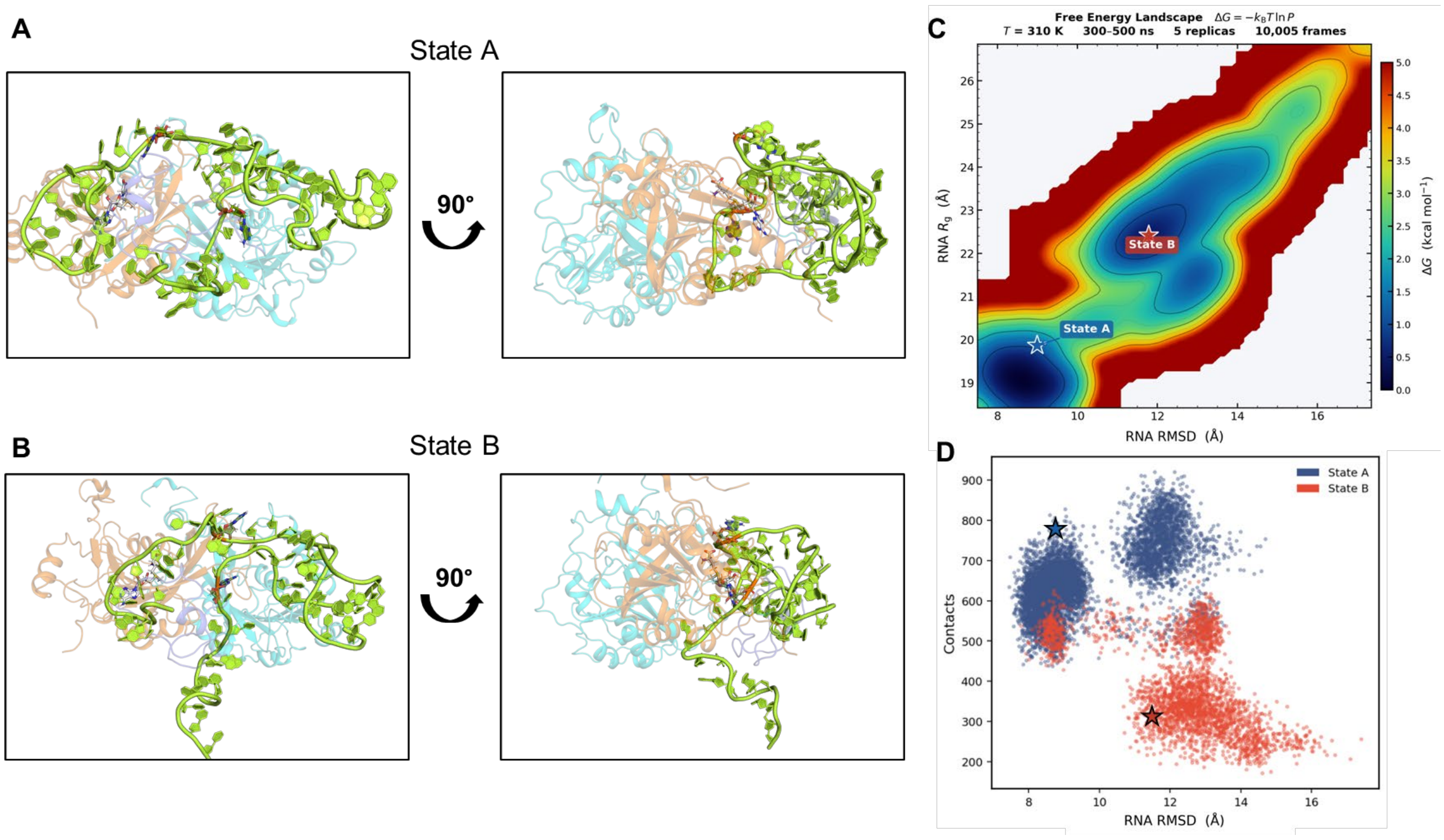

Figure S5. Conformational clustering analysis of the MD trajectories for the RGG-containing METTL3-METTL14-RNAp5 complex. A) Representative structures of State A shown in two orientations. B) Representative structures of State B shown in two orientations. METTL3 is shown in orange, METTL14 in cyan, the RGG-containing RNA-bound region in green, and SAM as sticks. C) Approximate free-energy landscape projected onto RNA RMSD and RNA radius of gyration, showing two populated minima corresponding to States A and B. D) Scatter plot of the MD frames projected onto RNA RMSD and protein-RNA contacts, highlighting the two clustered states.

A

B

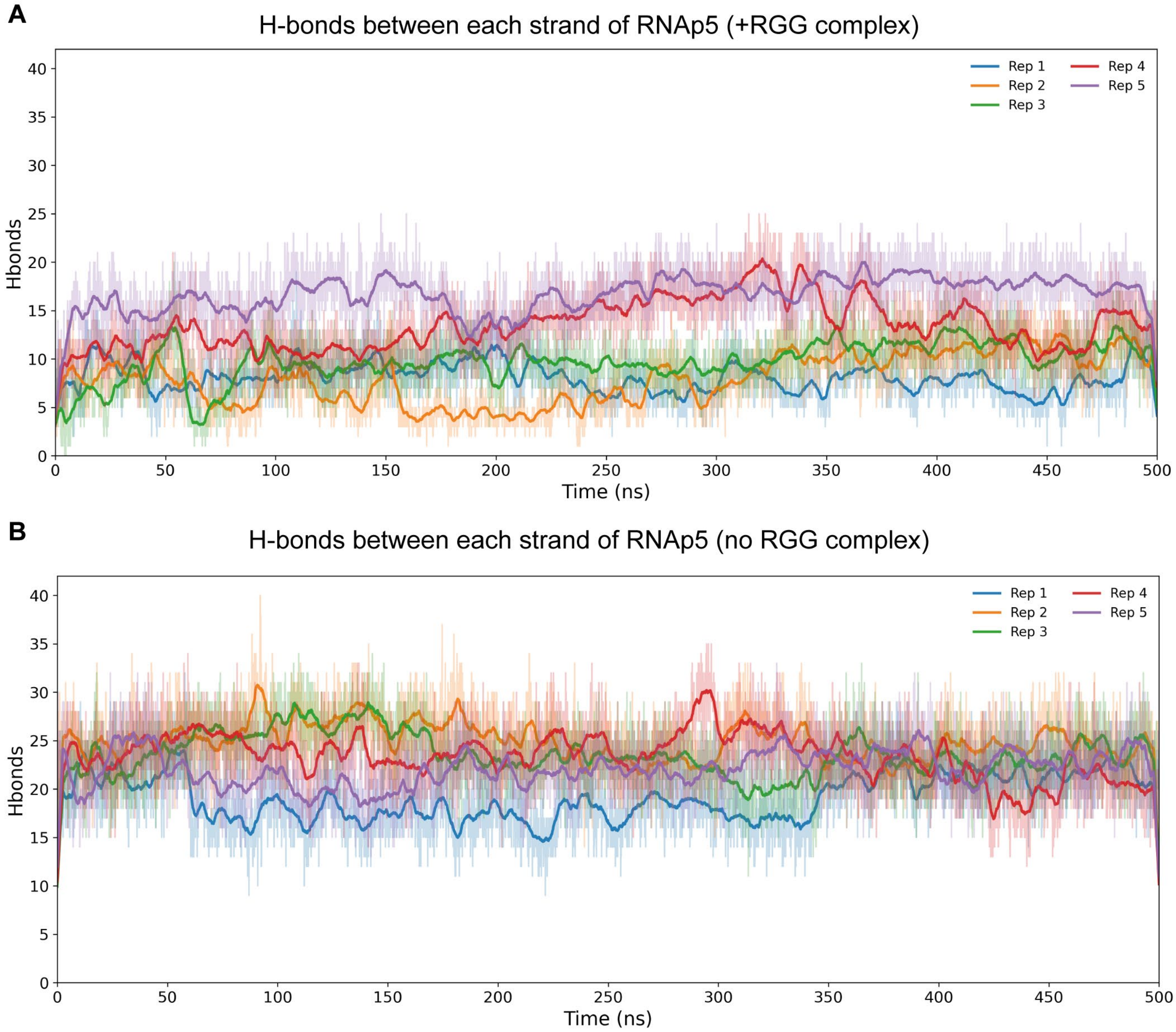

Figure S6. A) Number of inter-strand RNAp5 hydrogen bonds throughout the MD simulations for the METTL3-METTL14 complex containing the RGG motif. B) Corresponding analysis for the complex lacking the RGG motif.

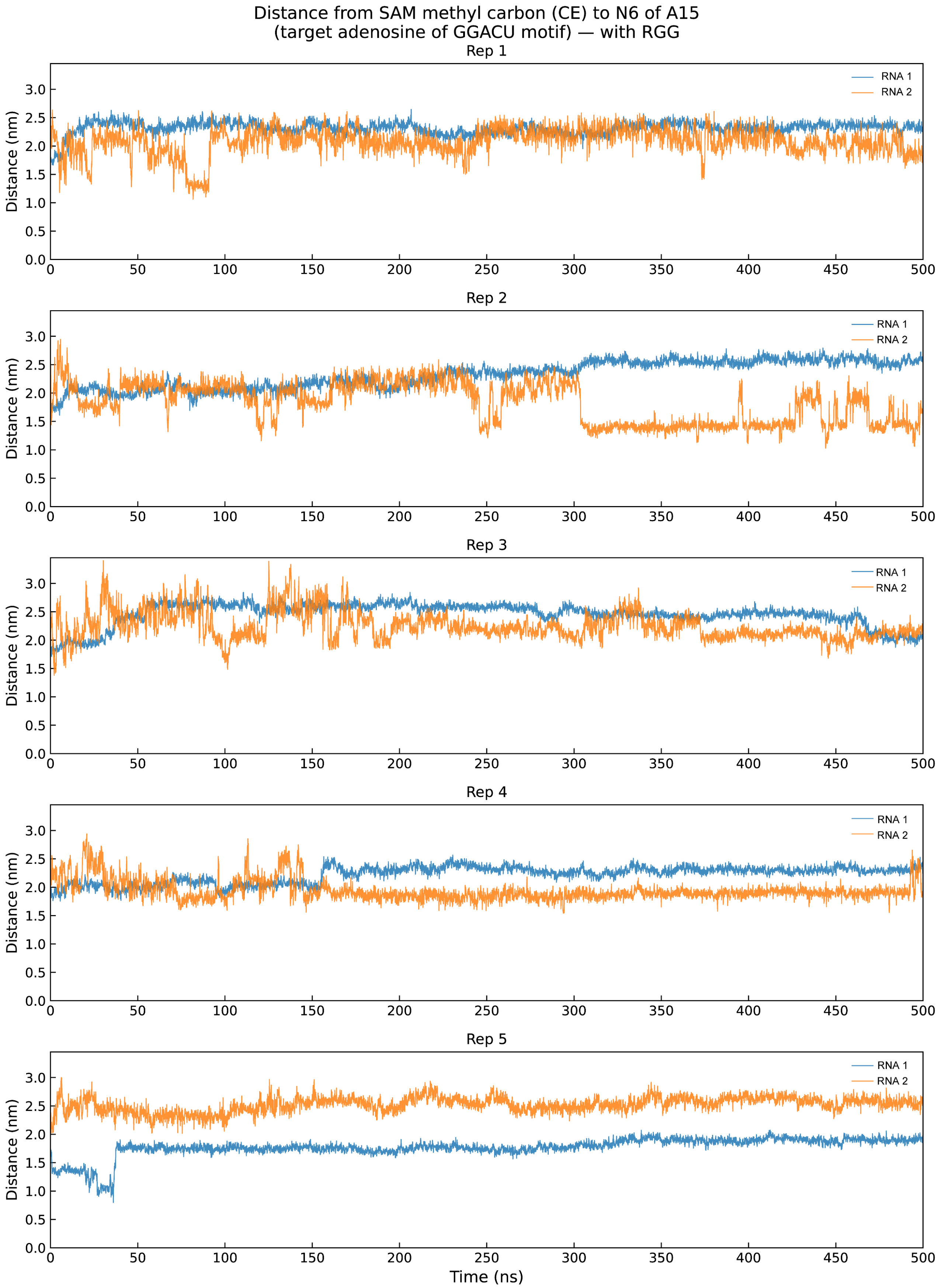

Figure S7. Distances between the SAM methyl carbon and the N6 atom of the target adenosine (A15) of RNAp5 during the MD simulations of the RGG-containing complex.

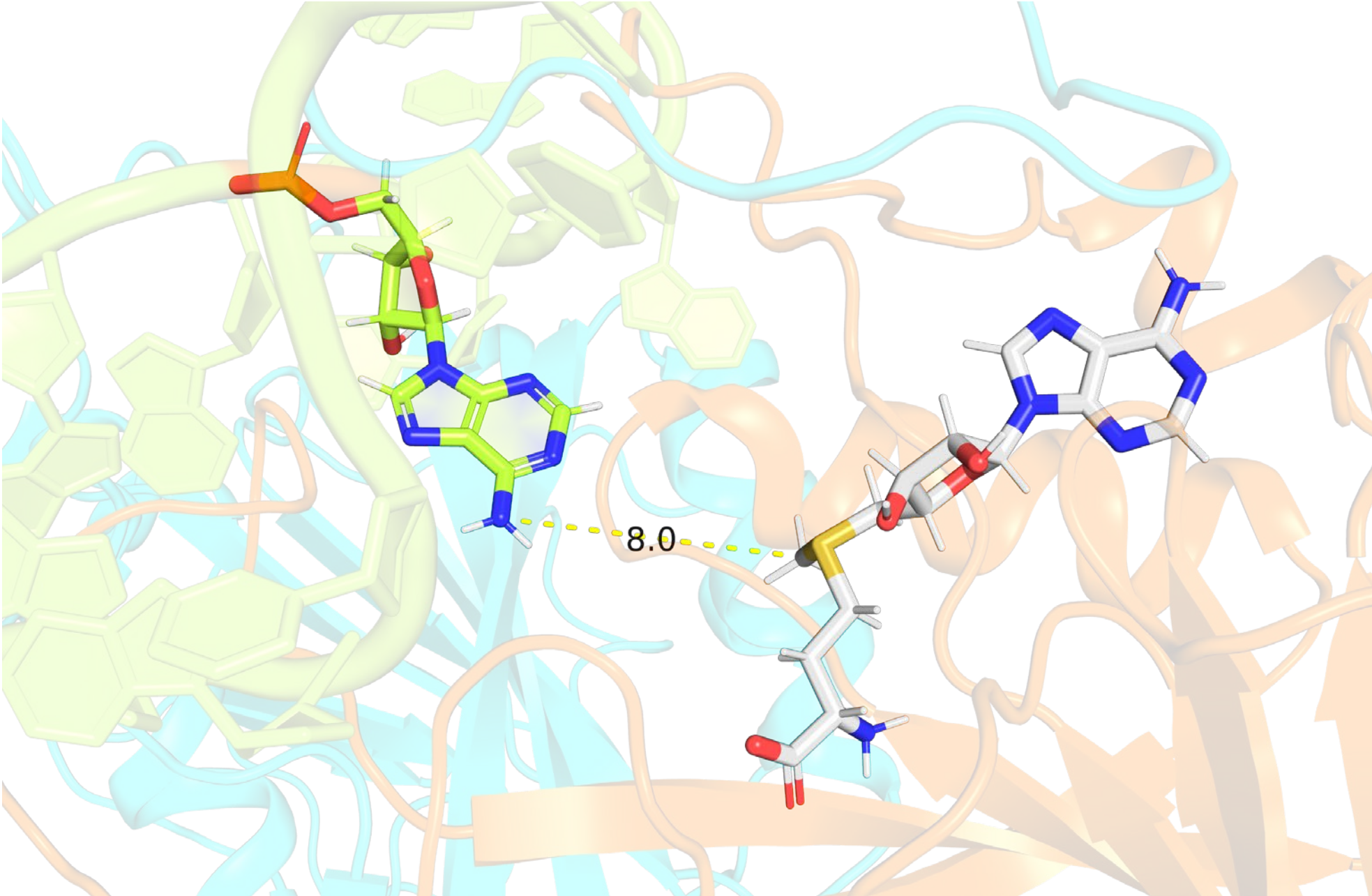

Figure S8. Representative pose showing the shortest distance sampled between the SAM methyl group and the N6 atom of the target RNAp5 adenosine during the MD simulations.

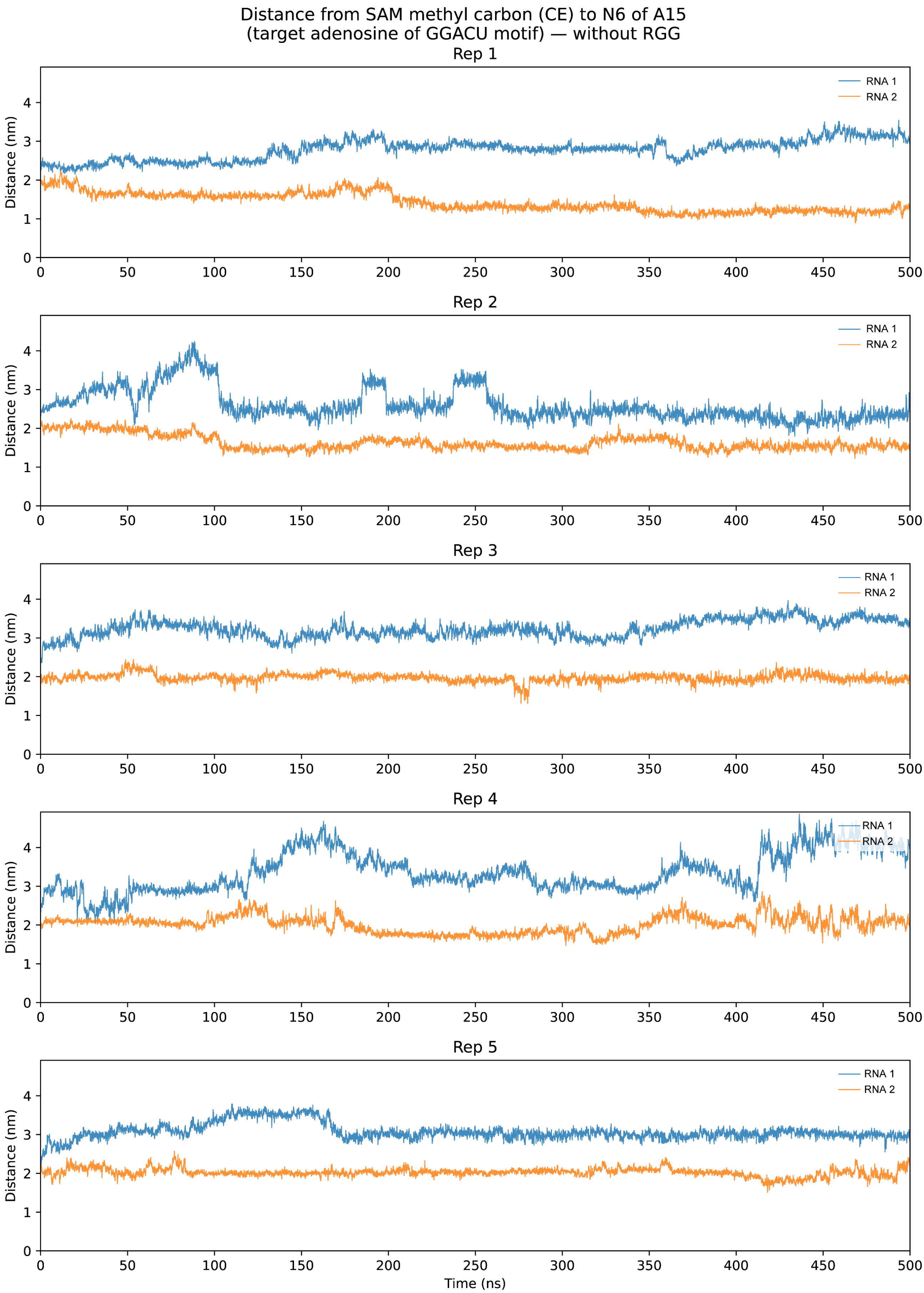

Figure S9. Distances between the SAM methyl carbon and the N6 atom of the target adenosine (A15) of RNAp5 during the MD simulations of the RGG-lacking complex.

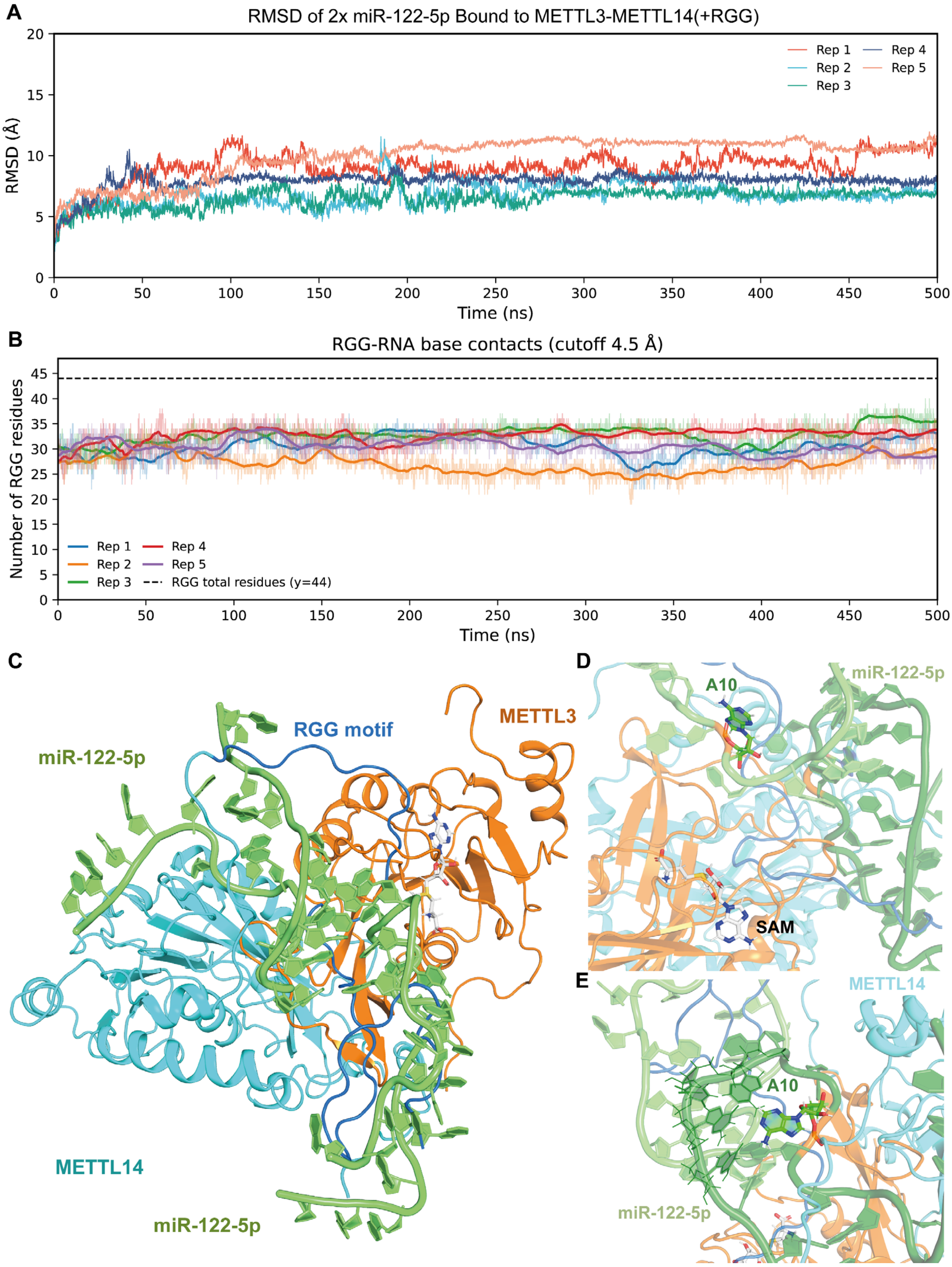

Figure S10. AI and molecular dynamics of the METTL3-METTL14/miR-122-5p complex. A) RMSD of two miR-122-5p molecules bound to METTL3-METTL14 in the presence of the RGG motif, calculated after fitting the trajectory to the protein backbone. Each trace represents an independent MD repeat. B) Number of contacts formed between the RGG motif and the miR-122-5p molecules throughout the MD simulations. C) Structural model of the METTL3-METTL14 complex predicted by Chai-1, shown with METTL3 in orange, METTL14 in cyan, the RGG motif in blue, and bound miR-122-5p in lime. D) Close-up view of the miR-122-5p strand located closer to the SAM pocket, in which the target A remains solvent-accessible. E) Close-up view of the more distal miR-122-5p strand, showing intra-strand base pairing and partial occlusion of the target motif.

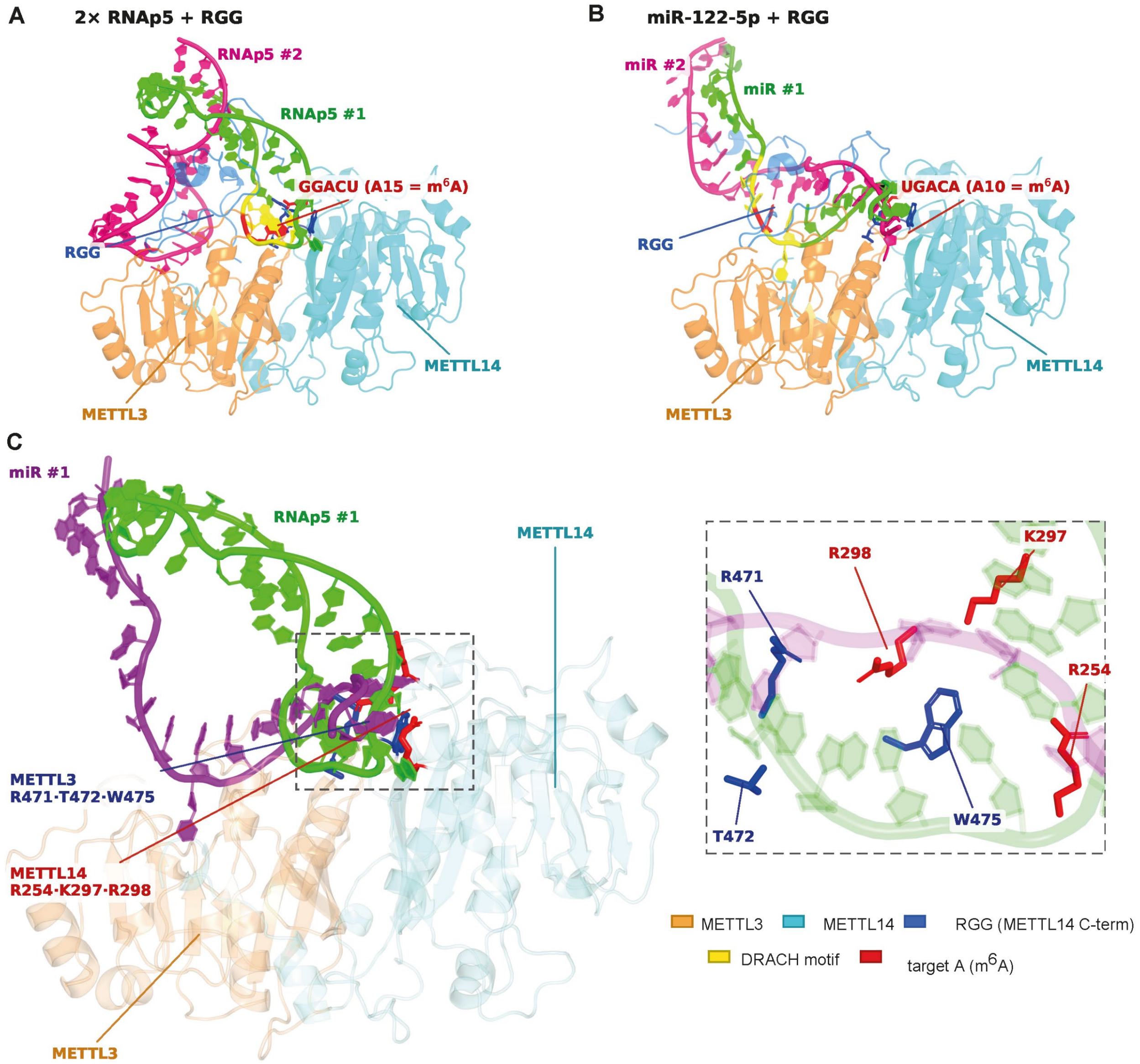

Figure S11. Comparative interaction patterns of RNAp5 and miR-122-5p in the 2× RNA-bound METTL3–METTL14 (+ RGG motif). A,B) Representative structural models of METTL3–METTL14 bound to two RNAp5 (A) or two miR-122-5p molecules (B). The DRACH-containing segments are highlighted in yellow, with the target adenosines A15 in RNAp5 and A10 in miR-122-5p shown in red. The RNA substrates engage different portions of the flexible RGG region. In the RNAp5 complex, the target-motif-containing segment is contacted predominantly by the C-terminal RGG residues R450, F453, and R456, whereas in the miR-122-5p complex the equivalent region interacts mainly with central RGG residues R431, F434, R435, R442, and R445. C) Structural superposition of RNAp5 strand 1 and miR-122-5p strand 1 highlighting the conserved interaction surface at the METTL3–METTL14 interface. Both RNAs contact METTL3 residues R471, T472, and W475 and METTL14 residues R254, K297, and R298. R471, K297, and R298 predominantly form electrostatic and hydrogen-bonding interactions with the RNA phosphate backbone, while T472, K297, and R298 can also contact ribose 2′-hydroxyl groups. R471 and R298 may additionally interact with guanine base edges. R254 contributes cation–π or stacking interactions with an adenine base, whereas W475 forms a hydrophobic contact with the RNA edge. The inset provides an enlarged view of these conserved protein–RNA contacts.

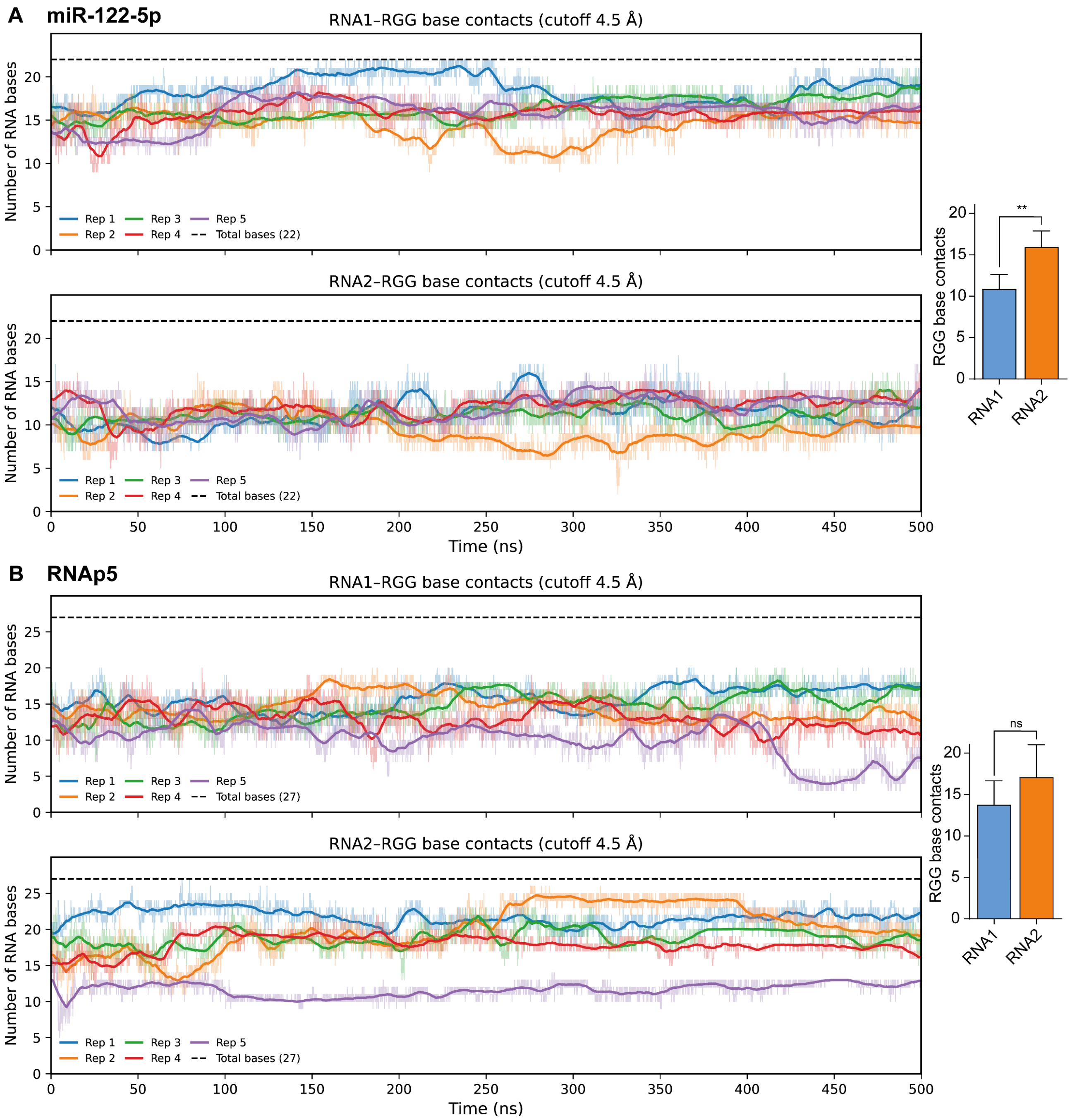

Figure S12. Base-level analysis of RGG motif-RNA contacts in METTL3-METTL14 complexes containing two miR-122-5p or two RNAp5 molecules. A) Number of miR-122-5p bases in contact with the METTL14 RGG motif during MD simulations, shown separately for RNA1 and RNA2 with a 4.5 Å cutoff. Each trace represents an independent MD replicate. B) Corresponding analysis for the METTL3-METTL14 complex containing two RNAp5 molecules, again shown separately for RNA1 and RNA2. Dashed lines indicate the total number of bases in each RNA molecule. The bar charts indicate the average number of RGG base contacts for each RNA molecules ± SD of five replicates ($n = 5$). Statistical significance was assessed using a two-tailed Welch's t-test (ns, non-significant; $^{**}p < 0.01$).

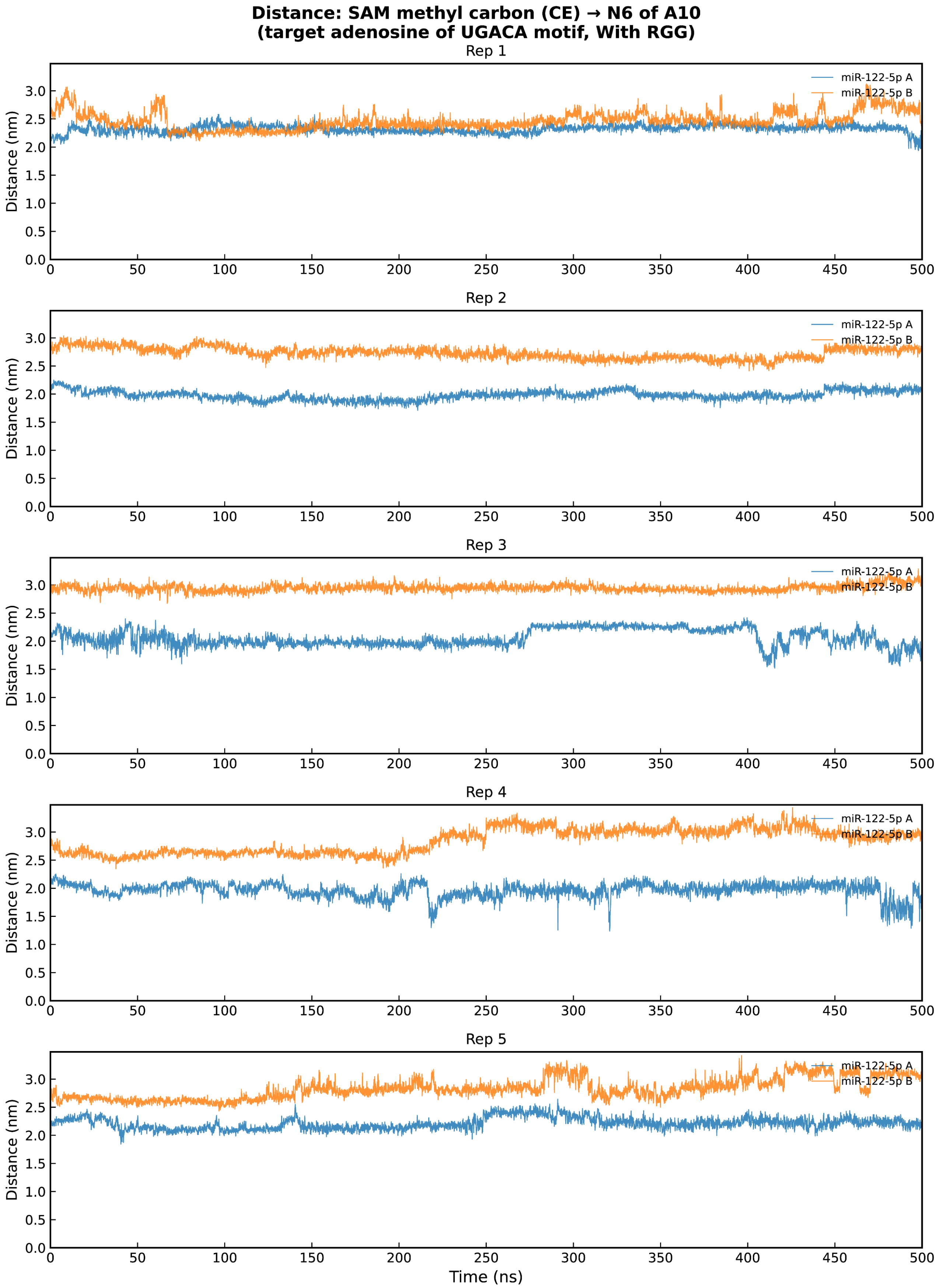

Figure S13. Distances between the SAM methyl carbon and the N6 atom of the target adenosine (A10) of miR-122-5p during the MD simulations of the RGG-containing complex.

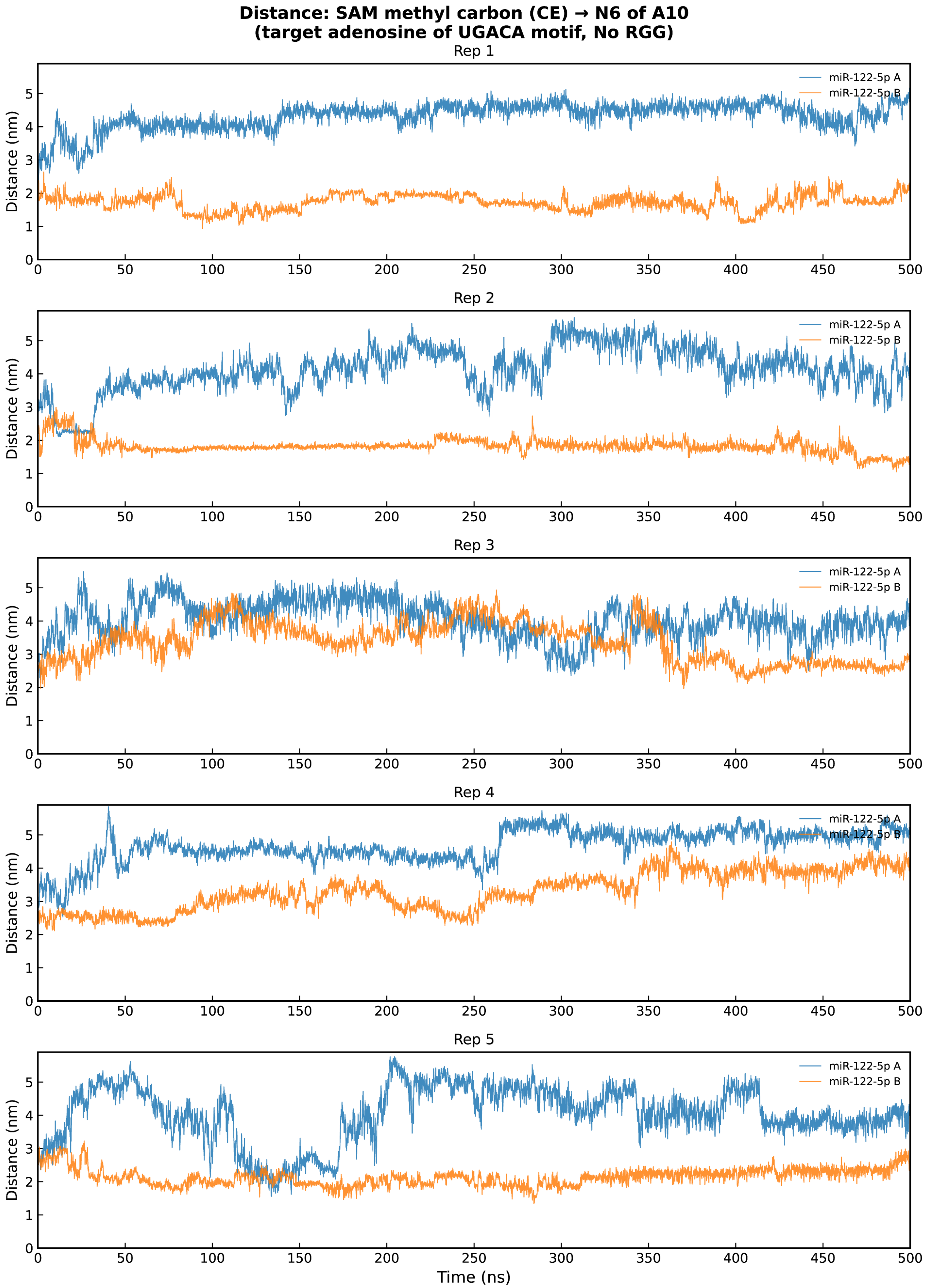

Figure S14. Distances between the SAM methyl carbon and the N6 atom of the target adenosine (A10) of miR-122-5p during the MD simulations of the RGG-lacking complex.

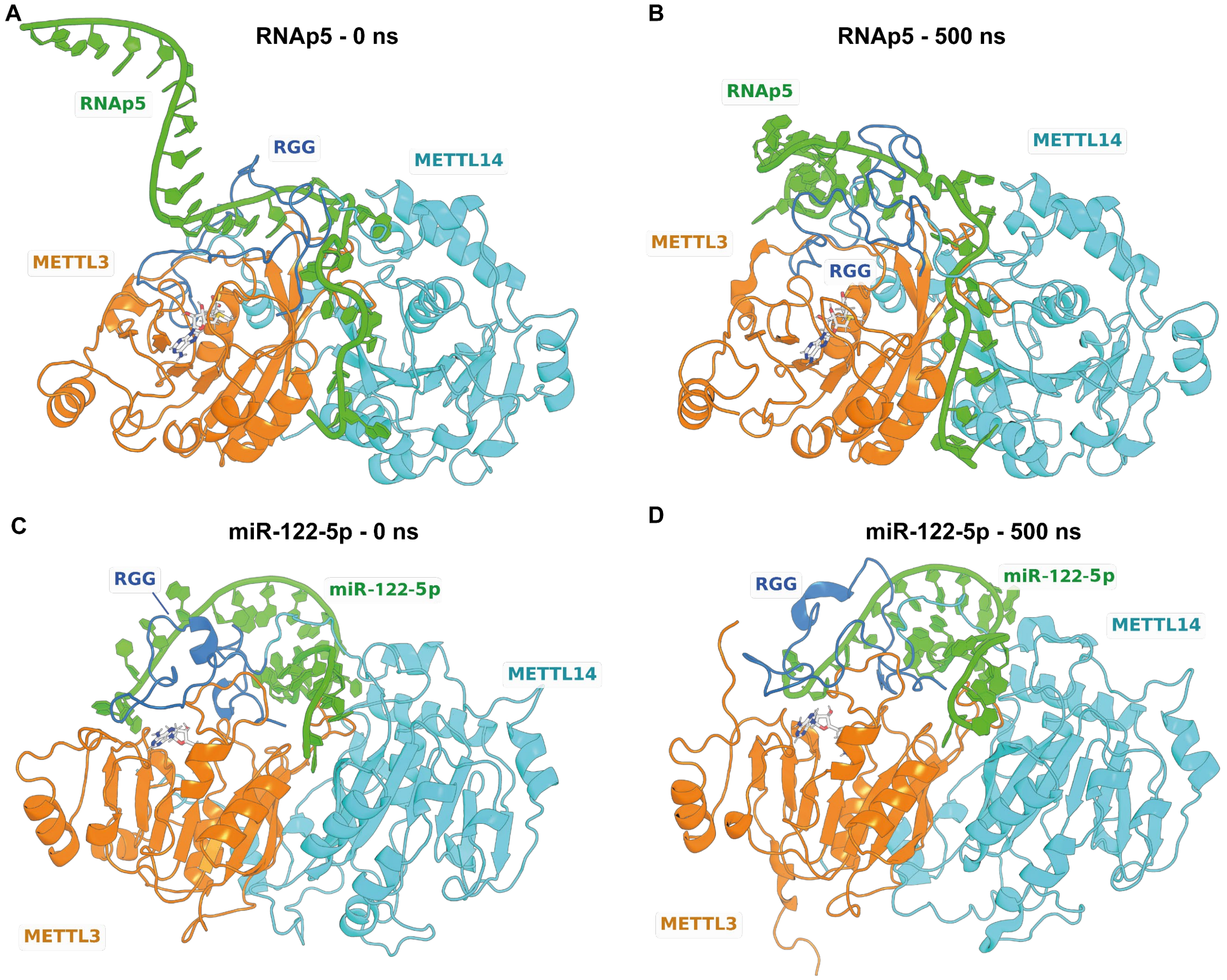

Figure S15. Representative structures of the 1×RNA-bound METTL3–METTL14 (+RGG) complexes. Snapshots from MD simulations of METTL3–METTL14 bound to a single RNA molecule: RNAp5 at 0 ns (A) and 500 ns (B), and miR-122-5p at 0 ns (C) and 500 ns (D). METTL3 is shown in orange, METTL14 in cyan, the METTL14 RGG region in blue, and the RNA in green; the ligand in the METTL3 active site is shown as sticks. During the simulations, RNAp5 remained comparatively extended, with one region retained within the METTL3–METTL14 groove and the 3′ region progressively engaging the RGG motif. By contrast, miR-122-5p maintained a more compact groove-associated conformation while redistributing part of its interactions toward the RGG region. These substrate-specific rearrangements illustrate how the interfacial groove and the RGG motif form a composite RNA-binding surface.

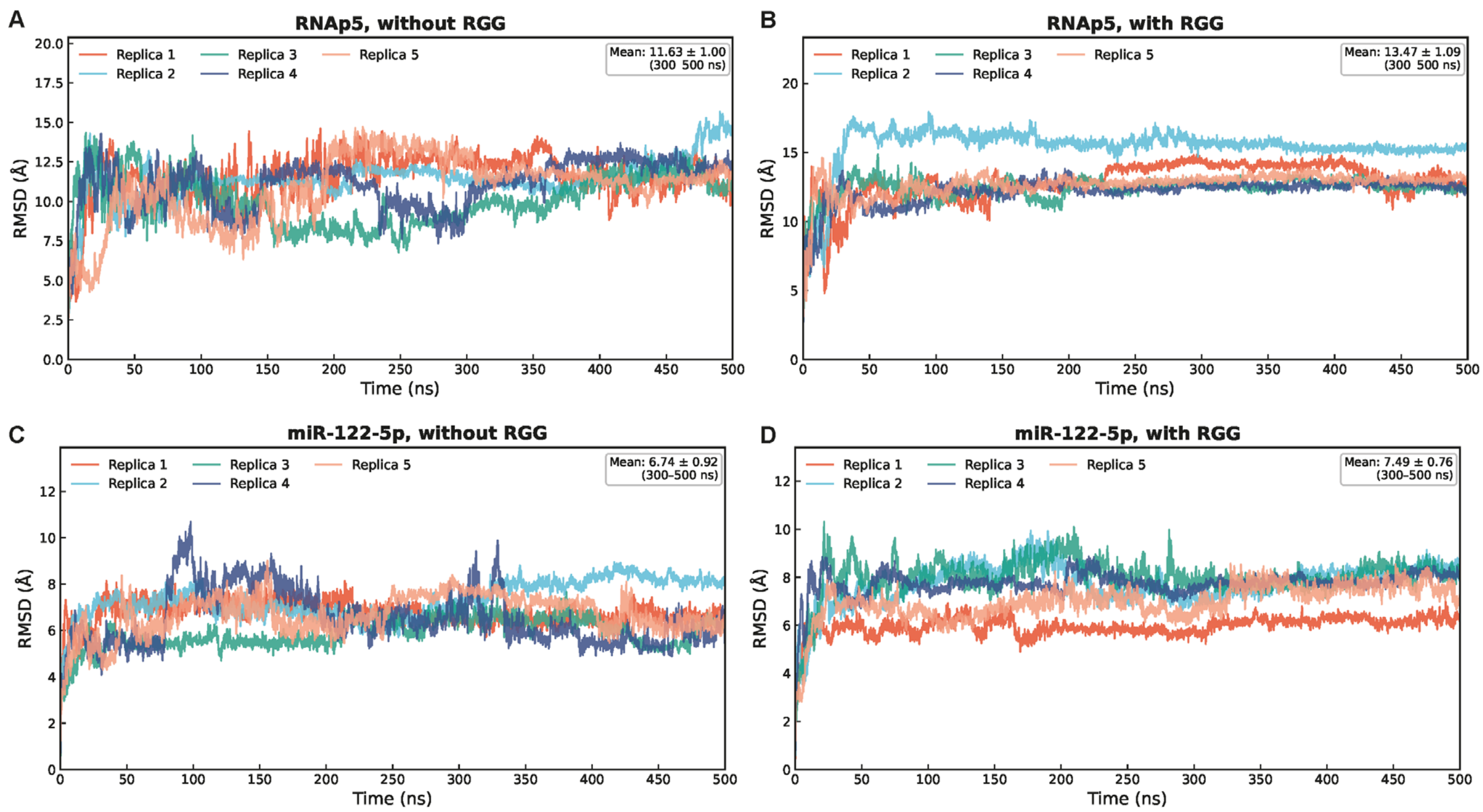

Figure S16. RNA structural dynamics in the 1× RNA-bound METTL3–METTL14 systems. RNA RMSD over five independent 500 ns simulations for RNAp5 in the absence (A) or presence (B) of the METTL14 RGG region, and for miR-122-5p in the absence (C) or presence (D) of the RGG region. RMSD was calculated after least-squares fitting of each trajectory to the RNA coordinates of its first frame. Boxed values report the mean ± SD over the 300–500 ns analysis interval. The RGG-containing systems maintained broadly stable RMSD plateaus over this interval. Their moderately higher mean RMSD values may reflect adoption of alternative RGG-associated RNA conformations.

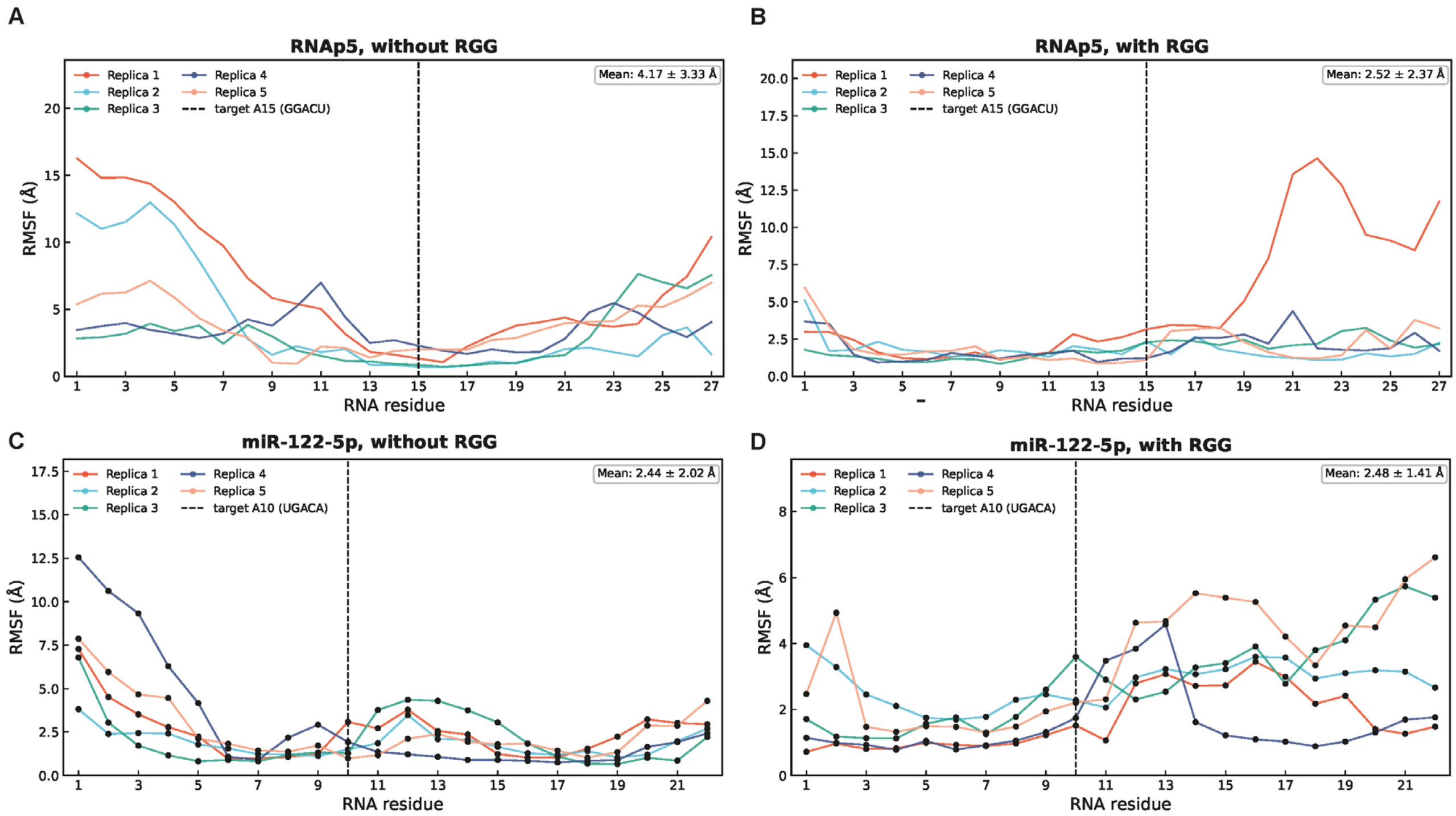

Figure S17. Per-residue RNA flexibility in the 1× RNA-bound METTL3–METTL14 systems. Per-residue RNA RMSF calculated over the 300–500 ns interval for RNAp5 in the absence (A) or presence (B) of the METTL14 RGG region, and for miR-122-5p in the absence (C) or presence (D) of the RGG region. Trajectories were aligned to the METTL3–METTL14 complex before calculation, and five independent simulations are shown for each system. Dashed vertical lines indicate the target adenosine, A15 within the RNAp5 GGACU motif and A10 within the miR-122-5p UGACA motif. Boxed values report the mean per-residue RMSF ± SD across the analysed trajectories. Inclusion of the RGG region reduced the mean flexibility of RNAp5 from 4.17 to 2.52 Å, consistent with the RGG region providing an additional anchoring surface. By contrast, the mean flexibility of miR-122-5p remained essentially unchanged (2.44 versus 2.48 Å), although the distribution of local flexibility along the RNA sequence was altered.

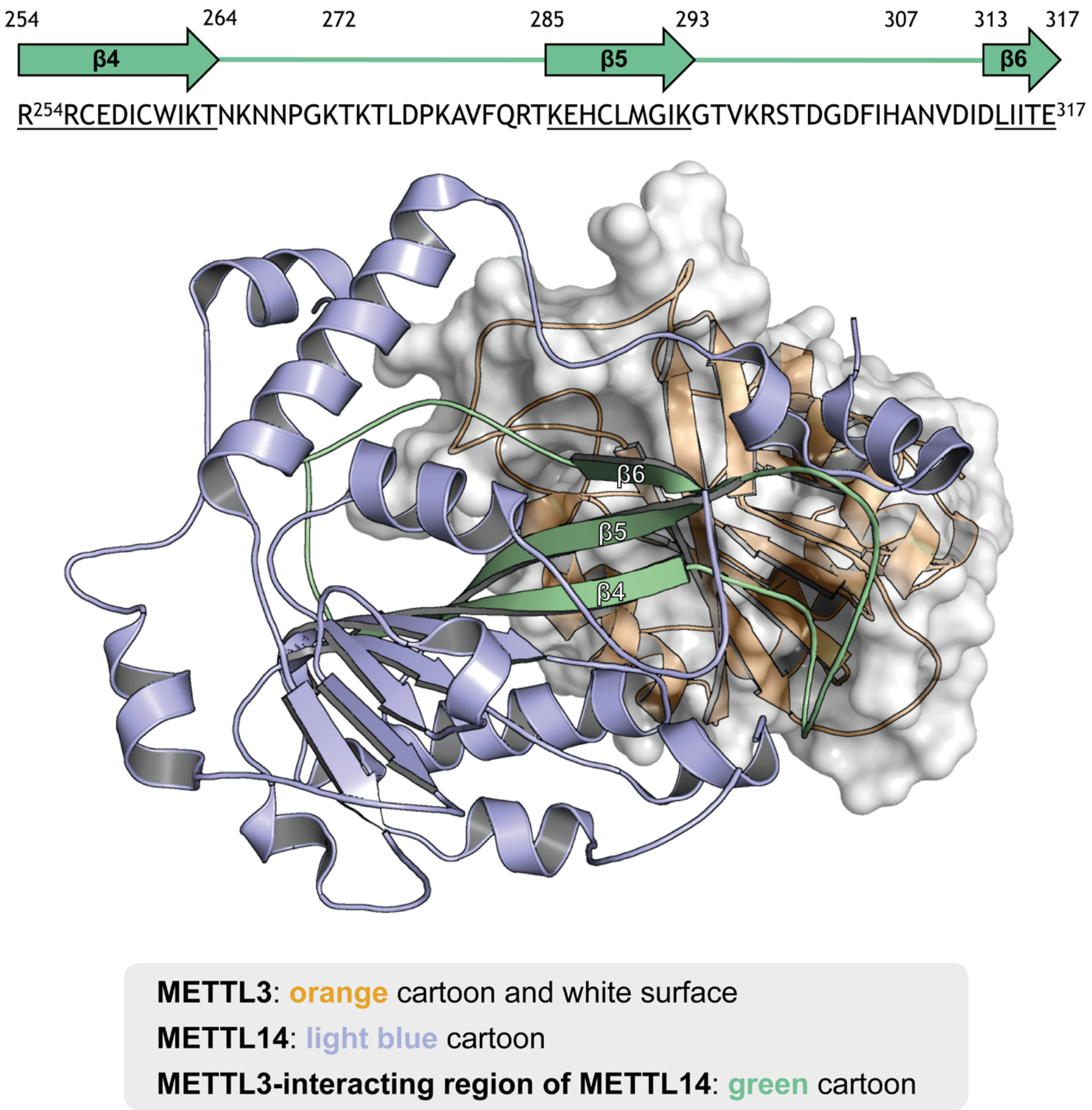

Figure S18. METTL3–METTL14 interaction interface. Top: Schematic representation of the METTL3-interacting region of METTL14. Arrows denote β-strand elements, and connecting lines indicate loop regions; the corresponding METTL14 residues are shown in single-letter code. Bottom: Crystal structure of the METTL3–METTL14 complex (PDB: 5IL2).[16] METTL3 is displayed as an orange cartoon with a white surface, METTL14 as a light-blue cartoon, and the METTL3-interacting region of METTL14 is highlighted in green.

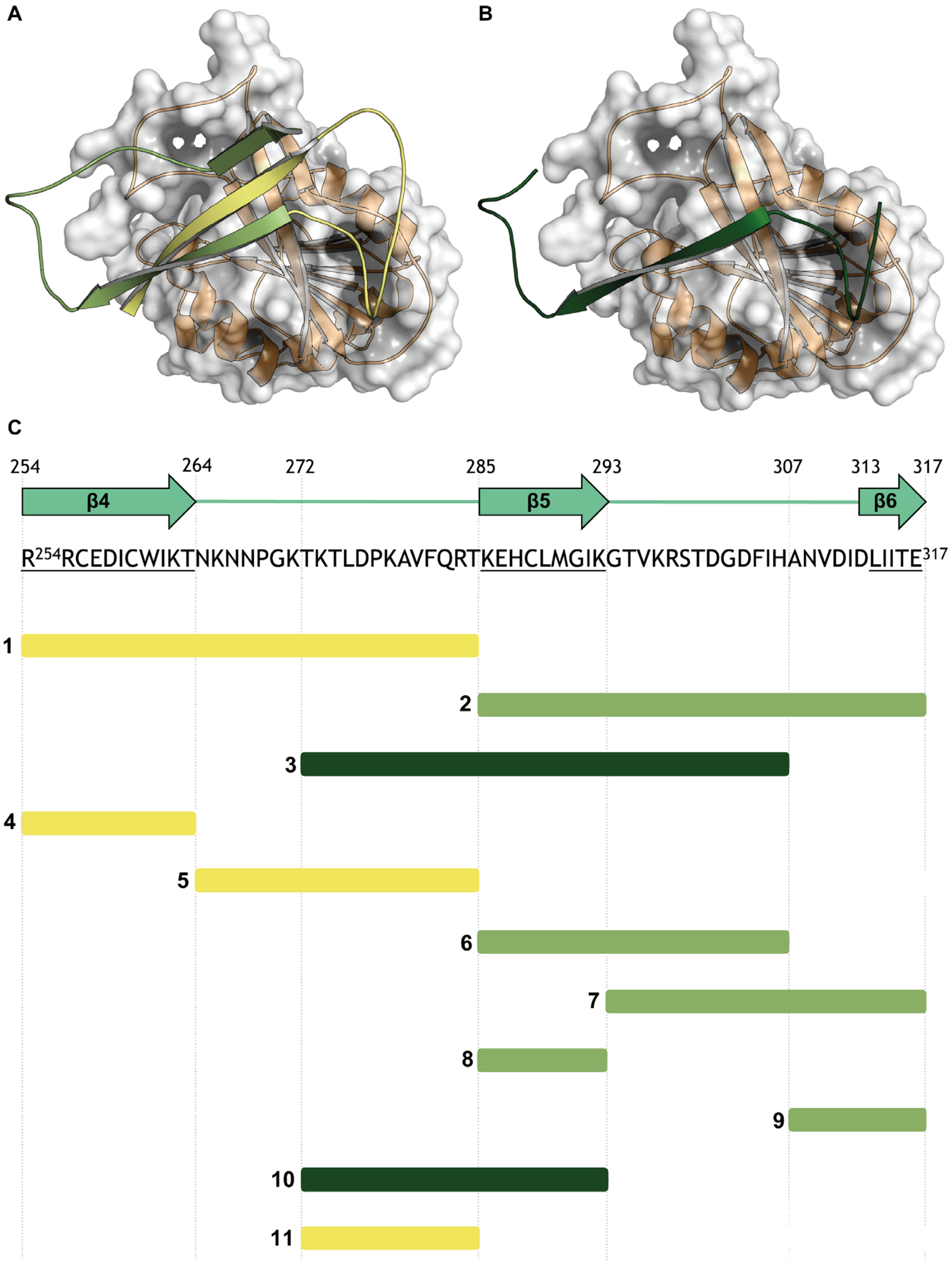

Figure S19. Progressive truncation of the METTL3–METTL14 interaction surface. A) Mapping of the initial METTL14-derived segments peptide **1** (yellow) and peptide **2** (light green), spanning the β4–β5–β6 region (METTL14 residues 254–317), onto the METTL3–METTL14 crystal structure (PDB: 5IL2).[16] METTL3 is shown as an orange cartoon with a white surface, the extracted interface segments of METTL14 are shown in yellow (**1**) and light green (**2**). B) Structural placement of peptide **3** (dark green), derived from the β5 strand and flanking loop regions (METTL14 residues 272–307), displayed on the same complex. C) Linear schematic of METTL14 residues 254–317 showing secondary-structure elements (β4–β6, arrows) and sequence; colored bars (**1–11**) summarize the stepwise truncation strategy, with colors indicating the starting peptide (**1-3**) from which each fragment was derived.

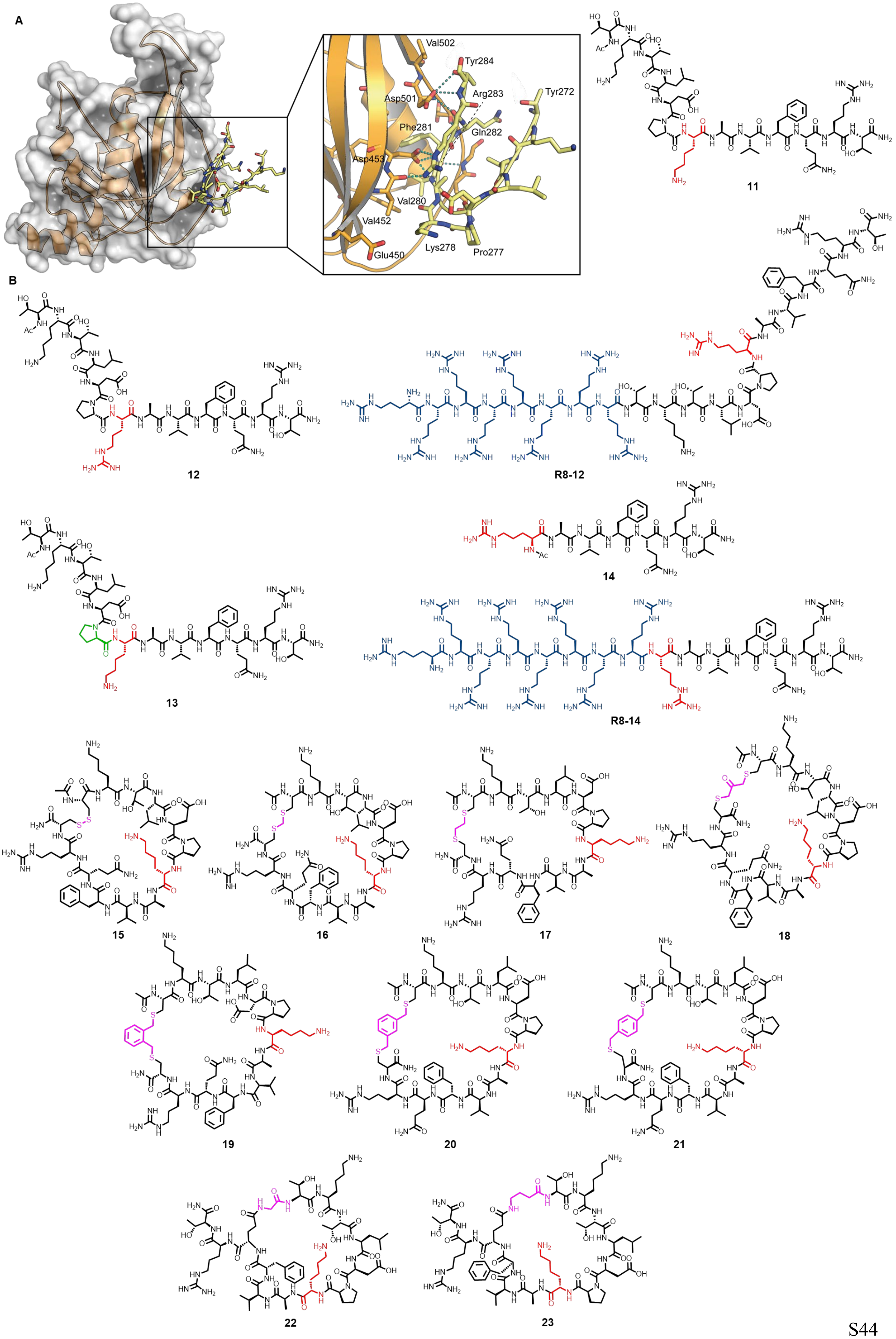
A
Val502
Tyr284
Arg283
Tyr272
Asp501
Phe281
Gln282
Asp453
Val280
Val452
Lys278
Pro277
Glu450
11
B
12
R8-12
13
14
R8-14
15
16
17
18
19
20
21
22
23

Figure S20. A) Left panel: View of METTL3 (orange cartoon with a white surface) and the METTL14 loop region corresponding to peptide **11** (yellow) (PDB ID: 5IL2).[16] The zoomed inset shows the region covering peptide **11** embedded within the METTL3 molecular surface. Selected residues are displayed as sticks; cyan dashed lines denote polar interactions, and red dashed lines indicate the Tyr272–Tyr284 distance (5.5 Å). Right panel: chemical structure of peptide **11**. B) Chemical structures of peptides **11-23**.

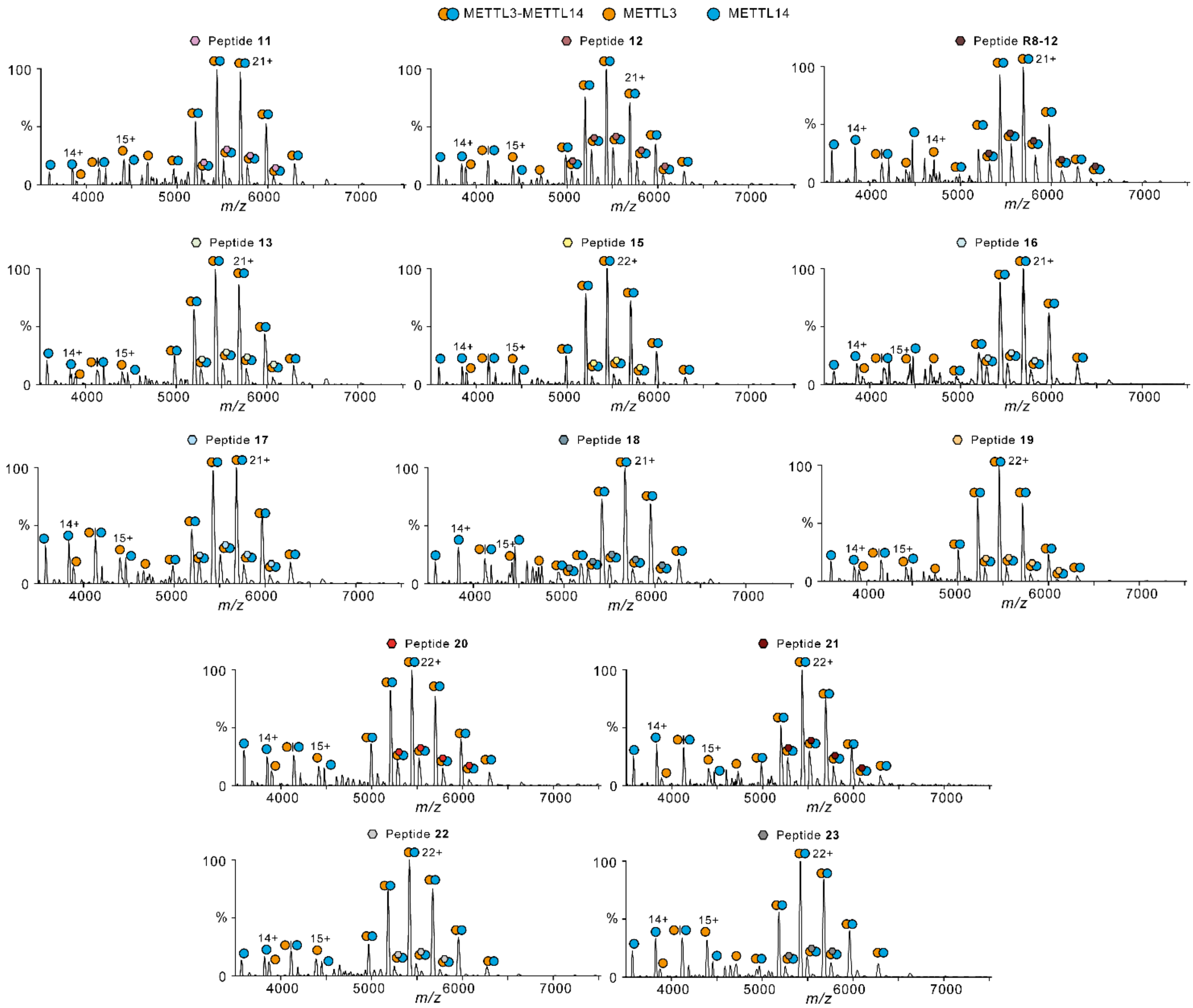

Figure S21. Native mass spectra of METTL3–METTL14 (2.5 µM) recorded in the presence of peptides **11-23** (5 µM).

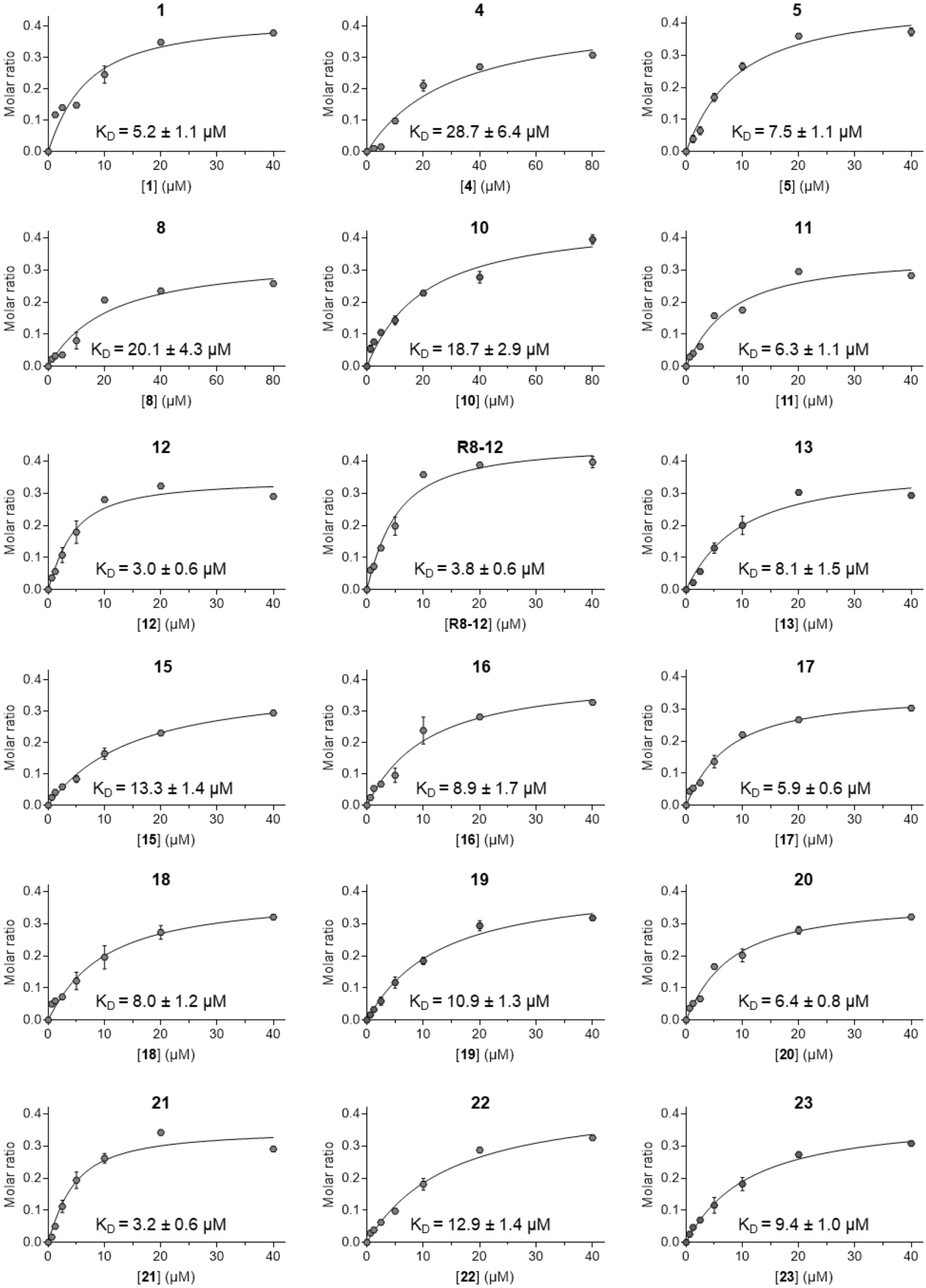

Figure S22. Dose–response curves showing concentration-dependent binding of METTL3–METTL14 to peptides **1**, **4**, **5**, **8**, **10-12**, **R8-12**, **13**, and **15-23**. The molar ratio of the 1× peptide-bound heterodimer was calculated relative to the total assigned heterodimeric population, and fitted to derive apparent K_D values. Data are reported as mean ± SD from three independent biological replicates (n = 3).

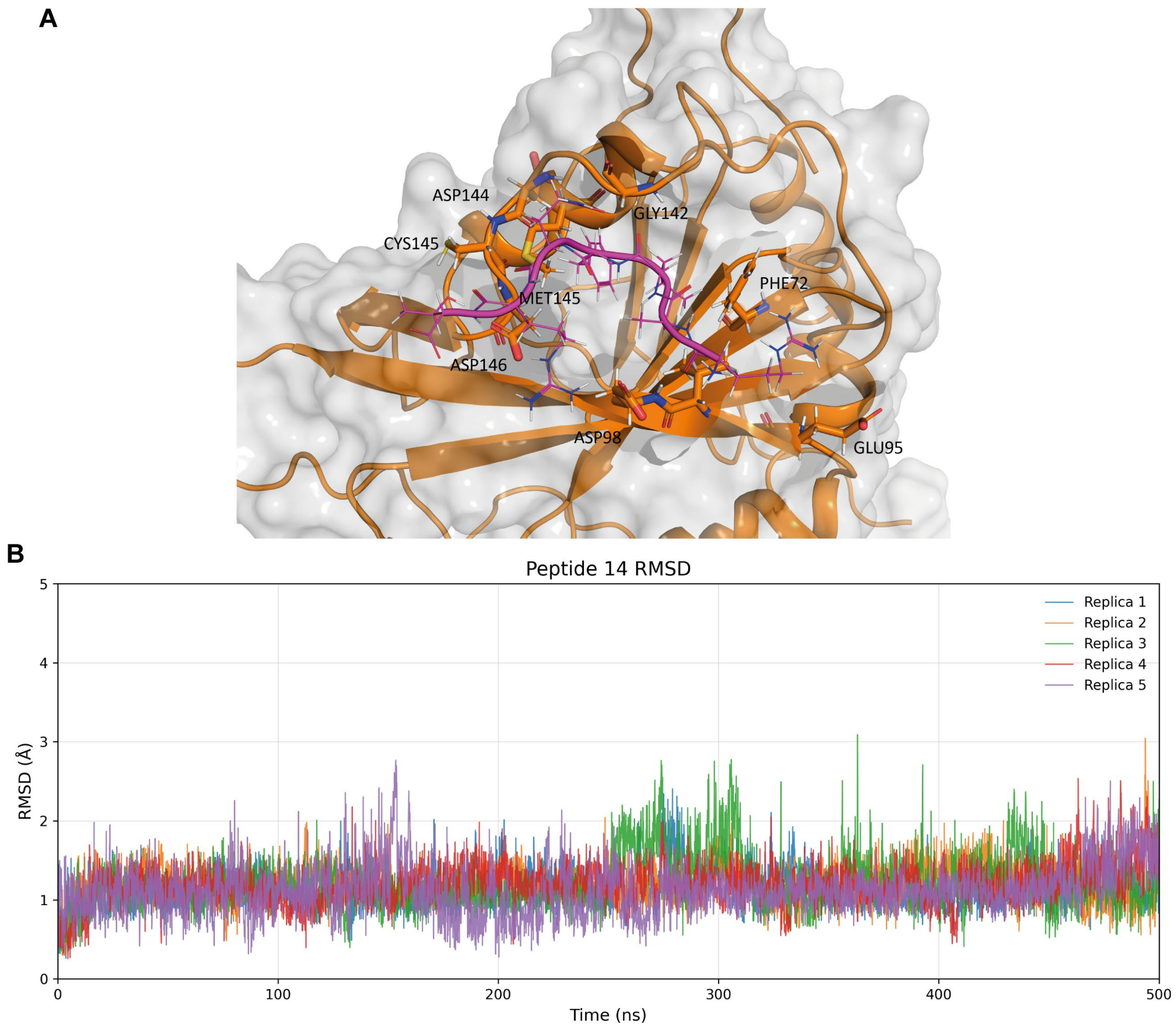

Figure S23. Docking and MD of peptide binding to the METTL complex. A) Detailed view of peptide **14** binding to METTL3. Key residues in METTL3 that interact with peptide **14** are shown as sticks. B) RMSD of the Cα atoms of docked peptide 14 relative to its initial bound pose over 500 ns of MD simulations.

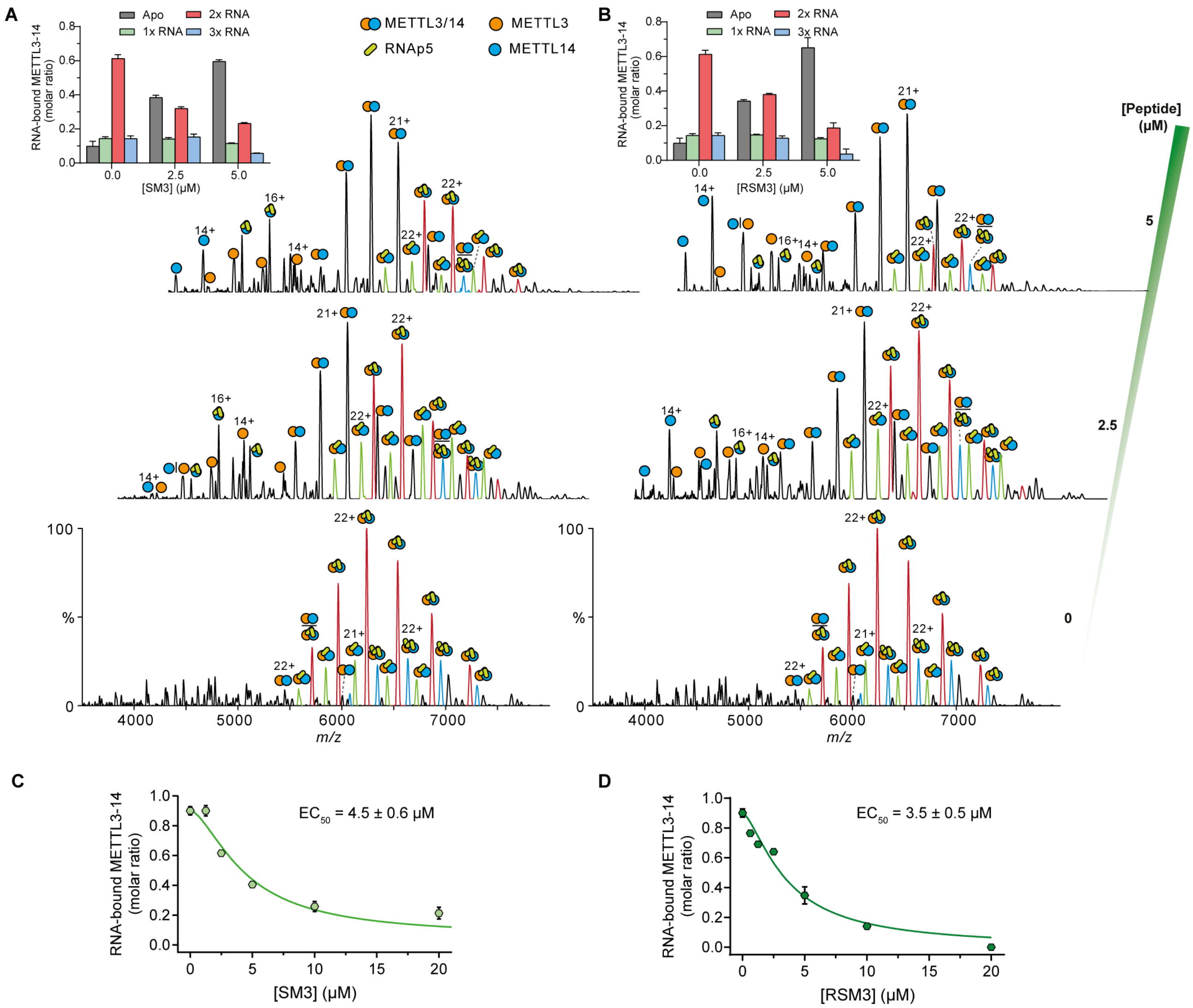

Figure S24. A,B) Native mass spectra of METTL3–METTL14 (2.5 µM) recorded in the presence of RNAp5 (25 µM) and increasing concentrations of SM3 (A) or RSM3 (B). Charge state series corresponding to RNA-bound complexes decrease in a concentration-dependent manner, accompanied by an increased contribution of charge states assigned to free METTL3 and METTL14. Bar charts report the relative abundances of METTL3–METTL14 bound to one, two, or three RNA molecules as determined from the assigned charge-state series. C,D) Quantification of METTL3–METTL14 bound to RNAp5 across the tested peptide concentrations and fitting to derive EC_{50} values. The quantification shows a dose-dependent decrease of total RNA bound species at the tested concentrations. Data are expressed as mean ± standard deviation (SD) of three biological replicates (n = 3).

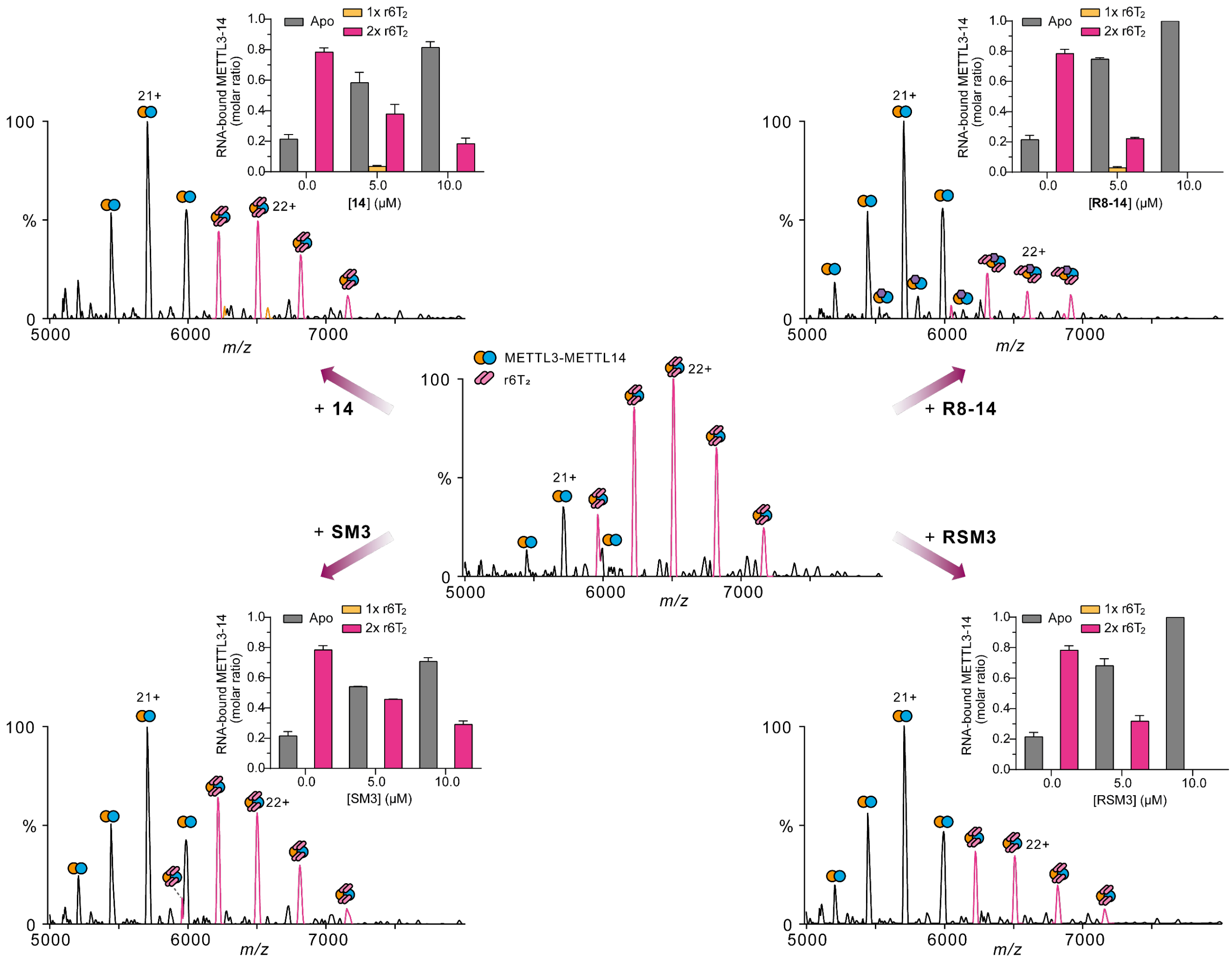

Figure S25. Native mass spectra of METTL3–METTL14 (2.5 µM) recorded in the presence of r6T (25 µM) and peptides **14**, **R8-14**, SM3, and RSM3 (5 µM). Charge state series corresponding to RNA-bound complexes decrease compared to the non-treated sample. Bar charts report the relative abundances of METTL3–METTL14 bound to one or two r6T duplex units ($r6T_2$) as determined from the assigned charge-state series. Data are expressed as mean ± standard deviation (SD) of three biological replicates (n = 3).

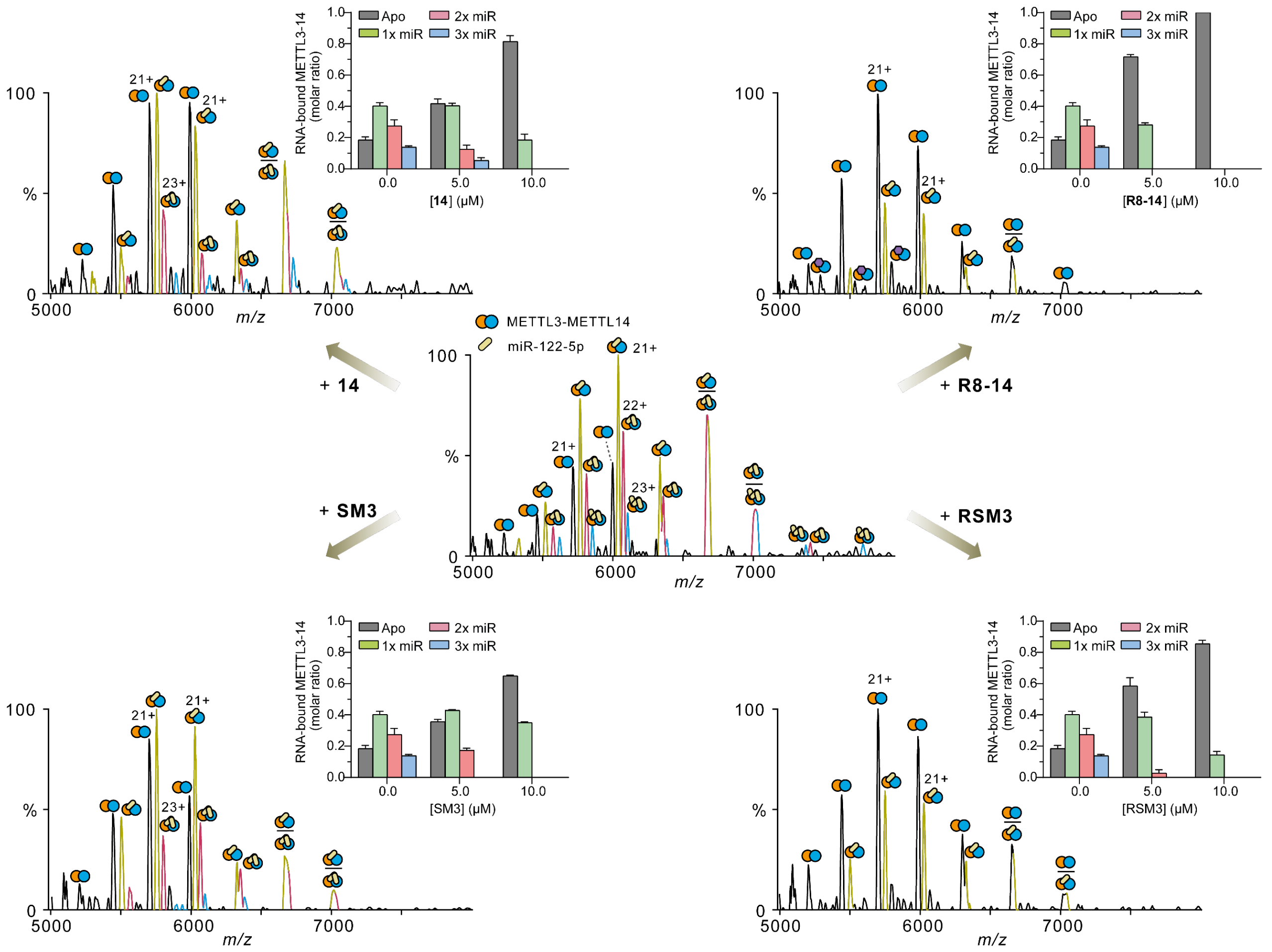

Figure S26. Native mass spectra of METTL3–METTL14 (2.5 µM) recorded in the presence of miR-122-5p (25 µM) and peptides **14**, **R8-14**, SM3, and RSM3 (5 µM). Charge state series corresponding to RNA-bound complexes decrease compared to the non-treated sample. Bar charts report the relative abundances of METTL3–METTL14 bound to one, two, or three RNA molecules as determined from the assigned charge-state series. Data are expressed as mean ± standard deviation (SD) of three biological replicates (n = 3).

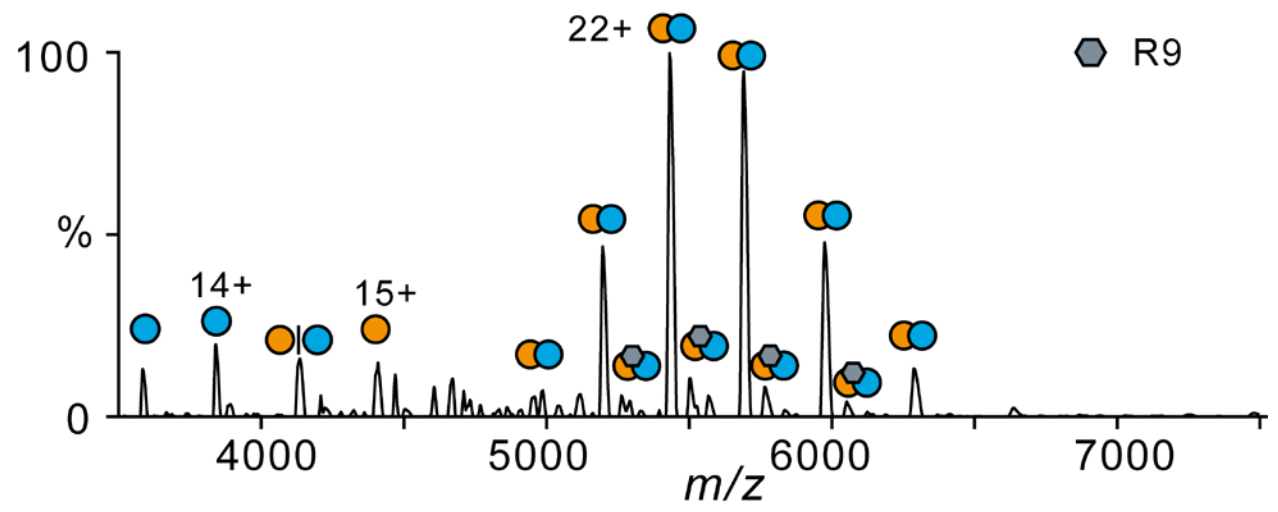

Figure S27. Native mass spectrum of METTL3–METTL14 (2.5 µM) in the presence of R9 (40 µM). Only a minor fraction of the complex (~10%) is bound to R9, indicating weak interaction.

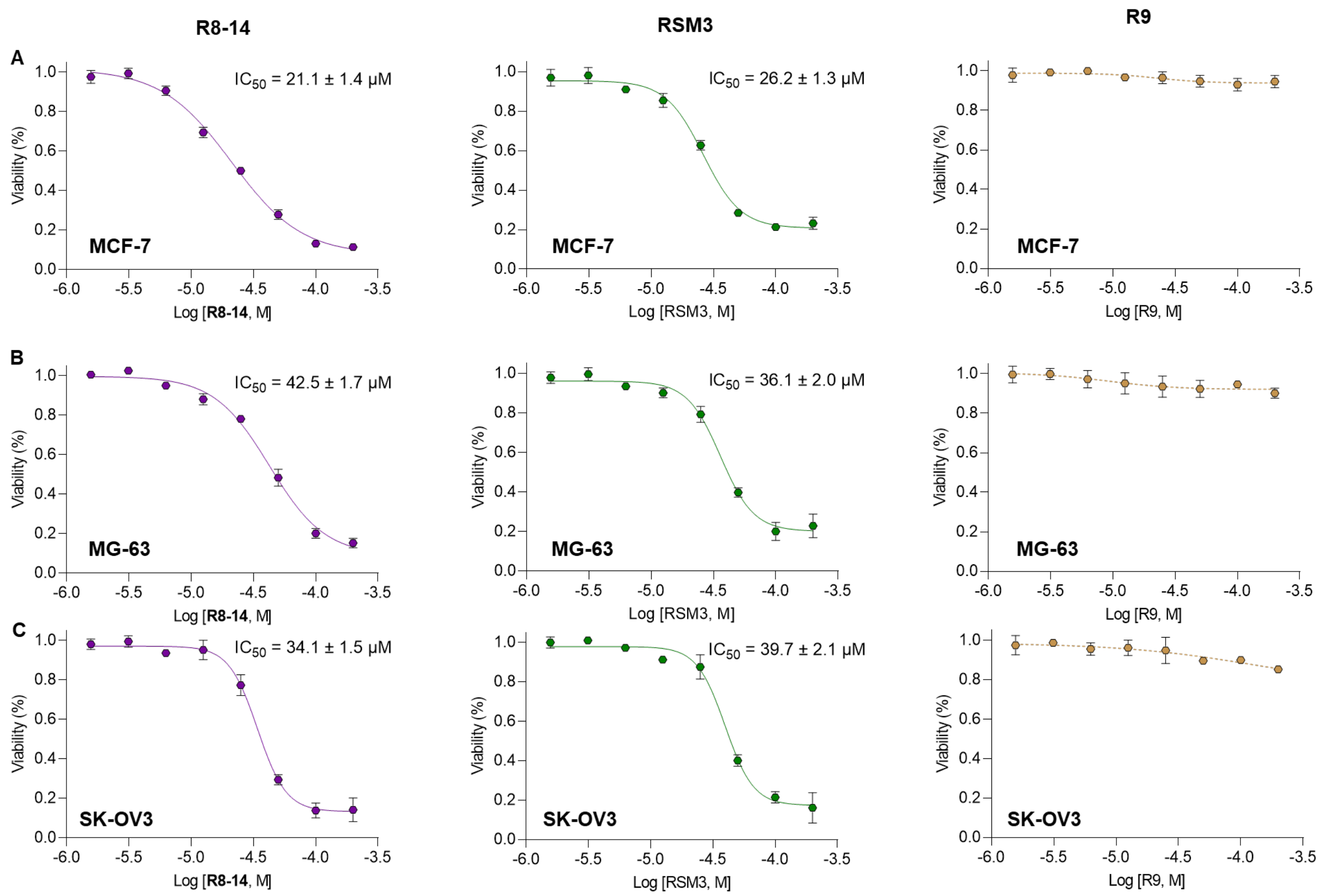

Figure S28. Dose–response curves showing the antiproliferative effects of **R8–14**, RSM3, and R9 after 24 h treatment at increasing concentrations in MCF-7 (A), MG-63 (B), and SK-OV3 (C) cells. Vehicle-treated cells (H_2O) served as controls.

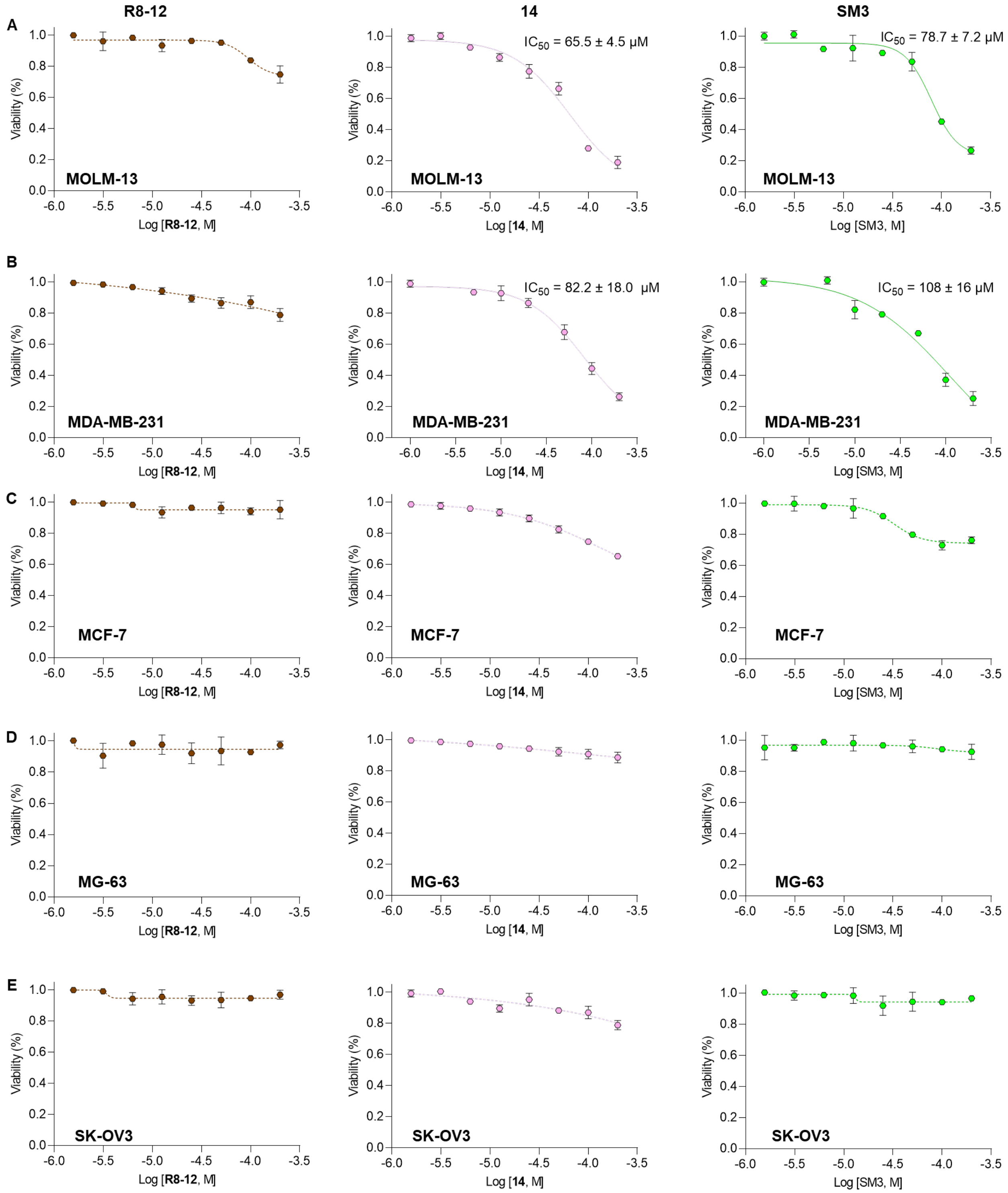

Figure S29. Dose–response curves showing the antiproliferative effects of **R8–12**, **14**, and SM3, after 24 h treatment at increasing concentrations in MOLM-13 (A), MDA-MB-231 (B), MCF-7 (C), MG-63 (D), and SK-OV3 (E) cells. Vehicle-treated cells (H_2O) served as controls.

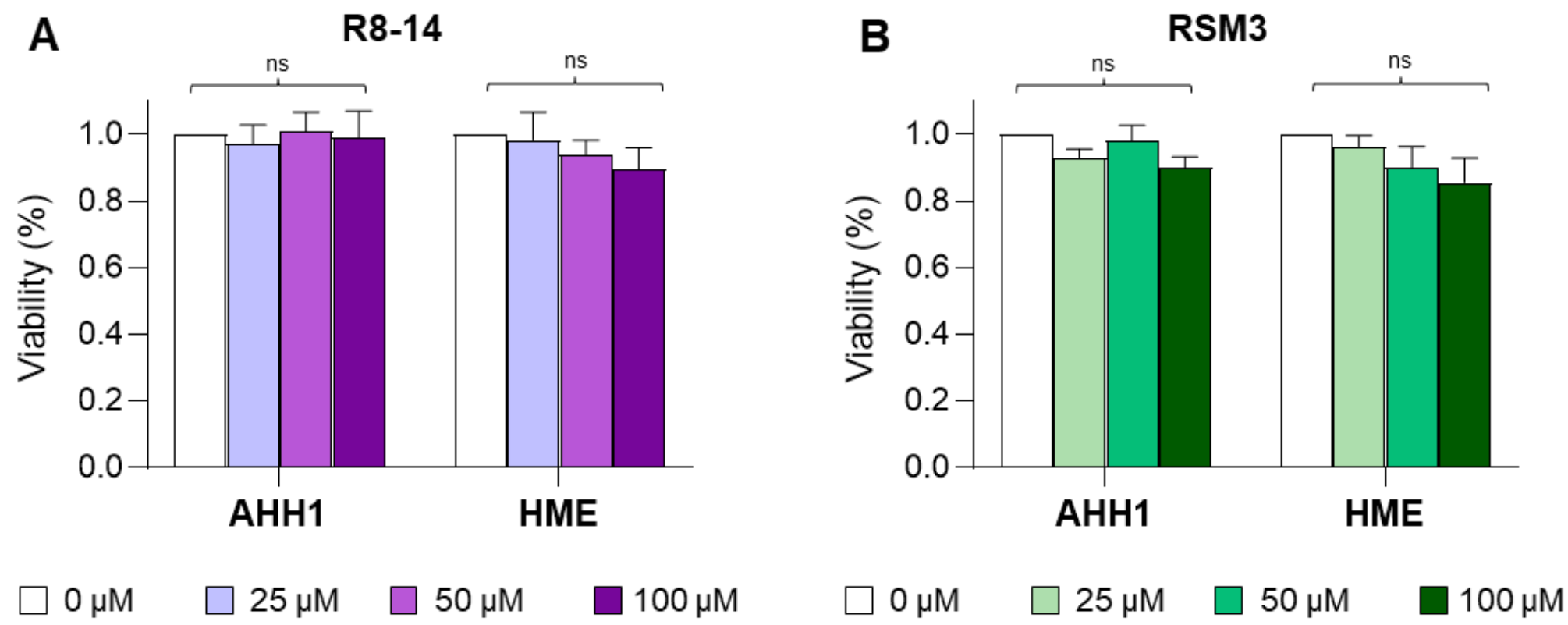

Figure S30. Effect of **R8-14** (A) and RSM3 (B) on the viability of B lymphocyte AHH1 cells and mammary epithelial cells (HME) after 24 h incubation at 2, 50, and 100 µM.

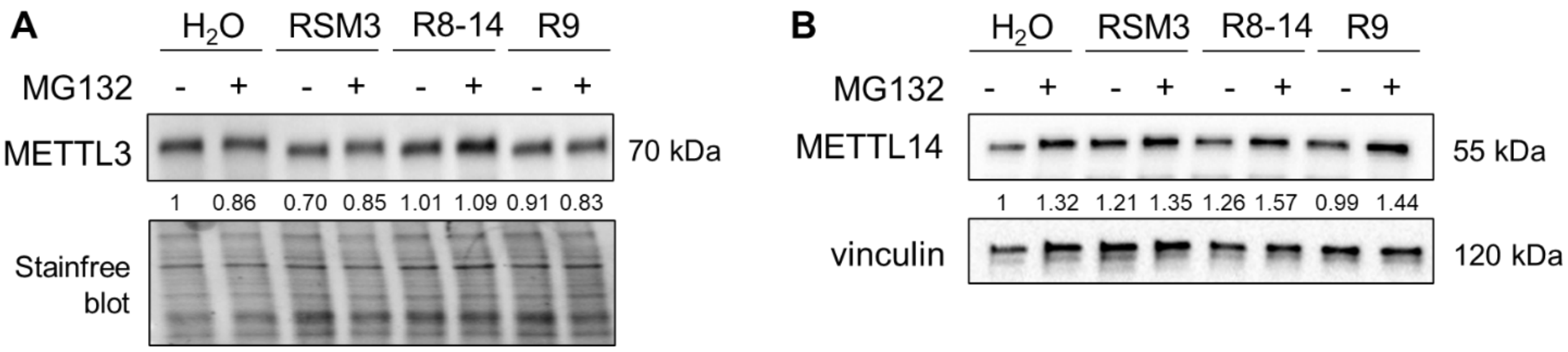

Figure S31. (A) WB analysis of METTL3 levels in MOLM-13 cells following 24 h exposure to RSM3, **R8–14**, or R9 (2.5 µM) in the absence or in the presence of the proteasome inhibitor MG132 (10 µM). The densiometric quantification is expressed as fold change relative to vehicle (H_2O) after normalization. (B) WB analysis of METTL3 levels in MOLM-13 cells following 24 h exposure to RSM3, **R8–14**, or R9 (2.5 µM) in the absence or in the presence of the proteasome inhibitor MG132 (10 µM). Vinculin was used as a loading control. The densiometric quantification is expressed as fold change relative to vehicle (H_2O) after normalization.

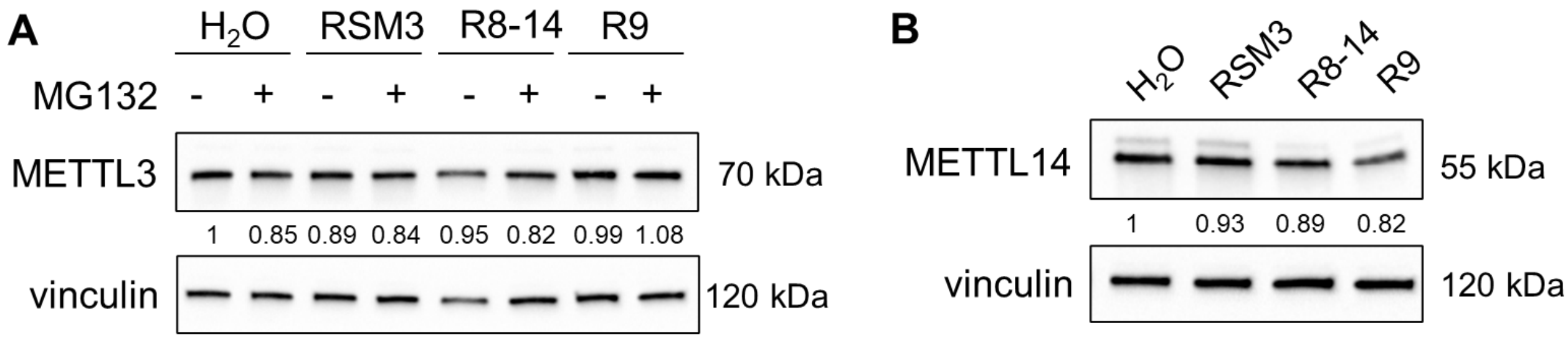

Figure S32. (A) WB analysis of METTL3 levels in MDA-MB-231 cells following 24 h exposure to RSM3, **R8–14**, or R9 (8 μM) in the absence or in the presence of the proteasome inhibitor MG132 (10 μM). Vinculin was used as a loading control. The densiometric quantification is expressed as fold change relative to vehicle (H_2O) after normalization. (B) WB analysis of METTL3 levels in MOLM-13 cells following 24 h exposure to RSM3, **R8–14**, or R9 (8 μM). Vinculin was used as a loading control. The densiometric quantification is expressed as fold change relative to vehicle (H_2O) after normalization.

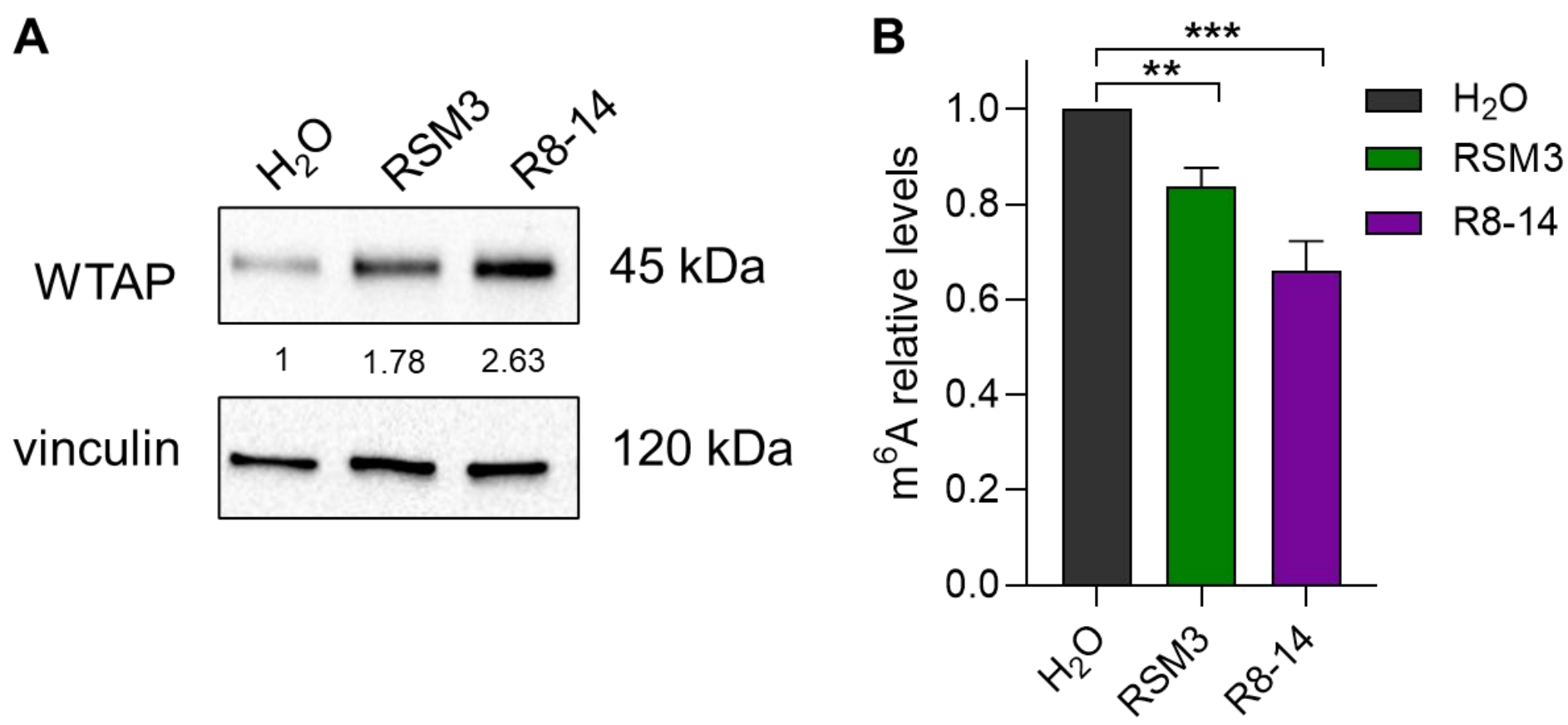

Figure S33. A) WB analysis of WTAP levels in MOLM-13 cells following 24 h exposure to RSM3 or **R8–14** (2.5 μM). Vinculin was used as a loading control. The densiometric quantification is expressed as fold change relative to vehicle (H_2O) after normalization. B) Global m^6A quantification in MDA-MB-231 cells after 24 h treatment with RSM3 or **R8-14**, expressed as fold change relative to vehicle. All experiments were performed in triplicate ($n = 3$); data are presented as mean ± SD. Statistical significance was assessed by one-way ANOVA versus vehicle (**$p < 0.01$; ***$p < 0.001$).

Table S1. Effect on cell viability of peptides **R8-14**, RSM3, R9, **R8-12**, **14**, and SM3 expressed as IC_{50} values (μM) after 24 h treatment in MOLM-13, MDA-MB-231, MCF-7, MG-63, and SK-OV3 cells.

Peptide	IC_{50} (μM)[a] - 24h				
	MOLM-13	MDA-MB-231	MCF-7	MG-63	SK-OV3
**R8-14**	2.9 ± 0.3	11.0 ± 0.6	21.1 ± 1.4	42.5 ± 1.7	34.1 ± 1.5
**RSM3**	5.2 ± 0.4	14.8 ± 1.3	26.2 ± 1.3	36.1 ± 2.0	39.7 ± 2.1
**R9**	N.I.[b]	N.I.	N.I.	N.I.	N.I.
**R8-12**	N.I.	N.I.	N.I.	N.I.	N.I.
**14**	65.5 ± 4.5	82.2 ± 18.0	N.I.	N.I.	N.I.
**SM3**	78.7 ± 7.2	108 ± 16	N.I.	N.I.	N.I.

[a]IC_{50}: Concentration required to reduce cell viability by 50%. Values are means ± standard deviation (SD).

[b]N.I.: no inhibition. IC_{50} was not determined since no effect on cell viability was observed at 100 μM.

HPLC and HRMS analysis of peptides 8, 9, 11-23, RM3, SM3, RSM3.

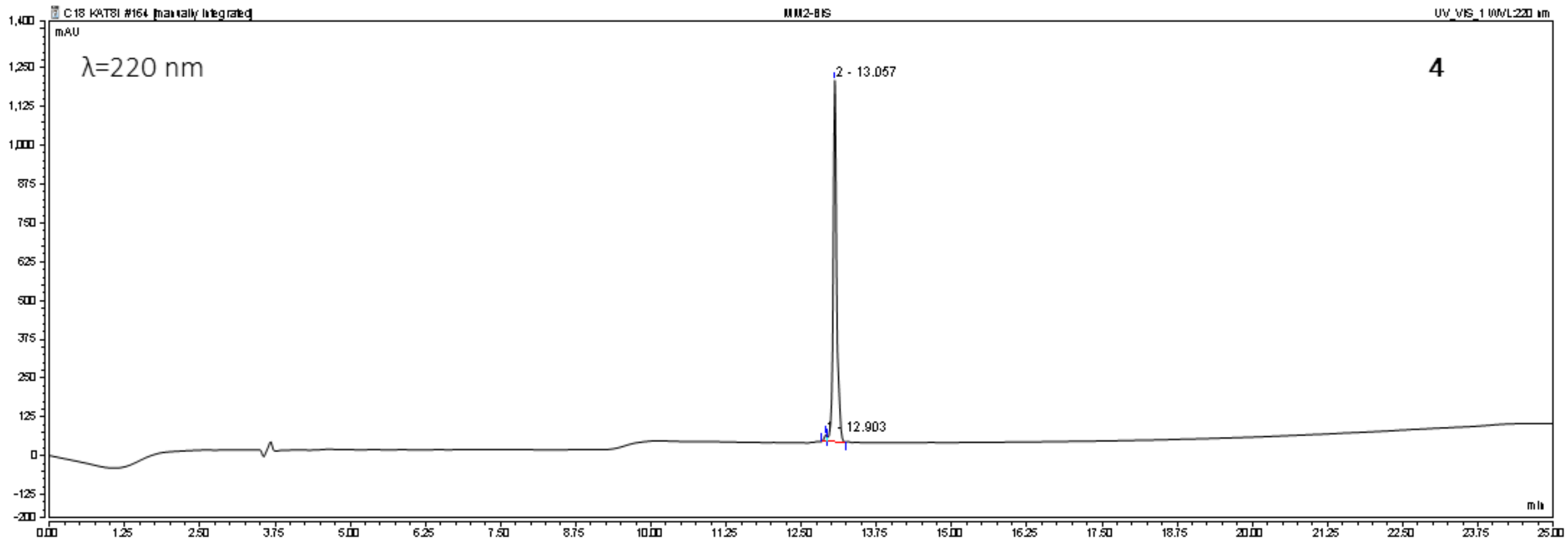

Peak n.	Retention Time (min)	Rel. Area (%)	Area (mAU*min)	Height (mAU)
1	12.903	1.48	1.1757	21.41
2	13.057	98.52	78.526	1164.47

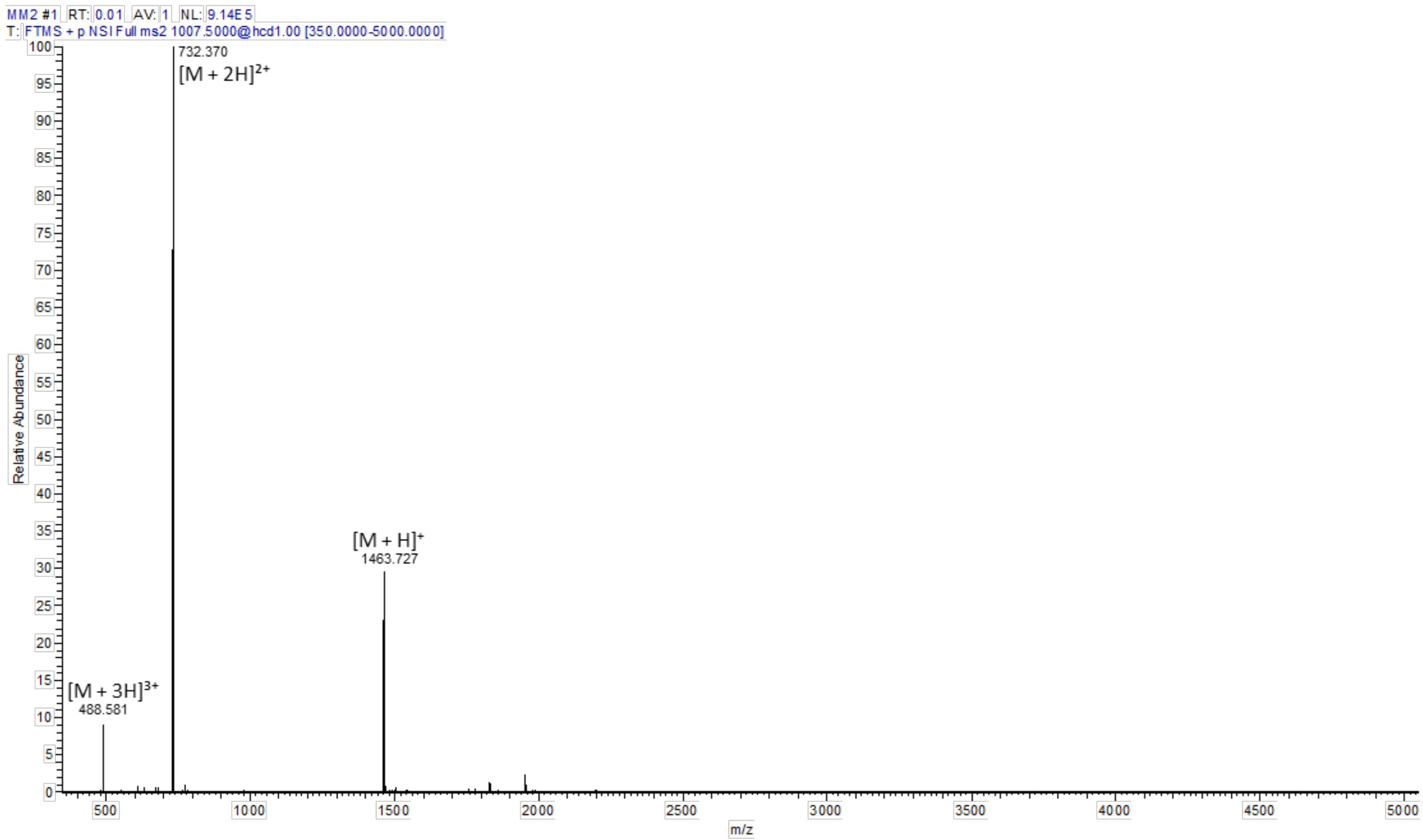

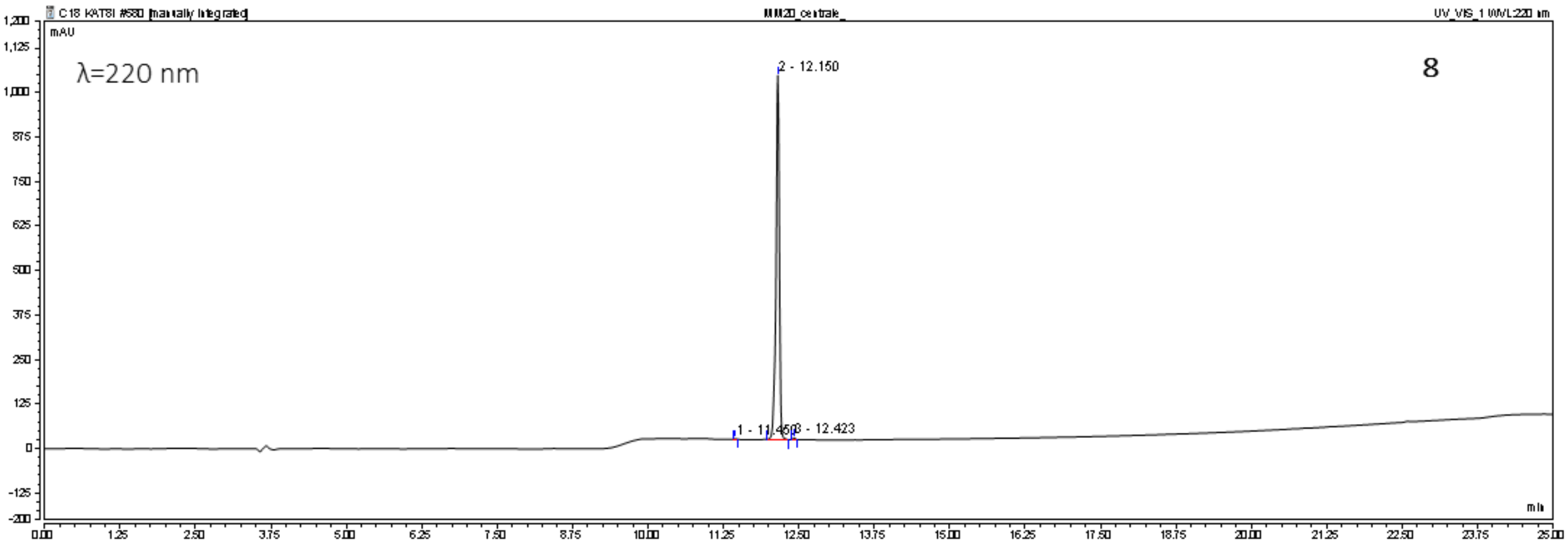

Peak n.	Retention Time (min)	Rel. Area (%)	Area (mAU*min)	Height (mAU)
1	11.45	0.1	0.0657	1.37
2	12.15	99.54	65.6774	1023.15
3	12.423	0.36	0.2406	5.2

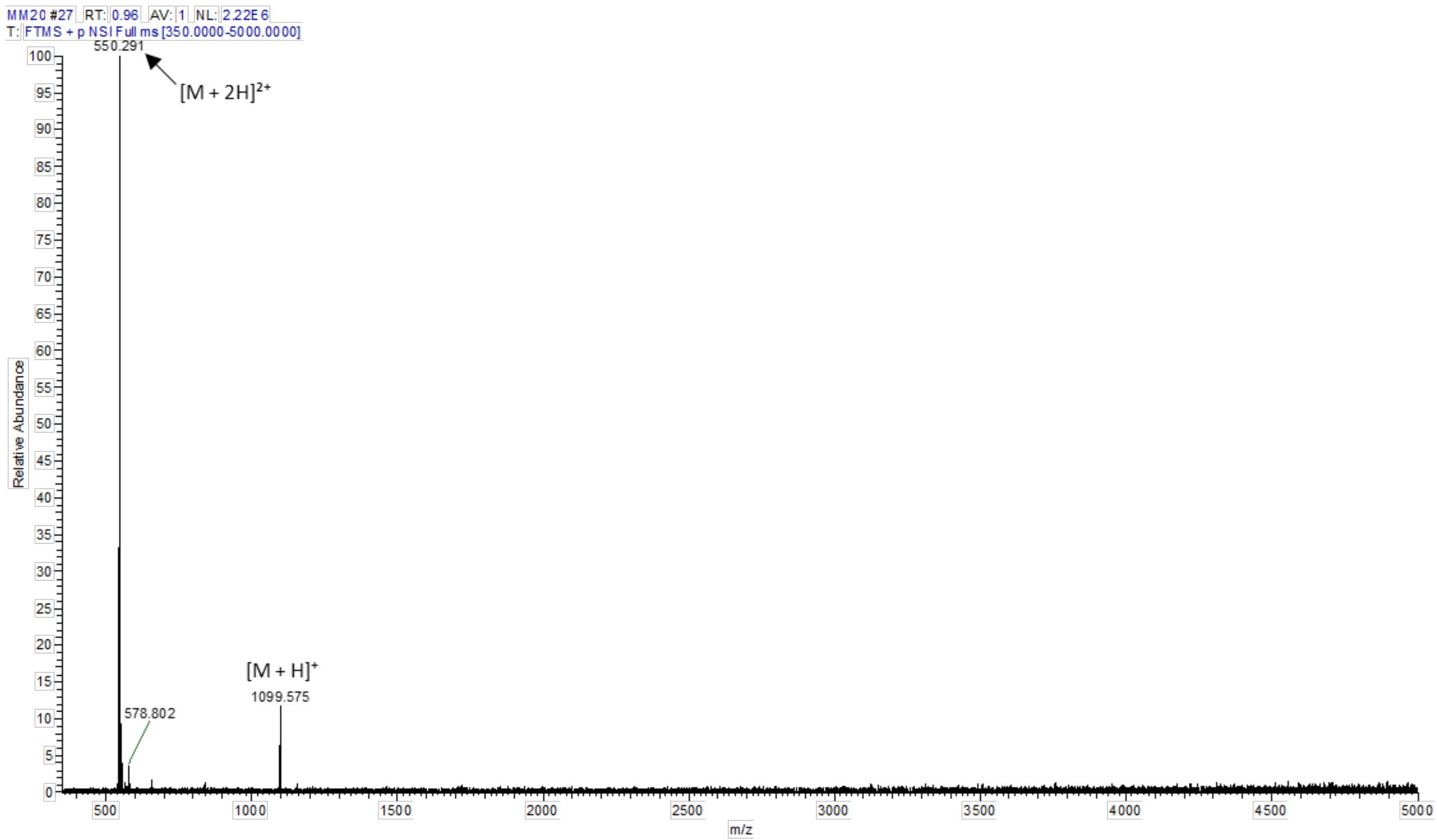

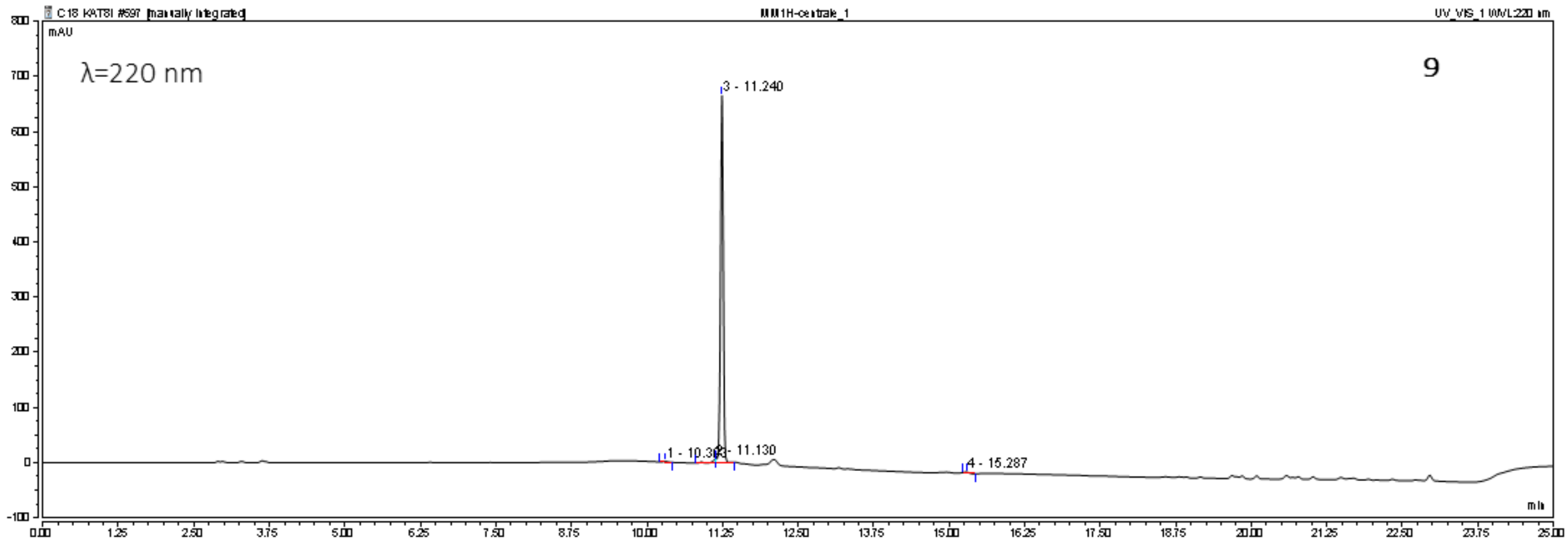

Retention Time (min)	Rel. Area (%)	Area (mAU*min)	Height (mAU)
10.303	0.29	0.11	1.1
11.13	1.11	0.4	6.3
11.24	98.32	35.38	664.9
15.287	2.64	0.097	1.8

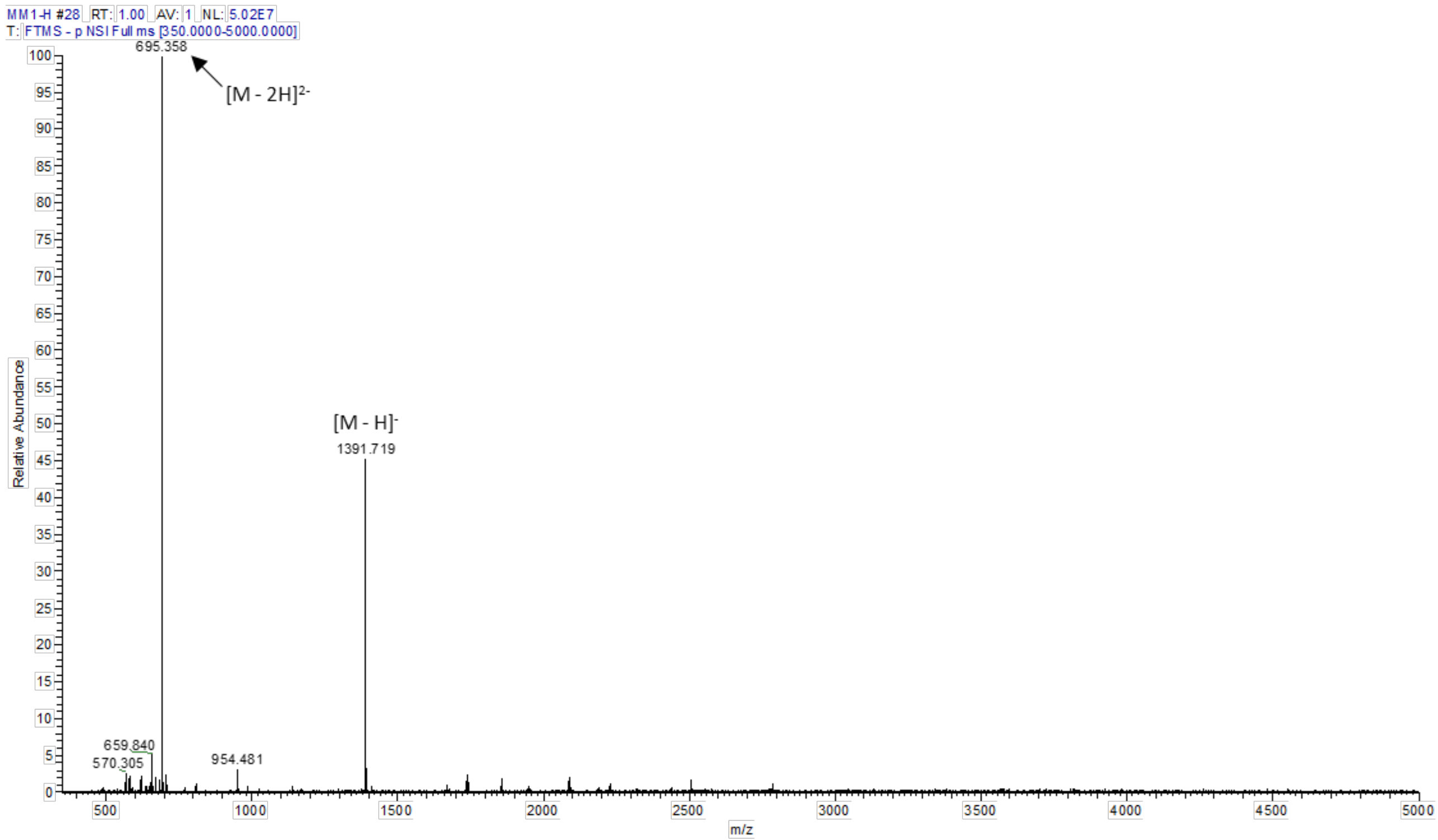

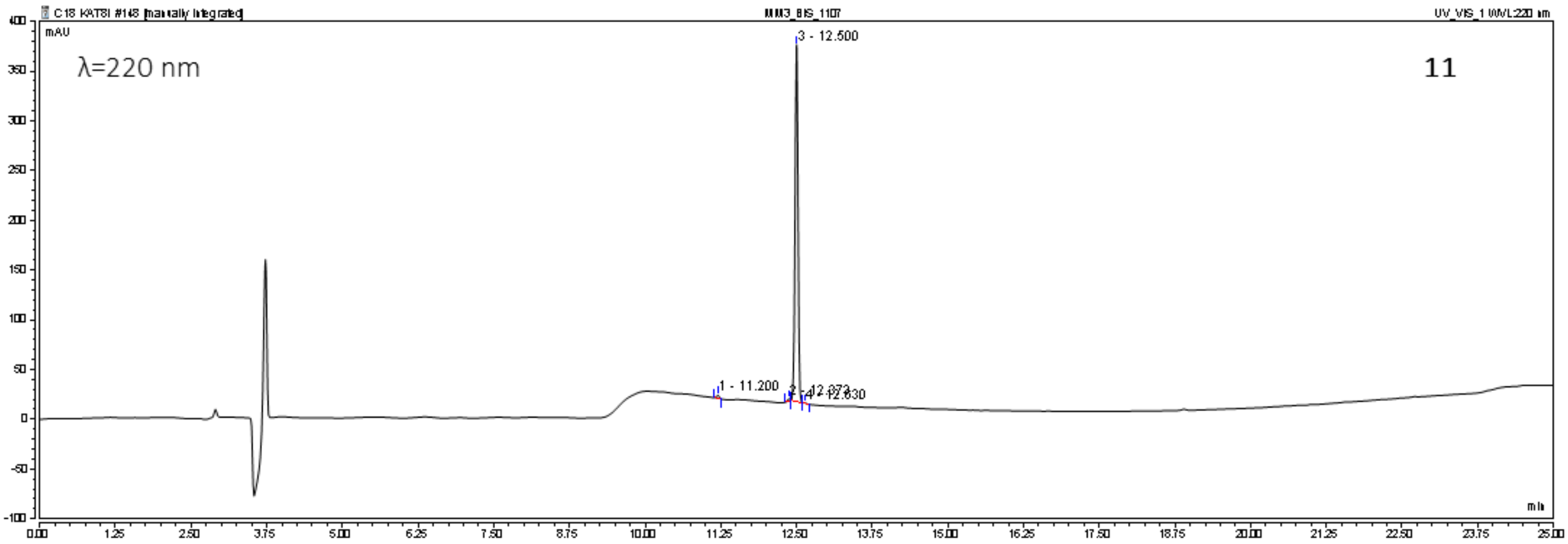

Peak n.	Retention Time (min)	Rel. Area (%)	Area (mAU*min)	Height (mAU)
1	11.2	0.72	0.1489	3.16
2	12.373	0.43	0.0889	2.44
3	12.5	98.72	20.3849	358.88
4	12.63	0.13	0.0266	0.63

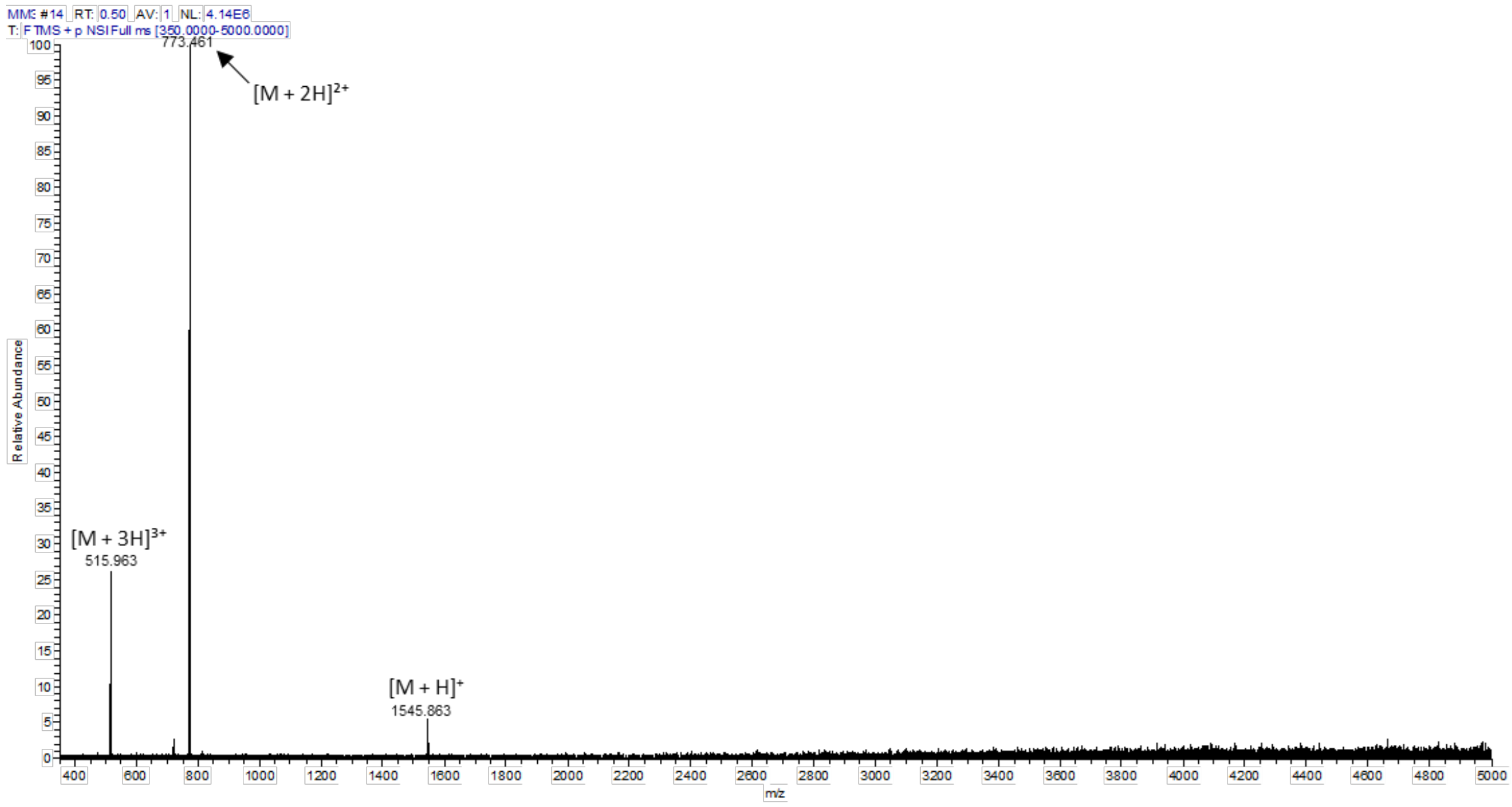

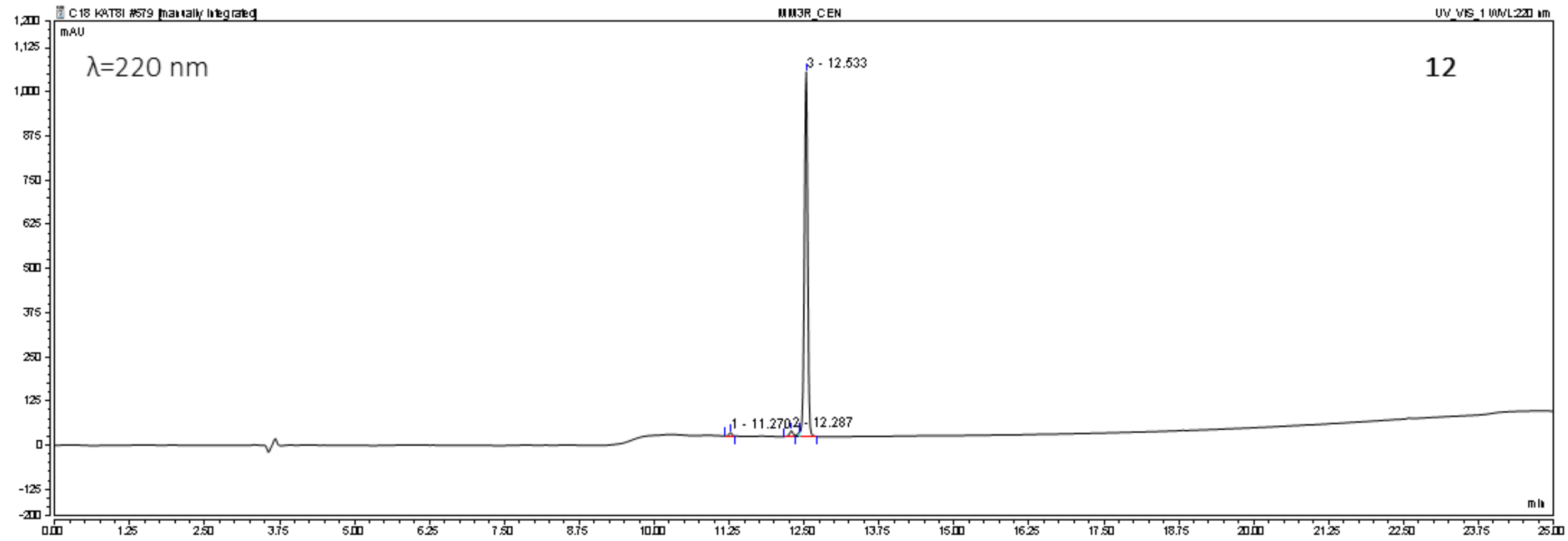

Peak n.	Retention Time (min)	Rel. Area (%)	Area (mAU*min)	Height (mAU)
1	11.27	0.75	0.5004	9.85
2	12.287	1.73	1.1604	16.55
3	12.533	97.52	65.2403	1032.15

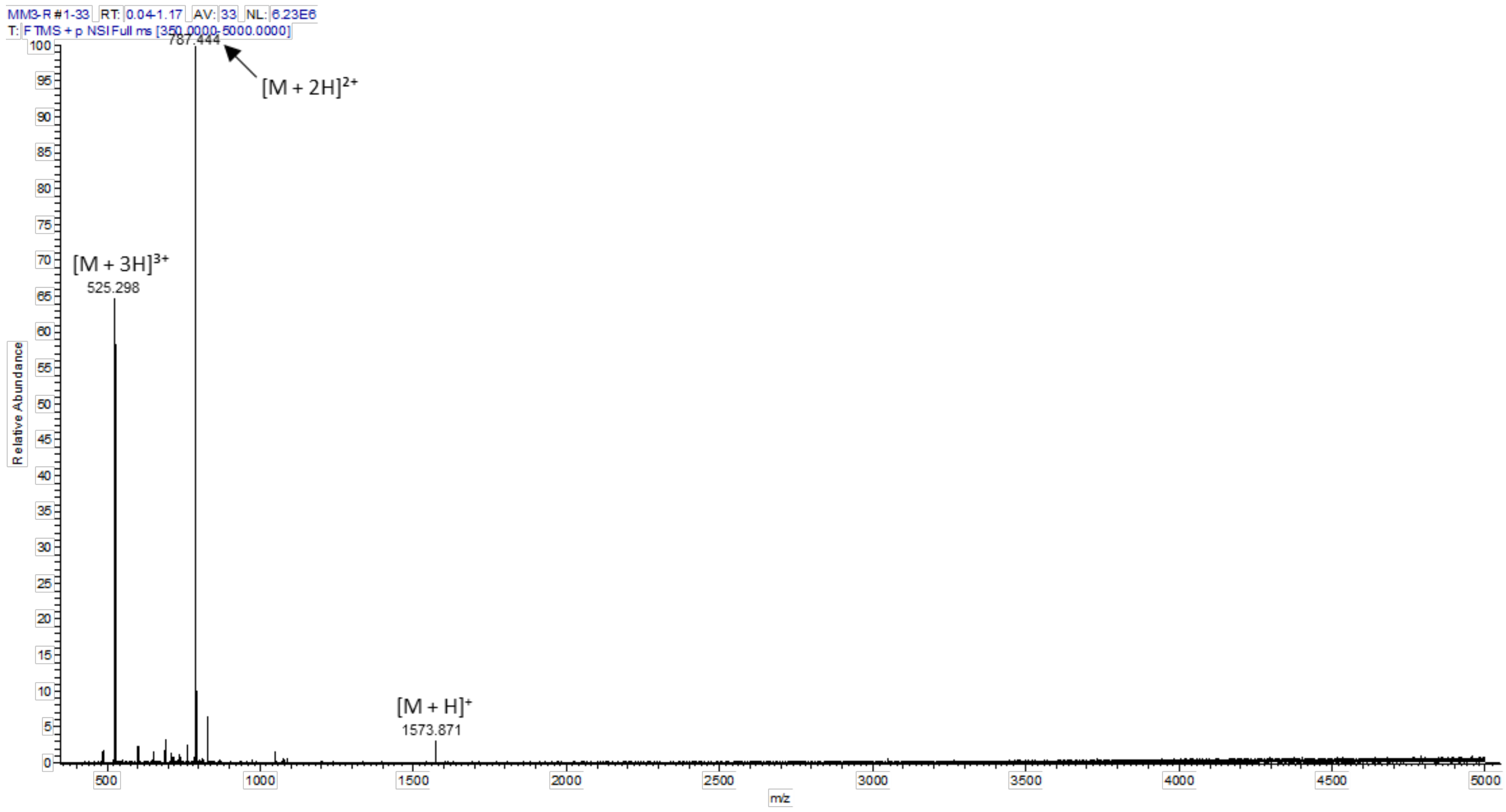

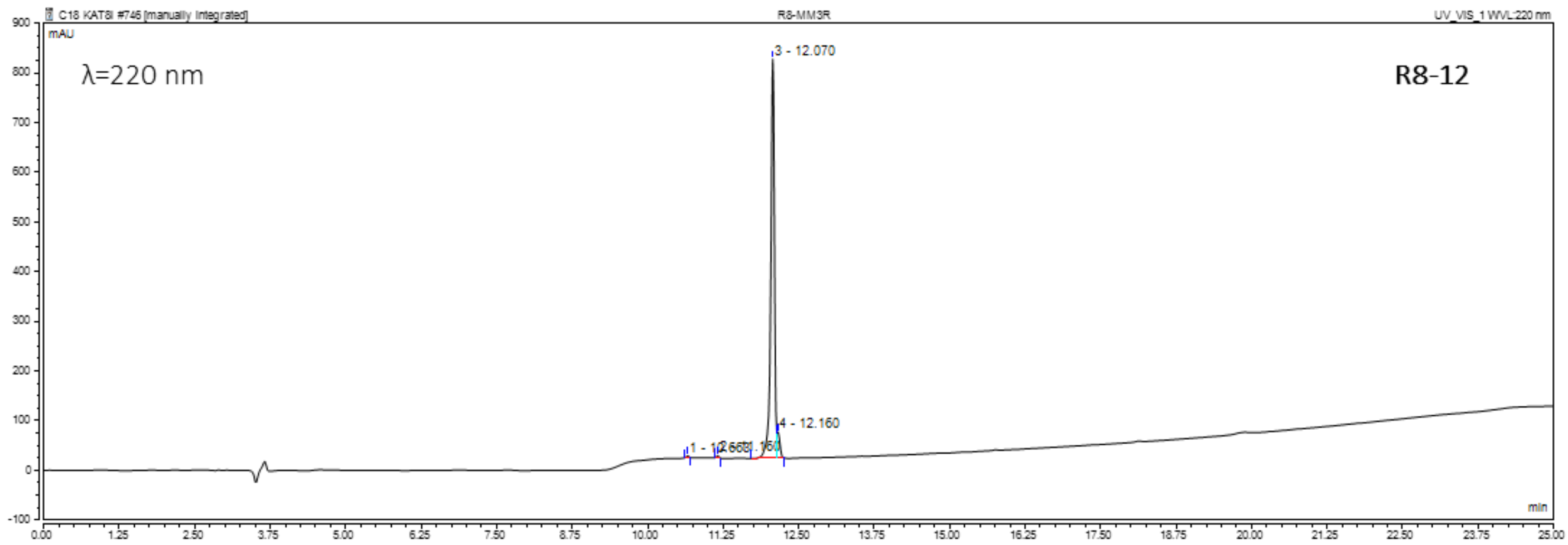

Peak n.	Retention Time (min)	Rel. Area (%)	Area (mAU*min)	Height (mAU)
1	10.663	0.35	0.2099	4.06
2	11.16	0.37	0.2206	4.83
3	12.07	95.25	56.7704	803.1
4	12.16	4.03	2.4025	52.21

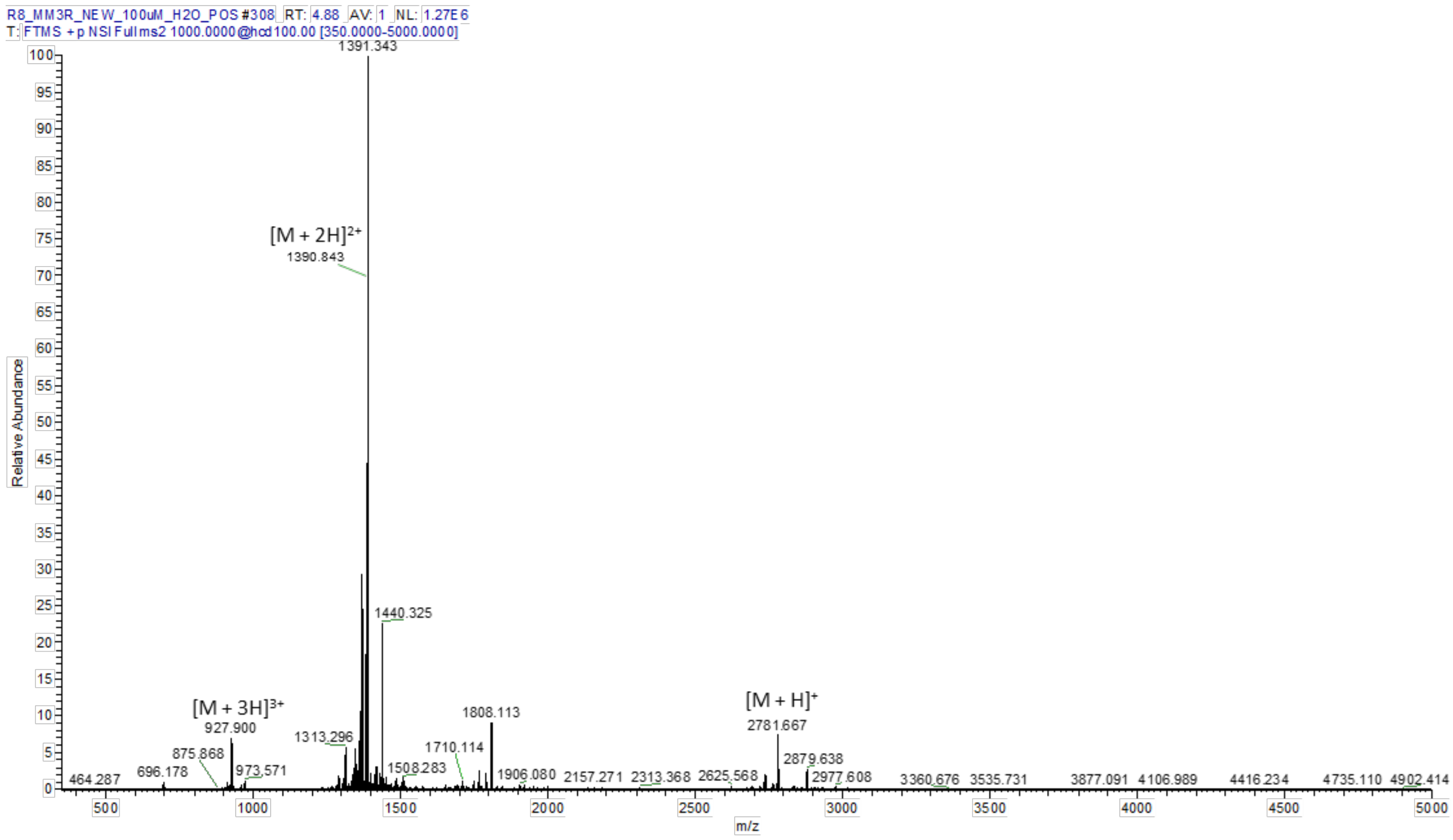

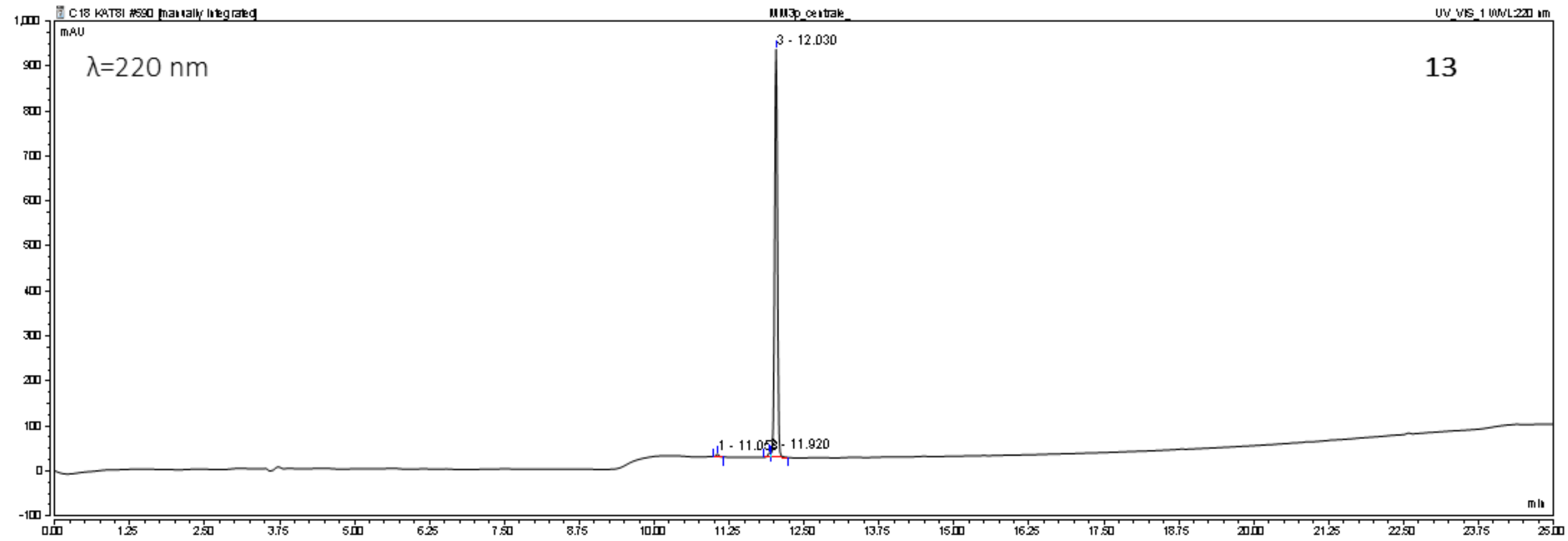

Peak n.	Retention Time (min)	Rel. Area (%)	Area (mAU*min)	Height (mAU)
1	11.053	0.67	0.3344	6.5
2	11.92	1.1	0.5497	10.26
3	12.03	98.23	48.9315	907.2

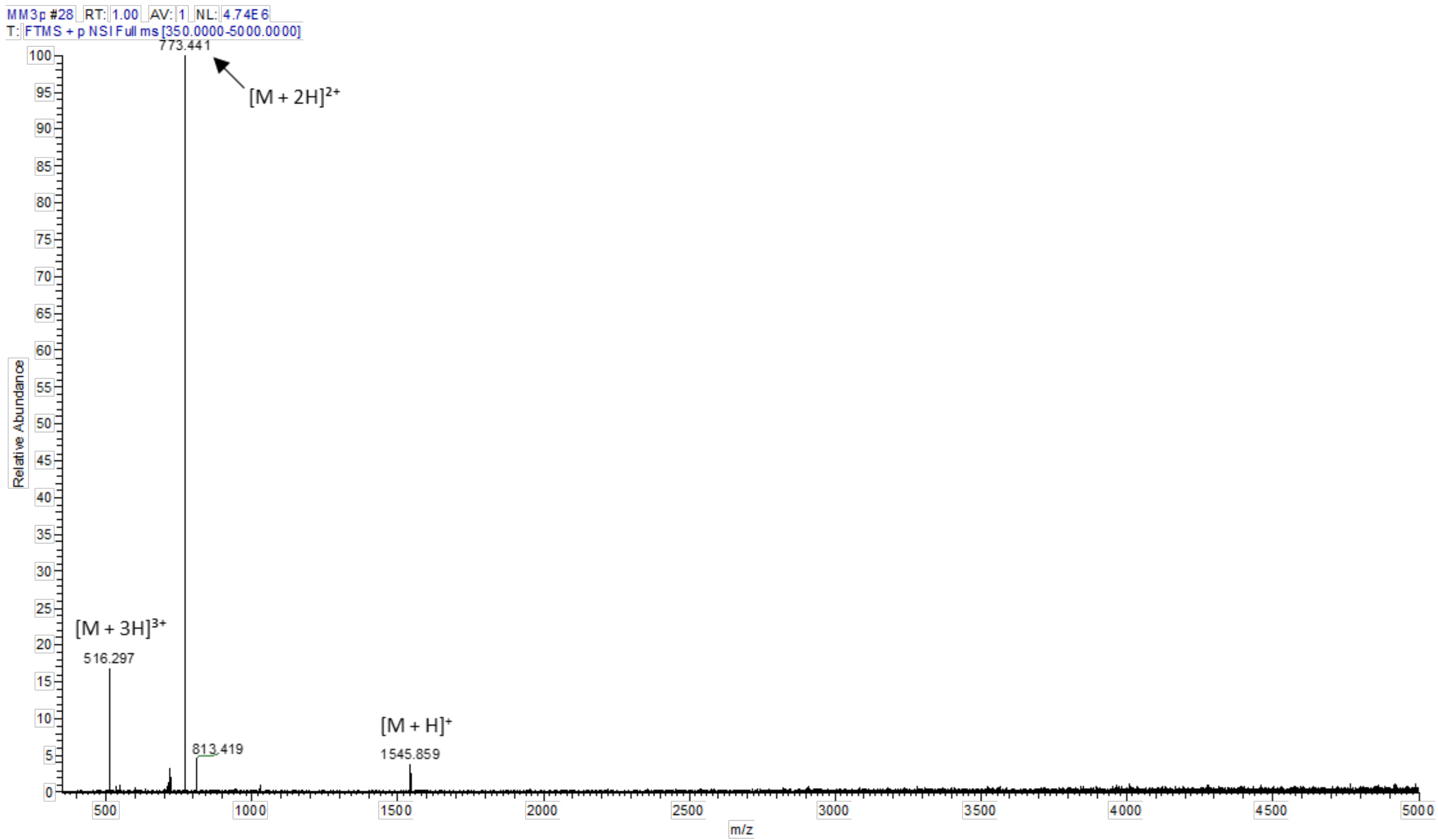

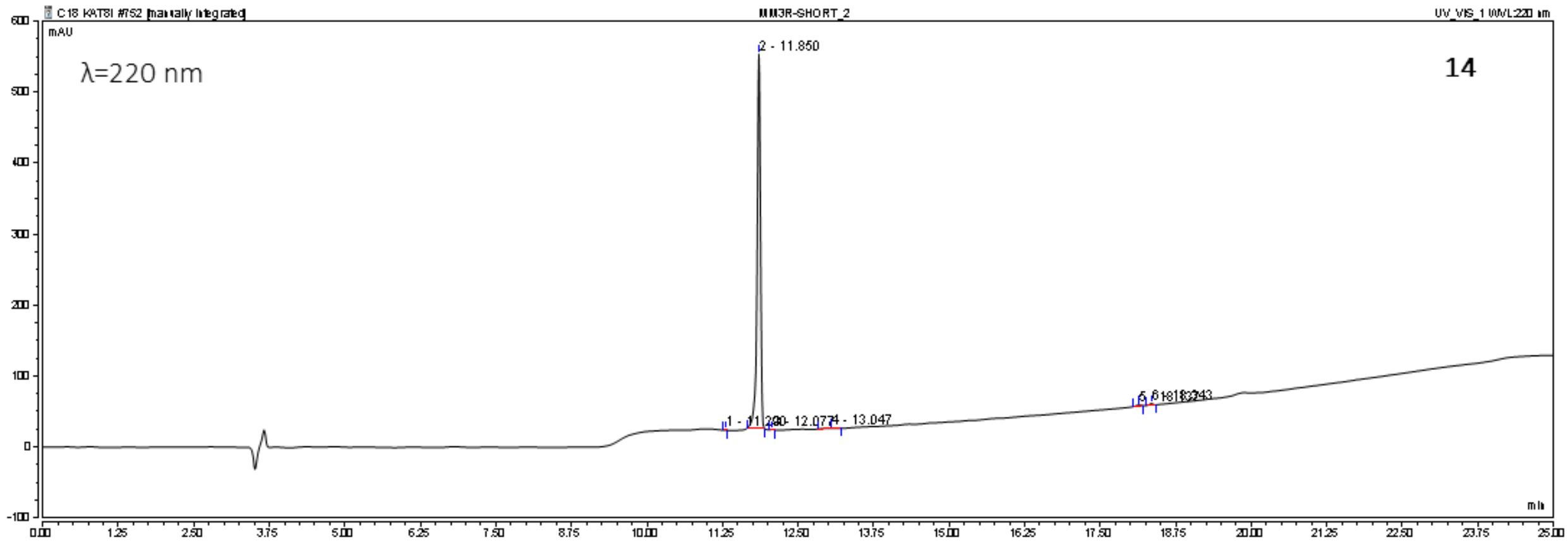

Peak n.	Retention Time (min)	Rel. Area (%)	Area (mAU*min)	Height (mAU)
1	11.29	0.04	0.0124	0.3
2	11.85	98.58	33.5377	527.98
3	12.077	0.1	0.0327	0.69
4	13.047	0.53	0.1796	0.7
5	18.137	0.33	0.1129	1.29
6	18.343	0.43	0.1449	2.28

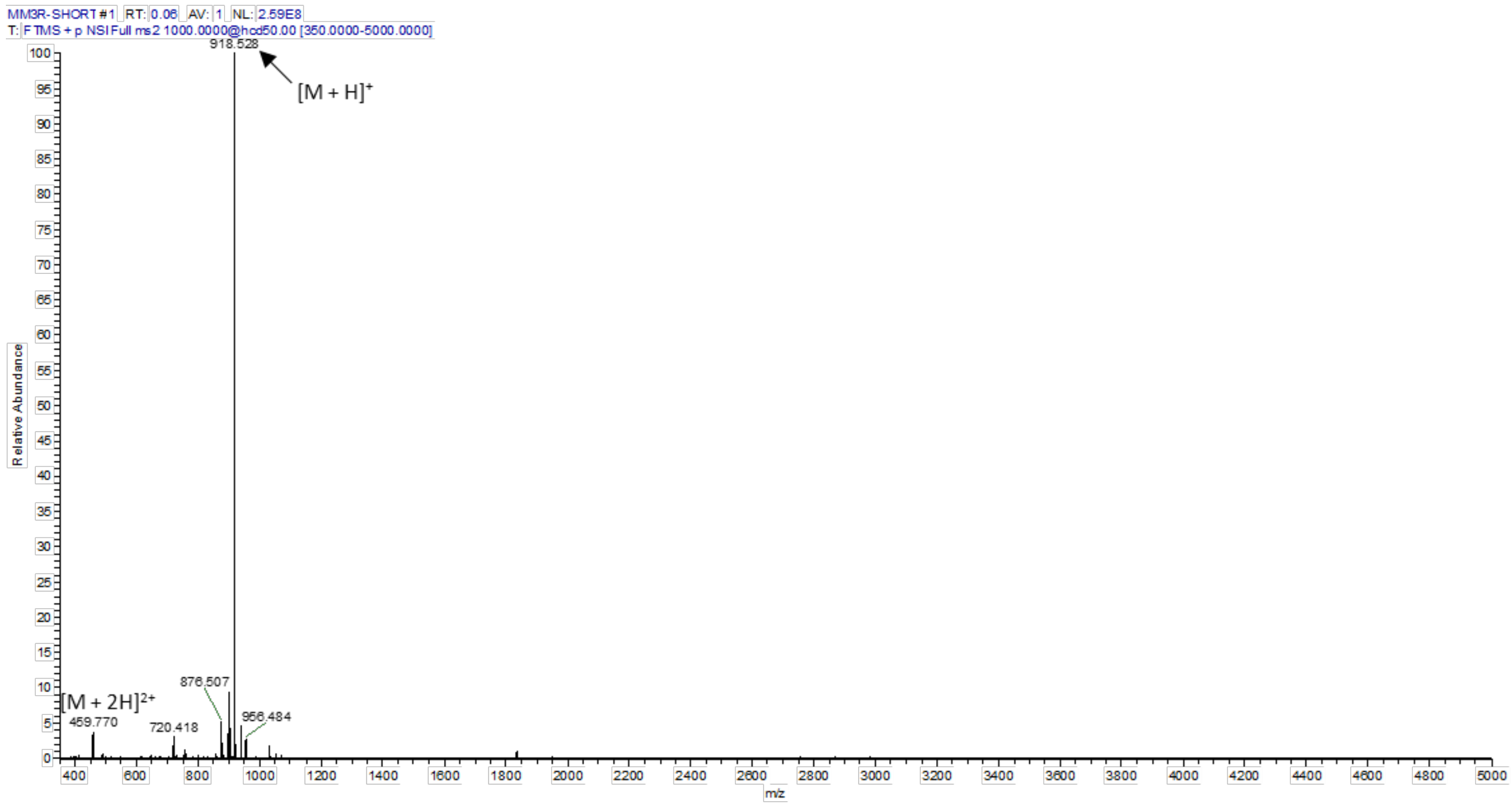

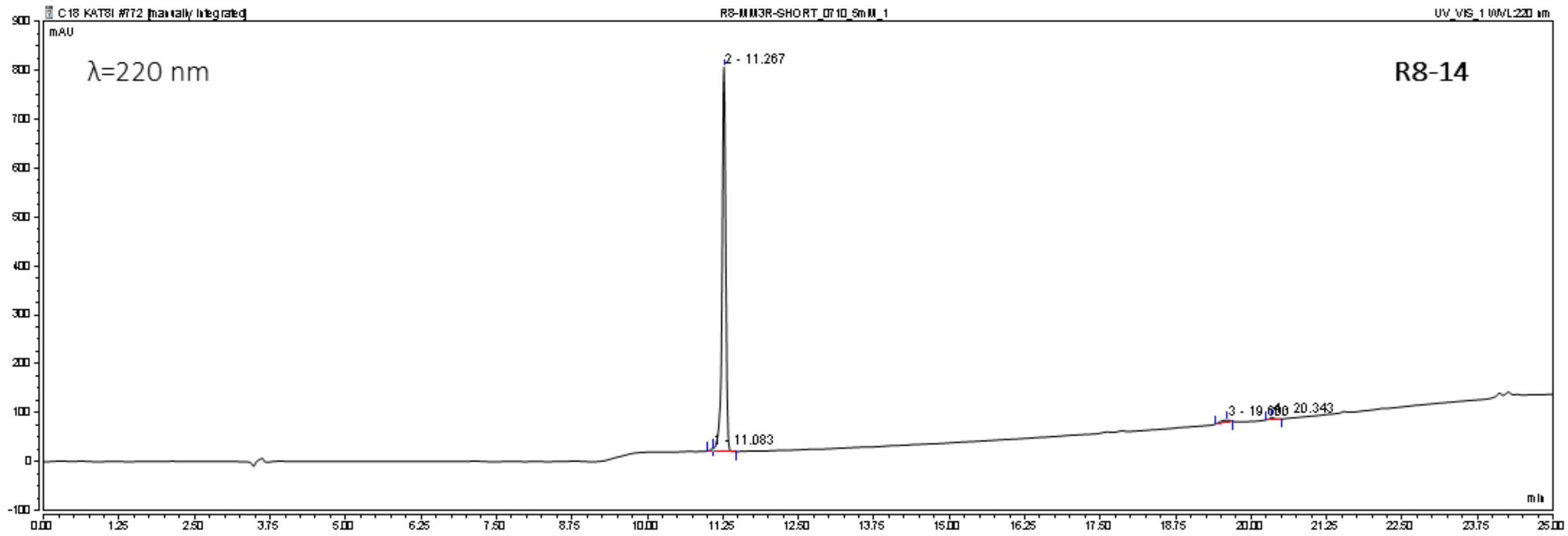

Peak n.	Retention Time (min)	Rel. Area (%)	Area (mAU*min)	Height (mAU)
1	11.083	0.48	0.2731	5.95
2	11.267	97.27	55.0046	785.35
3	19.6	1.61	0.9126	4.31
4	20.343	0.64	0.3595	4.25

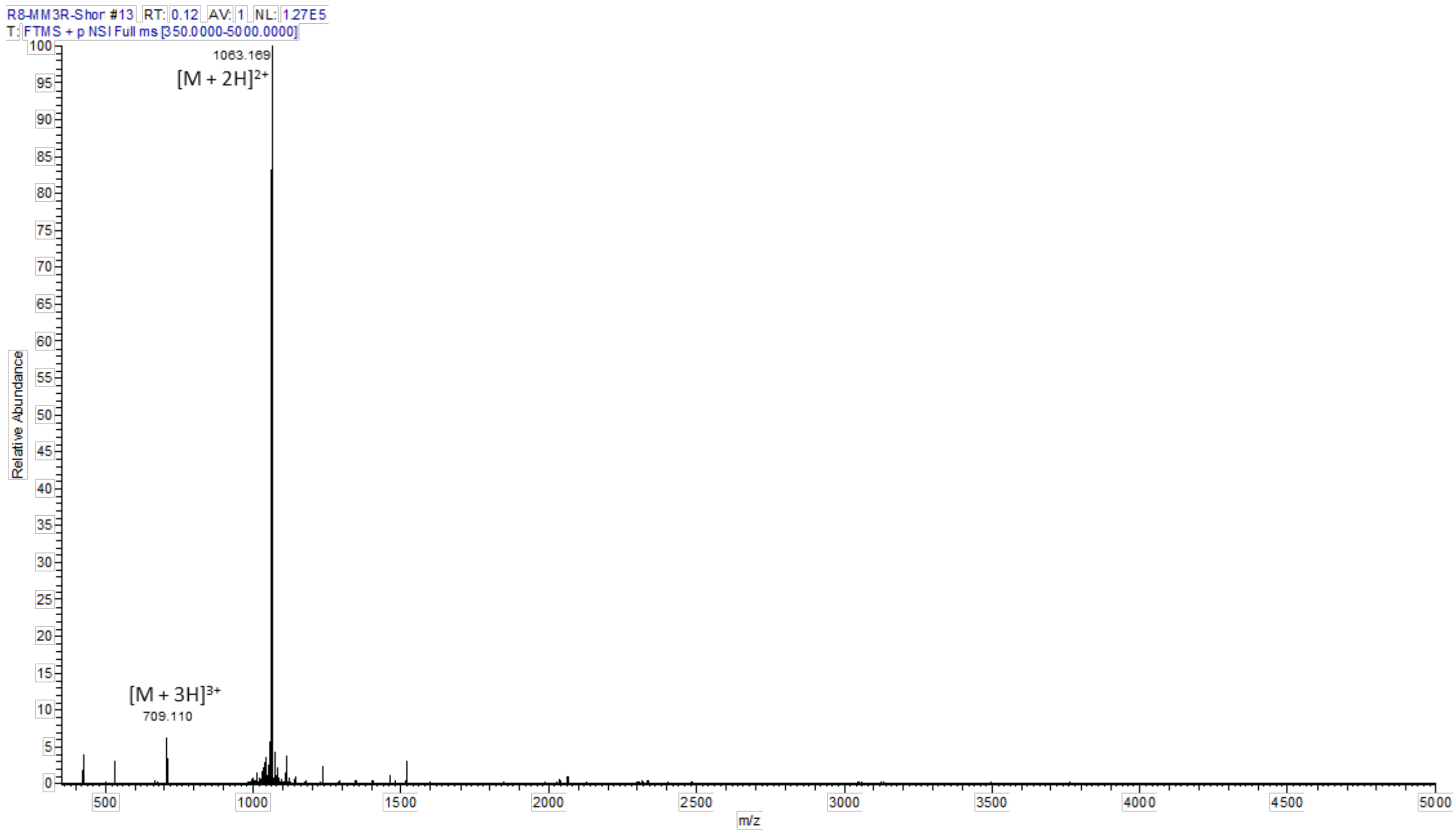

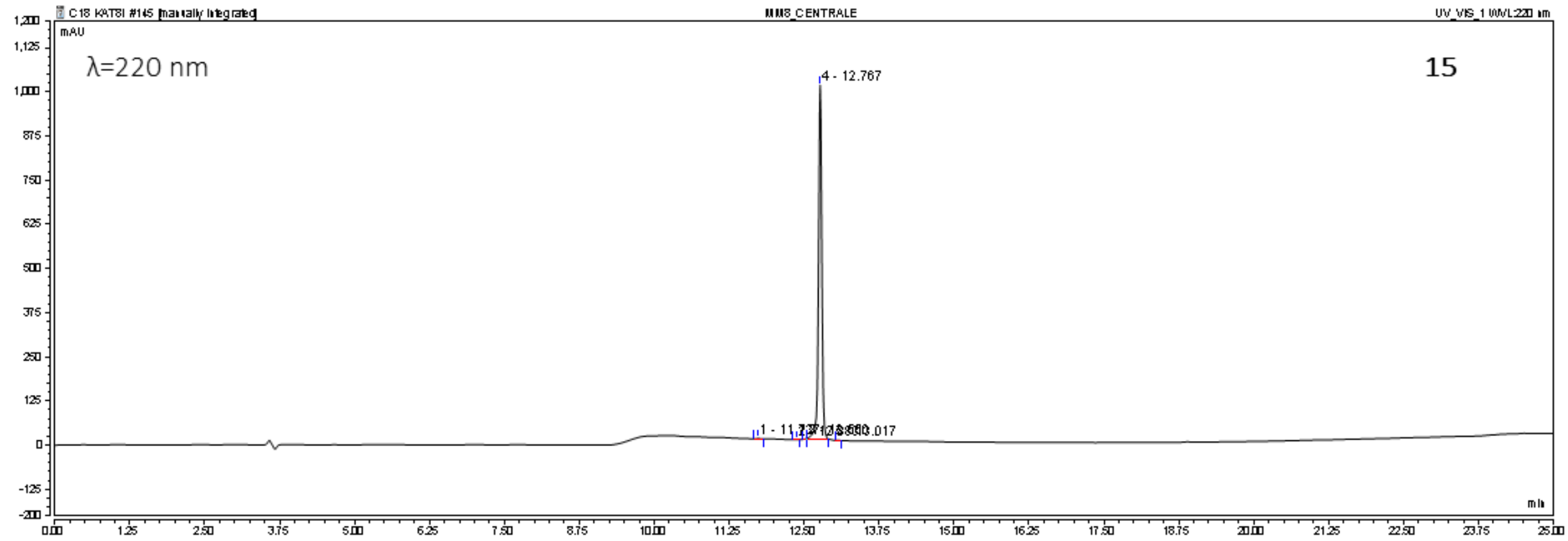

Peak n.	Retention Time (min)	Rel. Area (%)	Area (mAU*min)	Height (mAU)
1	11.737	0.12	0.077	1.58
2	12.383	0.04	0.0268	0.47
3	12.55	0.22	0.143	3.3
4	12.767	99.61	65.0611	1002.64
5	13.017	0.02	0.0109	0

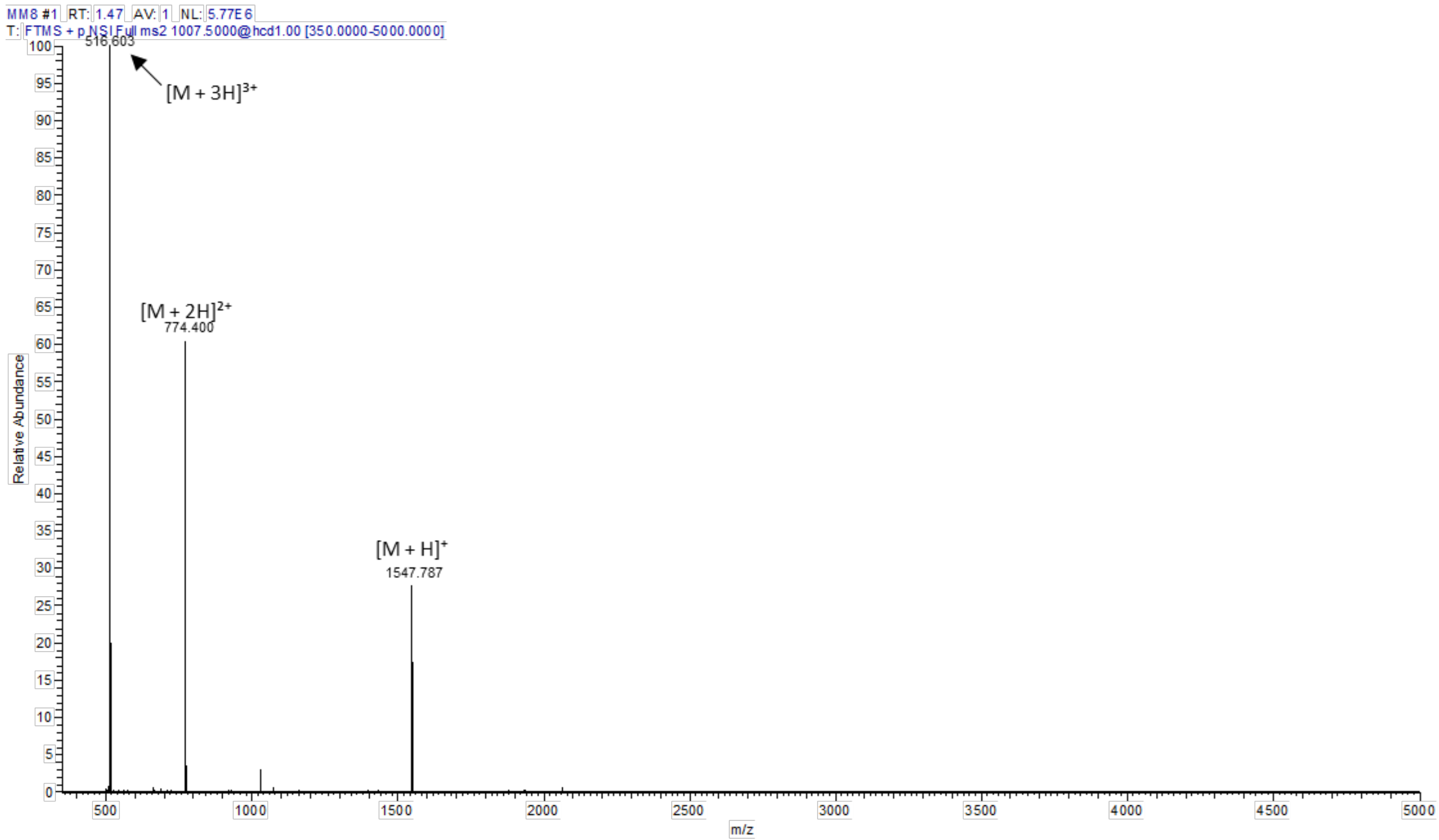

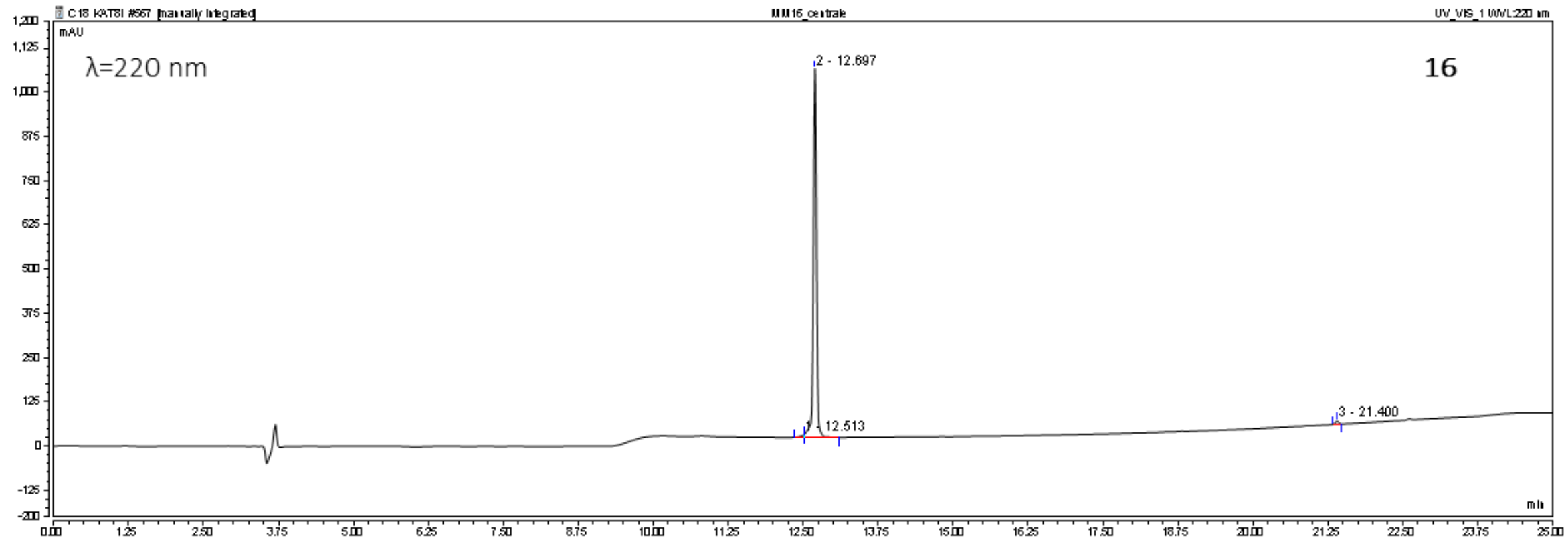

Peak n.	Retention Time (min)	Rel. Area (%)	Area (mAU*min)	Height (mAU)
1	12.513	0.66	0.4321	7.24
2	12.697	98.52	64.4702	1042.37
3	21.4	0.82	0.5388	8.62

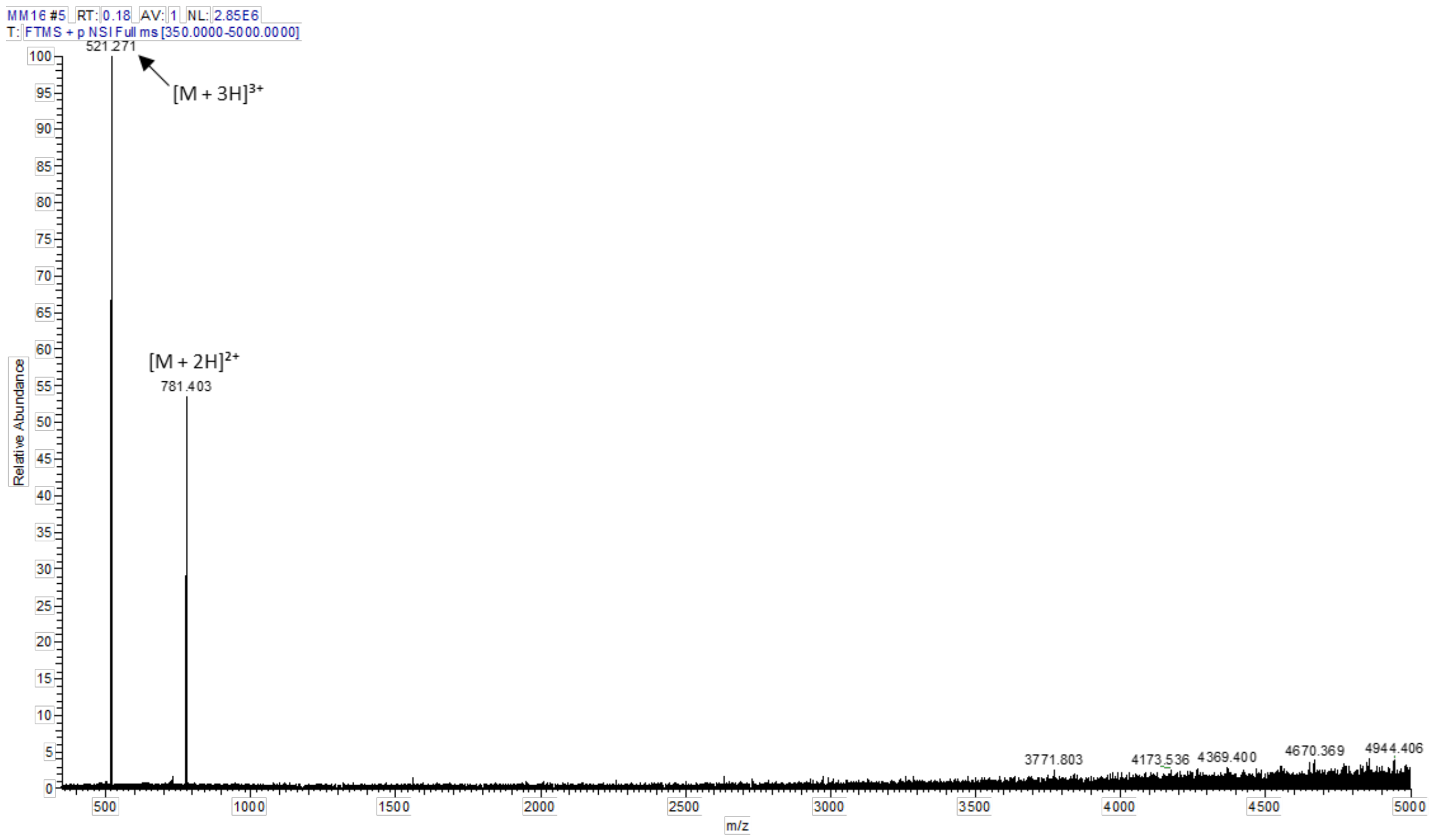

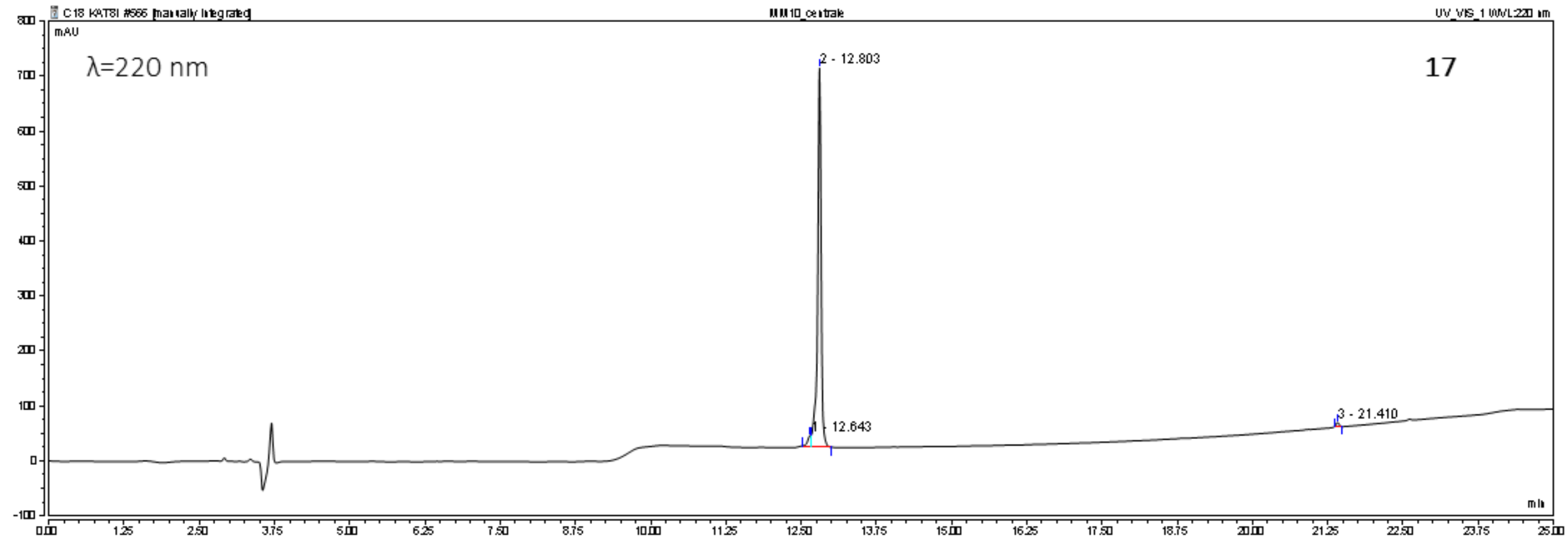

Peak n.	Retention Time (min)	Rel. Area (%)	Area (mAU*min)	Height (mAU)
1	12.643	2.66	1.2787	19.57
2	12.803	96.36	46.2903	688.72
3	21.41	0.98	0.47	7.7

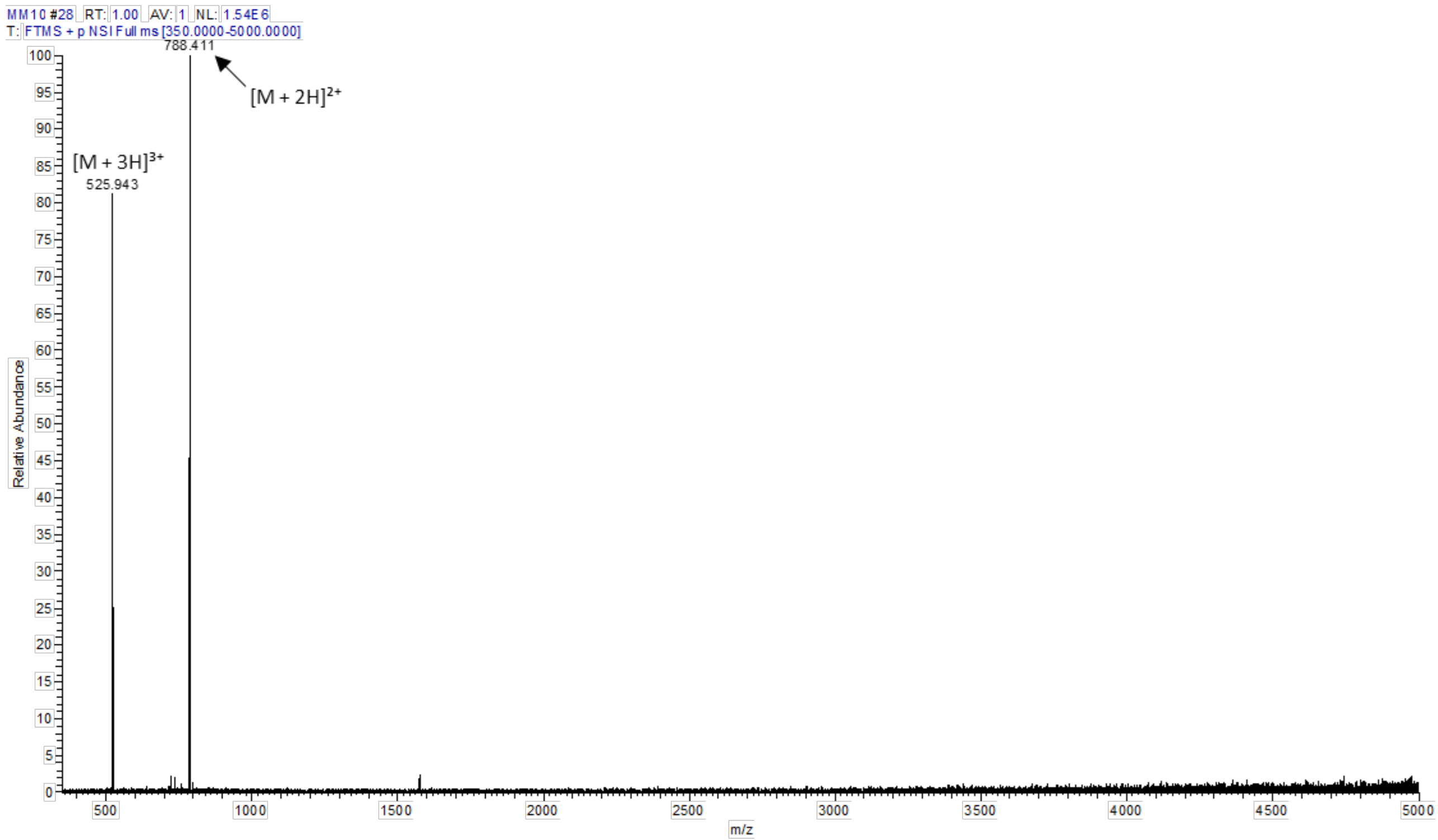

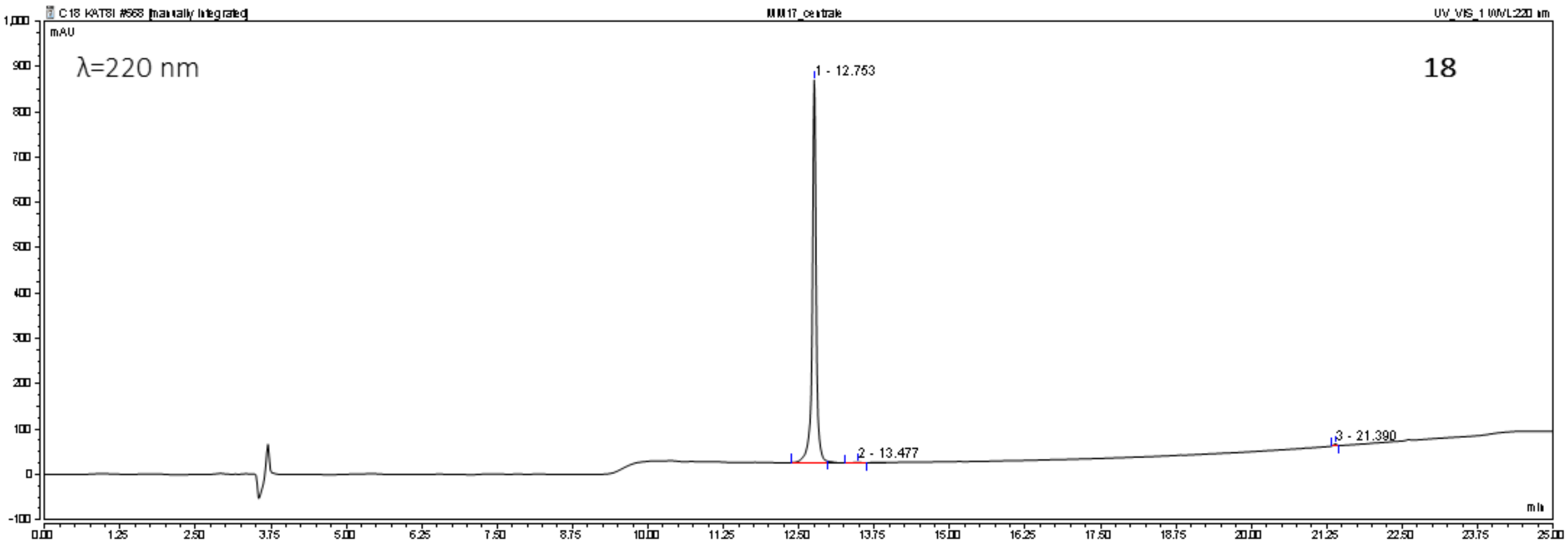

Peak n.	Retention Time (min)	Rel. Area (%)	Area (mAU*min)	Height (mAU)
1	12.753	99.45	63.9115	844.67
2	13.477	0.19	0.1224	0.6
3	21.39	0.36	0.23	3.9

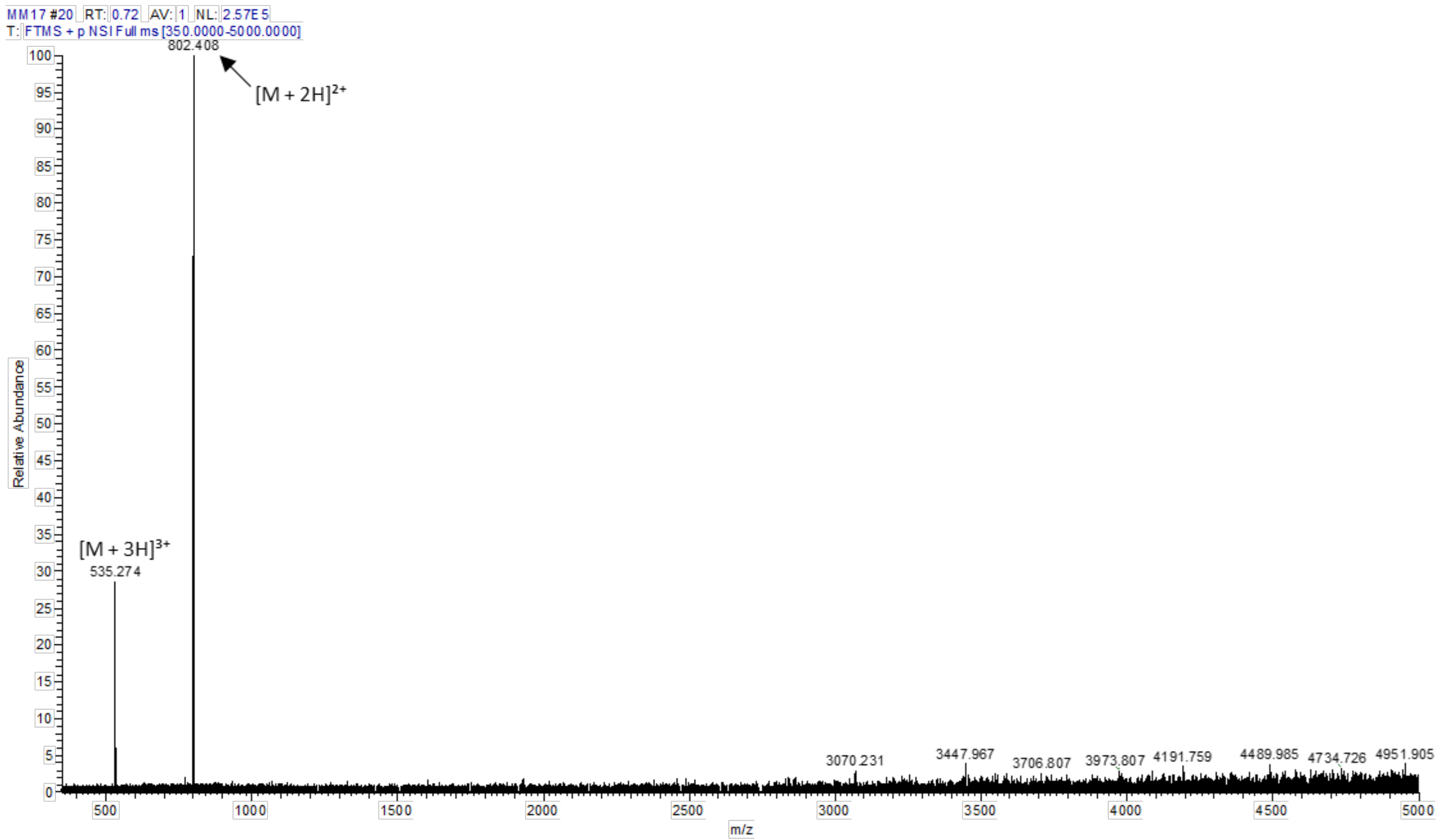

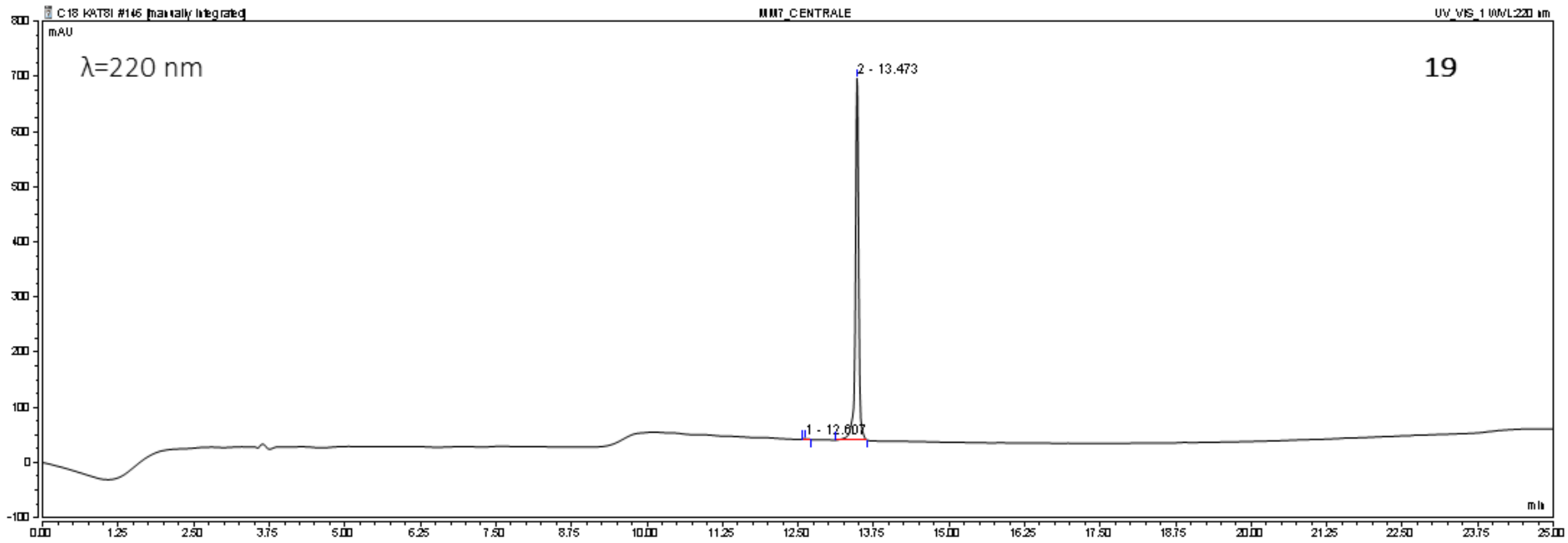

Peak n.	Retention Time (min)	Rel. Area (%)	Area (mAU*min)	Height (mAU)
1	12.607	0.29	0.121	1.34
2	13.473	99.71	41.2333	655.59

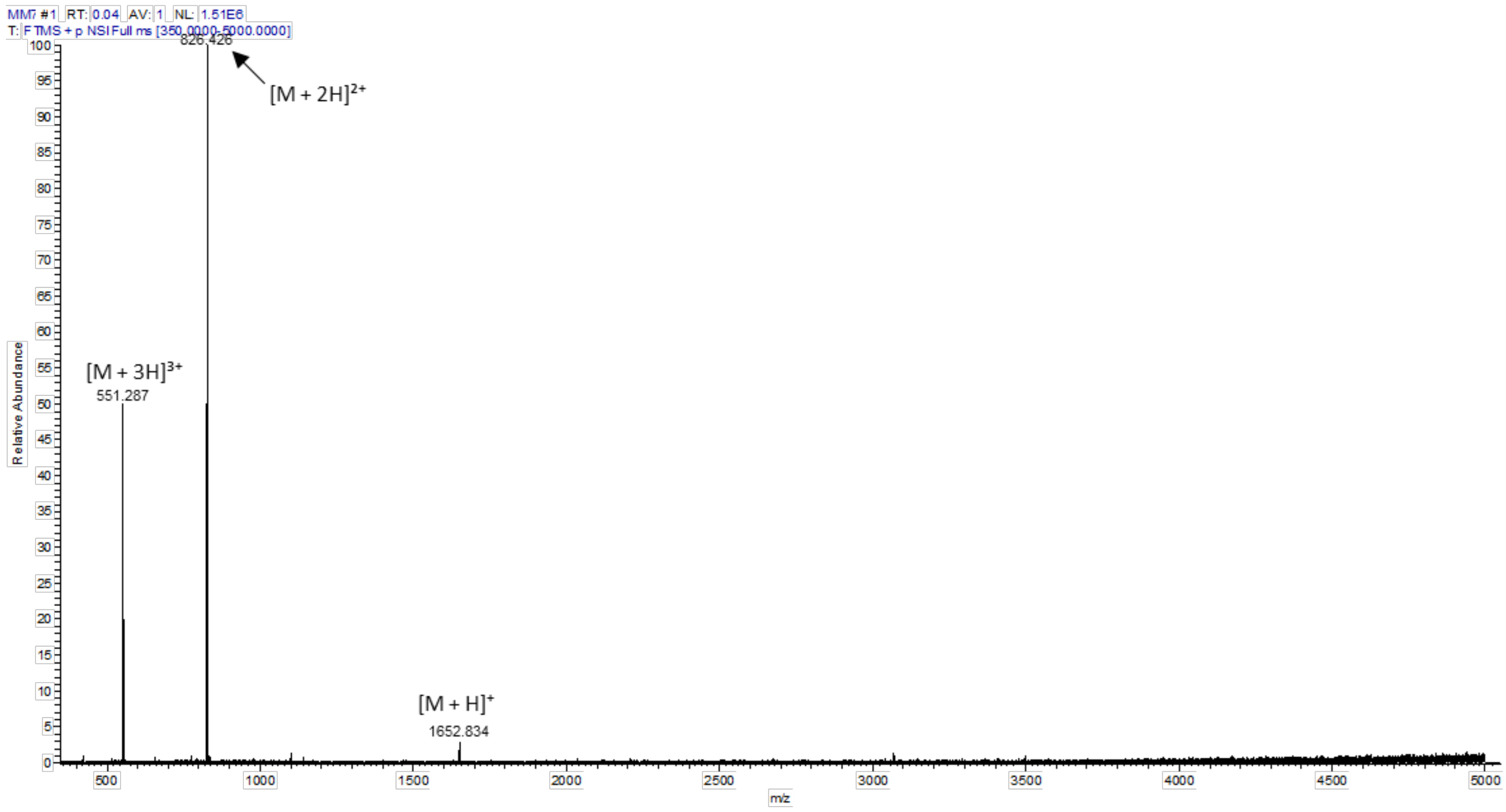

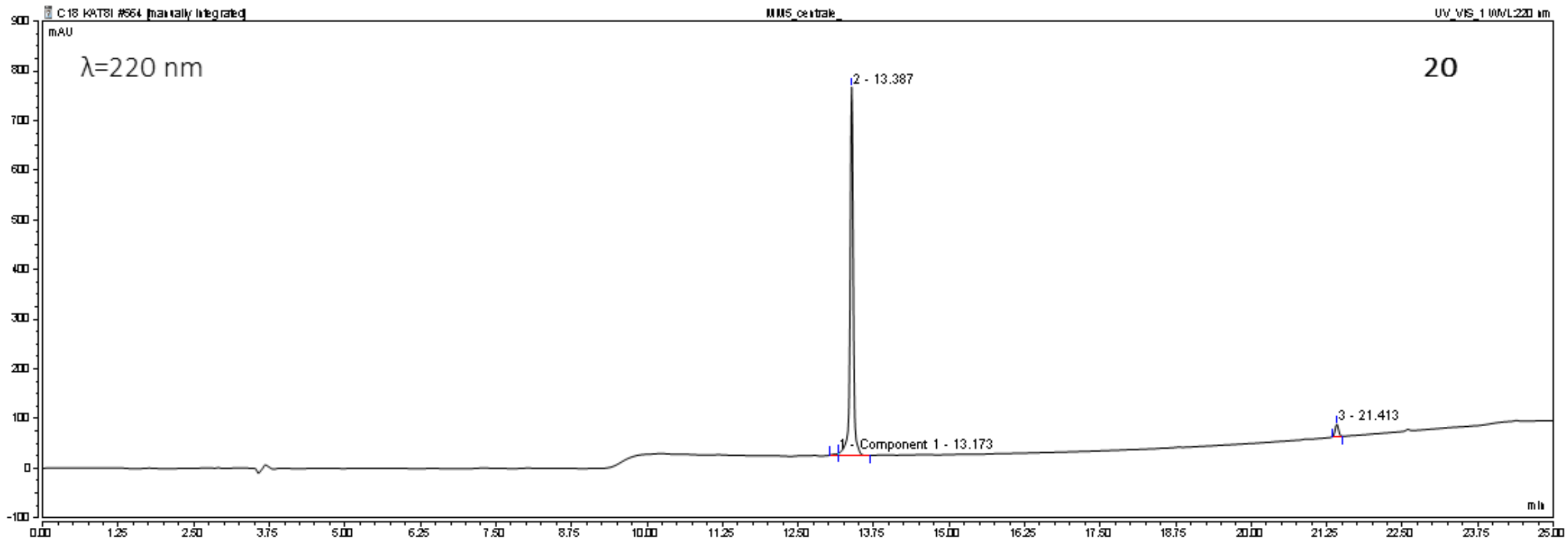

Peak n.	Retention Time (min)	Rel. Area (%)	Area (mAU*min)	Height (mAU)
1	13.173	0.59	0.2821	3.79
2	13.387	96.06	45.7079	741.29
3	21.413	3.35	1.592	25.32

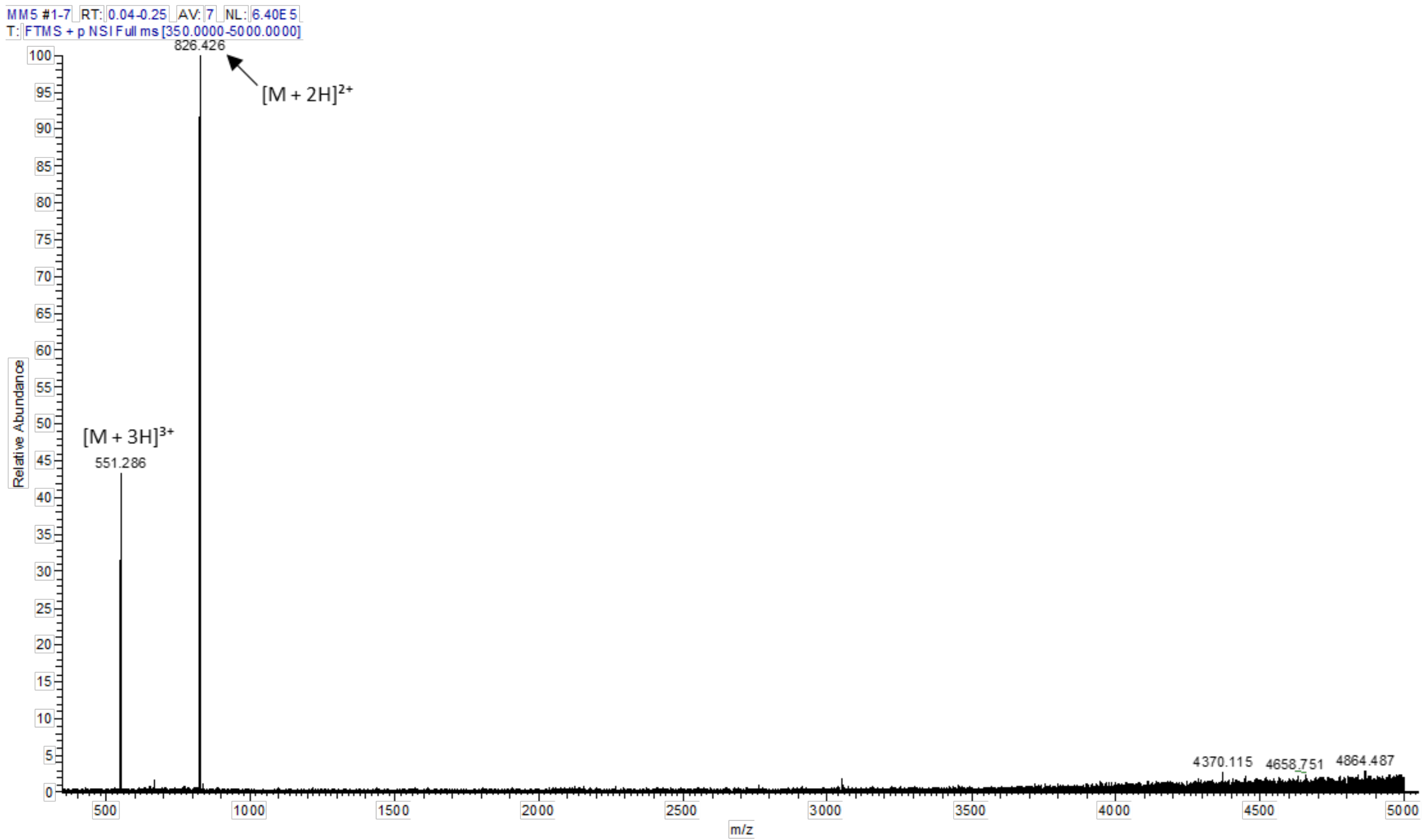

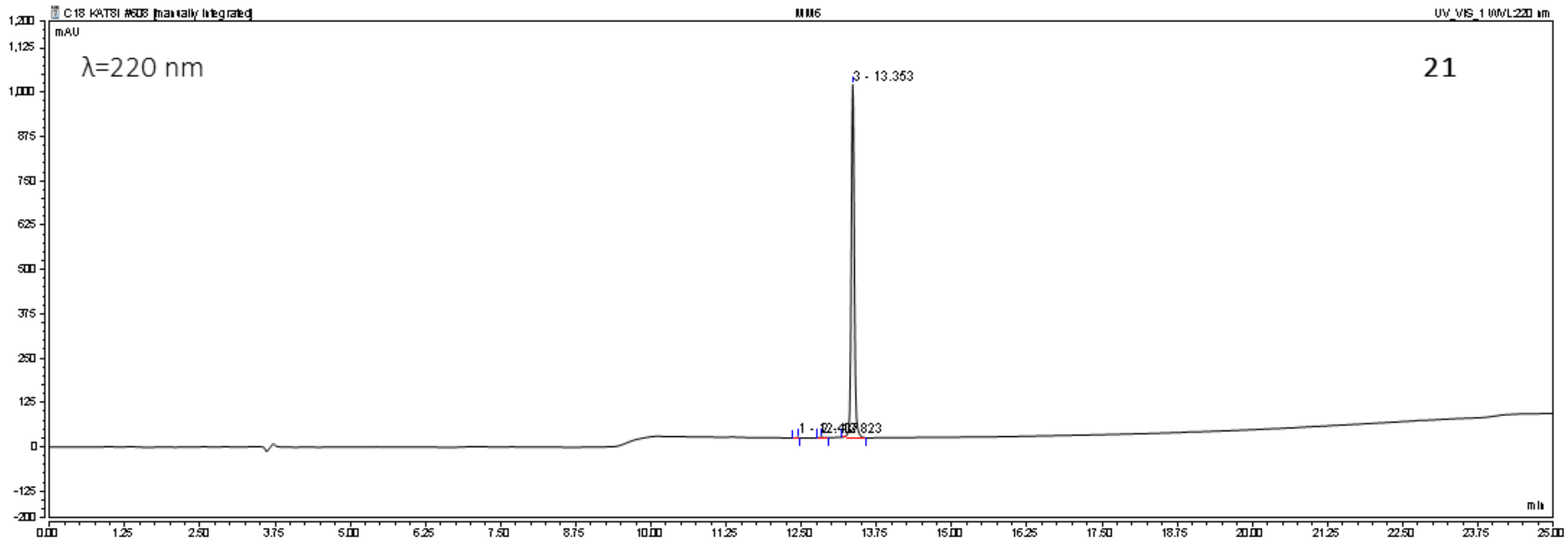

Peak n.	Retention Time (min)	Rel. Area (%)	Area (mAU*min)	Height (mAU)
1	12.437	0.18	0.1037	2.08
2	12.823	0.27	0.1605	1.9
3	13.353	99.55	58.4752	995.19

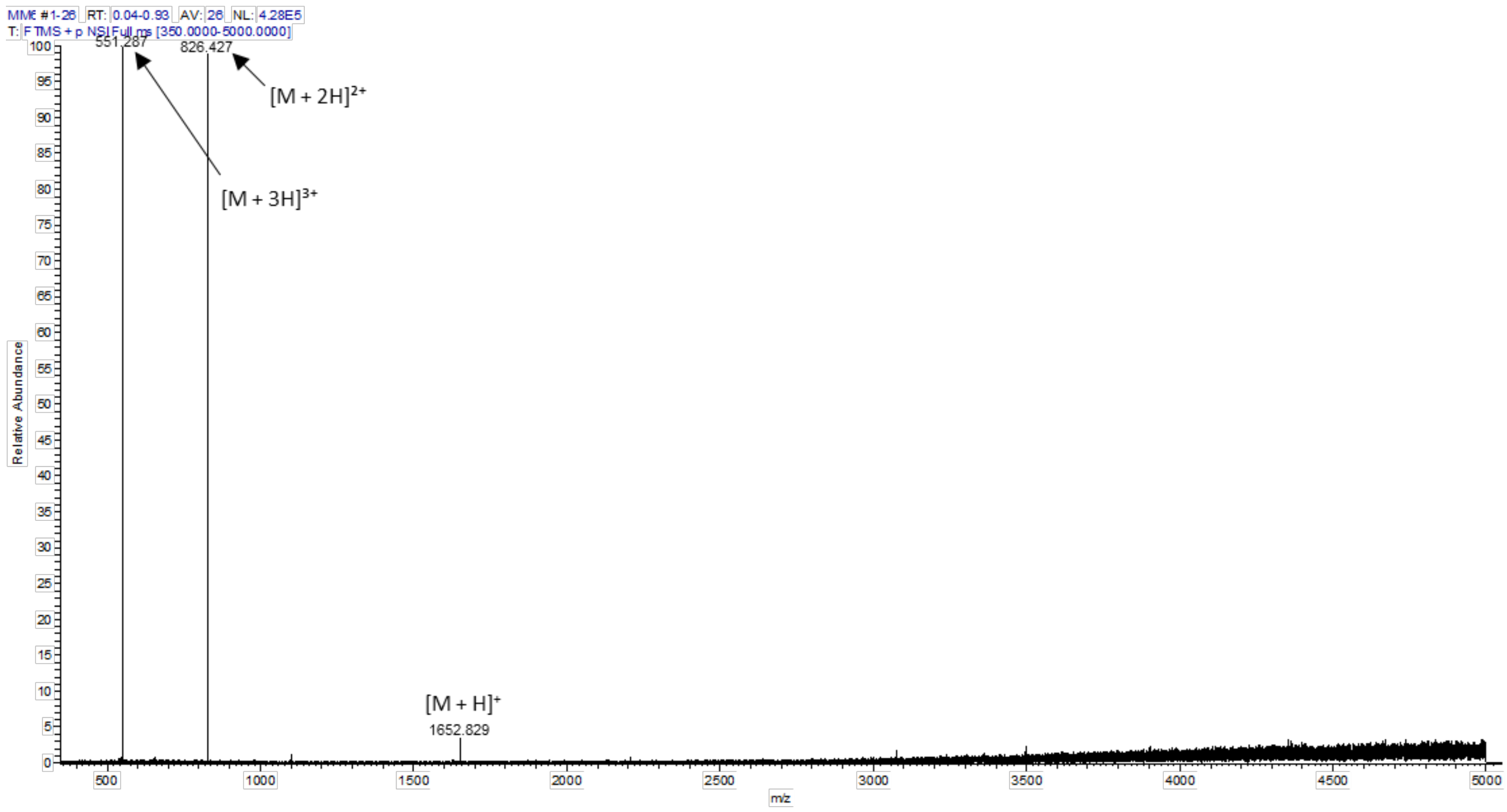

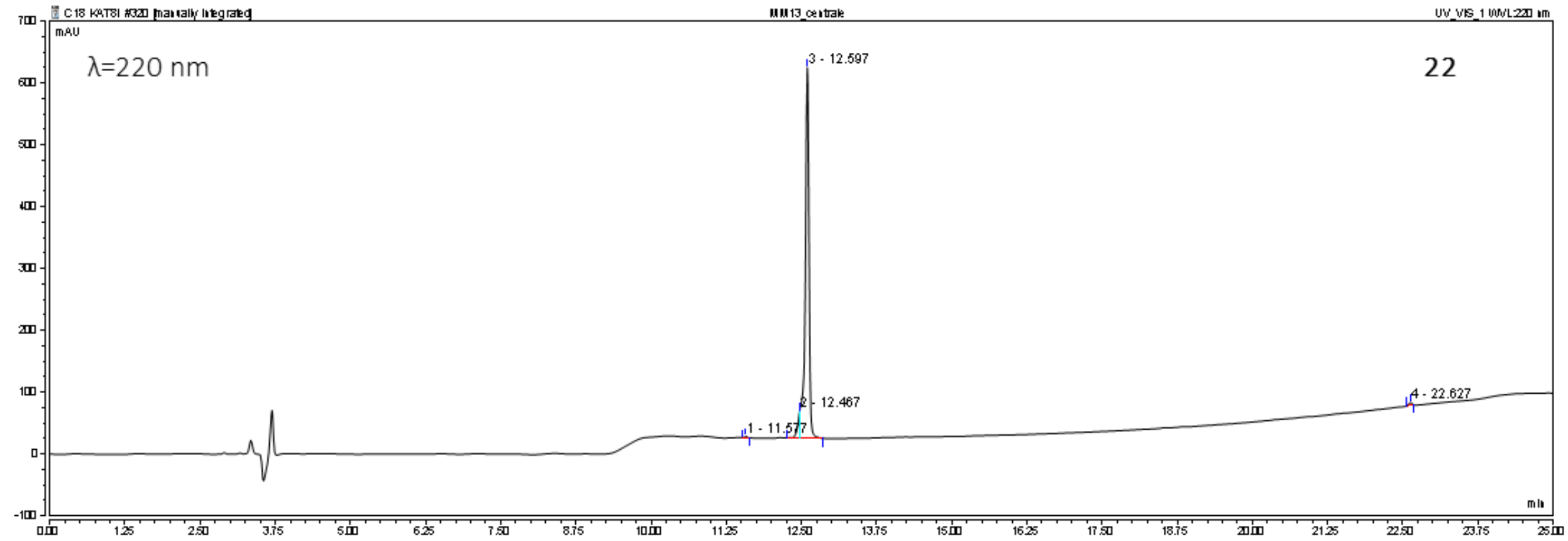

Peak n.	Retention Time (min)	Rel. Area (%)	Area (mAU*min)	Height (mAU)
1	11.577	0.23	0.1009	2.03
2	12.467	3.22	1.4399	42.22
3	12.597	96.56	43.1961	598.07

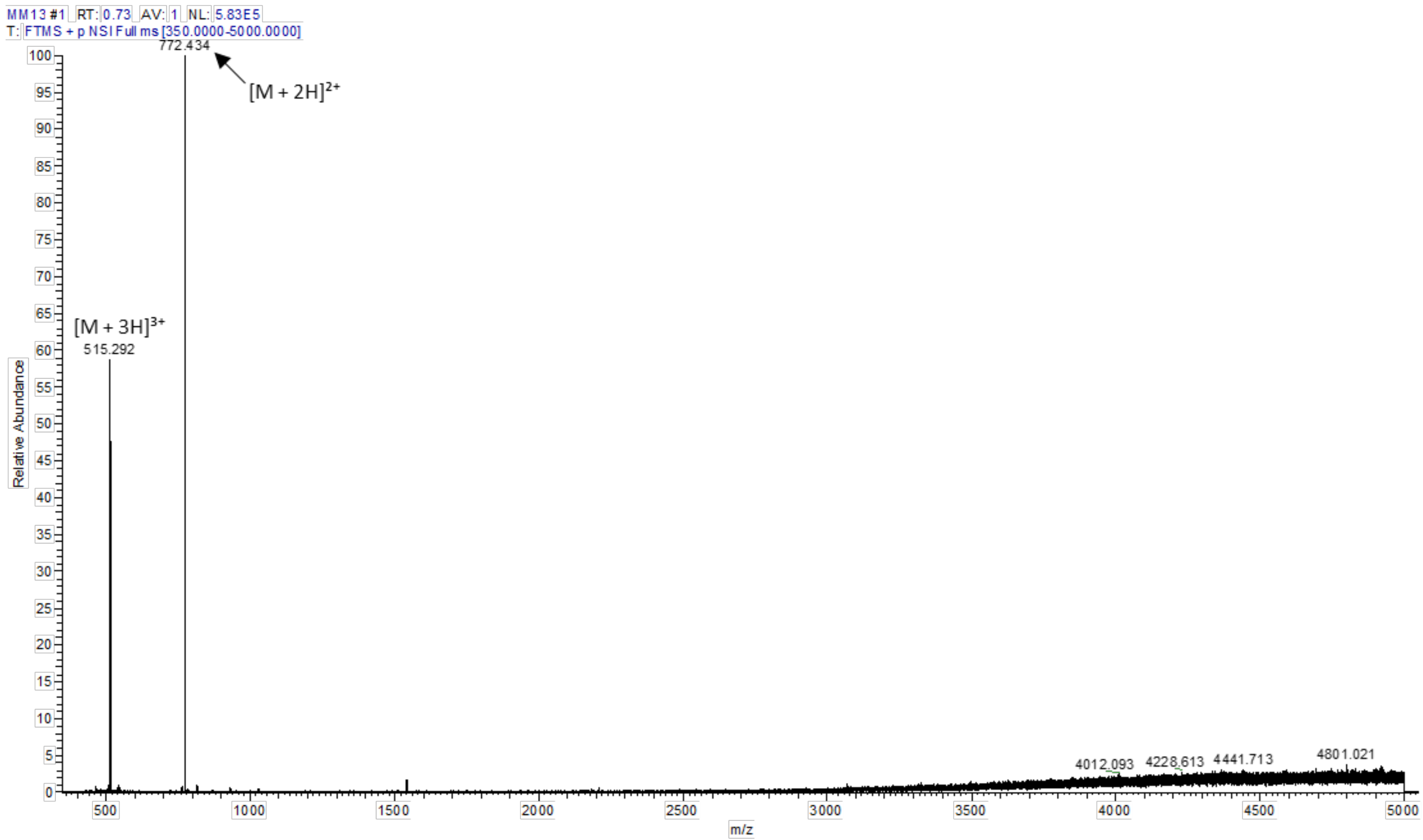

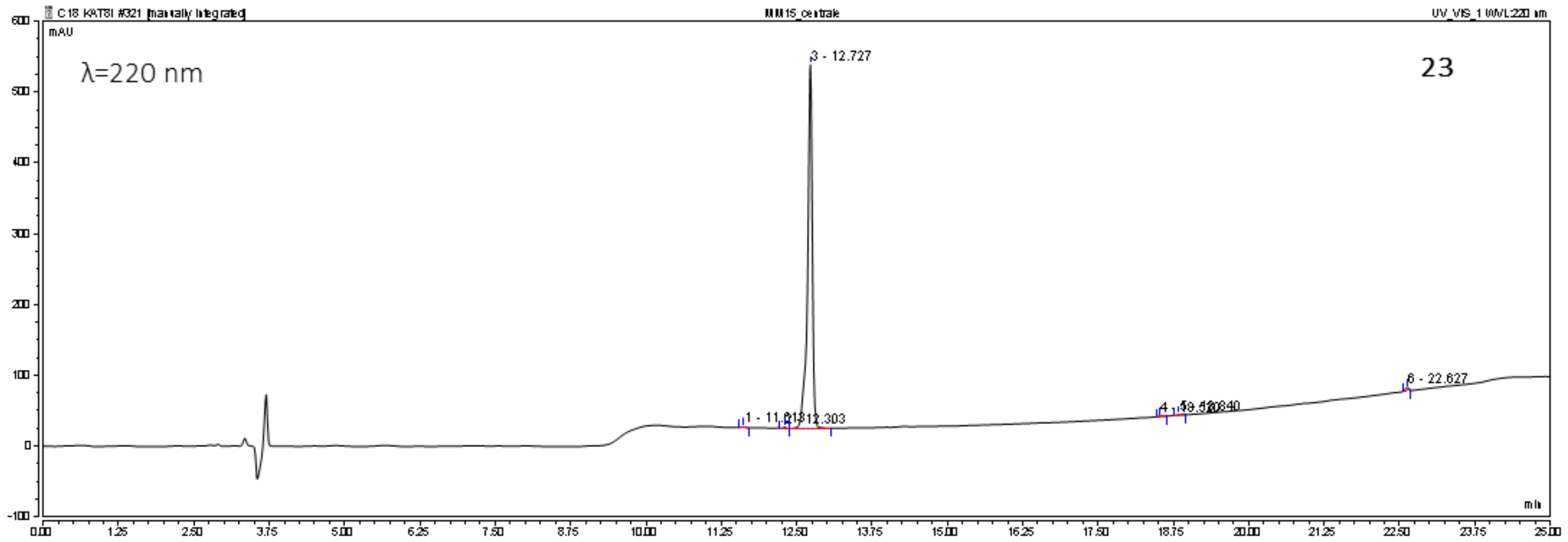

Peak n.	Retention Time (min)	Rel. Area (%)	Area (mAU*min)	Height (mAU)
1	11.613	0.17	0.0816	1.49
2	12.303	0.19	0.0913	1.84
3	12.727	98.81	46.5433	513.86
4	18.52	0.1	0.0473	0.84
5	18.84	0.1	0.0487	0.61
6	22.627	0.62	0.2907	4.67

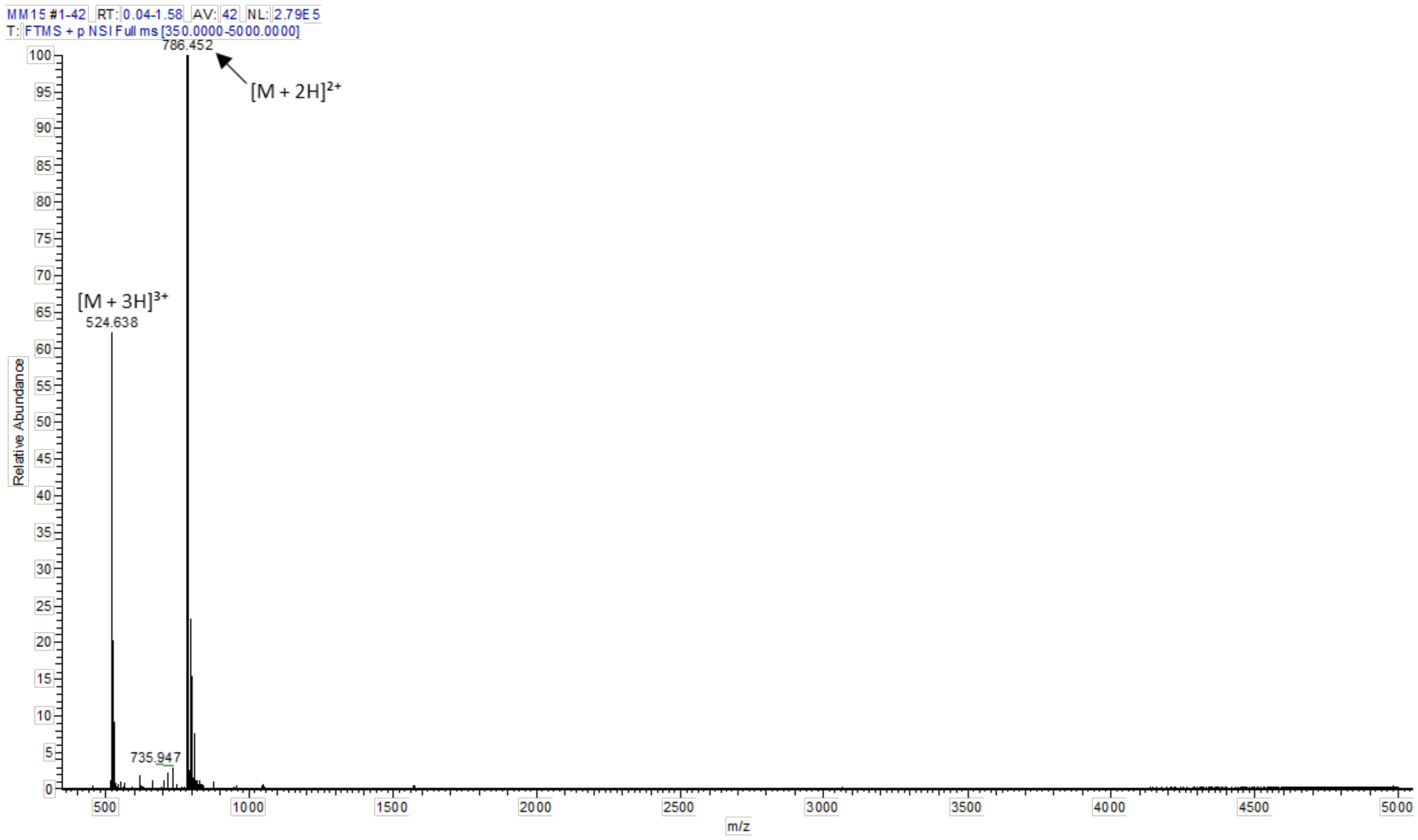

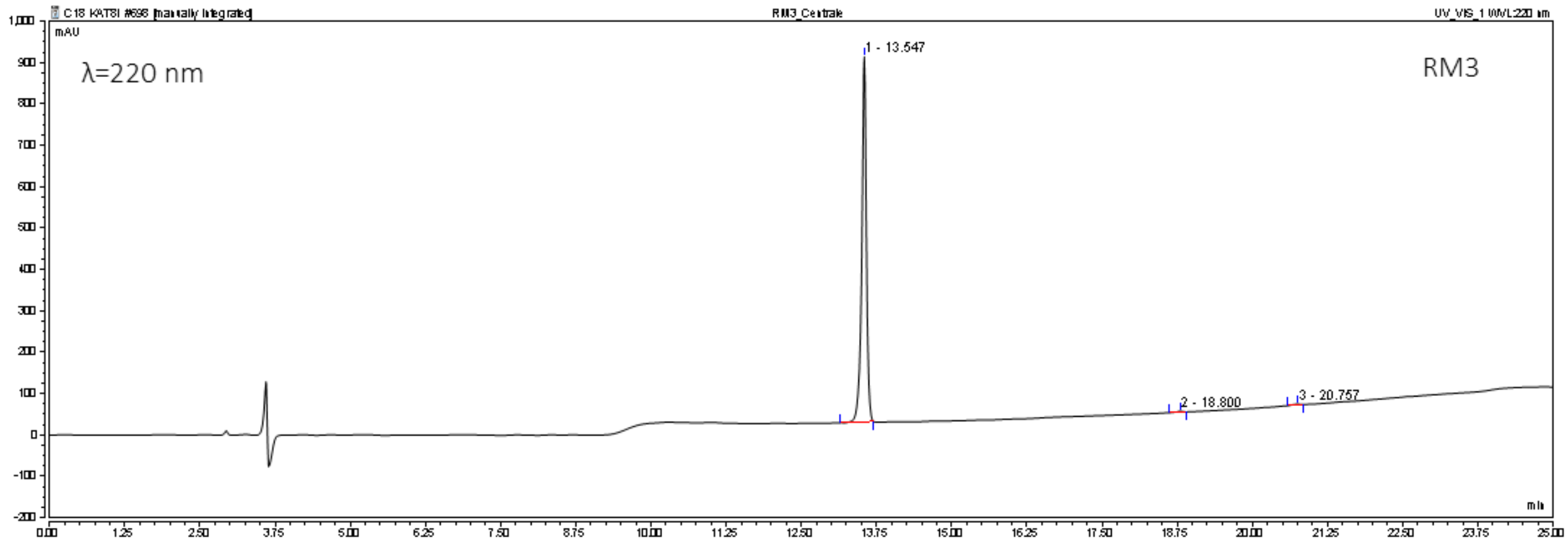

Peak n.	Retention Time (min)	Rel. Area (%)	Area (mAU*min)	Height (mAU)
1	13.547	99.36	78.6589	882.45
2	18.8	0.35	0.2735	2.49
3	20.757	0.29	0.2294	1.26

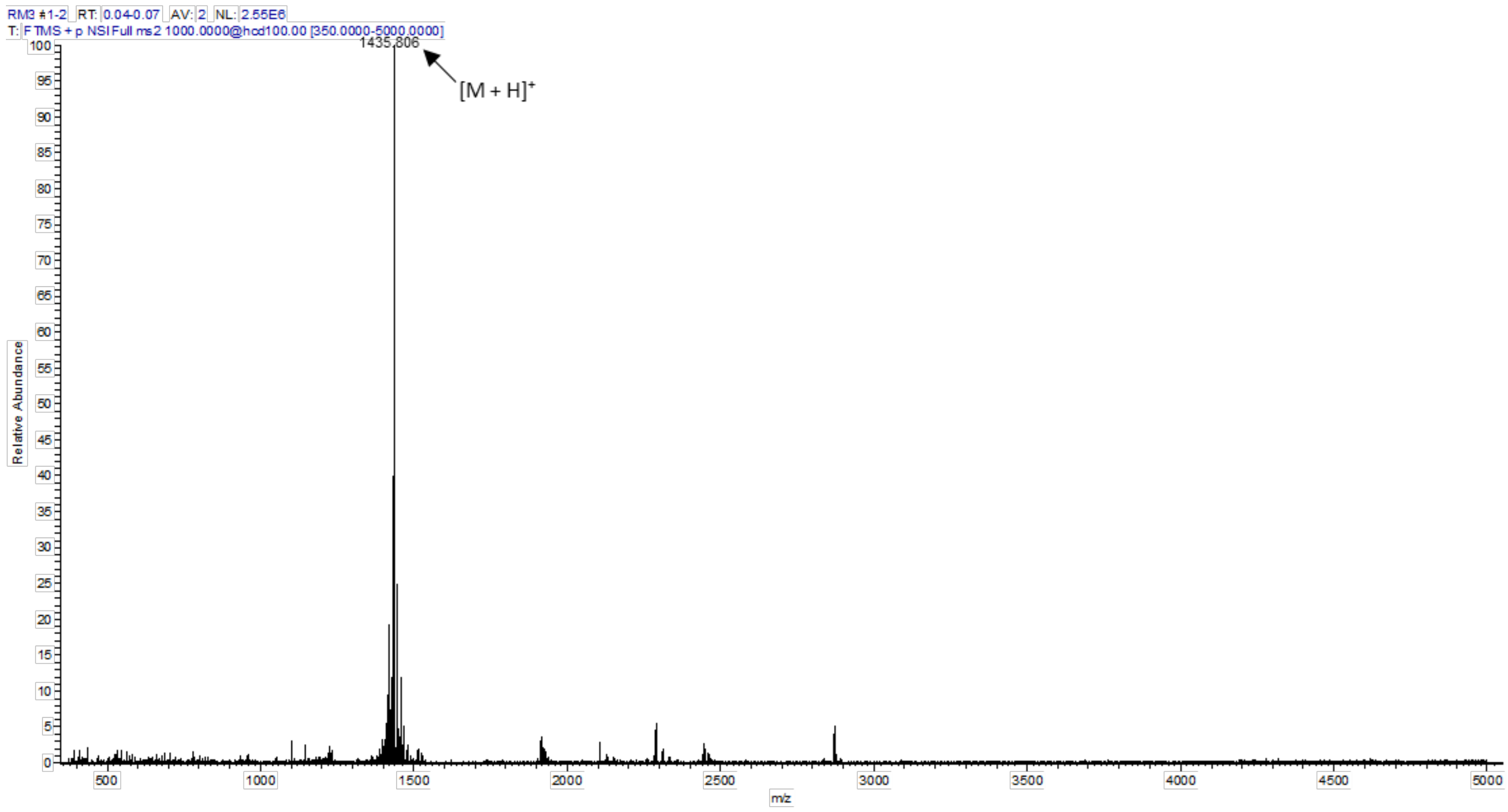

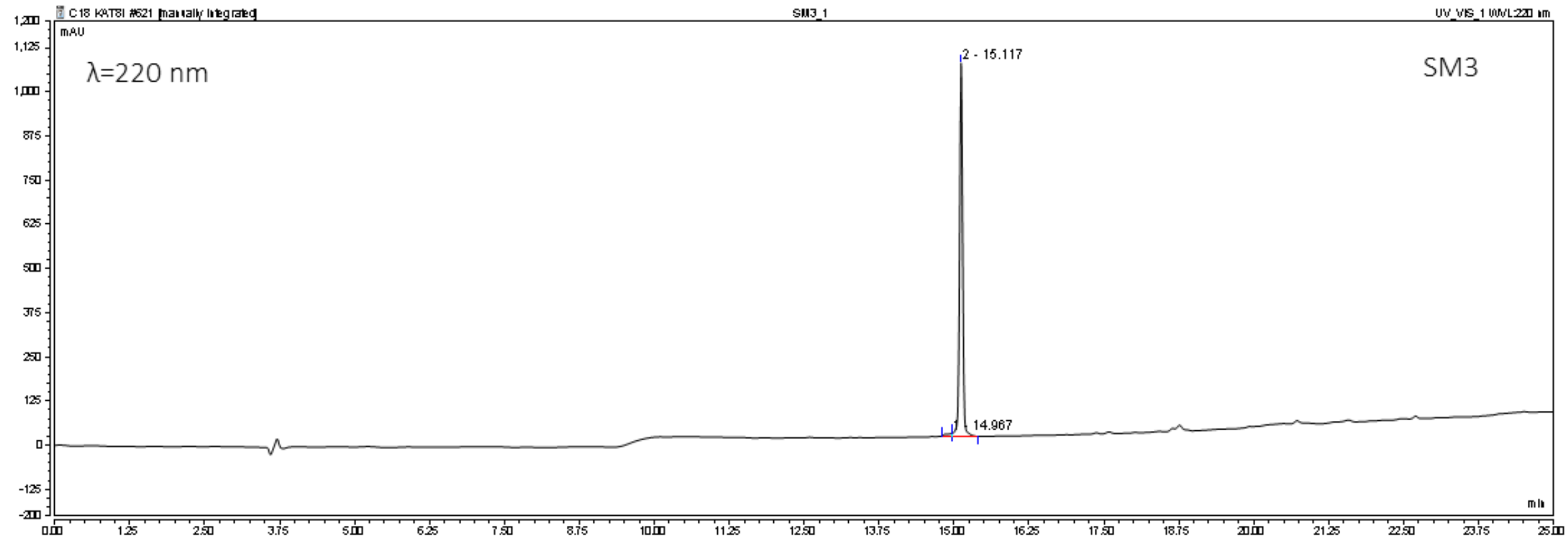

Peak n.	Retention Time (min)	Rel. Area (%)	Area (mAU*min)	Height (mAU)
1	14.967	1.42	0.9129	7.79
2	15.117	98.58	63.47	1056.39

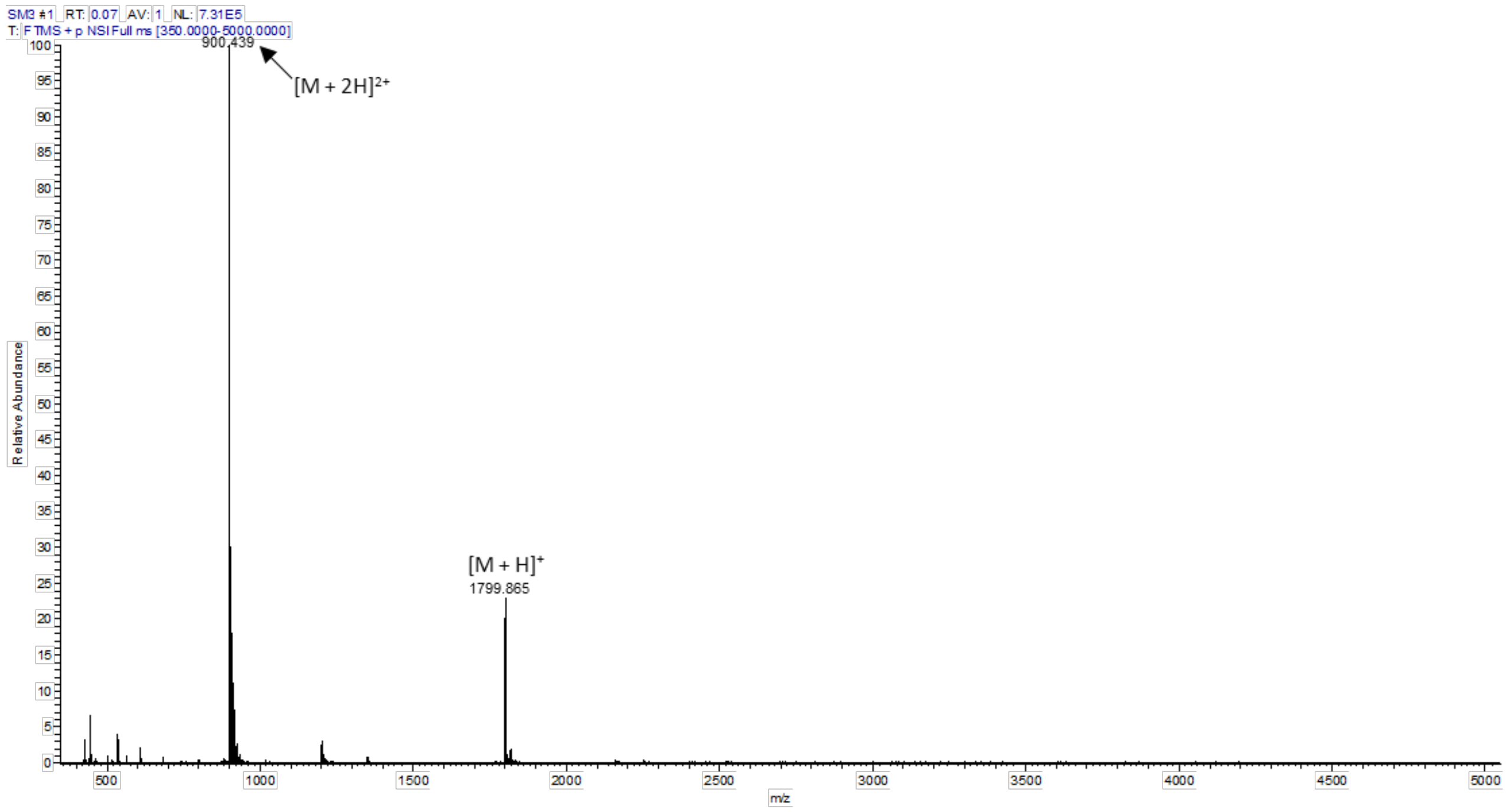

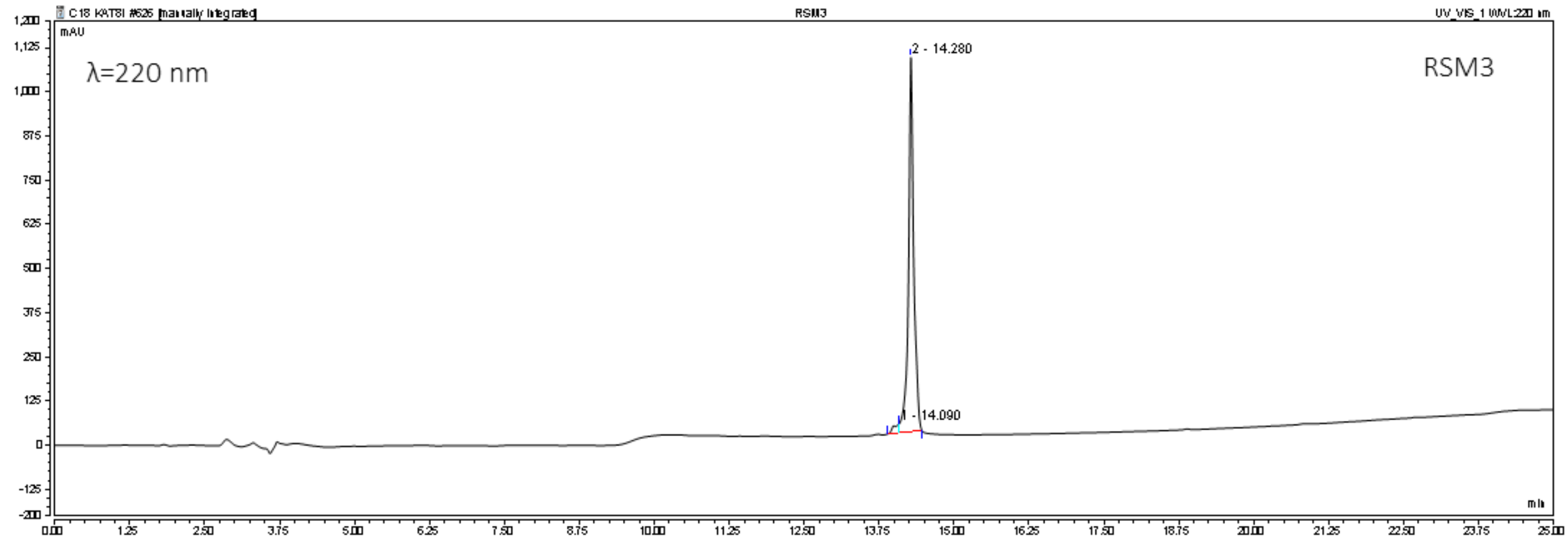

Peak n.	Retention Time (min)	Rel. Area (%)	Area (mAU*min)	Height (mAU)
1	14.09	2.64	2.9155	26.08
2	14.28	97.36	107.4858	1059.34

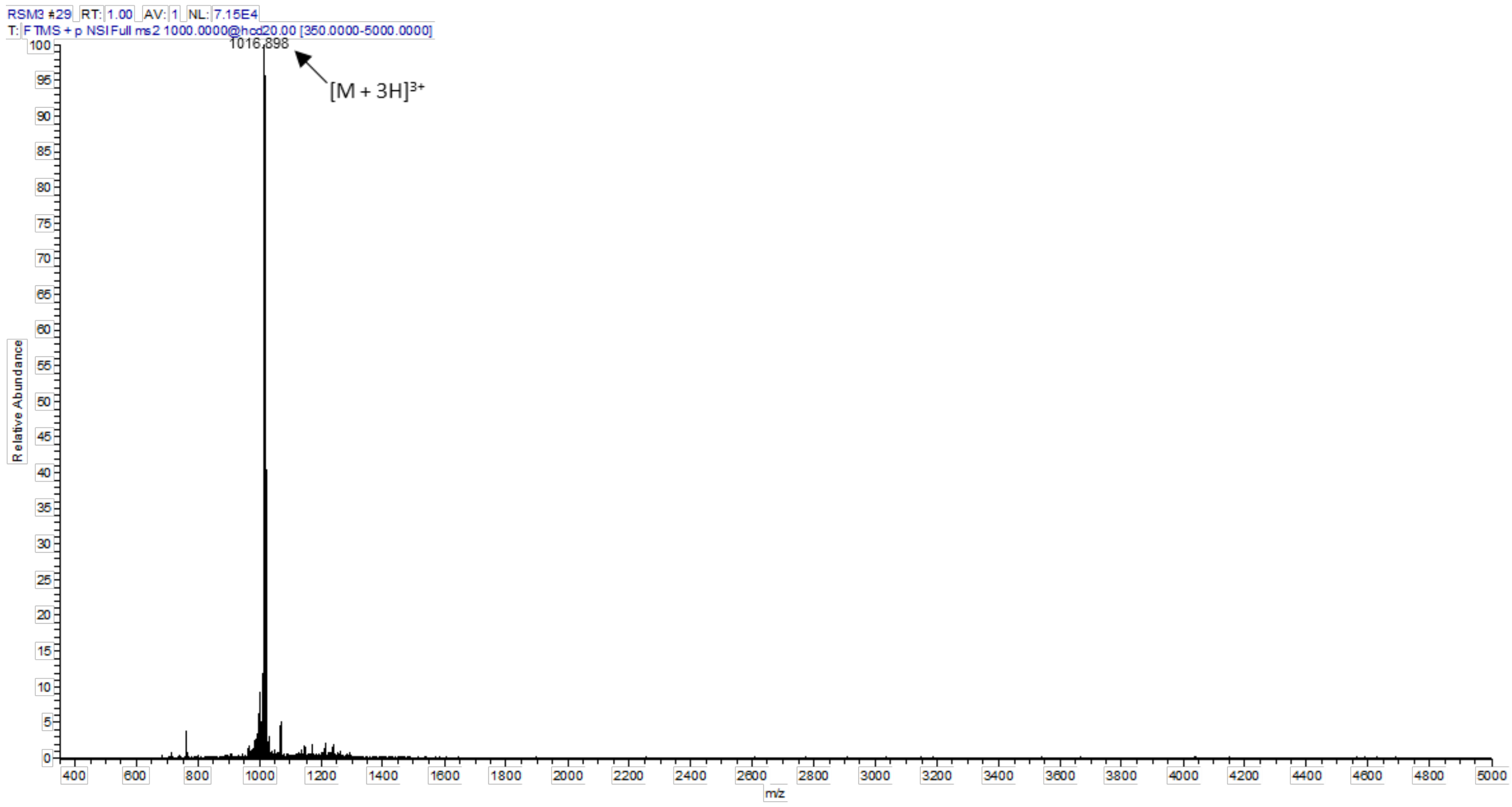

Supporting References

1. Woodcock, C. B.; Yu, D.; Hajian, T.; Li, J.; Huang, Y.; Dai, N.; Corrêa, I. R., Jr.; Wu, T.; Vedadi, M.; Zhang, X.; Cheng, X., Human MettL3-MettL14 complex is a sequence-specific DNA adenine methyltransferase active on single-strand and unpaired DNA in vitro. *Cell Discov* **2019,** *5*, 63.
2. Fiorentino, F.; Bolla, J. R., Mass Spectrometry Analysis of Dynamics and Interactions of the LPS Translocon LptDE. *Methods Mol Biol* **2022,** *2548*, 109-128.
3. Quetschlich, D.; Esser, T. K.; Newport, T. D.; Fiorentino, F.; Shutin, D.; Chen, S.; Davis, R.; Lovera, S.; Liko, I.; Stansfeld, P. J.; Robinson, C. V., NaViA: A program for the visual analysis of complex mass spectra. *Bioinformatics* **2021,** *37* (24), 4876-4878.
4. Marty, M. T.; Baldwin, A. J.; Marklund, E. G.; Hochberg, G. K.; Benesch, J. L.; Robinson, C. V., Bayesian deconvolution of mass and ion mobility spectra: from binary interactions to polydisperse ensembles. *Anal Chem* **2015,** *87* (8), 4370-6.
5. Fiorentino, F.; Sauer, J. B.; Qiu, X.; Corey, R. A.; Cassidy, C. K.; Mynors-Wallis, B.; Mehmood, S.; Bolla, J. R.; Stansfeld, P. J.; Robinson, C. V., Dynamics of an LPS translocon induced by substrate and an antimicrobial peptide. *Nat Chem Biol* **2021,** *17* (2), 187-195.
6. Merlino, F.; Tomassi, S.; Yousif, A. M.; Messere, A.; Marinelli, L.; Grieco, P.; Novellino, E.; Cosconati, S.; Di Maro, S., Boosting Fmoc Solid-Phase Peptide Synthesis by Ultrasonication. *Organic Letters* **2019,** *21* (16), 6378-6382.
7. Li, Z.; Feng, Y.; Han, H.; Jiang, X.; Chen, W.; Ma, X.; Mei, Y.; Yuan, D.; Zhang, D.; Shi, J., A Stapled Peptide Inhibitor Targeting the Binding Interface of N6-Adenosine-Methyltransferase Subunits METTL3 and METTL14 for Cancer Therapy. *Angew Chem Int Ed Engl* **2024,** *63* (24), e202402611.
8. Discovery, C.; Boitreaud, J.; Dent, J.; McPartlon, M.; Meier, J.; Reis, V.; Rogozhnikov, A.; Wu, K., Chai-1: Decoding the molecular interactions of life. *bioRxiv* **2024**, 2024.10.10.615955.
9. McNutt, A. T.; Li, Y.; Meli, R.; Aggarwal, R.; Koes, D. R., GNINA 1.3: the next increment in molecular docking with deep learning. *J Cheminform* **2025,** *17* (1), 28.
10. Jo, S.; Kim, T.; Iyer, V. G.; Im, W., CHARMM-GUI: a web-based graphical user interface for CHARMM. *J Comput Chem* **2008,** *29* (11), 1859-65.
11. Huang, J.; Rauscher, S.; Nawrocki, G.; Ran, T.; Feig, M.; de Groot, B. L.; Grubmüller, H.; MacKerell, A. D., CHARMM36m: an improved force field for folded and intrinsically disordered proteins. *Nature Methods* **2017,** *14* (1), 71-73.
12. Berendsen, H. J. C.; van der Spoel, D.; van Drunen, R., GROMACS: A message-passing parallel molecular dynamics implementation. *Computer Physics Communications* **1995,** *91* (1), 43-56.
13. Del Bene, A.; D'Aniello, A.; Tomassi, S.; Merlino, F.; Mazzarella, V.; Russo, R.; Chambery, A.; Cosconati, S.; Di Maro, S.; Messere, A., Ultrasound-assisted Peptide Nucleic Acids synthesis (US-PNAS). *Ultrasonics Sonochemistry* **2023,** *95*, 106360.
14. Schirle, N. T.; Sheu-Gruttadauria, J.; MacRae, I. J., Structural basis for microRNA targeting. *Science* **2014,** *346* (6209), 608-613.
15. Schirle, N. T.; Sheu-Gruttadauria, J.; Chandradoss, S. D.; Joo, C.; MacRae, I. J., Water-mediated recognition of t1-adenosine anchors Argonaute2 to microRNA targets. *eLife* **2015,** *4*, e07646.
16. Wang, X.; Feng, J.; Xue, Y.; Guan, Z.; Zhang, D.; Liu, Z.; Gong, Z.; Wang, Q.; Huang, J.; Tang, C.; Zou, T.; Yin, P., Structural basis of N6-adenosine methylation by the METTL3–METTL14 complex. *Nature* **2016,** *534* (7608), 575-578.
